# Comparative efficacy and safety of CFTR modulators for people with cystic fibrosis with phe508del mutation: a systematic review and bayesian network meta-analysis

**DOI:** 10.1016/j.eclinm.2025.103655

**Published:** 2025-11-25

**Authors:** Mohammed Safeer V S, Simran Behl, Pankaj C. Vaidya, Pawan Tiwari, Saroj Kundan Bharti, Najiya Nahan, Jitendra Kumar Sahu, Dipika Bansal

**Affiliations:** a Department of Pharmacy Practice and Clinical Research, National Institute of Pharmaceutical Education and Research (NIPER), S.A.S. Nagar, India; b Pediatric Pulmonology Unit, Department of Pediatrics, Postgraduate Institute of Medical Education and Research (PGIMER), Chandigarh, India; c Department of Pulmonary, Critical Care and Sleep Medicine, All India Institute of Medical Sciences (AIIMS), New Delhi, India; d Department of Pediatrics, Postgraduate Institute of Medical Education and Research (PGIMER), Chandigarh, India

**Keywords:** Cystic fibrosis, CFTR modulator, phe508del, Network meta-analysis

## Abstract

**Background:**

The development of cystic fibrosis transmembrane conductance regulator (CFTR) modulators (correctors and potentiators) emerged as a promising approach, aiming to restore CFTR protein function. A lack of head-to-head trials comparing CFTR modulators leaves uncertainty about the optimal treatment. We aimed to evaluate the comparative efficacy and safety of CFTR modulators for people with cystic fibrosis who have a phe508del mutation.

**Methods:**

We conducted an extensive literature search for both published and unpublished randomized controlled trials in databases such as PubMed, EMBASE, Scopus, Ovid, Cochrane Central Register of Controlled Trials, and international trial registers from inception until May 21, 2025. We included studies that used any CFTR modulators (monotherapy or combination) for the treatment of children and adults with a confirmed diagnosis of cystic fibrosis with phe508del CFTR mutation. Two reviewers independently and in duplicate performed study selection, data extraction, and quality assessment. Our primary outcomes were efficacy (change in percent predicted forced expiratory volume (ppFEV_1_), sweat chloride) and safety (frequency of serious adverse events). We performed a random effect bayesian network meta-analysis for each outcome using the gemtc and BUGSnet package in R. The confidence in the network meta-analysis framework was utilized to determine the certainty of evidence. The study protocol was registered with Prospective Register of Systematic Reviews (CRD42024505081).

**Findings:**

Of the 3473 studies identified through our literature search, 29 studies involving 6450 patients examining 34 treatment combinations were included. For adults treated over 4–8 weeks, vanzacaftor 10 mg-tezacaftor 100 mg-deutivacaftor 150 mg combination therapy had a significant improvement over placebo in improving ppFEV_1_ (MD: 15.9; 95% CrI: 7.2–24.2 [high certainty]) with a SUCRA of 92% suggesting the highest probability of effectiveness. Moreover, the vanzacaftor 20 mg-tezacaftor 100 mg-deutivacaftor 150 mg showed a significant reduction in sweat chloride levels (MD: −49.3 mmol/L; 95% CrI: −67.2 to −31.7 [high certainty]) and improved the CFQ-R scores (MD: 39; 95% CrI: 21.2–56.9; [high certainty]) when compared to placebo after 4–8 weeks of treatment. Our findings also highlighted that the triple combination therapies of vanzacaftor 20 mg-tezacaftor 100 mg-deutivacaftor 250 mg and elexacaftor 200 mg-tezacaftor 100 mg-ivacaftor 150 mg provided clinically meaningful improvements across all measured outcomes in adults treated for more than 8 weeks. Confidence in the estimates ranged from high to low, and safety analyses were limited by the low serious adverse event rates.

**Interpretation:**

Our findings indicate that vanzacaftor-tezacaftor-deutivacaftor and elexacaftor-tezacaftor-deutivacaftor emerged as the most effective treatment options in adults. However, these results should be interpreted cautiously due to limited data and the low quality of existing evidence.

**Funding:**

None.

**Research in context**

**Evidence before this study**

**C**ystic fibrosis (CF) is a life-limiting, multiorgan genetic disorder caused by mutations in the CFTR gene, with phe508del mutation being the most prevalent. Despite the development of several CFTR modulator combinations, the differences in their efficacy and safety remain unclear. We searched PubMed (Dec 15, 2023), without language restrictions using the search terms (((cystic fibrosis)) AND (“CFTR modulators” OR ivacaftor OR tezacaftor OR vanzacaftor OR elexacaftor OR deutivacaftor OR lumacaftor)) AND filter “Article type: Meta-analysis” and identified 12 results. We identified outdated pairwise meta-analyses that examined individual interventions (elexacaftor-tezacaftor-ivacaftor), except a Cochrane review that evaluated the efficacy and safety of CFTR modulators in CF patients.

**Added value of this study**

To the best of our knowledge, this is the first network meta-analysis that offers a comprehensive overview of the available evidence on the comparative efficacy and safety of CFTR modulators in CF patients with the phe508del mutation. Our findings indicate that triple combination of vanzacaftor-tezacaftor-deutivacaftor and elexacaftor-tezacaftor-ivacaftor as the most effective treatment options in adults. Additionally, we conducted a subgroup network meta-analysis, which provided separate evidence for homozygous and heterozygous genotypes.

**Implications of all the available evidence**

Our study offers a comprehensive comparative evidence on the effects of CFTR modulators for CF in adults. Our findings suggest that the triple combinations elexacaftor-tezacaftor-ivacaftor and vanzacaftor-tezacaftor-deutivacaftor are the most effective when considering treatment options for adult patients with CF carrying the phe508del mutation.

## Introduction

Cystic fibrosis (CF) is a multiorgan genetic disorder caused by a deficiency or malfunction of the CF transmembrane conductance regulator (CFTR) protein, leading to a reduced life expectancy of roughly 40 years due to the gradual deterioration of lung function. It affects more than 92,000 individuals globally with a median survival age of 44–53 years and a median age at death between 29 and 35.6 years.^1^, ^2^, ^3^, ^4^ With over 2000 mutations identified in the CFTR gene, phe508del is the most prevalent,^5^ with approximately 90% of patients carrying at least one copy of this mutation and nearly 50% carrying two copies.^6^ Individuals with two copies of the phe508del mutation (F/F genotype) typically present with a severe multisystem form of the disease, marked by progressive decline in lung function.^7^, ^8^, ^9^, ^10^

The development of small-molecule CFTR modulator (CFTRm) therapies have emerged as a promising approach to restoring CFTR protein dysfunction.^11^^,^^12^ CFTRm include correctors (e.g., elexacaftor, tezacaftor, and lumacaftor) and potentiators (e.g., ivacaftor, deutivacaftor). CFTR correctors improve folding and trafficking of the misfolded phe508del CFTR protein to cell surface while potentiators increase the probability of channel opening at cell surface, thereby improving chloride transport.^13^ Registry data from the US and UK revealed that the first approved CFTRm therapies, ivacaftor, and lumacaftor–ivacaftor, have slowed CF disease progression compared to untreated individuals.^14^, ^15^, ^16^, ^17^ In 2019, the triple therapy elexacaftor–tezacaftor–ivacaftor (Trikafta)^18^ was approved for patients ≥12 years with at least one phe508del allele, demonstrating efficacy and safety in phase 3 trials. Surpassing earlier CFTRm treatments, the triple combination significantly improves respiratory symptoms (assessed by cystic fibrosis questionnaire revised (CFQ-R) respiratory domain), lung function (assessed by ppFEV_1_), and CFTR activity (assessed by sweat chloride concentration).^19^^,^^20^

These advances have raised questions regarding the comparative efficacy and safety of various CFTRm. Previous meta-analyses including a Cochrane review^21^, ^22^, ^23^ could not answer these questions, due to a lack of direct comparisons between treatment combinations. Therefore, to ascertain the most effective combinations, we utilized a network meta-analysis (NMA) technique which integrates direct evidence from head-to-head trials and indirect evidence derived from comparisons through a common comparator. We conducted a NMA of randomized controlled trials (RCTs) to evaluate the relative efficacy and safety of CFTRm in patients with CF carrying at least one phe508del mutation.

## Methods

### Search strategy and selection criteria

We conducted an extensive literature search for both published and unpublished RCTs in databases such as PubMed, Scopus, Ovid, EMBASE, Cochrane Central Register of Controlled Trials, and international trial registers (ClinicalTrials.gov, Australian New Zealand Clinical Trials Registry, World Health Organization International Clinical Trials Registry Platform, and the EU Clinical Trials Register) from inception until May 21, 2025. The search terms utilized a comprehensive set of restricted vocabulary phrases (MeSH and EMTREE) in different combinations, along with additional keywords such as cystic fibrosis, pulmonary fibrosis, and CFTR modulators (Appendix p 3–4). No language or date restrictions were imposed. We also conducted a thorough check of the reference lists of the articles that were included and sought input from experts to identify any additional studies.

Eligible studies included any CFTR modulators (monotherapy or combination) for the treatment of children and adults (6 years or older when enrolled in the trials) with a confirmed diagnosis of CF with phe508del CFTR mutation (homozygous or heterozygous) and a forced expiratory volume (FEV_1_) of at least 40% of predicted in the first second. Studies that were not RCTs, used other comparators (other than CFTR modulators), involved nonhuman participants, or had a total sample size of less than 10 patients were excluded from the study. We did not impose any limitation on the kind of RCTs, but for crossover RCTs, we only considered outcome data before the crossover.

Two reviewers (M.S. and S.B) independently and in duplicate screened titles and abstracts, retrieved full texts of articles that either reviewer considered potentially eligible and determined eligibility from the full texts. Consensus or a third adjudicator (D.B.) was employed to resolve all the discrepancies. The same two review authors independently extracted information from each eligible study using a pilot-tested standardized form. The data extracted included information on the study and population characteristics, as well as the treatments and outcomes (ppFEV1, sweat chloride concentration, CFQ-R, and SAEs).

For effectiveness outcomes, we extracted relevant data such as the mean change from the baseline along with a measure of variance. When change from baseline data was not reported, we estimated the mean change using the baseline and follow-up mean values. The corresponding standard deviation was calculated assuming a correlation coefficient of 0.5 between baseline and follow-up measurements, based on the guidance from the Cochrane Handbook for Systematic Reviews of Interventions. For SAEs, we extracted information on the number of individuals who experienced the events and the total number included in the analysis. When results were presented only graphically without exact numerical values, we extracted data using Engauge Digitizer software. Two reviewers (M.S. and S.B.) independently derived estimates, and we used the mean of their estimates. The risk of bias in individual studies was assessed using the Cochrane Risk of Bias (ROB-2) tool.^24^ Assessments were conducted in duplicate (M.S. and either S.B. or S.K.), under the supervision of D.B., with discrepancies resolved through discussion.

### Statistics

The primary efficacy outcomes were absolute change in ppFEV_1_, sweat chloride concentration from baseline; and safety outcome included the frequency of serious adverse events (SAEs). The secondary outcome was a change in respiratory-specific health-related quality of life score, assessed using CFQ-R.^25^

We conducted an NMA for each outcome using a bayesian framework implemented in R (version 3.6), utilizing the gemtc and BUGSnet packages.^26^ For the bayesian approach, we applied a random-effects model with default prior heterogeneity parameters provided in the BUGSnet package. The model utilizes a generalized linear model with an identity link for continuous outcomes and a logit link for binary outcomes. Markov chain Monte Carlo (MCMC) simulations were conducted with a burn-in of 10,000 iterations, followed by 100,000 iterations in each of the three chains. The convergence of the three chains was assessed using the Gelman-Rubin statistic (Appendix p 5) and by visual inspection of density and trace plots.

We evaluated the inconsistency in the network using the global inconsistency and node-splitting methods. Global inconsistency was evaluated by comparing the fit of consistency and inconsistency models, while the node-splitting approach separately contrasted direct and indirect evidence for specific comparisons (nodes).^27^^,^^28^ We created network plots, league tables, and forest plots for each outcome, alongside estimating the overall treatment rankings by calculating the surface under the cumulative ranking curve (SUCRA) for each outcome.^29^ The SUCRA value ranges from 0% to 100%, with higher values indicating better treatment and lower indicating worse treatments.

We synthesized both direct and indirect evidence to compare treatments based on efficacy and safety, reporting mean differences (MD) for continuous outcomes such as ppFEV_1_, sweat chloride concentration, and CFQ-R, and log odds ratio (log OR) for the binary outcome SAEs, accompanied by corresponding 95% credible intervals (CrI). Our analyses separately pooled studies with treatment durations of 4–8 weeks from those longer than 8 weeks. We distinguished trials involving adults from those with children but included trials with participants aged 12 years and older in the adult analysis. Given that ivacaftor monotherapy is ineffective in individuals homozygous for the F508del mutation, a sensitivity analysis was conducted excluding the ivacaftor trial (Flume et al., 2012) across all outcomes, in order to examine whether its inclusion introduced bias into the overall treatment effect estimates. Subgroup NMA was further performed to differentiate trials involving participants who were homozygous or heterozygous for the phe508del CFTR mutation. Additionally, we employed the Confidence in Network Meta-Analysis (CINeMA) framework^30^^,^^31^ to evaluate the confidence of evidence across the six domains: within-study bias, reporting bias, indirectness, imprecision, heterogeneity, and incoherence. Our analysis adhered to the guidelines of the Preferred Reporting Items for Systematic Reviews and Meta-Analyses (PRISMA), including its extension statement for NMA.^32^^,^^33^ The protocol for this NMA was registered in the Prospective Register of Systematic Reviews (PROSPERO CRD42024505081).

### Ethics

This study used only publicly available data, and therefore did not require ethics committee approval.

### Role of funding source

There was no funding source for this study.

## Results

**Fig. 1:**
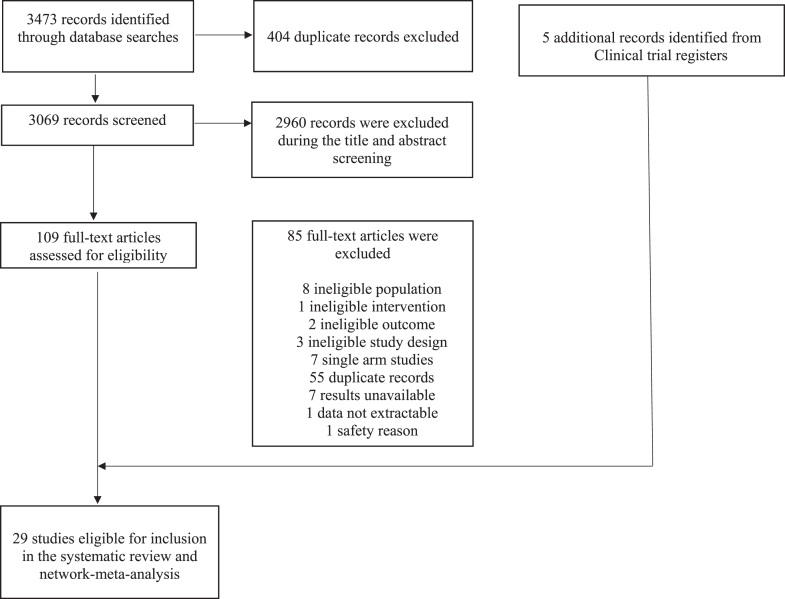
**Study selection process**.

Our literature search identified 3473 citations (3069 after removal of duplicates). After screening titles and abstracts, 2960 were excluded as ineligible, leaving 109 studies and five additional studies from clinical trial registers for full-text review. Finally, 29 eligible studies were included in the systematic review and NMA (Fig. 1).

Table 1 summarizes the characteristics of the 29 studies involving 6450 patients. These studies were published from 2011 to 2025 and were conducted multicentre across multiple countries with most studies being sponsored by the industries (i.e., Vertex Industries). The median study sample size was 121 (range, 22–1108 patients) and around half of the total participants (2989 [46.3%]) were males. Three trials^40^^,^^55^^,^^57^ enrolled only children (6–11 years); 11^34^^,^^36^^,^^38^^,^^42^^,^^44^^,^^46^, ^47^, ^48^, ^49^, ^50^^,^^58^ enrolled adults (18 years or old) and the remainder enrolled both^17^^,^^19^^,^^20^^,^^37^^,^^39^^,^^41^^,^^43^^,^^45^^,^^51^, ^52^, ^53^, ^54^^,^^56^^,^^59^ adolescents and adults (12 years or old). The median duration of the treatment period was 8 weeks (range, 4–96 weeks). 13 studies^17^^,^^20^^,^^34^^,^^35^^,^^37^^,^^40^, ^41^, ^42^, ^43^^,^^48^^,^^50^^,^^52^^,^^56^ included patients homozygous (F/F) for phe508del mutation; seven studies^19^^,^^44^^,^^45^^,^^51^^,^^53^^,^^54^^,^^57^ included heterozygous genotype while nine studies^36^^,^^38^^,^^39^^,^^46^^,^^47^^,^^49^^,^^55^ included both genotype patients.

**Table 1:** Study Characteristics of the included studies.

Author (Publication year)	Age group (years)	Sample Size (n)	Male (n)	Female (n)	Genotype	Follow-up duration (weeks)	Treatments	Outcomes assessed	Risk of bias
Clancy et al. (2011)^34^	18+	89	53	36	Homozygous	4	Luma 25 mg, Luma 50 mg, Luma 100 mg, Luma 200 mg, Placebo	ppFEV_1_, Sweat chloride, CFQ-R, SAE	Moderate
Flume et al. (2012)^35^	12+	140	74	66	Homozygous	16	Iva 150 mg, Placebo	ppFEV_1_, Sweat chloride, CFQ-R, SAE	Low
Boyle et al. (2014)^36^	18+	154	83	71	Mixed	8	Luma 200 mg + Iva 150 mg, Luma 200 mg + Iva 250 mg, Luma 400 mg + Iva 250 mg, Luma 600 mg + Iva 250 mg, Placebo	ppFEV_1_, Sweat chloride, CFQ-R, SAE	Low
Wainwright et al. (2015)^37^	12+	1108	563	545	Homozygous	24	Luma 400 mg + Iva 250 mg, Luma 600 mg + Iva 250 mg, Placebo	ppFEV1, CFQ-R, SAE	Low
NCT02951195 (2016)^38^	18+	76	37	39	Mixed	4	VX-152 100 mg + Teza 100 mg + Iva 150 mg, VX-152 200 mg + Teza 100 mg + Iva 150 mg, VX-152 300 mg + Teza 100 mg + Iva 150 mg, Teza 100 mg + Iva 150 mg, Placebo	ppFEV1, Sweat chloride, CFQ-R, SAE	Low
NCT02951182 (2016)^39^	12+	73	47	26	Mixed	4	Ola 200 mg + Teza 100 mg + Iva 150 mg, Ola 200 mg + Teza 50 mg + Iva 150 mg, Ola 600 mg + Teza 50 mg + Iva 300 mg, Teza 50 mg + Iva 300 mg, Placebo	ppFEV1, Sweat chloride, CFQ-R, SAE	Low
Ratjen et al. (2017)^40^	6 to 11	204	83	121	Homozygous	24	Luma 200 mg + Iva 250 mg, Placebo	ppFEV_1_, Sweat chloride, CFQ-R, SAE	Low
Taylor et al. (2017)^41^	12+	510	264	246	Homozygous	24	Teza 100 mg + Iva 150 mg, Placebo	ppFEV_1_, Sweat chloride, CFQ-R, SAE	Low
NCT02508207 (2017)^42^	18+	34	15	19	Homozygous	4	Teza 100 mg + Iva 150 mg, Placebo	ppFEV_1_, Sweat chloride, SAE	Low
NCT03150719 (2017)^43^	12+	97	36	61	Homozygous	8	Teza 100 mg + Iva 150 mg, Placebo	ppFEV1, CFQ-R, SAE	Low
Rowe et al. (2017)^44^	18+	125	65	60	Heterozygous	8	Luma 400 mg + Iva 250 mg, Placebo	ppFEV_1_, Sweat chloride, CFQ-R, SAE	Moderate
Rowe et al. (2017)^45^	12+	244	120	134	Heterozygous	8	Teza 100 mg + Iva 150 mg, Iva 150 mg, Placebo	ppFEV1, Sweat chloride, CFQ-R, SAE	Low
Konstan et al. (2017)^17^	12+	664	NR	NR	Homozygous	96	Luma 400 mg + Iva 250 mg, Luma 600 mg + Iva 250 mg	ppFEV1, CFQ-R, SAE	Low
Keating et al. (2018)^46^	18+	93	57	36	Mixed	4	Elexa 50 mg + Teza 100 mg + Iva 150 mg, Elexa 100 mg + Teza 100 mg + Iva 150 mg, Elexa 200 mg + Teza 100 mg + Iva 150 mg, Teza 100 mg + Iva 150 mg, Placebo	ppFEV_1_, Sweat chloride, CFQ-R, SAE	Low
Donaldson et al. (2018)^47^	18+	67	38	29	Mixed	4	Teza 100 mg + Iva 150 mg, Teza 100 mg + Iva 50 mg, Teza 50 mg + Iva 150 mg, Placebo	ppFEV_1_, Sweat chloride, SAE	Moderate
NCT02070744 (2018)^48^	18+	39	25	14	Homozygous	12	Teza 50 mg + Iva 150 mg, Teza 100 mg + Iva 150 mg, Placebo	ppFEV1, Sweat chloride, CFQ-R, SAE	Moderate
Heijerman et al. (2019)^20^	12+	107	48	59	Homozygous	4	Elexa 200 mg + Teza 100 mg + Iva 150 mg, Teza 100 mg + Iva 150 mg	ppFEV1, Sweat chloride, CFQ-R, SAE	Low
Middleton et al. (2019)^19^	12+	403	209	194	Heterozygous	24	Elexa 200 mg + Teza 100 mg + Iva 150 mg, Placebo	ppFEV1, Sweat chloride, CFQ-R, SAE	Low
Bell et al. (2019)^49^	18+	96	55	41	Mixed	4	GLPG2222 50 mg, GLPG2222 100 mg, GLPG2222 150 mg, GLPG2222 200 mg, GLPG2222 300 mg, GLPG2222 400 mg, Placebo	ppFEV1, Sweat chloride, CFQ-R, SAE	Low
Koningsbruggen et al. (2020)^50^	18+	22	14	8	Homozygous	4	GLPG2737 75 mg, Placebo	ppFEV1, Sweat chloride, CFQ-R, SAE	Low
Munck et al. (2020)^51^	12+	168	87	81	Heterozygous	12	Teza 100 mg + Iva 150 mg, Placebo	ppFEV1, Sweat chloride, CFQ-R, SAE	Moderate
Schwarz et al. (2021)^52^	12+	97	36	61	Homozygous	8	Teza 100 mg + Iva 150 mg, Placebo	ppFEV1, CFQ-R, SAE	Low
McKone et al. (2021)^53^	12+	150	84	66	Heterozygous	8	Teza 100 mg + Iva 150 mg, Iva 150 mg	ppFEV1, Sweat chloride, CFQ-R, SAE	Moderate
Barry et al. (2021)^54^	12+	258	130	128	Heterozygous	8	Elexa 200 mg + Teza 100 mg + Iva 150 mg, Teza 100 mg + Iva 150 mg, Iva 150 mg	ppFEV1, Sweat chloride, CFQ-R, SAE	Low
Davies et al. (2021)^55^	6 to 11	67	30	37	Mixed	8	Teza 100 mg + Iva 150 mg, Iva 150 mg, Placebo	ppFEV1, Sweat chloride, CFQ-R, SAE	Moderate
Sutharsan et al. (2022)^56^	12+	175	87	88	Homozygous	24	Elexa 200 mg + Teza 100 mg + Iva 150 mg, Teza 100 mg + Iva 150 mg	ppFEV1, Sweat chloride, CFQ-R, SAE	Low
Mall et al. (2022)^57^	6 to 11	121	51	70	Heterozygous	24	Elexa 200 mg + Teza 100 mg + Iva 150 mg, Placebo	ppFEV1, Sweat chloride, CFQ-R, SAE	Moderate
Uluer et al. (2023)^58^	18+	98	70	28	Mixed	4	Vanza 5 mg + Teza 100 mg + Deuti 150 mg, Vanza 10 mg + Teza 100 mg + Deuti 150 mg, Vanza 20 mg + Teza 100 mg + Deuti 150 mg, Vanza 5 mg + Teza 100 mg + Iva 150 mg, Teza 100 mg + Iva 150 mg, Placebo	ppFEV1, Sweat chloride, CFQ-R	Low
Keating et al. (2025)^59^	12+	971	528	443	Mixed	24, 52	Vanza 20 mg + Teza 100 mg + Deuti 250 mg, Elexa 200 mg + Teza 100 mg + Iva 150 mg	ppFEV1, Sweat chloride, CFQ-R, SAE	Low

Mixed: Involves patients with both the genotypes; Elexa: elexacaftor; Teza: Tezacaftor; Iva: Ivacaftor; Luma: Lumacaftor; Deuti: Deutivacaftor; Vanza: Vanzacaftor; Ola: Olaxacaftor; ppFEV_1_: percentage predicted forced expiratory volume; CFQ-R: Cystic fibrosis questionnaire revised; SAE: Serious adverse event.

**Fig. 2:**
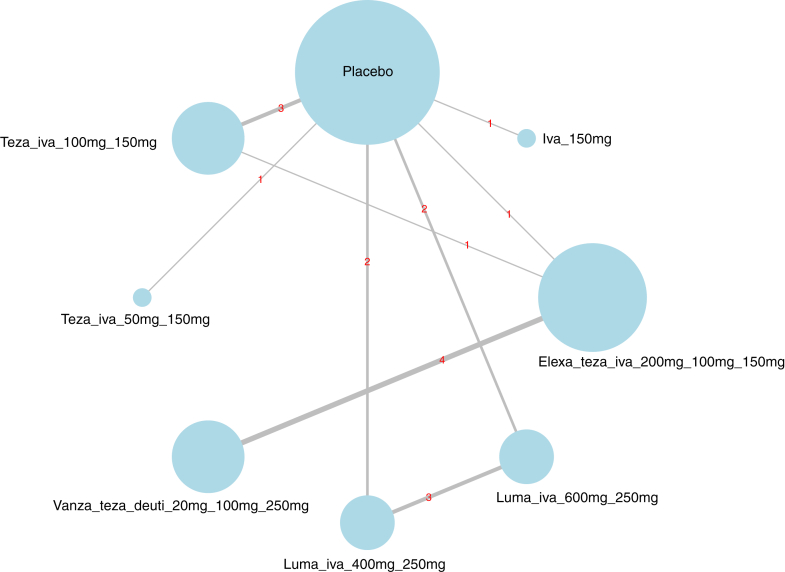
**Network plot of ppFEV1 in adults treated for greater than 8 weeks.** Line thickness represents the number of trials evaluating each comparison, while node size reflects the number of trials investigating each intervention. Elexa: elexacaftor; Teza: Tezacaftor; Iva: Ivacaftor; Luma: Lumacaftor; Deuti: Deutivacaftor; Vanza:Vanzacaftor

In terms of study quality, nine trials (31%) were assessed as having a moderate risk of bias, whereas 20 trials (69%) were assessed as having a low risk of bias (Appendix p 138). A total of 34 CFTRm therapies (excluding placebo) were compared across the studies. Fig. 2 illustrates the network geometry of ppFEV_1_ in adults treated for greater than 8 weeks. The networks for other efficacy and safety outcomes are displayed in the Supplementary file (Appendix p 139–149). In our network graphs, we observed placebo connected to multiple nodes, links between nodes representing different doses of the same combinations, and limited direct connections between different CFTR modulators.

For inconsistency assessment, the consistency model demonstrated a fit comparable to or greater than the inconsistency model (Appendix p. 6–7). Node-splitting analysis revealed no significant differences between direct and indirect estimates for most comparisons, with exceptions detailed in Appendix p. 8–11. We included three RCTs in children, each evaluating a different CFTR modulator. As the number of studies were very limited, a NMA was not conducted, since it would not provide reliable estimates.

Concerning adults treated for a duration of 4–8 weeks, we included 17 trials involving 2477 patients, comparing 33 treatment combinations in the ppFEV_1_ analysis. The triple combination of vanzacaftor 10 mg-tezacaftor 100 mg-deutivacaftor 150 mg (MD: 15.9; 95% CrI: 7.2–24.2 [high certainty]), vanzacaftor 20 mg-tezacaftor 100 mg-deutivacaftor 150 mg (MD: 13.7; 95% CrI: 6.9–20.3 [high certainty]), elexacaftor 200 mg-tezacaftor 100 mg-ivacaftor 150 mg (MD: 12.0; 95% CrI: 8.9–14.9 [high certainty]), and VX152 200 mg-tezacaftor 100 mg-ivacaftor 150 mg (MD: 11.7; 95% CrI: 5.3–18.0 [high certainty]) showed a significant improvement in ppFEV_1_ compared to placebo. Based on SUCRA values, vanzacaftor 10 mg-tezacaftor 100 mg-deutivacaftor 150 mg (92%), vanzacaftor 20 mg-tezacaftor 100 mg-deutivacaftor 150 mg (88%), elexacaftor 200 mg-tezacaftor 100 mg-ivacaftor 150 mg (85%), and VX152 200 mg-tezacaftor 100 mg-ivacaftor 150 mg (83%) were the four treatment that had the highest probability of effectiveness in improving ppFEV_1_. For sweat chloride analysis, we identified a total of 15 trials (1797 patients) comparing 32 treatment combinations. Compared with placebo, vanzacaftor 20 mg-tezacaftor 100 mg-deutivacaftor 150 mg (MD: −49.3 mmol/L; 95% CrI: −67.2 to −31.7 [high certainty]) showed a significant reduction in sweat chloride levels followed by vanzacaftor 5 mg-tezacaftor 100 mg-ivacaftor150 mg (MD: −50.2 mmol/L; 95% CrI: −80.5 to −19.8 [high certainty]), and vanzacaftor 10 mg-tezacaftor 100 mg-deutivacaftor 150 mg (MD: −46.6 mmol/L; 95% CrI: −69.4 to −23.8 [high certainty]). The SUCRA analysis suggested that vanzacaftor 20 mg-tezacaftor 100 mg-deutivacaftor 150 mg (94%), vanzacaftor 5 mg-tezacaftor 100 mg-ivacaftor150 mg (92%), and vanzacaftor 10 mg-tezacaftor 100 mg-deutivacaftor 150 mg (91%) is likely best treatment for reducing the sweat chloride levels. In terms of CFQ-R score improvement, vanzacaftor 20 mg-tezacaftor 100 mg-deutivacaftor 150 mg (MD: 39; 95% CrI: 21.2–56.9; SUCRA: 97% [high certainty]), elexacaftor 200 mg-tezacaftor 100 mg-ivacaftor 150 mg (MD: 29.9; 95% CrI: 16.4–43.6; SUCRA: 86% [high certainty]), and VX152 200 mg-tezacaftor 100 mg-ivacaftor 150 mg (MD: 30.4; 95% CrI: 13.0–47.9; SUCRA: 85% [moderate certainty]) showed a significant improvement compared to placebo.

**Fig. 3:**
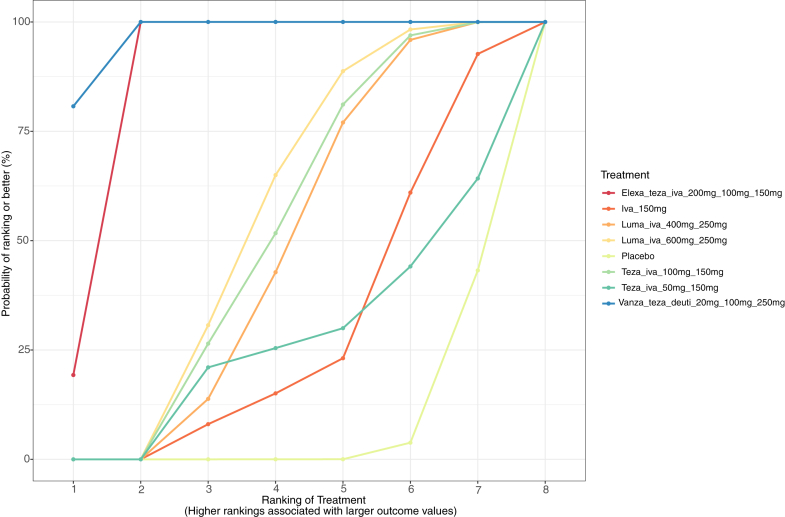
**SUCRA plot of ppFEV1 in adults treated for more than 8 weeks.** A higher SUCRA percentage indicates a higher likelihood of being among the most effective treatments in improving ppFEV1. Elexa: elexacaftor; Teza: Tezacaftor; Iva: Ivacaftor; Luma: Lumacaftor; Deuti: Deutivacaftor; Vanza:Vanzacaftor

For adults treated for more than 8 weeks, we included nine trials involving 4209 patients and eight treatment combinations in the ppFEV_1_ analysis. Overall, vanzacaftor 20 mg-tezacaftor 100 mg-deutivacaftor 250 mg (MD: 14.0; 95% CrI: 12.2–15.7 [high certainty] Table 2); and elexacaftor 200 mg-tezacaftor 100 mg-ivacaftor 150 mg (MD: 13.8; 95% CrI: 12.1–15.4; [high certainty]) showed a significant improvement in ppFEV_1_ compared to placebo. Based on the SUCRA values, vanzacaftor 20 mg-tezacaftor 100 mg-deutivacaftor 250 mg (97%; Fig. 3) and elexacaftor 200 mg-tezacaftor 100 mg-ivacaftor 150 mg (88%) had the highest probability of effectiveness in improving ppFEV_1_. These two regimens also showed significant reductions in sweat chloride levels and improvements in CFQ-R respiratory domain scores. In the sensitivity analysis excluding the ivacaftor monotherapy trial (Flume et al., 2012), the overall treatment effect estimates remained consistent with those of the primary analysis. Additional details in terms of league tables, forest plots, SUCRA values, and plots for all the outcomes are detailed in Appendix p 12–26, p 150–177.

**Table 2:** League table of the ppFEV_1_ in adults treated for greater than 8 weeks.

Vanza_teza_deuti_20 mg_100 mg_250 mg							
*0.2 (−0.5, 0.9)*	Elexa_teza_iva_200 mg_100 mg_150 mg						
**∗∗10.8∗∗ (8.5, 13.0)**	**∗∗10.6∗∗ (8.5, 12.7)**	Luma_iva_600 mg_250 mg					
**∗∗11.0∗∗ (9.2, 12.8)**	**∗∗10.8∗∗ (9.1, 12.5)**	*0.2 (−1.5, 2.0)*	Teza_iva_100 mg_150 mg				
**∗∗11.1∗∗ (8.8, 13.2)**	**∗∗10.9∗∗ (8.7, 12.9)**	*0.2 (−0.9, 1.4)*	*0.1 (−1.8, 1.7)*	Luma_iva_400 mg_250 mg			
**∗∗12.3∗∗ (9.1, 15.5)**	**∗∗12.1∗∗ (8.9, 15.2)**	*1.5 (−1.5, 4.5)*	*1.4 (−1.7, 4.2)*	*1.3 (−1.7, 4.3)*	Iva_150 mg		
**∗∗13.0∗∗ (6.7, 19.2)**	**∗∗12.8∗∗ (6.5, 19.0)**	*2.1 (−4.1, 8.3)*	*2.0 (−4.2, 8.1)*	*1.9 (−4.4, 8.1)*	*0.7 (−6.0, 7.3)*	Teza_iva_50 mg_150 mg	
**∗∗14.0∗∗ (12.2, 15.7)**	**∗∗13.8∗∗ (12.1, 15.4)**	**∗∗3.2∗∗ (1.9, 4.5)**	**∗∗3.1∗∗ (1.8, 4.1)**	**∗∗3.0∗∗ (1.6, 4.3)**	*1.7 (−1.0, 4.4)*	*1.1 (−5.0, 7.2)*	Placebo

Results from the network meta-analysis are presented in the cells and significant results are presented in ∗∗,∗∗. Relative treatment effects are measured by mean difference along with their 95% CrIs. The font style in the lower triangle represent the confidence in the estimate results obtained with CINeMA: bold indicates high confidence, and italic indicates low confidence.

Elexa: elexacaftor; Teza: Tezacaftor; Iva: Ivacaftor; Luma: Lumacaftor; Deuti: Deutivacaftor; Vanza:Vanzacaftor.

Due to the low incidence of serious adverse event rates, many studies reported zero events for one or more treatments, leading to results that are either non-estimable or characterized by high uncertainty. For the adults treated for greater than 8 weeks, tezacaftor 50 mg-ivacaftor 150 mg (log OR: −1.5; 95% CrI: −5.0 to 1.5; [low certainty]) and vanzacaftor 20 mg-tezacaftor 100 mg-deutivacaftor 250 mg (log OR: −0.8; 95% CrI: −1.9 to 0.2; [low certainty]) was associated with fewer SAEs than placebo. However, neither of these results reached statistical significance. Certainty in the estimates (assessed by CINeMA) ranged from high to low for all the outcomes and is detailed in Appendix p 27–137.

The subgroup NMA was conducted to identify the best treatment options in patients with the homozygous (F/F) and heterozygous genotypes. The treatments that offered the greatest benefits varied between the two subgroups. In terms of ppFEV_1_, vanzacaftor 20 mg-tezacaftor 100 mg-deutivacaftor 150 mg (MD: 19.7; 95% CrI: 11.2–28.4; SUCRA: 97%) showed superiority over placebo in homozygous subgroup while elexacaftor 200 mg-tezacaftor 100 mg-ivacaftor 150 mg (MD: 12.2; 95% CrI: 8.8–15.5; SUCRA: 85%) and vanzacaftor 20 mg-tezacaftor 100 mg-deutivacaftor 150 mg (MD: 12.4; 95% CrI: 6.5–18.1; SUCRA: 84%) showed superiority than placebo in the heterozygous subgroup. In terms of sweat chloride and CFQ-R score, both vanzacaftor 20 mg-tezacaftor 100 mg-deutivacaftor 150 mg and elexacaftor 200 mg-tezacaftor 100 mg-ivacaftor 150 mg was found to be superior in homozygous subgroup while only vanzacaftor 20 mg-tezacaftor 100 mg-deutivacaftor 150 mg was found to be beneficial in the heterozygous subgroup compared to placebo.

## Discussion

To the best of our knowledge, we conducted the first systematic review and NMA investigating the comparative efficacy and safety of CFTRm therapies in CF patients who have a phe508del mutation. We included data from 29 RCTs involving 6450 patients and 34 treatment combinations at different doses (excluding placebo). While three of these trials were conducted in children, an NMA was not performed in this subgroup due to the limited number of studies. For adults, the triple combinations of vanzacaftor 20 mg-tezacaftor 100 mg-deutivacaftor 150 mg, vanzacaftor 10 mg-tezacaftor 100 mg-deutivacaftor 150 mg, vanzacaftor 20 mg-tezacaftor 100 mg-deutivacaftor 250 mg and elexacaftor 200 mg-tezacaftor 100 mg-ivacaftor 150 mg showed clinically meaningful improvements across all the outcomes with high certainty of evidence. Subgroup analyses further revealed that these triple combinations demonstrated superiority in both homozygous and heterozygous genotypes. However, the clinical interpretation of these findings is limited by the small number of trials in each node and the low certainty of evidence assessed by CINeMA.

The primary objective of CF clinical management is to preserve lung function since a faster decline is closely linked to more advanced CF lung disease and increased mortality.^60^^,^^61^ Lung function is predominantly measured using ppFEV_1_, with patients generally facing an annual decrease of 1–3 percentage points.^62^ In adults, treatment with vanzacaftor 10 mg-tezacaftor 100 mg-deutivacaftor for 4–8 weeks improved ppFEV_1_ by 15.9 percentage points compared with placebo, while vanzacaftor 20 mg-tezacaftor 100 mg-deutivacaftor 150 mg treated for more than 8 weeks improved ppFeV_1_ by 14 percentage points. Vanzacaftor serves as a CFTR corrector, while the deutivacaftor acts as a potentiator. Compared to ivacaftor, deutivacaftor offers a longer half-life, greater exposure, higher plasma concentrations at 24 h, and lower clearance, making it well-suited for once-daily dosing.^63^ Recently, the USFDA approved the triple combination of ALYFTREK (vanzacaftor/tezacaftor/deutivacaftor) for individuals with CF aged 6 and older who have a mutation eligible for Trikafta (elexacaftor/tezacaftor/ivacaftor) or one of 31 previously unapproved rare mutations. Unlike other modulators requiring twice-daily dosing, ALYFTREK only needs to be taken once daily, potentially simplifying the treatment regimen for people with CF.^64^

The second critical outcome assessed was the sweat chloride concentration which serves as an indicator of CFTR function, with studies consistently showing that lower concentrations are associated with better clinical outcomes and reduced mortality in CF patients. In our study, adults with the triple combination of vanzacaftor 20 mg-tezacaftor 100 mg-deutivacaftor 150 mg showed a significant reduction in sweat chloride levels, with mean changes of −49.3 mmol/L at 4–8 weeks, and −50.4 mmol/L beyond 8 weeks compared with placebo, with high certainty of evidence. Similarly, elexacaftor 200 mg-tezacaftor 100 mg-ivacaftor 150 mg reduced sweat chloride by −44.9 mmol/L versus placebo. These regimens achieved SUCRA values of 94% for vanzacaftor 20 mg–tezacaftor 100 mg–deutivacaftor 150 mg at 4–8 weeks, 99% for the same regimen beyond 8 weeks, and 81% for elexacaftor 200 mg–tezacaftor 100 mg–ivacaftor 150 mg beyond 8 weeks, indicating their potential as the most effective options for reducing sweat chloride. Consistent with our findings, a recent observational study conducted on adolescents and adults with CF who have at least one phe508del allele found that elexacaftor–tezacaftor–ivacaftor treatment led to improvements in sweat chloride concentration.^65^

The third important outcome was CFQ-R which is a disease-specific, validated patient-reported outcome measure designed to assess the health-related quality of life in individuals with CF across various health-related domains. It includes 12 domains, both generic and disease-specific, one of which focuses on respiratory symptoms in CF patients. Prior studies have shown that subscales of the CFQ-R are correlated with critical CF clinical variables, such as BMI, lung function, pulmonary exacerbation, and mortality.^66^^,^^67^ Importantly, a change of 4 points in the respiratory domain is considered the minimal clinically important difference (MCID).^68^ We found that the CFQ-R respiratory domain score increased by 39 points in adults treated for 4–8 weeks with vanzacaftor 20 mg–tezacaftor 100 mg–deutivacaftor 150 mg and by 21 points in adults treated for more than 8 weeks with vanzacaftor 20 mg–tezacaftor 100 mg–deutivacaftor 250 mg.

Additionally, the subgroup analysis conducted in the study revealed that both vanzacaftor-tezacaftor -deutivacaftor, and elexacaftor–tezacaftor–ivacaftor were beneficial across all measured outcomes in both homozygous and heterozygous subgroups. These findings highlight the broad applicability of these regimens and suggest that their clinical benefits extend beyond specific genotype categories. Sensitivity analysis excluding the ivacaftor monotherapy trial yielded results that were directionally and quantitatively similar to the primary analysis.

Only three RCTs conducted in children were eligible for inclusion in our review; therefore, an NMA was not performed, as the limited number of trials would not provide reliable estimates. Notably, Mall et al.^57^ reported significant improvements across all outcomes in children treated with elexacaftor 200 mg-tezacaftor 100 mg-ivacaftor 150 mg. Consistent with these findings, single-arm studies, such as those by Hoppe et al.^69^ and Zemanick et al.,^70^ also demonstrated improvements in clinical outcomes with CFTR modulators in pediatric populations. Taken together, these findings indicate that while robust comparative evidence in children is still lacking, the available data consistently suggest clinically meaningful benefits of CFTR modulators in the pediatric population. Furthermore, we observed that the safety outcomes were highly uncertain. Our estimates were unstable, which limits drawing strong conclusions regarding relative safety, primarily because of the low incidence of SAEs in the studies included. Observational studies with larger sample sizes and longer-term follow-up are needed to detect any significant yet rare severe adverse events.

The strength of our NMA lies in its comprehensive literature search, which identified 29 eligible studies involving a total of 6450 participants. The rigor of our analysis is bolstered by the high quality and low risk of bias in the primary trials. Although the RCTs included in our study may offer less precise estimates due to their smaller sample sizes, they nevertheless provide a thorough overview of the current treatment landscape for patients with CF. We analysed each dose separately due to the clear dose–response effects observed with certain medications, which are unlikely to be applicable at lower doses evaluated only in early-phase clinical trials (e.g., tezacaftor 50 mg-ivacaftor 100 mg). Trials involving children and adults were pooled separately, and a subgroup network meta-analysis was conducted specifically for patients with CF who are homozygous or heterozygous for the phe508del mutation, aiming to identify the most effective treatment option.

However, our study also has several limitations. Firstly, due to the small number of trials for several nodes in this NMA, the statistical power for certain comparisons was limited. The majority of direct comparisons were supported by evidence from only a single trial, and approximately three-fourths of all treatment comparisons were based solely on indirect evidence. This limitation should be considered when interpreting our study's findings. Our analysis in children was constrained by the very limited number of studies, and therefore a NMA could not be performed. Future primary studies in children that include direct head-to-head comparisons are crucial for advancing the evidence base. Pooling trials with durations ranging from 4 to 8 weeks may have biased towards medications evaluated in greater than 8-week trials, allowing more time for improvement in CF. Our safety analyses provided results that are either non-estimable or characterized by high uncertainty. Future studies with extended durations and larger sample sizes are needed to offer valuable insights into safety.

In conclusion, our systematic review and NMA offer the first indirect comprehensive comparison of CFTRm therapies for treating patients with CF who have the phe508del mutation. In adults, vanzacaftor-tezacaftor-deutivacaftor and elexacaftor-tezacaftor-ivacaftor treatment combinations were identified as the most effective treatment options. However, healthcare practitioners, patients, and their families should interpret these results with caution, given the limited data and the low quality of the existing evidence. Lacking well-powered head-to-head studies that compare all active treatments, our study provides the most reliable comparative estimates currently available to develop clinical guidelines, guide treatment decisions, and support health technology assessments.

## Contributors

MSVS, SB, and DB conceptualized the study. MSVS, PCV, JKS, and DB formulated the methodology. MSVS, SB, SKB, JKS, and NN extracted the data. MSVS, SKB, SB, NN, and DB analysed the data. MSVS, SB, and PT drafted the initial manuscript. PCV, PT, and DB critically evaluated and revised the manuscript. MSVS and DB have accessed and verified the underlying data. All authors read and approved the final version of the manuscript.

## Data sharing statement

All processed data relevant to this systematic review are included in the article or uploaded as Supplementary information. The datasets analysed in this review are available through a reasonable request to the corresponding author.

## Declaration of interests

All authors declare no competing interests. None of the authors or their immediate family members hold any financial interests, shares, or receive any form of support (including funding, consultancy fees, honoraria, or educational grants) from Vertex Pharmaceuticals.
