# Supplementary material for: Comparative efficacy and safety of CFTR modulators for people with cystic fibrosis with phe508del mutation: a systematic review and bayesian network meta-analysis
Source: eClinicalMedicine. 2025 Nov 25;90:103655. doi: 10.1016/j.eclinm.2025.103655 (PMC12686891; doi:10.1016/j.eclinm.2025.103655)
Supplement: Supplementary Figures and Tables [file mmc1.pdf]

Supplementary Appendix

Comparative efficacy and safety of CFTR modulators for people with cystic fibrosis with phe508del mutation: a systematic review and bayesian network meta-analysis

Mohammed Safeer V S -1, PhD; Simran Behl -1, M Pharmacy; Pankaj C Vaidya -2, DM; Pawan Tiwari -3, DM; Saroj Kundan Bharti -1, PhD; Najiya Nahan -1, M Pharmacy; Jitendra Kumar Sahu -4, DM; Dipika Bansal -1*, DM

1) Department of Pharmacy Practice and Clinical Research, National Institute of Pharmaceutical Education and Research (NIPER), S.A.S. Nagar, India
2) Pediatric Pulmonology Unit, Department of Pediatrics, Postgraduate Institute of Medical Education and Research (PGIMER), Chandigarh, India
3) Department of Pulmonary, Critical Care and Sleep Medicine, All India Institute of Medical Sciences (AIIMS), New Delhi, India
4) Department of Pediatrics, Postgraduate Institute of Medical Education and Research (PGIMER), Chandigarh, India

***Corresponding Author:**

Dr. Dipika Bansal, MD DM;

Professor and Head

Department of Pharmacy Practice and Clinical Research

National Institute of Pharmaceutical Education and Research (NIPER)

S.A.S Nagar, Punjab-160062, India

email: dipikabansal079@gmail.com

Index

Title	Page No.
Literature search strategy	3
Model convergence assessed using the potential scale reduction factor for all the outcomes	5
Evaluation of global inconsistency	6
Node-splitting method for assessing the inconsistency	8
League tables	12
Treatment ranking based on the Surface under the cumulating ranking curve (SUCRA) data for all outcomes	19
Certainty of evidence	27
Summary of results from risk of bias (ROB-2) tool	138
Network diagrams	139
Forest plots	150
SUCRA plots	168

eTable1. Literature Search Strategy

Database	Search Terms	Results
PubMed	1) "Cystic Fibrosis"[Mesh] OR "Fibrocystic" OR "pneumoconiosis" OR "bronchiectasis" OR "pulmonary fibrosis"	1,08,780
	2) "Tezacaftor, ivacaftor drug combination" OR "Kalydeco" OR "VX-770" OR "elexacaftor, ivacaftor, tezacaftor drug combination" OR "CTP-656" OR "VX-445" OR "tezacaftor" OR "VX-661" OR "Deutivacaftor" OR "lumacaftor, ivacaftor drug combination" OR "VRT-826809" OR "VX 809"	1,384
	3) ("Cystic Fibrosis"[Mesh] OR "Fibrocystic" OR "pneumoconiosis" OR "bronchiectasis" OR "pulmonary fibrosis") AND ("tezacaftor, ivacaftor drug combination" OR "Kalydeco" OR "VX-770" OR "elexacaftor, ivacaftor, tezacaftor drug combination" OR "CTP-656" OR "VX-445" OR "tezacaftor" OR "VX-661" OR "Deutivacaftor" OR "lumacaftor, ivacaftor drug combination" OR "VRT-826809" OR "VX 809")	951
Embase	#1 'cystic fibrosis'	118589
	#2 fibrocystic	4,681
	#3 pneumoconiosis	11,584
	#4 bronchiectasis	36,704
	#5 'lung fibrosis'	54,475
	#6 #1 OR #2 OR #3 OR #4 OR #5	2,10,744
	#7 tezacaftor	3550
	#8 ivacaftor	6714
	#9 elexacaftor	2936
	#10 lumacaftor	2839
	#11 deutivacaftor	61
	#12 #7 OR #8 OR #9 OR #10 OR #11	7253
	#13 #6 AND #12	6767
	#14 #13 AND 'randomized controlled trial topic'/de	270

Ovid	1) cystic fibrosis.mp. [mp=title, book title, abstract, original title, name of substance word, subject heading word, floating sub-heading word, keyword heading word, organism supplementary concept word, protocol supplementary concept word, rare disease supplementary concept word, unique identifier, synonyms, population supplementary concept word, anatomy supplementary concept word]<br>2) fibrocystic.mp.<br>3) pneumoconiosis.mp.<br>4) bronchiectasis.mp.<br>5) pulmonary fibrosis.mp.<br>6) 1 or 2 or 3 or 4 or 5<br>7) tezacaftor.mp.<br>8) ivacaftor.mp.<br>9) elexacaftor.mp.<br>10) lumacaftor.mp.<br>11) deutivacaftor.mp.<br>12) 7 or 8 or 9 or 10 or 11<br>13) 6 and 12<br>14) limit 13 to randomized controlled trial	61551<br>4567<br>8694<br>16030<br>40663<br>126155<br>962<br>1853<br>846<br>655<br>9<br>2013<br>1917<br>61
Scopus	1) (“cysticfibrosis" OR "fibrocystic" OR "pneumoconiosis" OR bronchiectasis " or " pulmonary AND fibrosis)<br>2) ALL (“tezacaftor" OR "ivacaftor" OR "lumacaftor" OR "deutivacaftor" OR "e lexacaftor”)<br>3) (ALL (“cysticfibrosis" OR "fibrocystic" OR "pneumoconiosis" OR bronchiect asis "or" pulmonary AND fibrosis)) AND (ALL (“tezacaftor" OR "ivacaftor" OR "lumacaftor" OR "deutivacaftor" OR "elexacaftor”))<br>4) (ALL (“cystic fibrosis" OR "fibrocystic" OR "pneumoconiosis" OR b ronchiectasis "" pulmonary AND fibrosis)) AND (ALL (“tezacaftor" OR "ivaca ftor" OR "lumacaftor" OR "deutivacaftor" OR "elexacaftor”)) AND (LIMIT-TO (DOCTYPE, "ar”)) AND (LIMIT-TO (EXACTKEYWORD, "human”))	72,612<br>8,622<br>3,302<br>1,716
Cochrane	#1 ("pulmonary fibrosis"):ti,ab,kw<br>#2 Cystic fibrosis<br>#3 Fibrocystic<br>#4 Bronchiectasis<br>#5 Tezacaftor<br>#6 elexacaftor<br>#7 ivacaftor<br>#8 lumacaftor<br>#9 deutivacaftor<br>#10 VX-659<br>#11 Vanzacaftor<br>#12 #1 OR #2 OR #3 OR #4<br>#13 #5 OR #6 OR #7 OR #8 OR #9 OR #10 OR #11<br>#14 #12 AND #13	2278<br>7073<br>163<br>1796<br>201<br>106<br>520<br>143<br>10<br>19<br>5<br>10528<br>527<br>475

eTable 2. Model convergence assessed using the potential scale reduction factor for all the outcomes

Outcome	Potential Scale reduction factor (PSRF)
**Percentage predicted forced expiratory volume ($ppFeV_1$)**	
Adults treated for 4 to 8 weeks	1.00
Adults treated for greater than 8 weeks	1.00
Adults treated for greater than 8 weeks (excluding flume et al.)	1.00
Adults in homozygous subpopulation	1.04
Adults in heterozygous subpopulation	1.00
**Sweat chloride**	
Adults treated for 4 to 8 weeks	1.00
Adults treated for greater than 8 weeks	1.00
Adults treated for greater than 8 weeks (excluding flume et al.)	1.00
Adults in homozygous subpopulation	1.00
Adults in heterozygous subpopulation	1.00
**Cystic Fibrosis Questionnaire-Revised (CFQ-R)**	
Adults treated for 4 to 8 weeks	1.00
Adults treated for greater than 8 weeks	1.00
Adults treated for greater than 8 weeks (excluding flume et al.)	1.00
Adults in homozygous subpopulation	1.00
Adults in heterozygous subpopulation	1.00
**Serious Adverse events (SAEs)**	
Adults treated for 4 to 8 weeks	1.04
Adults treated for greater than 8 weeks	1.00
Adults treated for greater than 8 weeks (excluding flume et al.)	1.00

eTable 3. Evaluation of global inconsistency

To assess global inconsistency, we report the mean posterior deviance (D), the number of data points, and the Deviance Information Criterion (DIC) of the NMA model. For models with a good fit, the mean posterior deviance should approximate the number of data points. The DIC, a Bayesian model evaluation criterion, balances model fit with complexity; lower DIC values indicate more preferable models.

Model Assumption	D	No of data points	DIC
**ppFEV$_1$ in adults treated for 4 to 8 weeks**			
Consistency	70.06	72	143.49
Inconsistency	72.27	72	145.36
**ppFEV$_1$ in adults treated for greater than 8 weeks**			
Consistency	25.52	28	58.57
Inconsistency	27.6	28	59.95
**ppFEV$_1$ in adults treated for greater than 8 weeks (excluding flume et al.)**			
Consistency	23.64	26	54.74
Inconsistency	25.63	26	56.05
**ppFEV$_1$ in adults in homozygous subpopulation**			
Consistency	42.32	49	90.52
Inconsistency	44.22	49	91.37
**ppFEV$_1$ in adults in heterozygous subpopulation**			
Consistency	35.14	34	70.96
Inconsistency	35.21	34	70.92
**Sweat chloride in adults treated for 4 to 8 weeks**			
Consistency	65.93	67	134.05
Inconsistency	66.33	67	133.17
**Sweat chloride in adults treated for greater than 8 weeks**			
Consistency	21.56	22	43.42
Inconsistency	21.62	22	43.47
**Sweat chloride in adults treated for greater than 8 weeks (excluding flume et al.)**			
Consistency	19.52	20	39.36
Inconsistency	19.59	20	39.41
**Sweat chloride in adults in homozygous subpopulation**			
Consistency	36.67	39	75.64
Inconsistency	36.47	39	75.38
**Sweat chloride in adults in heterozygous subpopulation**			
Consistency	32.35	33	65.38
Inconsistency	32.31	33	66.9
**CFQ-R in adults treated for 4 to 8 weeks**			
Consistency	55.01	59	114.49

Inconsistency	56.95	59	116.22
**CFQ-R in adults treated for greater than 8 weeks**			
Consistency	22.99	26	47.8
Inconsistency	24.06	26	48.94
**CFQ-R in adults treated for greater than 8 weeks (excluding flume et al.)**			
Consistency	20.99	24	43.81
Inconsistency	22.12	24	45.03
**CFQ-R in adults in homozygous subpopulation**			
Consistency	36.98	43	78.26
Inconsistency	38.27	43	78.89
**CFQ-R in adults in heterozygous subpopulation**			
Consistency	26.7	27	54.21
Inconsistency	26	27	52.13
**Serious adverse events in adults treated for 4 to 8 weeks**			
Consistency	48.36	51	77.68
Inconsistency	48.55	51	70.03
**Serious adverse events in adults treated for greater than 8 weeks**			
Consistency	20.51	24	43.87
Inconsistency	22.23	24	45.75
**Serious adverse events in adults treated for greater than 8 weeks (excluding flume et al.)**			
Consistency	18.39	22	39.72
Inconsistency	20.22	22	41.75

eTable 4 Node-splitting method for assessing the inconsistency.

Results are presented as Mean difference (95%CrI) for efficacy outcomes such as ppFEV1, sweat chloride and CFQ-R. Serious adverse events are presented as Log odds ratio (95%CrI). Significant values (P ≤ 0.05) are in bold, indicating a significant inconsistency between the direct effect and indirect estimates.

Nodes	Direct estimate	Indirect estimate	Network estimate	P-value
**ppFEV$_1$ in adults treated for 4 to 8 weeks**				
Iva_150mg, Elexa_teza_iva_200mg_100mg_150mg,	-5.7 (-12, 0.86)	-8.6 (-14, -3.6)	-7.5 (-11, -3.6)	0.45
Placebo, Elexa_teza_iva_200mg_100mg_150mg,	-14 (-18, -9.5)	-10 (-14, -6.0)	-12 (-15, -8.9)	0.20
Teza_iva_100mg_150mg, Elexa_teza_iva_200mg_100mg_150mg	-7.3 (-11, -3.6)	-9.1 (-14, -4.7)	-8.0 (-11, -5.3)	0.51
Placebo, Iva_150mg	-5.3 (-12, 1.3)	-4.3 (-9.7, 1.2)	-4.4 (-8.2, -0.49)	0.80
Teza_iva_100mg_150mg , Iva_150mg	0.92 (-3.5, 5.4)	-3 (-10, 4.3)	-0.54 (-4.1, 3)	0.33
Luma_iva_400mg_250mg, Luma_iva_200mg_250mg	-1.2 (-9.1, 6.5)	0.38 (-9.8, 11)	-0.72 (-6.5, 5.2)	0.79
Teza_iva_100mg_150mg , Placebo	3.9 (0.47, 7.2)	3.8 (-1.5, 8.4)	3.9 (1.3, 6.3)	0.99
Vanza_teza_deuti_20mg_100mg_150mg, Placebo	7.9 (-1.1, 17)	20 (11, 30)	14 (7, 20)	0.06
VX152_teza_iva_200mg_100mg_150mg, Placebo	10 (2.2, 19)	12 (-0.08, 24)	12 (5.2, 18)	0.80
VX152_teza_iva_300mg_100mg_150mg, Placebo	8.8 (0.43, 17)	13 (4.2, 21)	11 (5.1, 16)	0.49
Vanza_teza_deuti_20mg_100mg_150mg Teza_iva_100mg_150mg	16 (7.1, 25)	3.7 (-5.1, 13)	9.8 (3.5, 16)	0.06
VX152_teza_iva_200mg_100mg_150mg, Teza_iva_100mg_150mg	8.4 (-3.6, 20)	7.6 (0.16, 15)	7.8 (1.3, 14)	0.91
VX152_teza_iva_300mg_100mg_150mg, Teza_iva_100mg_150mg	8.7 (0.97, 16)	5.1 (-3.1, 13)	7 (1.4, 13)	0.52
VX152_teza_iva_300mg_100mg_150mg, VX152_teza_iva_200mg_100mg_150mg	-1.7 (-9.9, 6.5)	0.37 (-14, 15)	-0.80 (-7.7, 6.1)	0.80
**ppFEV$_1$ in adults treated for greater than 8 weeks**				
Placebo, Elexa_teza_iva_200mg_100mg_150mg,	-14 (-17, -12)	-13 (-16, -10)	-14 (-15, -12)	0.47
Teza_iva_100mg_150mg, Elexa_teza_iva_200mg_100mg_150mg	-10 (-13, -7.7)	-11 (-14, -9.0)	-11 (-13, -9.1)	0.47
Teza_iva_100mg_150mg , Placebo	2.9 (1.3, 4.0)	4.1 (0.78, 7.4)	3.1 (1.8, 4.1)	0.47
**ppFEV$_1$ in adults treated for greater than 8 weeks (excluding Flume et al.)**				
Placebo, Elexa_teza_iva_200mg_100mg_150mg,	-14 (-17, -12)	-13 (-16, -10)	-14 (-15, -12)	0.47
Teza_iva_100mg_150mg, Elexa_teza_iva_200mg_100mg_150mg	-10 (-13, -7.8)	-11 (-14, -9.0)	-11 (-13, -9.2)	0.47
Teza_iva_100mg_150mg , Placebo	2.9 (1.3, 4.0)	4.1 (0.79, 7.4)	3.1 (1.8, 4.1)	0.48
**ppFEV1 in homozygous subpopulation**				

Luma_iva_400mg_250mg, Luma_iva_200mg_250mg	-1.1 (-5.8, 3.5)	2.7 (-1.9, 7.5)	1.2 (-1.9, 4.3)	0.23
Luma_iva_600mg_250mg, Luma_iva_200mg_250mg	1.8 (-2.8, 6.5)	3 (-1.7, 7.6)	1.6 (-1.6. 4.7)	0.73
Placebo, Luma_iva_200mg_250mg	-0.23 (-4.9, 4.4)	-2.8 (-7, 1.4)	-1.5 (-4.7, 1.5)	0.41
Placebo , Luma_iva_400mg_250mg	-2.8 (-4.3, 1.3)	-0.4 (-6.8, 5.7)	-2.8 (-4.1, -1.4)	0.46
Placebo , Luma_iva_600mg_250mg	-3.3 (-4.9, -1.8)	-0.49 (-6.8, 5.8)	-3.2 (-4.5, -1.8)	0.37
**ppFEV1 in heterozygous subpopulation**				
Iva_150mg, Elexa_teza_iva_200mg_100mg_150mg	-5.7 (-13, 1.9)	-7.3 (-14, -0.97)	-6.7 (-11, -2.2)	0.70
Placebo, Elexa_teza_iva_200mg_100mg_150mg	-14 (-17, -11)	-7.5 (-12, -2.8)	-12 (-16, -8.8)	**0.02**
Teza_iva_100mg_150mg, Elexa_teza_iva_200mg_100mg_150mg	-2.0 (-7.3, 3.3)	-8.7 (-13, -4.8)	-6.3 (-10, -2.4)	**0.04**
Placebo, Iva_150mg	-5.3 (-14, 3.2)	-5.8 (-13, 1.7)	-5.5 (-10, -0.99)	0.91
Teza_iva_100mg_150mg, Iva_150mg	0.91 (-5.1, 7)	-1.3 (-12, 9.1)	0.36 (-3.8, 4.5)	0.66
Teza_iva_100mg_150mg, Placebo	4.0 (0.33, 7.5)	11 (5.3, 16)	5.9 (2.1, 9.4)	**0.04**
**Sweat chloride in adults treated for 4 to 8 weeks**				
Iva_150mg, Elexa_teza_iva_200mg_100mg_150mg,	20 (-1.3, 41)	42 (19, 66)	30 (13, 47)	0.14
Placebo, Elexa_teza_iva_200mg_100mg_150mg,	39 (22, 56)	37 (17, 56)	38 (26, 50)	0.85
Teza_iva_100mg_150mg, Elexa_teza_iva_200mg_100mg_150mg	37 (23, 50)	29 (11, 48)	34 (24, 45)	0.47
Teza_iva_100mg_150mg , Iva_150mg	-5.9 (-27, 15)	16 (-7, 40)	4.0 (-13, 21)	0.13
Luma_iva_400mg_250mg, Luma_iva_200mg_250mg	-4.6 (-26, 17)	-0.08 (-31, 30)	-3.2 (-19, 13)	0.79
Teza_iva_100mg_150mg, Placebo	-12 (-29, 5.0)	1 (-13, 16)	-4.2 (-15, 7.0)	0.22
Vanza_teza_deuti_20mg_100mg_150mg, Placebo	-52 (-78, -26)	-46 (-74, -18)	-49 (-67, -31)	0.75
VX152_teza_iva_200mg_100mg_150mg, Placebo	5.9 (-16, 27)	-34 (-60, -8.2)	-12 (-30, 6.6)	**0.02**
VX152_teza_iva_300mg_100mg_150mg, Placebo	-7.9 (-29, 14)	-33 (-56, -9.6)	-22 (-40, -3.7)	0.11
Vanza_teza_deuti_20mg_100mg_150mg, Teza_iva_100mg_150mg	-43 (-68, -18)	-48 (-77, -19)	-45 (-63, -27)	0.76
VX152_teza_iva_200mg_100mg_150mg, Teza_iva_100mg_150mg	-25 (-50, 0.0)	6.2 (-16, 29)	-7.6 (-24, 10)	0.06
VX152_teza_iva_300mg_100mg_150mg, Teza_iva_100mg_150mg	-24 (-49, 0.6)	-11 (-37, 14)	-18 (-35, -0.39)	0.43
VX152_teza_iva_300mg_100mg_150mg, VX152_teza_iva_200mg_100mg_150mg	-14 (-35, 7.4)	1.0 (-30, 33)	-10 (-30, 10)	0.41
**Sweat chloride in adults treated for greater than 8 weeks**				
Placebo, Elexa_teza_iva_200mg_100mg_150mg	42 (32, 52)	49 (37, 62)	45 (37, 53)	0.26

Teza_iva_100mg_150mg, Elexa_teza_iva_200mg_100mg_150mg	43 (32, 53)	35 (23, 47)	39 (32, 47)	0.26
Teza_iva_100mg_150mg , Placebo	-6.6 (-13, -0.4)	0.98 (-14, 16)	-5.5 (-11, 0.17)	0.26
**Sweat chloride in adults treated for greater than 8 weeks (excluding Flume et al.)**				
Placebo, Elexa_teza_iva_200mg_100mg_150mg	42 (31, 52)	49 (37, 61)	45 (37, 53)	0.26
Teza_iva_100mg_150mg, Elexa_teza_iva_200mg_100mg_150mg	43 (32, 53)	35 (23, 47)	39 (32, 47)	0.27
Teza_iva_100mg_150mg , Placebo	-6.6 (-13, -0.29)	1.0 (-14, 16)	-5.5 (-11, 0.21)	0.26
**Sweat chloride in adults in heterozygous subpopulation**				
Iva_150mg, Elexa_teza_iva_200mg_100mg_150mg	20 (5.9, 34)	35 (17, 51)	26 (13, 38)	0.14
Placebo, Elexa_teza_iva_200mg_100mg_150mg	40 (33, 47)	28 (16, 43)	38 (30, 46)	0.11
Teza_iva_100mg_150mg, Elexa_teza_iva_200mg_100mg_150mg	25 (6.5, 43)	27 (11, 40)	26 (16, 35)	0.82
Teza_iva_100mg_150mg, Iva_150mg	-5.8 (-20, 8.2)	9.1 (-8.9, 26)	0.01 (-12, 12)	0.15
Teza_iva_100mg_150mg, Placebo	-5.7 (-18, 1.9)	-18 (-30, -6.8)	-12 (-22, -2.8)	0.10
**CFQ-R in adults treated for 4 to 8 weeks**				
Iva_150mg, Elexa_teza_iva_200mg_100mg_150mg	-8.9 (-19, 0.78)	-17 (-28, -7.2)	-13 (-21, -5.2)	0.18
Placebo, Elexa_teza_iva_200mg_100mg_150mg	-21 (-27, -14)	-15 (-24, -7.6)	-19 (-24, -14)	0.25
Teza_iva_100mg_150mg, Elexa_teza_iva_200mg_100mg_150mg	-14 (-20, -6.9)	-14 (-22, -4.6)	-14 (-19, -8.8)	0.92
Teza_iva_100mg_150mg , Iva_150mg	2.8 (-6.6, 12)	-5.8 (-17, 5.4)	-0.53 (-8.3, 6.9)	0.18
Teza_iva_100mg_150mg, Placebo	0.9 (-5.6, 7.5)	9.2 (3, 16)	5.3 (-0.09, 11)	0.06
Vanza_teza_deuti_20mg_100mg_150mg, Placebo	27 (8.2, 45)	30 (12, 48)	28 (16, 41)	0.79
VX152_teza_iva_200mg_100mg_150mg, Placebo	29 (12, 47)	9 (-10, 28)	20 (7.1, 32)	0.11
VX152_teza_iva_300mg_100mg_150mg, Placebo	26 (8.7, 43)	15 (0.78, 30)	18 (7.7, 29)	0.33
Vanza_teza_deuti_20mg_100mg_150mg, Teza_iva_100mg_150mg	24 (7.4, 41)	21 (1.5, 40)	23 (10, 35)	0.79
VX152_teza_iva_200mg_100mg_150mg, Teza_iva_100mg_150mg	4.8 (-14, 24)	21 (5.5, 37)	14 (2.1, 26)	0.18
VX152_teza_iva_300mg_100mg_150mg, Teza_iva_100mg_150mg	11 (-2.6, 25)	16 (-0.6, 33)	13 (2.9, 24)	0.65
VX152_teza_iva_300mg_100mg_150mg, VX152_teza_iva_200mg_100mg_150mg	-3.2 (-20, 13)	6.5 (-17, 29)	-1.1 (-14, 12)	0.48
**CFQ-R in adults treated for greater than 8 weeks**				
Placebo, Elexa_teza_iva_200mg_100mg_150mg	-20 (-26, -15)	-19 (-27, -12)	-20 (-24, -16)	0.86
Teza_iva_100mg_150mg, Elexa_teza_iva_200mg_100mg_150mg	-16 (-22, -9.6)	-17 (-24, -10)	-16 (-21, -12)	0.87
Teza_iva_100mg_150mg, Placebo	3.6 (-0.41, 7.0)	4.3 (-4.3, 13)	3.8 (0.5, 6.7)	0.88

**CFQ-R in adults treated for greater than 8 weeks (excluding Flume et al.)**				
Placebo, Elexa_teza_iva_200mg_100mg_150mg	-20 (-26, -15)	-20 (-27, -12)	-20 (-24, -16)	0.86
Teza_iva_100mg_150mg, Elexa_teza_iva_200mg_100mg_150mg	-16 (-22, -9.5)	-17 (-24, -10)	-16 (-20, -12)	0.87
Teza_iva_100mg_150mg, Placebo	3.6 (-0.34, 7.0)	4.3 (-4.3, 13)	3.8 (0.5, 6.6)	0.87
**CFQ-R in adults in heterozygous subpopulation**				
Iva_150mg, Elexa_teza_iva_200mg_100mg_150mg	-8.9 (-24, 6.7)	-16 (-35, 3.6)	-12 (-22, -1.5)	0.39
Placebo, Elexa_teza_iva_200mg_100mg_150mg	-21 (-24, -17)	-9.7 (-18, -1.2)	-19 (-25, -12)	**0.03**
Teza_iva_100mg_150mg, Elexa_teza_iva_200mg_100mg_150mg	-8.5 (-24, 7.2)	-14 (-27, 0.0)	-12 (-20, -2.7)	0.42
Teza_iva_100mg_150mg, Iva_150mg	2.8 (-13, 18)	-4.3 (-24, 15)	0.4 (-9.9, 10)	0.40
Teza_iva_100mg_150mg, Placebo	2.0 (-4.9, 8.9)	13 (5.8, 20)	7.3 (-1.7, 17)	**0.04**
**Serious adverse events in adults treated for 4 to 8 weeks**				
Placebo, Elexa_teza_iva_200mg_100mg_150mg	19 (1.5, 63)	2.1 (-3, 7.7)	3.4 (-0.3, 8.5)	0.10
Teza_iva_100mg_150mg, Elexa_teza_iva_200mg_100mg_150mg	0.64 (-4, 5.3)	26 (0.4, 86)	1.6 (-2.1, 6.0)	0.07
Placebo, Iva_150mg	-0.05 (-4.2, 4.2)	-25 (-95, 0.8)	-0.95 (-5.1, 2.5)	0.08
Teza_iva_100mg_150mg , Placebo	-1.3 (-4.2, 1.1)	-15 (-47, 0.65)	-1.8 (-4.7, 0.4)	0.11
**Serious adverse events in adults treated for greater than 8 weeks**				
Placebo, Elexa_teza_iva_200mg_100mg_150mg,	0.5 (-0.35, 1.3)	1.3 (-0.2, 2.9)	0.66 (-0.008, 1.4)	0.32
Teza_iva_100mg_150mg, Elexa_teza_iva_200mg_100mg_150mg	1.2 (-0.03, 2.5)	0.39 (-0.76, 1.5)	0.72 (-0.08, 1.6)	0.32
Teza_iva_100mg_150mg, Placebo	-0.12 (-0.91, 0.7)	0.67 (-0.84, 2.2)	0.05 (-0.63, 0.75)	0.33
**Serious adverse events in adults treated for greater than 8 weeks (excluding Flume et al.)**				
Placebo, Elexa_teza_iva_200mg_100mg_150mg,	0.5 (-0.36, 1.4)	1.3 (-0.2, 2.9)	0.66 (-0.016, 1.4)	0.33
Teza_iva_100mg_150mg, Elexa_teza_iva_200mg_100mg_150mg	1.2 (-0.03, 2.6)	0.37 (-0.8, 1.6)	0.72 (-0.07, 1.6)	0.33
Teza_iva_100mg_150mg, Placebo	-0.12 (-0.9, 0.7)	0.68 (-0.81, 2.3)	0.06 (-0.63, 0.75)	0.33

Abbreviation: Elexa: elexacaftor; Teza: Tezacaftor; Iva: Ivacaftor; Luma: Lumacaftor; Deuti: Deutivacaftor; Vanza: Vanzacaftor; ppFEV$_1$: percentage predicted forced expiratory volume; CFQ-R: Cystic fibrosis questionnaire revised

eTable 5 League tables

League tables for adults treated for over 8 weeks and after excluding Flume et al. trial are provided below. However, the league tables for adults treated for 4 to 8 weeks are not included due to the large number of interventions, making it impractical to present them as league tables. These tables can be requested directly from the authors.

eTable 5a League table of sweat chloride in adults treated for greater than 8 weeks. Results are presented as mean difference (95%CrI)

**Vanza_teza_deuti_20 mg_100mg_250mg**					
**-5.5 (-10.6 to -0.5)**	**Elexa_teza_iva_200m g_100mg_150mg**				
**-36.9 (-53.0 to -20.6)**	**-31.4 (-46.7 to -16.0)**	**Teza_iva_50mg_150 mg**			
**-44.9 (-54.3 to -35.7)**	**-39.4 (-47.3 to -31.7)**	**-8.0 (-22.5 to 6.3)**	**Teza_iva_100mg_150 mg**		
**-47.6 (-61.4 to -34.1)**	**-42.1 (-54.9 to -29.4)**	**-10.7 (-27.5 to 5.9)**	**-2.7 (-14.2 to 9.0)**	**Iva_150mg**	
**-50.4 (-59.7 to -41.1)**	**-44.9 (-52.6 to -37.0)**	**-13.4 (-26.7 to -0.3)**	**-5.5 (-11.0 to 0.2)**	**-2.8 (-12.9 to 7.4)**	**Placebo**

eTable 5b League table of CFQ-R in adults treated for greater than 8 weeks. Results are presented as mean difference (95%CrI)

**Vanza_teza_deuti_20 mg_100mg_250mg**							
**1.1 (-1.9 to 4.1)**	**Elexa_teza_iva_200 mg_100mg_150mg**						
**10.9 (-4.5 to 26.1)**	**9.7 (-5.4 to 24.7)**	**Teza_iva_50mg_150mg**					
**17.3 (12.3 to 22.6)**	**16.2 (12.1 to 20.5)**	**6.5 (-8.4 to 21.4)**	**Teza_iva_100mg_15 0mg**				
**18.3 (12.2 to 24.4)**	**17.3 (11.9 to 22.5)**	**7.5 (-7.5 to 22.5)**	**1.0 (-3.8 to 5.5)**	**Luma_iva_400mg_2 50mg**			
**18.6 (12.4 to 24.6)**	**17.5 (12.1 to 22.7)**	**7.8 (-7.3 to 22.8)**	**1.3 (-3.6 to 5.7)**	**0.3 (-2.8 to 3.3)**	**Luma_iva_600mg_2 50mg**		
**19.8 (12.0 to 27.4)**	**18.7 (11.5 to 25.7)**	**9.0 (-6.7 to 24.6)**	**2.5 (-4.2 to 9.0)**	**1.4 (-5.4 to 8.2)**	**1.2 (-5.7 to 8.0)**	**Iva_150mg**	
**21.1 (16.0 to 26.0)**	**20.0 (15.9 to 23.9)**	**10.3 (-4.3 to 24.8)**	**3.8 (0.6 to 6.6)**	**2.8 (-0.8 to 6.3)**	**2.5 (-1.0 to 6.1)**	**1.3(-4.6 to 7.2)**	**Placebo**

eTable 5c League table of serious adverse events in adults treated for greater than 8 weeks. Results are presented as log odds ratio (95%CrI)

**Teza_iva_50mg_150mg**							
**-0.6 (-4.3 to 2.5)**	**Vanza_teza_deuti_20mg_100mg_250mg**						
**-0.9 (-4.5 to 2.1)**	**0.4 (-3.0 to 4.3)**	**Luma_iva_400mg_250mg**					
**-0.8 (-4.4 to 2.2)**	**-0.5 (-1.8 to 0.8)**	**0.1 (-0.8 to 1.0)**	**Elexa_teza_iva_200mg_100mg_150mg**				
**-0.9 (-4.7 to 2.2)**	**-0.4 (-1.3 to 0.4)**	**-0.1 (-1.5 to 1.3)**	**-0.1 (-1.6 to 1.3)**	**Iva_150mg**			
**-1.1 (-4.7 to 1.9)**	**-0.5 (-2.3 to 1.1)**	**-0.3 (-0.7 to 0.2)**	**-0.3 (-1.3 to 0.6)**	**-0.2 (-1.5 to 1.2)**	**Luma_iva_600mg_250mg**		
**-1.5 (-5.0 to 1.5)**	**-0.7 (-2.1 to 0.5)**	**-0.6 (-1.2 to -0.1)**	**-0.7 (-1.4 to 0.0)**	**-0.5 (-1.8 to 0.8)**	**-0.3 (-0.9 to 0.2)**	**Placebo**	
**-1.5 (-5.2 to -1.5)**	**-1.1 (-2.3 to 0.0)**	**-0.7 (-1.6 to 0.2)**	**-0.7 (-1.6 to 0.1)**	**-0.6 (-2.0 to 0.9)**	**-0.4 (-1.3 to 0.5)**	**-0.1 (-0.8 to 0.6)**	**Teza_iva_100mg_150mg**

eTable 5d League table of ppFeV$_1$ in adults treated for greater than 8 weeks (excluding Flume et al.). Results are presented as mean difference (95%CrI)

**Vanza_teza_deuti_20mg_100mg_250mg**						
**0.2 (-0.5 to 0.9)**	**Elexa_teza_iva_200mg_100mg_150mg**					
**10.9 (8.5 to 13.0)**	**10.7 (8.5 to 12.7)**	**Luma_iva_600mg_250mg**				
**11.0 (9.2 to 12.9)**	**10.8 (9.1 to 12.5)**	**0.1 (-1.5 to 2.0)**	**Teza_iva_100mg_150mg**			
**11.1 (8.7 to 13.2)**	**10.9 (8.6 to 12.9)**	**0.2 (-0.9 to 1.4)**	**0.1 (-1.8 to 1.7)**	**Luma_iva_400mg_250mg**		
**13.1 (6.8 to 19.4)**	**12.9 (6.6 to 19.2)**	**2.3 (-3.9 to 8.4)**	**2.1 (-4.1 to 8.2)**	**2.0 (-4.2 to 8.2)**	**Teza_iva_50mg_150mg**	
**14.1 (12.2 to 15.8)**	**13.9 (12.1 to 15.5)**	**3.2 (1.9 to 4.5)**	**3.1 (1.8 to 4.1)**	**3.0 (1.6 to 4.3)**	**0.9 (-5.1 to 7.0)**	**Placebo**

eTable 5e League table of sweat chloride in adults treated for greater than 8 weeks (excluding Flume et al.). Results are presented as mean difference (95%CrI)

**Vanza_teza_deuti_20mg_100mg_250mg**				
**-5.5 (-10.5 to -0.4)**	**Elexa_teza_iva_200mg_100mg_150mg**			
**-36.9 (-53.1 to -20.9)**	**-31.4 (-46.8 to -16.2)**	**Teza_iva_50mg_150mg**		
**-44.9 (-54.4 to -35.7)**	**-39.4 (-47.4 to -31.7)**	**-8.0 (-22.3 to 6.2)**	**Teza_iva_100mg_150mg**	
**-50.3 (-59.7 to -41.0)**	**-44.8 (-52.7 to -37.1)**	**-13.5 (-26.6 to -0.2)**	**-5.5 (-10.9 to 0.2)**	**Placebo**

eTable 5f League table of CFQ-R in adults treated for greater than 8 weeks (excluding Flume et al.). Results are presented as mean difference (95%CrI)

**Vanza_teza_deuti_20 mg_100mg_250mg**						
**1.1 (-1.9 to 4.1)**	**Elexa_teza_iva_200 mg_100mg_150mg**					
**10.9 (-4.5 to 26.2)**	**9.8 (-5.3 to 24.9)**	**Teza_iva_50mg_150mg**				
**17.3 (12.3 to 22.5)**	**16.2 (12.2 to 20.5)**	**6.5 (-8.5 to 21.3)**	**Teza_iva_100mg_15 0mg**			
**18.3 (12.1 to 24.3)**	**17.2 (11.8 to 22.5)**	**7.4 (-7.6 to 22.5)**	**1.0 (-3.9 to 5.5)**	**Luma_iva_400mg_2 50mg**		
**18.6 (12.4 to 24.6)**	**17.5 (12.1 to 22.7)**	**7.7 (-7.3 to 22.6)**	**1.3 (-3.6 to 5.8)**	**0.3 (-2.8 to 3.3)**	**Luma_iva_600mg_2 50mg**	
**21.1 (16.0 to 26.0)**	**20.0 (15.9 to 23.9)**	**10.2 (-4.5 to 24.8)**	**3.8 (0.5 to 6.6)**	**2.8 (-0.8 to 6.3)**	**2.5 (-1.0 to 6.0)**	**Placebo**

eTable 5g League table of serious adverse events in adults treated for greater than 8 weeks (excluding Flume et al.). Results are presented as log odds ratio (95%CrI)

**Vanza_teza_deuti_20mg_100mg_250mg**						
**0.6 (-2.5 to 4.4)**	**Teza_iva_50mg_150mg**					
**-0.2 (-1.4 to 0.9)**	**-0.9 (-4.6 to 2.2)**	**Luma_iva_400mg_250mg**				
**-0.2 (-0.9 to 0.6)**	**-0.8 (-4.5 to 2.3)**	**0.0 (-0.8 to 1.0)**	**Elexa_teza_iva_200mg_100mg_150mg**			
**-0.5 (-1.7 to 0.6)**	**-1.1 (-4.8 to 1.9)**	**-0.3 (-0.7 to 0.2)**	**-0.3 (-1.2 to 0.5)**	**Luma_iva_600mg_250mg**		
**-0.8 (-1.9 to 0.1)**	**-1.5 (-5.1 to 1.5)**	**-0.6 (-1.2 to -0.1)**	**-0.7 (-1.4 to -0.0)**	**-0.4 (-0.9 to 0.2)**	**Placebo**	
**-0.9 (-2.1 to 0.2)**	**-1.5 (-5.2 to 1.6)**	**-0.7 (-1.5 to 0.2)**	**-0.7 (-1.6 to 0.1)**	**-0.4 (-1.3 to 0.5)**	**-0.1 (-0.7 to 0.6)**	**Teza_iva_100mg_150mg**

eTable 6 Treatment ranking based on Surface under the cumulating ranking curve (SUCRA) data for all outcomes

Treatments	SUCRA(%)
**SUCRA rankings for ppFEV$_1$ in adults treated for 4 to 8 weeks**	
Vanza_teza_deuti_10mg_100mg_150mg	92.09
Vanza_teza_deuti_20mg_100mg_150mg	88.04
Elexa_teza_iva_200mg_100mg_150mg	84.6
VX152_teza_iva_200mg_100mg_150mg	82.52
VX152_teza_iva_300mg_100mg_150mg	79.83
Ola_teza_iva_600mg_50mg_300mg	77.64
Elexa_teza_iva_50mg_100mg_150mg	77.22
Vanza_teza_iva_5mg_100mg_150mg	73.51
VX152_teza_iva_100mg_100mg_150mg	65.19
Elexa_teza_iva_100mg_100mg_150mg	63.65
Vanza_teza_deuti_5mg_100mg_150mg	58.07
Iva_150mg	51.17
Luma_iva_600mg_250mg	50.83
Teza_iva_100mg_150mg	48
GLPG2737_75mg	46.89
Luma_iva_200mg_150mg	45.69
Luma_200mg	42.13
Luma_100mg	41.69
GLPG2222_300mg	40.3
Luma_50mg	38.32
Luma_25mg	37.04
GLPG2222_400mg	35.64
Luma_iva_200mg_250mg	35.13
Luma_iva_400mg_250mg	30.57
Teza_iva_50mg_150mg	30.53
GLPG2222_100mg	29.2
GLPG2222_50mg	29.03
GLPG2222_200mg	28.55
GLPG2222_150mg	27.04
Teza_iva_50mg_300mg	23.8
Teza_iva_100mg_50mg	23.49

Placebo	22.59
**SUCRA rankings for ppFEV$_1$ in adults treated for greater than 8 weeks**	
Vanza_teza_deuti_20mg_100mg_250mg	97.25
Elexa_teza_iva_200mg_100mg_150mg	88.47
Luma_iva_600mg_250mg	54.67
Teza_iva_100mg_150mg	50.88
Luma_iva_400mg_250mg	47.07
Iva_150mg	28.56
Teza_iva_50mg_150mg	26.39
Placebo	6.72
**SUCRA rankings for ppFEV$_1$ in adults treated for greater than 8 weeks (excluding Flume et al.)**	
Vanza_teza_deuti_20mg_100mg_250mg	96.69
Elexa_teza_iva_200mg_100mg_150mg	86.62
Luma_iva_600mg_250mg	49.84
Teza_iva_100mg_150mg	45.63
Luma_iva_400mg_250mg	41.91
Teza_iva_50mg_150mg	22.89
Placebo	6.4
**SUCRA rankings for ppFEV$_1$ in adults in homozygous subpopulation**	
Vanza_teza_deuti_20mg_100mg_150mg	97
Elexa_teza_iva_200mg_100mg_150mg	91.1
GLPG2737_75mg	69.53
Teza_iva_100mg_150mg	66.93
Luma_iva_600mg_250mg	66.2
Luma_iva_200mg_150mg	64.12
Luma_iva_400mg_250mg	59.51
GLPG2222_400mg	47.31
Iva_150mg	46.67
Luma_iva_200mg_250mg	44.98
Luma_200mg	43.95
Luma_100mg	40.77
Teza_iva_50mg_150mg	37.98
Luma_50mg	37.54
GLPG2222_50mg	35.8
GLPG2222_100mg	35.11
GLPG2222_200mg	33.71
Luma_25mg	30.75
Placebo	26.01
Teza_iva_100mg_50mg	25.03

SUCRA rankings for ppFEV$_1$ in adults in heterozygous subpopulation	
Elexa_teza_iva_200mg_100mg_150mg	84.84
Vanza_teza_deuti_20mg_100mg_250mg	84.35
Vanza_teza_deuti_10mg_100mg_150mg	81.35
Elexa_teza_iva_50mg_100mg_150mg	73.24
Vanza_teza_iva_5mg_100mg_150mg	68.77
Vanza_teza_deuti_20mg_100mg_150mg	58.37
Elexa_teza_iva_100mg_100mg_150mg	53.8
Teza_iva_100mg_150mg	46.81
Iva_150mg	44.23
Vanza_teza_deuti_5mg_100mg_150mg	29.76
GLPG2222_300mg	28.62
Luma_iva_400mg_250mg	17.71
GLPG2222_150mg	16.08
Placebo	12.05
**SUCRA rankings for sweat chloride in adults treated for 4 to 8 weeks**	
Vanza_teza_deuti_20mg_100mg_150mg	93.97
Vanza_teza_iva_5mg_100mg_150mg	91.98
Vanza_teza_deuti_10mg_100mg_150mg	91.22
Vanza_teza_deuti_5mg_100mg_150mg	88.64
Elexa_teza_iva_200mg_100mg_150mg	85.41
Elexa_teza_iva_50mg_100mg_150mg	83.14
Ola_teza_iva_600mg_50mg_300mg	80.53
Elexa_teza_iva_100mg_100mg_150mg	77.56
VX152_teza_iva_300mg_100mg_150mg	65.86
GLPG2222_200mg	54.1
GLPG2737_75mg	53.38
GLPG2222_300mg	51.41
VX152_teza_iva_200mg_100mg_150mg	46.56
Luma_iva_400mg_250mg	45.8
Luma_iva_600mg_250mg	44.93
VX152_teza_iva_100mg_100mg_150mg	43.74
GLPG2222_150mg	42.91
Iva_150mg	38.66
Luma_iva_200mg_250mg	38.2
GLPG2222_400mg	37.06
Teza_iva_50mg_150mg	35.79
Luma_200mg	35.77
GLPG2222_100mg	32.46

Teza_iva_100mg_50mg	31.92
Luma_100mg	31.27
Luma_iva_200mg_150mg	28.8
Luma_50mg	28.57
GLPG2222_50mg	28.54
Teza_iva_100mg_150mg	28.43
Teza_iva_50mg_300mg	28.41
Luma_25mg	17.55
Placebo	17.41
**SUCRA rankings for sweat chloride in adults treated for greater than 8 weeks**	
Vanza_teza_deuti_20mg_100mg_150mg	99.59
Elexa_teza_iva_200mg_100mg_150mg	80.36
Teza_iva_50mg_150mg	55.35
Teza_iva_100mg_150mg	36
Iva_150mg	22.42
Placebo	6.27
**SUCRA rankings for sweat chloride in adults treated for greater than 8 weeks (excluding Flume et al.)**	
Vanza_teza_deuti_20mg_100mg_150mg	99.49
Elexa_teza_iva_200mg_100mg_150mg	75.47
Teza_iva_50mg_150mg	46.39
Teza_iva_100mg_150mg	27.37
Placebo	1.27
**SUCRA rankings for sweat chloride in adults in homozygous subpopulation**	
Vanza_teza_deuti_20mg_100mg_150mg	97.48
Elexa_teza_iva_200mg_100mg_150mg	97.18
GLPG2222_200mg	77.02
Luma_iva_400mg_250mg	76
Luma_iva_600mg_250mg	75.36
GLPG2737_75mg	74.99
Luma_iva_200mg_250mg	61.65
Teza_iva_100mg_150mg	56.08
Teza_iva_50mg_150mg	54.32
GLPG2222_400mg	47.14
Luma_200mg	43.7
Teza_iva_100mg_50mg	37.52
GLPG2222_100mg	36.62
Luma_iva_200mg_150mg	35.17
Luma_100mg	33.63
GLPG2222_50mg	27.94

Luma_50mg	26.9
Iva_150mg	24.55
Placebo	10.16
Luma_25mg	6.5
**SUCRA rankings for sweat chloride in adults in heterozygous subpopulation**	
Vanza_teza_deuti_20mg_100mg_150mg	88.55
Vanza_teza_iva_5mg_100mg_150mg	82.78
Vanza_teza_deuti_10mg_100mg_150mg	81.63
Vanza_teza_deuti_20mg_100mg_250mg	80.14
Vanza_teza_deuti_5mg_100mg_150mg	75.44
Elexa_teza_iva_200mg_100mg_150mg	62.38
Elexa_teza_iva_50mg_100mg_150mg	60.16
Elexa_teza_iva_100mg_100mg_150mg	50.36
GLPG2222_300mg	28.23
Teza_iva_100mg_150mg	23.22
Iva_150mg	23.04
Luma_iva_400mg_250mg	21.73
GLPG2222_150mg	20.19
Placebo	2.19
**SUCRA rankings for CFQ-R in adults treated for 4 to 8 weeks**	
Vanza_teza_deuti_20mg_100mg_150mg	96.71
Elexa_teza_iva_200mg_100mg_150mg	85.57
VX152_teza_iva_200mg_100mg_150mg	84.56
Ola_teza_iva_600mg_50mg_300mg	81.83
VX152_teza_iva_300mg_100mg_150mg	82.85
Vanza_teza_deuti_10mg_100mg_150mg	81.07
Elexa_teza_iva_50mg_100mg_150mg	75.95
Vanza_teza_deuti_5mg_100mg_150mg	71.46
Luma_iva_200mg_250mg	70.32
Elexa_teza_iva_100mg_100mg_150mg	60.91
Luma_iva_400mg_250mg	57.26
GLPG2222_200mg	53.5
VX152_teza_iva_100mg_100mg_150mg	53.09
Iva_150mg	47.45
Teza_iva_100mg_150mg	45.41
Luma_iva_600mg_250mg	42.88
GLPG2222_50mg	40.48
GLPG2222_100mg	38.54
GLPG2222_400mg	35.65

GLPG2222_300mg	31.15
GLPG2222_150mg	29.53
GLPG2737_75mg	28.84
Teza_iva_50mg_300mg	27.05
Placebo	26.16
Luma_200mg	23.4
Luma_100mg	14.97
Luma_25mg	7.56
Luma_50mg	5.87
**SUCRA rankings for CFQ-R in adults treated for greater than 8 weeks**	
Vanza_teza_deuti_20mg_100mg_250mg	96.33
Elexa_teza_iva_200mg_100mg_150mg	86.77
Teza_iva_50mg_150mg	63.82
Teza_iva_100mg_150mg	48.17
Luma_iva_400mg_250mg	38.14
Luma_iva_600mg_250mg	34.58
Iva_150mg	24.43
Placebo	7.76
**SUCRA rankings for CFQ-R in adults treated for greater than 8 weeks (excluding Flume et al.)**	
Vanza_teza_deuti_20mg_100mg_250mg	95.71
Elexa_teza_iva_200mg_100mg_150mg	84.53
Teza_iva_50mg_150mg	59.76
Teza_iva_100mg_150mg	43.02
Luma_iva_400mg_250mg	33.38
Luma_iva_600mg_250mg	29.87
Placebo	3.76
**SUCRA rankings for CFQ-R in adults in homozygous subpopulation**	
Vanza_teza_deuti_20mg_100mg_150mg	98.63
Elexa_teza_iva_200mg_100mg_150mg	94.01
Teza_iva_50mg_150mg	74.69
GLPG2222_200mg	73.81
Luma_iva_200mg_250mg	73.03
Teza_iva_100mg_150mg	57.55
GLPG2222_50mg	54.74
Luma_iva_400mg_250mg	53.86
GLPG2222_100mg	52.17
Luma_iva_600mg_250mg	49.12
GLPG2222_400mg	48.35
Iva_150mg	42.95

GLPG2737_75mg	37.23
Placebo	32.62
Luma_200mg	29.01
Luma_100mg	16.8
Luma_25mg	6.74
Luma_50mg	4.67
**SUCRA rankings for CFQ-R in adults in heterozygous subpopulation**	
Vanza_teza_deuti_20mg_100mg_150mg	90.2
Vanza_teza_deuti_20mg_100mg_250mg	82.57
Elexa_teza_iva_200mg_100mg_150mg	77.96
Vanza_teza_deuti_10mg_100mg_150mg	69.64
Elexa_teza_iva_50mg_100mg_150mg	65.8
Vanza_teza_deuti_5mg_100mg_150mg	58.53
Elexa_teza_iva_100mg_100mg_150mg	47.64
Teza_iva_100mg_150mg	38.71
Iva_150mg	37.17
Luma_iva_400mg_250mg	36.34
GLPG2222_300mg	17.77
GLPG2222_150mg	16.24
Placebo	11.41
**SUCRA rankings for Serious adverse events in adults treated for 4 to 8 weeks**	
GLPG2222_50mg	83.26
VX152_teza_iva_200mg_100mg_150mg	82.69
Luma_iva_200mg_250mg	84.05
GLPG2222_200mg	82.75
Luma_100mg	81.86
VX152_teza_iva_100mg_100mg_150mg	82.64
GLPG2222_400mg	82.97
Ola_teza_iva_200mg_50mg_150mg	60.6
Ola_teza_iva_200mg_100mg_150mg	64.07
Vanza_teza_deuti_20mg_100mg_150mg	58.02
Elexa_teza_iva_200mg_100mg_150mg	60.85
Vanza_teza_deuti_10mg_100mg_150mg	58.3
Teza_iva_100mg_150mg	53.18
VX152_teza_iva_300mg_100mg_150mg	47.3
GLPG2222_100mg	44.25
Teza_iva_50mg_150mg	42.3
Elexa_teza_iva_50mg_100mg_150mg	41.36
Elexa_teza_iva_100mg_100mg_150mg	40.32

Luma_iva_400mg_250mg	41.13
Luma_50mg	39.11
Placebo	37.48
Luma_iva_600mg_250mg	34.41
Iva_150mg	30.61
Luma_200mg	27.98
Luma_25mg	22.82
Ola_teza_iva_600mg_50mg_300mg	7.57
Vanza_teza_deuti_5mg_100mg_150mg	4.13
Teza_iva_50mg_300mg	3.91
**SUCRA rankings for Serious adverse events in adults treated for greater than 8 weeks**	
Teza_iva_50mg_150mg	74.33
Vanza_teza_deuti_20mg_100mg_250mg	74.1
Luma_iva_400mg_250mg	63.35
Elexa_teza_iva_200mg_100mg_150mg	63
Iva_150mg	53.17
Luma_iva_600mg_250mg	40.6
Placebo	15.74
Teza_iva_100mg_150mg	15.68
**SUCRA rankings for Serious adverse events in adults treated for greater than 8 weeks (excluding Flume et al.)**	
Teza_iva_50mg_150mg	74.33
Vanza_teza_deuti_20mg_100mg_250mg	74.1
Luma_iva_400mg_250mg	63.35
Elexa_teza_iva_200mg_100mg_150mg	63
Luma_iva_600mg_250mg	40.6
Placebo	15.74
Teza_iva_100mg_150mg	15.68

**Larger SUCRAs denote effective/safe interventions*

Certainty of evidence

We assessed the confidence in the network meta-analysis (NMA) of the primary outcome using the CINeMA (Confidence in Network Meta-Analysis) framework, implemented via the CINeMA web application. This online tool utilizes the original data to perform a network meta-analysis, evaluate the results for each comparison, and generate a contribution matrix. The CINeMA framework evaluates confidence across six key domains that influence the reliability of NMA results: within-study bias, reporting bias, indirectness, imprecision, heterogeneity, and incoherence**.**

Domain 1 Within study bias

We assessed within-study bias using the overall risk of bias ratings from Cochrane's RoB 2.0 tool (eFigure 1). For each comparison, within-study bias was classified into three categories: 'no concerns,' 'some concerns,' or 'major concerns,' based on the average overall risk of bias derived from the contribution matrix.

Domain 2 Reporting bias

To evaluate the potential publication bias for the primary outcome, we examined the funnel plot. However, since the number of studies for each comparison was fewer than 10, publication bias could not be formally assessed, as per the guidance in the Cochrane Handbook for Systematic Reviews of Interventions. Nonetheless, our search strategy was thorough, encompassing both published and unpublished studies, as well as trial registries such as ClinicalTrials.gov.

Domain 3 Indirectness

This domain assesses whether the study populations, interventions, outcomes, and settings are representative of those relevant to the research questions. In the studies included in this review, the strict inclusion criteria ensured that these characteristics were directly aligned with the research question, resulting in no identified indirectness.

Domain 4 Imprecision

This domain involves establishing thresholds for clinically significant differences between interventions. We defined a mean difference of one as clinically important.

Domain 5 Heterogeneity

Heterogeneity was assessed by examining the relationship between both the 95% confidence intervals and the 95% prediction intervals with the clinically meaningful threshold established in the imprecision domain.

Domain 6 Incoherence

According to the CINeMA documentation, incoherence was evaluated using a design-by-treatment test for comparisons that relied solely on direct or indirect evidence. For comparisons involving both direct and indirect evidence, the SIDE approach was applied.

eTable 7 Certainty ratings of ppFEV$_1$ for adults treated for 4 to 8 weeks using CINEMA framework

Comparison	Number of studies	Within-study bias	Reporting bias	Indirectness	Imprecision	Heterogeneity	Incoherence	Confidence rating	Reason(s) for downgrading
**Mixed evidence**									
Elexa_teza_iva_100mg_100mg_150mg:Elexa_teza_iva_200mg_100mg_150mg	1	No concerns	Low risk	No concerns	No concerns	Major concerns	Major concerns	Very low	["Heterogeneity"," Incoherence"]
Elexa_teza_iva_100mg_100mg_150mg:Elexa_teza_iva_50mg_100mg_150mg	1	No concerns	Low risk	No concerns	No concerns	Major concerns	No concerns	Low	["Heterogeneity"]
Elexa_teza_iva_100mg_100mg_150mg:Placebo	1	No concerns	Low risk	No concerns	No concerns	Major concerns	No concerns	Low	["Heterogeneity"]
Elexa_teza_iva_200mg_100mg_150mg:Elexa_teza_iva_50mg_100mg_150mg	1	No concerns	Low risk	No concerns	No concerns	Major concerns	Major concerns	Very low	["Heterogeneity"," Incoherence"]
Elexa_teza_iva_200mg_100mg_150mg:Iva_150mg	1	No concerns	Low risk	No concerns	No concerns	No concerns	No concerns	High	NA
Elexa_teza_iva_200mg_100mg_150mg:Placebo	2	No concerns	Low risk	No concerns	No concerns	No concerns	No concerns	High	NA
Elexa_teza_iva_200mg_100mg_150mg:Teza_iva_100mg_150mg	3	No concerns	Low risk	No concerns	No concerns	No concerns	No concerns	High	NA
Elexa_teza_iva_50mg_100mg_150mg:Placebo	1	No concerns	Low risk	No concerns	No concerns	No concerns	No concerns	High	NA
GLPG2222_100mg:GLPG2222_200mg	1	No concerns	Low risk	No concerns	Major concerns	No concerns	No concerns	Low	["Imprecision"]
GLPG2222_100mg:GLPG2222_400mg	1	No concerns	Low risk	No concerns	Major concerns	No concerns	No concerns	Low	["Imprecision"]
GLPG2222_100mg:GLPG2222_50mg	1	No concerns	Low risk	No concerns	Major concerns	No concerns	No concerns	Low	["Imprecision"]
GLPG2222_100mg:Placebo	1	No concerns	Low risk	No concerns	Major concerns	No concerns	No concerns	Low	["Imprecision"]

GLPG2222_150mg:GLPG2222_300mg	1	No concerns	Low risk	No concerns	Major concerns	No concerns	No concerns	Low	["Imprecision"]
GLPG2222_150mg:Placebo	1	No concerns	Low risk	No concerns	Major concerns	No concerns	No concerns	Low	["Imprecision"]
GLPG2222_200mg:GLPG2222_400mg	1	No concerns	Low risk	No concerns	Major concerns	No concerns	No concerns	Low	["Imprecision"]
GLPG2222_200mg:GLPG2222_50mg	1	No concerns	Low risk	No concerns	Major concerns	No concerns	No concerns	Low	["Imprecision"]
GLPG2222_200mg:Placebo	1	No concerns	Low risk	No concerns	Major concerns	No concerns	No concerns	Low	["Imprecision"]
GLPG2222_300mg:Placebo	1	No concerns	Low risk	No concerns	Major concerns	No concerns	No concerns	Low	["Imprecision"]
GLPG2222_400mg:GLPG2222_50mg	1	No concerns	Low risk	No concerns	Major concerns	No concerns	No concerns	Low	["Imprecision"]
GLPG2222_400mg:Placebo	1	No concerns	Low risk	No concerns	Major concerns	No concerns	No concerns	Low	["Imprecision"]
GLPG2222_50mg:Placebo	1	No concerns	Low risk	No concerns	Major concerns	No concerns	No concerns	Low	["Imprecision"]
GLPG2737_75mg:Placebo	1	No concerns	Low risk	No concerns	No concerns	Major concerns	No concerns	Low	["Heterogeneity"]
Iva_150mg:Placebo	1	No concerns	Low risk	No concerns	No concerns	Major concerns	No concerns	Low	["Heterogeneity"]
Iva_150mg:Teza_iva_100mg_150mg	2	Some concerns	Low risk	No concerns	Major concerns	No concerns	Major concerns	Very low	["Within-study bias", "Imprecision", "Incoherence"]
Ola_teza_iva_600mg_50mg_300mg:Placebo	1	No concerns	Low risk	No concerns	No concerns	No concerns	No concerns	High	NA
Ola_teza_iva_600mg_50mg_300mg:Teza_iva_50mg_300mg	1	No concerns	Low risk	No concerns	No concerns	No concerns	No concerns	High	NA
Placebo:Teza_iva_100mg_150mg	5	No concerns	Low risk	No concerns	No concerns	Major concerns	No concerns	Low	["Heterogeneity"]
Placebo:Teza_iva_100mg_50mg	1	Some concerns	Low risk	No concerns	Major concerns	No concerns	No concerns	Low	["Within-study bias", "Imprecision"]

Placebo:Teza_iva_50mg_150mg	1	Some concerns	Low risk	No concerns	Major concerns	No concerns	No concerns	Low	["Within-study bias", "Imprecision"]
Placebo:VX152_teza_iva_100mg_100mg_150mg	1	No concerns	Low risk	No concerns	No concerns	Major concerns	No concerns	Low	["Heterogeneity"]
Placebo:VX152_teza_iva_200mg_100mg_150mg	1	No concerns	Low risk	No concerns	No concerns	No concerns	No concerns	High	NA
Placebo:VX152_teza_iva_300mg_100mg_150mg	1	No concerns	Low risk	No concerns	No concerns	No concerns	No concerns	High	NA
luma_100mg:Placebo	1	Some concerns	Low risk	No concerns	Major concerns	No concerns	No concerns	Low	["Within-study bias", "Imprecision"]
luma_200mg:Placebo	1	Some concerns	Low risk	No concerns	Major concerns	No concerns	No concerns	Low	["Within-study bias", "Imprecision"]
luma_25mg:Placebo	1	Some concerns	Low risk	No concerns	Major concerns	No concerns	No concerns	Low	["Within-study bias", "Imprecision"]
luma_50mg:Placebo	1	Some concerns	Low risk	No concerns	Major concerns	No concerns	No concerns	Low	["Within-study bias", "Imprecision"]
luma_iva_200mg_150mg:Placebo	1	No concerns	Low risk	No concerns	Major concerns	No concerns	No concerns	Low	["Imprecision"]
luma_iva_200mg_250mg:Placebo	2	No concerns	Low risk	No concerns	Major concerns	No concerns	No concerns	Low	["Imprecision"]
luma_iva_400mg_250mg:Placebo	2	Some concerns	Low risk	No concerns	Major concerns	No concerns	No concerns	Low	["Within-study bias", "Imprecision"]
luma_iva_600mg_250mg:Placebo	1	No concerns	Low risk	No concerns	No concerns	Major concerns	No concerns	Low	["Heterogeneity"]
Placebo:vanza_teza_deuti_10mg_100mg_150mg	1	No concerns	Low risk	No concerns	No concerns	No concerns	No concerns	High	NA
Placebo:vanza_teza_deuti_20mg_100mg_150mg	1	No concerns	Low risk	No concerns	No concerns	No concerns	No concerns	High	NA
Placebo:vanza_teza_deuti_5mg_100mg_150mg	1	No concerns	Low risk	No concerns	Major concerns	No concerns	Major concerns	Very low	["Imprecision", "Incoherence"]

Placebo:vanza_teza_iva_5mg_100mg_150mg	1	No concerns	Low risk	No concerns	Major concerns	No concerns	No concerns	Low	["Imprecision"]
Teza_iva_100mg_150mg:VX152_teza_iva_200mg_100mg_150mg	1	No concerns	Low risk	No concerns	No concerns	Major concerns	No concerns	Low	["Heterogeneity"]
Teza_iva_100mg_150mg:VX152_teza_iva_300mg_100mg_150mg	1	No concerns	Low risk	No concerns	No concerns	Major concerns	No concerns	Low	["Heterogeneity"]
Teza_iva_100mg_150mg:vanza_teza_deuti_20mg_100mg_150mg	1	No concerns	Low risk	No concerns	No concerns	No concerns	Major concerns	Low	["Incoherence"]
Teza_iva_100mg_50mg:Teza_iva_50mg_150mg	1	Some concerns	Low risk	No concerns	Major concerns	No concerns	No concerns	Low	["Within-study bias", "Imprecision"]
VX152_teza_iva_100mg_100mg_150mg:VX152_teza_iva_200mg_100mg_150mg	1	No concerns	Low risk	No concerns	Major concerns	No concerns	No concerns	Low	["Imprecision"]
VX152_teza_iva_100mg_100mg_150mg:VX152_teza_iva_300mg_100mg_150mg	1	No concerns	Low risk	No concerns	Major concerns	No concerns	No concerns	Low	["Imprecision"]
VX152_teza_iva_200mg_100mg_150mg:VX152_teza_iva_300mg_100mg_150mg	1	No concerns	Low risk	No concerns	Major concerns	No concerns	No concerns	Low	["Imprecision"]
luma_100mg:luma_200mg	1	Some concerns	Low risk	No concerns	Major concerns	No concerns	No concerns	Low	["Within-study bias", "Imprecision"]
luma_100mg:luma_25mg	1	Some concerns	Low risk	No concerns	Major concerns	No concerns	No concerns	Low	["Within-study bias", "Imprecision"]
luma_100mg:luma_50mg	1	Some concerns	Low risk	No concerns	Major concerns	No concerns	No concerns	Low	["Within-study bias", "Imprecision"]
luma_200mg:luma_25mg	1	Some concerns	Low risk	No concerns	Major concerns	No concerns	No concerns	Low	["Within-study bias", "Imprecision"]
luma_200mg:luma_50mg	1	Some concerns	Low risk	No concerns	Major concerns	No concerns	No concerns	Low	["Within-study bias", "Imprecision"]
luma_25mg:luma_50mg	1	Some concerns	Low risk	No concerns	Major concerns	No concerns	No concerns	Low	["Within-study bias", "Imprecision"]

luma_iva_200mg_150mg:luma_iva_200mg_250mg	1	No concerns	Low risk	No concerns	Major concerns	No concerns	No concerns	Low	["Imprecision"]
luma_iva_200mg_250mg:luma_iva_400mg_250mg	1	No concerns	Low risk	No concerns	Major concerns	No concerns	No concerns	Low	["Imprecision"]
luma_iva_200mg_250mg:luma_iva_600mg_250mg	1	No concerns	Low risk	No concerns	Major concerns	No concerns	No concerns	Low	["Imprecision"]
luma_iva_400mg_250mg:luma_iva_600mg_250mg	1	No concerns	Low risk	No concerns	Major concerns	No concerns	No concerns	Low	["Imprecision"]
vanza_teza_deuti_10mg_100mg_150mg:vanza_teza_deuti_20mg_100mg_150mg	1	No concerns	Low risk	No concerns	Major concerns	No concerns	Major concerns	Very low	["Imprecision", "Incoherence"]
vanza_teza_deuti_10mg_100mg_150mg:vanza_teza_deuti_5mg_100mg_150mg	1	No concerns	Low risk	No concerns	No concerns	Major concerns	No concerns	Low	["Heterogeneity"]
vanza_teza_deuti_20mg_100mg_150mg:vanza_teza_deuti_5mg_100mg_150mg	1	No concerns	Low risk	No concerns	No concerns	Major concerns	Major concerns	Very low	["Heterogeneity", "Incoherence"]
Indirect evidence									
Elexa_teza_iva_100mg_100mg_150mg:GLPG2222_100mg	0	No concerns	Low risk	No concerns	No concerns	Major concerns	No concerns	Low	["Heterogeneity"]
Elexa_teza_iva_100mg_100mg_150mg:GLPG2222_150mg	0	No concerns	Low risk	No concerns	No concerns	Major concerns	No concerns	Low	["Heterogeneity"]
Elexa_teza_iva_100mg_100mg_150mg:GLPG2222_200mg	0	No concerns	Low risk	No concerns	No concerns	Major concerns	No concerns	Low	["Heterogeneity"]
Elexa_teza_iva_100mg_100mg_150mg:GLPG2222_300mg	0	No concerns	Low risk	No concerns	No concerns	Major concerns	No concerns	Low	["Heterogeneity"]
Elexa_teza_iva_100mg_100mg_150mg:GLPG2222_400mg	0	No concerns	Low risk	No concerns	No concerns	Major concerns	No concerns	Low	["Heterogeneity"]
Elexa_teza_iva_100mg_100mg_150mg:GLPG2222_50mg	0	No concerns	Low risk	No concerns	No concerns	Major concerns	No concerns	Low	["Heterogeneity"]
Elexa_teza_iva_100mg_100mg_150mg:GLPG2737_75mg	0	No concerns	Low risk	No concerns	Major concerns	No concerns	No concerns	Low	["Imprecision"]
Elexa_teza_iva_100mg_100mg_150mg:Iva_150mg	0	No concerns	Low risk	No concerns	No concerns	Major concerns	No concerns	Low	["Heterogeneity"]
Elexa_teza_iva_100mg_100mg_150mg:Ola_teza_iva_600mg_50mg_300mg	0	No concerns	Low risk	No concerns	Major concerns	No concerns	No concerns	Low	["Imprecision"]

Elexa_teza_iva_100mg_100mg_150mg:Teza_iva_100mg_150mg	0	No concerns	Low risk	No concerns	No concerns	Major concerns	No concerns	Low	["Heterogeneity"]
Elexa_teza_iva_100mg_100mg_150mg:Teza_iva_100mg_50mg	0	No concerns	Low risk	No concerns	No concerns	Major concerns	No concerns	Low	["Heterogeneity"]
Elexa_teza_iva_100mg_100mg_150mg:Teza_iva_50mg_150mg	0	No concerns	Low risk	No concerns	No concerns	Major concerns	No concerns	Low	["Heterogeneity"]
Elexa_teza_iva_100mg_100mg_150mg:Teza_iva_50mg_300mg	0	No concerns	Low risk	No concerns	No concerns	Major concerns	No concerns	Low	["Heterogeneity"]
Elexa_teza_iva_100mg_100mg_150mg:VX152_teza_iva_100mg_100mg_150mg	0	No concerns	Low risk	No concerns	Major concerns	No concerns	No concerns	Low	["Imprecision"]
Elexa_teza_iva_100mg_100mg_150mg:VX152_teza_iva_200mg_100mg_150mg	0	No concerns	Low risk	No concerns	No concerns	Major concerns	No concerns	Low	["Heterogeneity"]
Elexa_teza_iva_100mg_100mg_150mg:VX152_teza_iva_300mg_100mg_150mg	0	No concerns	Low risk	No concerns	No concerns	Major concerns	No concerns	Low	["Heterogeneity"]
Elexa_teza_iva_100mg_100mg_150mg:luma_100mg	0	No concerns	Low risk	No concerns	Major concerns	No concerns	No concerns	Low	["Imprecision"]
Elexa_teza_iva_100mg_100mg_150mg:luma_200mg	0	No concerns	Low risk	No concerns	Major concerns	No concerns	No concerns	Low	["Imprecision"]
Elexa_teza_iva_100mg_100mg_150mg:luma_25mg	0	No concerns	Low risk	No concerns	Major concerns	No concerns	No concerns	Low	["Imprecision"]
Elexa_teza_iva_100mg_100mg_150mg:luma_50mg	0	No concerns	Low risk	No concerns	Major concerns	No concerns	No concerns	Low	["Imprecision"]
Elexa_teza_iva_100mg_100mg_150mg:luma_iva_200mg_150mg	0	No concerns	Low risk	No concerns	Major concerns	No concerns	No concerns	Low	["Imprecision"]
Elexa_teza_iva_100mg_100mg_150mg:luma_iva_200mg_250mg	0	No concerns	Low risk	No concerns	No concerns	Major concerns	No concerns	Low	["Heterogeneity"]
Elexa_teza_iva_100mg_100mg_150mg:luma_iva_400mg_250mg	0	No concerns	Low risk	No concerns	No concerns	Major concerns	No concerns	Low	["Heterogeneity"]
Elexa_teza_iva_100mg_100mg_150mg:luma_iva_600mg_250mg	0	No concerns	Low risk	No concerns	Major concerns	No concerns	No concerns	Low	["Imprecision"]
Elexa_teza_iva_100mg_100mg_150mg:vanza_teza_deuti_10mg_100mg_150mg	0	No concerns	Low risk	No concerns	No concerns	Major concerns	No concerns	Low	["Heterogeneity"]
Elexa_teza_iva_100mg_100mg_150mg:vanza_teza_deuti_20mg_100mg_150mg	0	No concerns	Low risk	No concerns	No concerns	Major concerns	No concerns	Low	["Heterogeneity"]

Elexa_teza_iva_100mg_100mg_150mg:vanza_teza_deuti_5mg_100mg_150mg	0	No concerns	Low risk	No concerns	Major concerns	No concerns	No concerns	Low	["Imprecision"]
Elexa_teza_iva_100mg_100mg_150mg:vanza_teza_iva_5mg_100mg_150mg	0	No concerns	Low risk	No concerns	Major concerns	No concerns	No concerns	Low	["Imprecision"]
Elexa_teza_iva_200mg_100mg_150mg:GLPG2222_100mg	0	No concerns	Low risk	No concerns	No concerns	No concerns	No concerns	High	NA
Elexa_teza_iva_200mg_100mg_150mg:GLPG2222_150mg	0	No concerns	Low risk	No concerns	No concerns	No concerns	No concerns	High	NA
Elexa_teza_iva_200mg_100mg_150mg:GLPG2222_200mg	0	No concerns	Low risk	No concerns	No concerns	No concerns	No concerns	High	NA
Elexa_teza_iva_200mg_100mg_150mg:GLPG2222_300mg	0	No concerns	Low risk	No concerns	No concerns	No concerns	No concerns	High	NA
Elexa_teza_iva_200mg_100mg_150mg:GLPG2222_400mg	0	No concerns	Low risk	No concerns	No concerns	No concerns	No concerns	High	NA
Elexa_teza_iva_200mg_100mg_150mg:GLPG2222_50mg	0	No concerns	Low risk	No concerns	No concerns	No concerns	No concerns	High	NA
Elexa_teza_iva_200mg_100mg_150mg:GLPG2737_75mg	0	No concerns	Low risk	No concerns	No concerns	Major concerns	No concerns	Low	["Heterogeneity"]
Elexa_teza_iva_200mg_100mg_150mg:Ola_teza_iva_600mg_50mg_300mg	0	No concerns	Low risk	No concerns	Major concerns	No concerns	No concerns	Low	["Imprecision"]
Elexa_teza_iva_200mg_100mg_150mg:Teza_iva_100mg_50mg	0	No concerns	Low risk	No concerns	No concerns	No concerns	No concerns	High	NA
Elexa_teza_iva_200mg_100mg_150mg:Teza_iva_50mg_150mg	0	No concerns	Low risk	No concerns	No concerns	No concerns	No concerns	High	NA
Elexa_teza_iva_200mg_100mg_150mg:Teza_iva_50mg_300mg	0	No concerns	Low risk	No concerns	No concerns	No concerns	No concerns	High	NA
Elexa_teza_iva_200mg_100mg_150mg:VX152_teza_iva_100mg_100mg_150mg	0	No concerns	Low risk	No concerns	Major concerns	No concerns	No concerns	Low	["Imprecision"]
Elexa_teza_iva_200mg_100mg_150mg:VX152_teza_iva_200mg_100mg_150mg	0	No concerns	Low risk	No concerns	Major concerns	No concerns	No concerns	Low	["Imprecision"]
Elexa_teza_iva_200mg_100mg_150mg:VX152_teza_iva_300mg_100mg_150mg	0	No concerns	Low risk	No concerns	Major concerns	No concerns	No concerns	Low	["Imprecision"]
Elexa_teza_iva_200mg_100mg_150mg:luma_100mg	0	No concerns	Low risk	No concerns	Major concerns	No concerns	No concerns	Low	["Imprecision"]

Elexa_teza_iva_200mg_100mg_150mg:luma_200mg	0	No concerns	Low risk	No concerns	Major concerns	No concerns	No concerns	Low	["Imprecision"]
Elexa_teza_iva_200mg_100mg_150mg:luma_25mg	0	No concerns	Low risk	No concerns	Major concerns	No concerns	No concerns	Low	["Imprecision"]
Elexa_teza_iva_200mg_100mg_150mg:luma_50mg	0	Some concerns	Low risk	No concerns	Major concerns	No concerns	No concerns	Low	["Within-study bias", "Imprecision"]
Elexa_teza_iva_200mg_100mg_150mg:luma_iva_200mg_150mg	0	No concerns	Low risk	No concerns	No concerns	Major concerns	No concerns	Low	["Heterogeneity"]
Elexa_teza_iva_200mg_100mg_150mg:luma_iva_200mg_250mg	0	No concerns	Low risk	No concerns	No concerns	No concerns	No concerns	High	NA
Elexa_teza_iva_200mg_100mg_150mg:luma_iva_400mg_250mg	0	No concerns	Low risk	No concerns	No concerns	No concerns	No concerns	High	NA
Elexa_teza_iva_200mg_100mg_150mg:luma_iva_600mg_250mg	0	No concerns	Low risk	No concerns	No concerns	Major concerns	No concerns	Low	["Heterogeneity"]
Elexa_teza_iva_200mg_100mg_150mg:vanza_teza_deuti_10mg_100mg_150mg	0	No concerns	Low risk	No concerns	Major concerns	No concerns	No concerns	Low	["Imprecision"]
Elexa_teza_iva_200mg_100mg_150mg:vanza_teza_deuti_20mg_100mg_150mg	0	No concerns	Low risk	No concerns	Major concerns	No concerns	No concerns	Low	["Imprecision"]
Elexa_teza_iva_200mg_100mg_150mg:vanza_teza_deuti_5mg_100mg_150mg	0	No concerns	Low risk	No concerns	Major concerns	No concerns	No concerns	Low	["Imprecision"]
Elexa_teza_iva_200mg_100mg_150mg:vanza_teza_iva_5mg_100mg_150mg	0	No concerns	Low risk	No concerns	Major concerns	No concerns	No concerns	Low	["Imprecision"]
Elexa_teza_iva_50mg_100mg_150mg:GLPG2222_100mg	0	No concerns	Low risk	No concerns	No concerns	No concerns	No concerns	High	NA
Elexa_teza_iva_50mg_100mg_150mg:GLPG2222_150mg	0	No concerns	Low risk	No concerns	No concerns	No concerns	No concerns	High	NA
Elexa_teza_iva_50mg_100mg_150mg:GLPG2222_200mg	0	No concerns	Low risk	No concerns	No concerns	No concerns	No concerns	High	NA
Elexa_teza_iva_50mg_100mg_150mg:GLPG2222_300mg	0	No concerns	Low risk	No concerns	No concerns	Major concerns	No concerns	Low	["Heterogeneity"]
Elexa_teza_iva_50mg_100mg_150mg:GLPG2222_400mg	0	No concerns	Low risk	No concerns	No concerns	Major concerns	No concerns	Low	["Heterogeneity"]

Elexa_teza_iva_50mg_100mg_150mg:GLPG2222_50mg	0	No concerns	Low risk	No concerns	No concerns	Major concerns	No concerns	Low	["Heterogeneity"]
Elexa_teza_iva_50mg_100mg_150mg:GLPG2737_75mg	0	No concerns	Low risk	No concerns	No concerns	Major concerns	No concerns	Low	["Heterogeneity"]
Elexa_teza_iva_50mg_100mg_150mg:Iva_150mg	0	No concerns	Low risk	No concerns	No concerns	Major concerns	No concerns	Low	["Heterogeneity"]
Elexa_teza_iva_50mg_100mg_150mg:Ola_teza_iva_600mg_50mg_300mg	0	No concerns	Low risk	No concerns	Major concerns	No concerns	No concerns	Low	["Imprecision"]
Elexa_teza_iva_50mg_100mg_150mg:Teza_iva_100mg_150mg	0	No concerns	Low risk	No concerns	No concerns	Major concerns	No concerns	Low	["Heterogeneity"]
Elexa_teza_iva_50mg_100mg_150mg:Teza_iva_100mg_50mg	0	No concerns	Low risk	No concerns	No concerns	No concerns	No concerns	High	NA
Elexa_teza_iva_50mg_100mg_150mg:Teza_iva_50mg_150mg	0	No concerns	Low risk	No concerns	No concerns	Major concerns	No concerns	Low	["Heterogeneity"]
Elexa_teza_iva_50mg_100mg_150mg:Teza_iva_50mg_300mg	0	No concerns	Low risk	No concerns	No concerns	Major concerns	No concerns	Low	["Heterogeneity"]
Elexa_teza_iva_50mg_100mg_150mg:VX152_teza_iva_100mg_100mg_150mg	0	No concerns	Low risk	No concerns	Major concerns	No concerns	No concerns	Low	["Imprecision"]
Elexa_teza_iva_50mg_100mg_150mg:VX152_teza_iva_200mg_100mg_150mg	0	No concerns	Low risk	No concerns	Major concerns	No concerns	No concerns	Low	["Imprecision"]
Elexa_teza_iva_50mg_100mg_150mg:VX152_teza_iva_300mg_100mg_150mg	0	No concerns	Low risk	No concerns	Major concerns	No concerns	No concerns	Low	["Imprecision"]
Elexa_teza_iva_50mg_100mg_150mg:luma_100mg	0	No concerns	Low risk	No concerns	Major concerns	No concerns	No concerns	Low	["Imprecision"]
Elexa_teza_iva_50mg_100mg_150mg:luma_200mg	0	No concerns	Low risk	No concerns	Major concerns	No concerns	No concerns	Low	["Imprecision"]
Elexa_teza_iva_50mg_100mg_150mg:luma_25mg	0	No concerns	Low risk	No concerns	Major concerns	No concerns	No concerns	Low	["Imprecision"]
Elexa_teza_iva_50mg_100mg_150mg:luma_50mg	0	No concerns	Low risk	No concerns	Major concerns	No concerns	No concerns	Low	["Imprecision"]
Elexa_teza_iva_50mg_100mg_150mg:luma_iva_200mg_150mg	0	No concerns	Low risk	No concerns	No concerns	Major concerns	No concerns	Low	["Heterogeneity"]
Elexa_teza_iva_50mg_100mg_150mg:luma_iva_200mg_250mg	0	No concerns	Low risk	No concerns	No concerns	Major concerns	No concerns	Low	["Heterogeneity"]

Elexa_teza_iva_50mg_100mg_150mg:luma_iva_400mg_250mg	0	No concerns	Low risk	No concerns	No concerns	No concerns	No concerns	High	NA
Elexa_teza_iva_50mg_100mg_150mg:luma_iva_600mg_250mg	0	No concerns	Low risk	No concerns	No concerns	Major concerns	No concerns	Low	["Heterogeneity"]
Elexa_teza_iva_50mg_100mg_150mg:vanza_teza_deuti_10mg_100mg_150mg	0	No concerns	Low risk	No concerns	No concerns	Major concerns	No concerns	Low	["Heterogeneity"]
Elexa_teza_iva_50mg_100mg_150mg:vanza_teza_deuti_20mg_100mg_150mg	0	No concerns	Low risk	No concerns	Major concerns	No concerns	No concerns	Low	["Imprecision"]
Elexa_teza_iva_50mg_100mg_150mg:vanza_teza_deuti_5mg_100mg_150mg	0	No concerns	Low risk	No concerns	Major concerns	No concerns	No concerns	Low	["Imprecision"]
Elexa_teza_iva_50mg_100mg_150mg:vanza_teza_iva_5mg_100mg_150mg	0	No concerns	Low risk	No concerns	Major concerns	No concerns	No concerns	Low	["Imprecision"]
GLPG2222_100mg:GLPG2222_150mg	0	No concerns	Low risk	No concerns	Major concerns	No concerns	No concerns	Low	["Imprecision"]
GLPG2222_100mg:GLPG2222_300mg	0	No concerns	Low risk	No concerns	Major concerns	No concerns	No concerns	Low	["Imprecision"]
GLPG2222_100mg:GLPG2737_75mg	0	No concerns	Low risk	No concerns	Major concerns	No concerns	No concerns	Low	["Imprecision"]
GLPG2222_100mg:Iva_150mg	0	No concerns	Low risk	No concerns	No concerns	Major concerns	No concerns	Low	["Heterogeneity"]
GLPG2222_100mg:Ola_teza_iva_600mg_50mg_300mg	0	No concerns	Low risk	No concerns	No concerns	Major concerns	No concerns	Low	["Heterogeneity"]
GLPG2222_100mg:Teza_iva_100mg_150mg	0	No concerns	Low risk	No concerns	No concerns	Major concerns	No concerns	Low	["Heterogeneity"]
GLPG2222_100mg:Teza_iva_100mg_50mg	0	No concerns	Low risk	No concerns	Major concerns	No concerns	No concerns	Low	["Imprecision"]
GLPG2222_100mg:Teza_iva_50mg_150mg	0	No concerns	Low risk	No concerns	Major concerns	No concerns	No concerns	Low	["Imprecision"]
GLPG2222_100mg:Teza_iva_50mg_300mg	0	No concerns	Low risk	No concerns	Major concerns	No concerns	No concerns	Low	["Imprecision"]
GLPG2222_100mg:VX152_teza_iva_100mg_100mg_150mg	0	No concerns	Low risk	No concerns	No concerns	Major concerns	No concerns	Low	["Heterogeneity"]
GLPG2222_100mg:VX152_teza_iva_200mg_100mg_150mg	0	No concerns	Low risk	No concerns	No concerns	No concerns	No concerns	High	NA

GLPG2222_100mg:VX152_teza_iva_300mg_100mg_150mg	0	No concerns	Low risk	No concerns	No concerns	No concerns	No concerns	High	NA
GLPG2222_100mg:luma_100mg	0	No concerns	Low risk	No concerns	Major concerns	No concerns	No concerns	Low	["Imprecision"]
GLPG2222_100mg:luma_200mg	0	Some concerns	Low risk	No concerns	Major concerns	No concerns	No concerns	Low	["Within-study bias", Imprecision"]
GLPG2222_100mg:luma_25mg	0	Some concerns	Low risk	No concerns	Major concerns	No concerns	No concerns	Low	["Within-study bias", Imprecision"]
GLPG2222_100mg:luma_50mg	0	Some concerns	Low risk	No concerns	Major concerns	No concerns	No concerns	Low	["Within-study bias", Imprecision"]
GLPG2222_100mg:luma_iva_200mg_150mg	0	No concerns	Low risk	No concerns	Major concerns	No concerns	No concerns	Low	["Imprecision"]
GLPG2222_100mg:luma_iva_200mg_250mg	0	No concerns	Low risk	No concerns	Major concerns	No concerns	No concerns	Low	["Imprecision"]
GLPG2222_100mg:luma_iva_400mg_250mg	0	No concerns	Low risk	No concerns	Major concerns	No concerns	No concerns	Low	["Imprecision"]
GLPG2222_100mg:luma_iva_600mg_250mg	0	No concerns	Low risk	No concerns	Major concerns	No concerns	No concerns	Low	["Imprecision"]
GLPG2222_100mg:vanza_teza_deuti_10mg_100mg_150mg	0	No concerns	Low risk	No concerns	No concerns	No concerns	No concerns	High	NA
GLPG2222_100mg:vanza_teza_deuti_20mg_100mg_150mg	0	No concerns	Low risk	No concerns	No concerns	No concerns	No concerns	High	NA
GLPG2222_100mg:vanza_teza_deuti_5mg_100mg_150mg	0	No concerns	Low risk	No concerns	Major concerns	No concerns	No concerns	Low	["Imprecision"]
GLPG2222_100mg:vanza_teza_iva_5mg_100mg_150mg	0	No concerns	Low risk	No concerns	Major concerns	No concerns	No concerns	Low	["Imprecision"]
GLPG2222_150mg:GLPG2222_200mg	0	No concerns	Low risk	No concerns	Major concerns	No concerns	No concerns	Low	["Imprecision"]
GLPG2222_150mg:GLPG2222_400mg	0	No concerns	Low risk	No concerns	Major concerns	No concerns	No concerns	Low	["Imprecision"]
GLPG2222_150mg:GLPG2222_50mg	0	No concerns	Low risk	No concerns	Major concerns	No concerns	No concerns	Low	["Imprecision"]

GLPG2222_150mg:GLPG2737_75mg	0	No concerns	Low risk	No concerns	Major concerns	No concerns	No concerns	Low	["Imprecision"]
GLPG2222_150mg:Iva_150mg	0	No concerns	Low risk	No concerns	No concerns	Major concerns	No concerns	Low	["Heterogeneity"]
GLPG2222_150mg:Ola_teza_iva_600mg_50mg_300mg	0	No concerns	Low risk	No concerns	No concerns	Major concerns	No concerns	Low	["Heterogeneity"]
GLPG2222_150mg:Teza_iva_100mg_150mg	0	No concerns	Low risk	No concerns	No concerns	Major concerns	No concerns	Low	["Heterogeneity"]
GLPG2222_150mg:Teza_iva_100mg_50mg	0	No concerns	Low risk	No concerns	Major concerns	No concerns	No concerns	Low	["Imprecision"]
GLPG2222_150mg:Teza_iva_50mg_150mg	0	No concerns	Low risk	No concerns	Major concerns	No concerns	No concerns	Low	["Imprecision"]
GLPG2222_150mg:Teza_iva_50mg_300mg	0	No concerns	Low risk	No concerns	Major concerns	No concerns	No concerns	Low	["Imprecision"]
GLPG2222_150mg:VX152_teza_iva_100mg_100mg_150mg	0	No concerns	Low risk	No concerns	No concerns	Major concerns	No concerns	Low	["Heterogeneity"]
GLPG2222_150mg:VX152_teza_iva_200mg_100mg_150mg	0	No concerns	Low risk	No concerns	No concerns	No concerns	No concerns	High	NA
GLPG2222_150mg:VX152_teza_iva_300mg_100mg_150mg	0	No concerns	Low risk	No concerns	No concerns	No concerns	No concerns	High	NA
GLPG2222_150mg:luma_100mg	0	Some concerns	Low risk	No concerns	Major concerns	No concerns	No concerns	Low	["Within-study bias", Imprecision"]
GLPG2222_150mg:luma_200mg	0	Some concerns	Low risk	No concerns	Major concerns	No concerns	No concerns	Low	["Within-study bias", Imprecision"]
GLPG2222_150mg:luma_25mg	0	Some concerns	Low risk	No concerns	Major concerns	No concerns	No concerns	Low	["Within-study bias", Imprecision"]
GLPG2222_150mg:luma_50mg	0	Some concerns	Low risk	No concerns	Major concerns	No concerns	No concerns	Low	["Within-study bias", Imprecision"]
GLPG2222_150mg:luma_iva_200mg_150mg	0	No concerns	Low risk	No concerns	Major concerns	No concerns	No concerns	Low	["Imprecision"]

GLPG2222_150mg:luma_iva_200mg_250mg	0	No concerns	Low risk	No concerns	Major concerns	No concerns	No concerns	Low	["Imprecision"]
GLPG2222_150mg:luma_iva_400mg_250mg	0	No concerns	Low risk	No concerns	Major concerns	No concerns	No concerns	Low	["Imprecision"]
GLPG2222_150mg:luma_iva_600mg_250mg	0	No concerns	Low risk	No concerns	Major concerns	No concerns	No concerns	Low	["Imprecision"]
GLPG2222_150mg:vanza_teza_deuti_10mg_100mg_150mg	0	No concerns	Low risk	No concerns	No concerns	No concerns	No concerns	High	NA
GLPG2222_150mg:vanza_teza_deuti_20mg_100mg_150mg	0	No concerns	Low risk	No concerns	No concerns	No concerns	No concerns	High	NA
GLPG2222_150mg:vanza_teza_deuti_5mg_100mg_150mg	0	No concerns	Low risk	No concerns	Major concerns	No concerns	No concerns	Low	["Imprecision"]
GLPG2222_150mg:vanza_teza_iva_5mg_100mg_150mg	0	No concerns	Low risk	No concerns	Major concerns	No concerns	No concerns	Low	["Imprecision"]
GLPG2222_200mg:GLPG2222_300mg	0	No concerns	Low risk	No concerns	Major concerns	No concerns	No concerns	Low	["Imprecision"]
GLPG2222_200mg:GLPG2737_75mg	0	No concerns	Low risk	No concerns	Major concerns	No concerns	No concerns	Low	["Imprecision"]
GLPG2222_200mg:Iva_150mg	0	No concerns	Low risk	No concerns	No concerns	Major concerns	No concerns	Low	["Heterogeneity"]
GLPG2222_200mg:Ola_teza_iva_600mg_50mg_300mg	0	No concerns	Low risk	No concerns	No concerns	Major concerns	No concerns	Low	["Heterogeneity"]
GLPG2222_200mg:Teza_iva_100mg_150mg	0	No concerns	Low risk	No concerns	No concerns	Major concerns	No concerns	Low	["Heterogeneity"]
GLPG2222_200mg:Teza_iva_100mg_50mg	0	No concerns	Low risk	No concerns	Major concerns	No concerns	No concerns	Low	["Imprecision"]
GLPG2222_200mg:Teza_iva_50mg_150mg	0	No concerns	Low risk	No concerns	Major concerns	No concerns	No concerns	Low	["Imprecision"]
GLPG2222_200mg:Teza_iva_50mg_300mg	0	No concerns	Low risk	No concerns	Major concerns	No concerns	No concerns	Low	["Imprecision"]
GLPG2222_200mg:VX152_teza_iva_100mg_100mg_150mg	0	No concerns	Low risk	No concerns	No concerns	Major concerns	No concerns	Low	["Heterogeneity"]
GLPG2222_200mg:VX152_teza_iva_200mg_100mg_150mg	0	No concerns	Low risk	No concerns	No concerns	No concerns	No concerns	High	NA

GLPG2222_200mg:VX152_teza_iva_300mg_100mg_150mg	0	No concerns	Low risk	No concerns	No concerns	No concerns	No concerns	High	NA
GLPG2222_200mg:luma_100mg	0	No concerns	Low risk	No concerns	Major concerns	No concerns	No concerns	Low	["Imprecision"]
GLPG2222_200mg:luma_200mg	0	Some concerns	Low risk	No concerns	Major concerns	No concerns	No concerns	Low	["Within-study bias", Imprecision"]
GLPG2222_200mg:luma_25mg	0	No concerns	Low risk	No concerns	Major concerns	No concerns	No concerns	Low	["Imprecision"]
GLPG2222_200mg:luma_50mg	0	Some concerns	Low risk	No concerns	Major concerns	No concerns	No concerns	Low	["Within-study bias", Imprecision"]
GLPG2222_200mg:luma_iva_200mg_150mg	0	No concerns	Low risk	No concerns	Major concerns	No concerns	No concerns	Low	["Imprecision"]
GLPG2222_200mg:luma_iva_200mg_250mg	0	No concerns	Low risk	No concerns	Major concerns	No concerns	No concerns	Low	["Imprecision"]
GLPG2222_200mg:luma_iva_400mg_250mg	0	No concerns	Low risk	No concerns	Major concerns	No concerns	No concerns	Low	["Imprecision"]
GLPG2222_200mg:luma_iva_600mg_250mg	0	No concerns	Low risk	No concerns	Major concerns	No concerns	No concerns	Low	["Imprecision"]
GLPG2222_200mg:vanza_teza_deuti_10mg_100mg_150mg	0	No concerns	Low risk	No concerns	No concerns	No concerns	No concerns	High	NA
GLPG2222_200mg:vanza_teza_deuti_20mg_100mg_150mg	0	No concerns	Low risk	No concerns	No concerns	No concerns	No concerns	High	NA
GLPG2222_200mg:vanza_teza_deuti_5mg_100mg_150mg	0	No concerns	Low risk	No concerns	Major concerns	No concerns	No concerns	Low	["Imprecision"]
GLPG2222_200mg:vanza_teza_iva_5mg_100mg_150mg	0	No concerns	Low risk	No concerns	Major concerns	No concerns	No concerns	Low	["Imprecision"]
GLPG2222_300mg:GLPG2222_400mg	0	No concerns	Low risk	No concerns	Major concerns	No concerns	No concerns	Low	["Imprecision"]
GLPG2222_300mg:GLPG2222_50mg	0	No concerns	Low risk	No concerns	Major concerns	No concerns	No concerns	Low	["Imprecision"]
GLPG2222_300mg:GLPG2737_75mg	0	No concerns	Low risk	No concerns	Major concerns	No concerns	No concerns	Low	["Imprecision"]

GLPG2222_300mg:Iva_150mg	0	No concerns	Low risk	No concerns	Major concerns	No concerns	No concerns	Low	["Imprecision"]
GLPG2222_300mg:Ola_teza_iva_600mg_50mg_300mg	0	No concerns	Low risk	No concerns	No concerns	Major concerns	No concerns	Low	["Heterogeneity"]
GLPG2222_300mg:Teza_iva_100mg_150mg	0	No concerns	Low risk	No concerns	Major concerns	No concerns	No concerns	Low	["Imprecision"]
GLPG2222_300mg:Teza_iva_100mg_50mg	0	Some concerns	Low risk	No concerns	Major concerns	No concerns	No concerns	Low	["Within-study bias", Imprecision"]
GLPG2222_300mg:Teza_iva_50mg_150mg	0	Some concerns	Low risk	No concerns	Major concerns	No concerns	No concerns	Low	["Within-study bias", Imprecision"]
GLPG2222_300mg:Teza_iva_50mg_300mg	0	No concerns	Low risk	No concerns	Major concerns	No concerns	No concerns	Low	["Imprecision"]
GLPG2222_300mg:VX152_teza_iva_100mg_100mg_150mg	0	No concerns	Low risk	No concerns	Major concerns	No concerns	No concerns	Low	["Imprecision"]
GLPG2222_300mg:VX152_teza_iva_200mg_100mg_150mg	0	No concerns	Low risk	No concerns	No concerns	Major concerns	No concerns	Low	["Heterogeneity"]
GLPG2222_300mg:VX152_teza_iva_300mg_100mg_150mg	0	No concerns	Low risk	No concerns	No concerns	Major concerns	No concerns	Low	["Heterogeneity"]
GLPG2222_300mg:luma_100mg	0	Some concerns	Low risk	No concerns	Major concerns	No concerns	No concerns	Low	["Within-study bias", Imprecision"]
GLPG2222_300mg:luma_200mg	0	Some concerns	Low risk	No concerns	Major concerns	No concerns	No concerns	Low	["Within-study bias", Imprecision"]
GLPG2222_300mg:luma_25mg	0	Some concerns	Low risk	No concerns	Major concerns	No concerns	No concerns	Low	["Within-study bias", Imprecision"]
GLPG2222_300mg:luma_50mg	0	Some concerns	Low risk	No concerns	Major concerns	No concerns	No concerns	Low	["Within-study bias", Imprecision"]
GLPG2222_300mg:luma_iva_200mg_150mg	0	No concerns	Low risk	No concerns	Major concerns	No concerns	No concerns	Low	["Imprecision"]
GLPG2222_300mg:luma_iva_200mg_250mg	0	No concerns	Low risk	No concerns	Major concerns	No concerns	No concerns	Low	["Imprecision"]

GLPG2222_300mg:luma_iva_400mg_250mg	0	No concerns	Low risk	No concerns	Major concerns	No concerns	No concerns	Low	["Imprecision"]
GLPG2222_300mg:luma_iva_600mg_250mg	0	No concerns	Low risk	No concerns	Major concerns	No concerns	No concerns	Low	["Imprecision"]
GLPG2222_300mg:vanza_teza_deuti_10mg_100mg_150mg	0	No concerns	Low risk	No concerns	No concerns	No concerns	No concerns	High	NA
GLPG2222_300mg:vanza_teza_deuti_20mg_100mg_150mg	0	No concerns	Low risk	No concerns	No concerns	No concerns	No concerns	High	NA
GLPG2222_300mg:vanza_teza_deuti_5mg_100mg_150mg	0	No concerns	Low risk	No concerns	Major concerns	No concerns	No concerns	Low	["Imprecision"]
GLPG2222_300mg:vanza_teza_iva_5mg_100mg_150mg	0	No concerns	Low risk	No concerns	Major concerns	No concerns	No concerns	Low	["Imprecision"]
GLPG2222_400mg:GLPG2737_75mg	0	No concerns	Low risk	No concerns	Major concerns	No concerns	No concerns	Low	["Imprecision"]
GLPG2222_400mg:Iva_150mg	0	No concerns	Low risk	No concerns	Major concerns	No concerns	No concerns	Low	["Imprecision"]
GLPG2222_400mg:Ola_teza_iva_600mg_50mg_300mg	0	No concerns	Low risk	No concerns	No concerns	Major concerns	No concerns	Low	["Heterogeneity"]
GLPG2222_400mg:Teza_iva_100mg_150mg	0	No concerns	Low risk	No concerns	Major concerns	No concerns	No concerns	Low	["Imprecision"]
GLPG2222_400mg:Teza_iva_100mg_50mg	0	No concerns	Low risk	No concerns	Major concerns	No concerns	No concerns	Low	["Imprecision"]
GLPG2222_400mg:Teza_iva_50mg_150mg	0	No concerns	Low risk	No concerns	Major concerns	No concerns	No concerns	Low	["Imprecision"]
GLPG2222_400mg:Teza_iva_50mg_300mg	0	No concerns	Low risk	No concerns	Major concerns	No concerns	No concerns	Low	["Imprecision"]
GLPG2222_400mg:VX152_teza_iva_100mg_100mg_150mg	0	No concerns	Low risk	No concerns	Major concerns	No concerns	No concerns	Low	["Imprecision"]
GLPG2222_400mg:VX152_teza_iva_200mg_100mg_150mg	0	No concerns	Low risk	No concerns	No concerns	Major concerns	No concerns	Low	["Heterogeneity"]
GLPG2222_400mg:VX152_teza_iva_300mg_100mg_150mg	0	No concerns	Low risk	No concerns	No concerns	Major concerns	No concerns	Low	["Heterogeneity"]
GLPG2222_400mg:luma_100mg	0	No concerns	Low risk	No concerns	Major concerns	No concerns	No concerns	Low	["Imprecision"]

GLPG2222_400mg:luma_200mg	0	No concerns	Low risk	No concerns	Major concerns	No concerns	No concerns	Low	["Imprecision"]
GLPG2222_400mg:luma_25mg	0	No concerns	Low risk	No concerns	Major concerns	No concerns	No concerns	Low	["Imprecision"]
GLPG2222_400mg:luma_50mg	0	No concerns	Low risk	No concerns	Major concerns	No concerns	No concerns	Low	["Imprecision"]
GLPG2222_400mg:luma_iva_200mg_150mg	0	No concerns	Low risk	No concerns	Major concerns	No concerns	No concerns	Low	["Imprecision"]
GLPG2222_400mg:luma_iva_200mg_250mg	0	No concerns	Low risk	No concerns	Major concerns	No concerns	No concerns	Low	["Imprecision"]
GLPG2222_400mg:luma_iva_400mg_250mg	0	No concerns	Low risk	No concerns	Major concerns	No concerns	No concerns	Low	["Imprecision"]
GLPG2222_400mg:luma_iva_600mg_250mg	0	No concerns	Low risk	No concerns	Major concerns	No concerns	No concerns	Low	["Imprecision"]
GLPG2222_400mg:vanza_teza_deuti_10mg_100mg_150mg	0	No concerns	Low risk	No concerns	No concerns	No concerns	No concerns	High	NA
GLPG2222_400mg:vanza_teza_deuti_20mg_100mg_150mg	0	No concerns	Low risk	No concerns	No concerns	No concerns	No concerns	High	NA
GLPG2222_400mg:vanza_teza_deuti_5mg_100mg_150mg	0	No concerns	Low risk	No concerns	Major concerns	No concerns	No concerns	Low	["Imprecision"]
GLPG2222_400mg:vanza_teza_iva_5mg_100mg_150mg	0	No concerns	Low risk	No concerns	Major concerns	No concerns	No concerns	Low	["Imprecision"]
GLPG2222_50mg:GLPG2737_75mg	0	No concerns	Low risk	No concerns	Major concerns	No concerns	No concerns	Low	["Imprecision"]
GLPG2222_50mg:Iva_150mg	0	No concerns	Low risk	No concerns	Major concerns	No concerns	No concerns	Low	["Imprecision"]
GLPG2222_50mg:Ola_teza_iva_600mg_50mg_300mg	0	No concerns	Low risk	No concerns	No concerns	Major concerns	No concerns	Low	["Heterogeneity"]
GLPG2222_50mg:Teza_iva_100mg_150mg	0	No concerns	Low risk	No concerns	No concerns	Major concerns	No concerns	Low	["Heterogeneity"]
GLPG2222_50mg:Teza_iva_100mg_50mg	0	No concerns	Low risk	No concerns	Major concerns	No concerns	No concerns	Low	["Imprecision"]
GLPG2222_50mg:Teza_iva_50mg_150mg	0	No concerns	Low risk	No concerns	Major concerns	No concerns	No concerns	Low	["Imprecision"]

GLPG2222_50mg:Teza_iva_50mg_300mg	0	No concerns	Low risk	No concerns	Major concerns	No concerns	No concerns	Low	["Imprecision"]
GLPG2222_50mg:VX152_teza_iva_100mg_100mg_150mg	0	No concerns	Low risk	No concerns	No concerns	Major concerns	No concerns	Low	["Heterogeneity"]
GLPG2222_50mg:VX152_teza_iva_200mg_100mg_150mg	0	No concerns	Low risk	No concerns	No concerns	No concerns	No concerns	High	NA
GLPG2222_50mg:VX152_teza_iva_300mg_100mg_150mg	0	No concerns	Low risk	No concerns	No concerns	No concerns	No concerns	High	NA
GLPG2222_50mg:luma_100mg	0	No concerns	Low risk	No concerns	Major concerns	No concerns	No concerns	Low	["Imprecision"]
GLPG2222_50mg:luma_200mg	0	No concerns	Low risk	No concerns	Major concerns	No concerns	No concerns	Low	["Imprecision"]
GLPG2222_50mg:luma_25mg	0	No concerns	Low risk	No concerns	Major concerns	No concerns	No concerns	Low	["Imprecision"]
GLPG2222_50mg:luma_50mg	0	No concerns	Low risk	No concerns	Major concerns	No concerns	No concerns	Low	["Imprecision"]
GLPG2222_50mg:luma_iva_200mg_150mg	0	No concerns	Low risk	No concerns	Major concerns	No concerns	No concerns	Low	["Imprecision"]
GLPG2222_50mg:luma_iva_200mg_250mg	0	No concerns	Low risk	No concerns	Major concerns	No concerns	No concerns	Low	["Imprecision"]
GLPG2222_50mg:luma_iva_400mg_250mg	0	No concerns	Low risk	No concerns	Major concerns	No concerns	No concerns	Low	["Imprecision"]
GLPG2222_50mg:luma_iva_600mg_250mg	0	No concerns	Low risk	No concerns	Major concerns	No concerns	No concerns	Low	["Imprecision"]
GLPG2222_50mg:vanza_teza_deuti_10mg_100mg_150mg	0	No concerns	Low risk	No concerns	No concerns	No concerns	No concerns	High	NA
GLPG2222_50mg:vanza_teza_deuti_20mg_100mg_150mg	0	No concerns	Low risk	No concerns	No concerns	No concerns	No concerns	Low	NA
GLPG2222_50mg:vanza_teza_deuti_5mg_100mg_150mg	0	No concerns	Low risk	No concerns	Major concerns	No concerns	No concerns	Low	["Imprecision"]
GLPG2222_50mg:vanza_teza_iva_5mg_100mg_150mg	0	No concerns	Low risk	No concerns	Major concerns	No concerns	No concerns	Low	["Imprecision"]
GLPG2737_75mg:Iva_150mg	0	No concerns	Low risk	No concerns	Major concerns	No concerns	No concerns	Low	["Imprecision"]

GLPG2737_75mg:Ola_teza_iva_600mg_50mg_300mg	0	No concerns	Low risk	No concerns	No concerns	Major concerns	No concerns	Low	["Heterogeneity"]
GLPG2737_75mg:Teza_iva_100mg_150mg	0	No concerns	Low risk	No concerns	Major concerns	No concerns	No concerns	Low	["Imprecision"]
GLPG2737_75mg:Teza_iva_100mg_50mg	0	Some concerns	Low risk	No concerns	Major concerns	No concerns	No concerns	Low	["Within-study bias", Imprecision"]
GLPG2737_75mg:Teza_iva_50mg_150mg	0	Some concerns	Low risk	No concerns	Major concerns	No concerns	No concerns	Low	["Within-study bias", Imprecision"]
GLPG2737_75mg:Teza_iva_50mg_300mg	0	No concerns	Low risk	No concerns	Major concerns	No concerns	No concerns	Low	["Imprecision"]
GLPG2737_75mg:VX152_teza_iva_100mg_100mg_150mg	0	No concerns	Low risk	No concerns	Major concerns	No concerns	No concerns	Low	["Imprecision"]
GLPG2737_75mg:VX152_teza_iva_200mg_100mg_150mg	0	No concerns	Low risk	No concerns	No concerns	Major concerns	No concerns	Low	["Heterogeneity"]
GLPG2737_75mg:VX152_teza_iva_300mg_100mg_150mg	0	No concerns	Low risk	No concerns	No concerns	Major concerns	No concerns	Low	["Heterogeneity"]
GLPG2737_75mg:luma_100mg	0	Some concerns	Low risk	No concerns	Major concerns	No concerns	No concerns	Low	["Within-study bias", Imprecision"]
GLPG2737_75mg:luma_200mg	0	Some concerns	Low risk	No concerns	Major concerns	No concerns	No concerns	Low	["Within-study bias", Imprecision"]
GLPG2737_75mg:luma_25mg	0	Some concerns	Low risk	No concerns	Major concerns	No concerns	No concerns	Low	["Within-study bias", Imprecision"]
GLPG2737_75mg:luma_50mg	0	Some concerns	Low risk	No concerns	Major concerns	No concerns	No concerns	Low	["Within-study bias", Imprecision"]
GLPG2737_75mg:luma_iva_200mg_150mg	0	No concerns	Low risk	No concerns	Major concerns	No concerns	No concerns	Low	["Imprecision"]
GLPG2737_75mg:luma_iva_200mg_250mg	0	No concerns	Low risk	No concerns	Major concerns	No concerns	No concerns	Low	["Imprecision"]
GLPG2737_75mg:luma_iva_400mg_250mg	0	No concerns	Low risk	No concerns	Major concerns	No concerns	No concerns	Low	["Imprecision"]

GLPG2737_75mg:luma_iva_600mg_250mg	0	No concerns	Low risk	No concerns	Major concerns	No concerns	No concerns	Low	["Imprecision"]
GLPG2737_75mg:vanza_teza_deuti_10mg_100mg_150mg	0	No concerns	Low risk	No concerns	No concerns	No concerns	No concerns	Low	NA
GLPG2737_75mg:vanza_teza_deuti_20mg_100mg_150mg	0	No concerns	Low risk	No concerns	No concerns	Major concerns	No concerns	Low	["Heterogeneity"]
GLPG2737_75mg:vanza_teza_deuti_5mg_100mg_150mg	0	No concerns	Low risk	No concerns	Major concerns	No concerns	No concerns	Low	["Imprecision"]
GLPG2737_75mg:vanza_teza_iva_5mg_100mg_150mg	0	No concerns	Low risk	No concerns	Major concerns	No concerns	No concerns	Low	["Imprecision"]
Iva_150mg:Ola_teza_iva_600mg_50mg_300mg	0	No concerns	Low risk	No concerns	No concerns	Major concerns	No concerns	Low	["Heterogeneity"]
Iva_150mg:Teza_iva_100mg_50mg	0	Some concerns	Low risk	No concerns	No concerns	Major concerns	No concerns	Low	["Within-study bias", "Heterogeneity"]
Iva_150mg:Teza_iva_50mg_150mg	0	Some concerns	Low risk	No concerns	No concerns	Major concerns	No concerns	Low	["Within-study bias", "Heterogeneity"]
Iva_150mg:Teza_iva_50mg_300mg	0	No concerns	Low risk	No concerns	Major concerns	No concerns	No concerns	Low	["Imprecision"]
Iva_150mg:VX152_teza_iva_100mg_100mg_150mg	0	No concerns	Low risk	No concerns	Major concerns	No concerns	No concerns	Low	["Imprecision"]
Iva_150mg:VX152_teza_iva_200mg_100mg_150mg	0	No concerns	Low risk	No concerns	No concerns	Major concerns	No concerns	Low	["Heterogeneity"]
Iva_150mg:VX152_teza_iva_300mg_100mg_150mg	0	No concerns	Low risk	No concerns	No concerns	Major concerns	No concerns	Low	["Heterogeneity"]
Iva_150mg:luma_100mg	0	Some concerns	Low risk	No concerns	Major concerns	No concerns	No concerns	Low	["Within-study bias", Imprecision"]
Iva_150mg:luma_200mg	0	Some concerns	Low risk	No concerns	Major concerns	No concerns	No concerns	Low	["Within-study bias", Imprecision"]
Iva_150mg:luma_25mg	0	Some concerns	Low risk	No concerns	Major concerns	No concerns	No concerns	Low	["Within-study bias", Imprecision"]

Iva_150mg:luma_50mg	0	Some concerns	Low risk	No concerns	Major concerns	No concerns	No concerns	Low	["Within-study bias", Imprecision"]
Iva_150mg:luma_iva_200mg_150mg	0	No concerns	Low risk	No concerns	Major concerns	No concerns	No concerns	Low	["Imprecision"]
Iva_150mg:luma_iva_200mg_250mg	0	No concerns	Low risk	No concerns	No concerns	Major concerns	No concerns	Low	["Heterogeneity"]
Iva_150mg:luma_iva_400mg_250mg	0	No concerns	Low risk	No concerns	No concerns	Major concerns	No concerns	Low	["Heterogeneity"]
Iva_150mg:luma_iva_600mg_250mg	0	No concerns	Low risk	No concerns	Major concerns	No concerns	No concerns	Low	["Imprecision"]
Iva_150mg:vanza_teza_deuti_10mg_100mg_150mg	0	No concerns	Low risk	No concerns	No concerns	No concerns	No concerns	High	NA
Iva_150mg:vanza_teza_deuti_20mg_100mg_150mg	0	No concerns	Low risk	No concerns	No concerns	No concerns	No concerns	High	NA
Iva_150mg:vanza_teza_deuti_5mg_100mg_150mg	0	No concerns	Low risk	No concerns	Major concerns	No concerns	No concerns	Low	["Imprecision"]
Iva_150mg:vanza_teza_iva_5mg_100mg_150mg	0	No concerns	Low risk	No concerns	Major concerns	No concerns	No concerns	Low	["Imprecision"]
Ola_teza_iva_600mg_50mg_300mg:Teza_iva_100mg_150mg	0	No concerns	Low risk	No concerns	No concerns	Major concerns	No concerns	Low	["Heterogeneity"]
Ola_teza_iva_600mg_50mg_300mg:Teza_iva_100mg_50mg	0	Some concerns	Low risk	No concerns	No concerns	Major concerns	No concerns	Low	["Within-study bias", "Heterogeneity"]
Ola_teza_iva_600mg_50mg_300mg:Teza_iva_50mg_150mg	0	Some concerns	Low risk	No concerns	No concerns	Major concerns	No concerns	Low	["Within-study bias", "Heterogeneity"]
Ola_teza_iva_600mg_50mg_300mg:VX152_teza_iva_100mg_100mg_150mg	0	No concerns	Low risk	No concerns	Major concerns	No concerns	No concerns	Low	["Imprecision"]
Ola_teza_iva_600mg_50mg_300mg:VX152_teza_iva_200mg_100mg_150mg	0	No concerns	Low risk	No concerns	Major concerns	No concerns	No concerns	Low	["Imprecision"]
Ola_teza_iva_600mg_50mg_300mg:VX152_teza_iva_300mg_100mg_150mg	0	No concerns	Low risk	No concerns	Major concerns	No concerns	No concerns	Low	["Imprecision"]

luma_100mg:Ola_teza_iva_600mg_50mg_300mg	0	Some concerns	Low risk	No concerns	Major concerns	No concerns	No concerns	Low	["Within-study bias", Imprecision"]
luma_200mg:Ola_teza_iva_600mg_50mg_300mg	0	Some concerns	Low risk	No concerns	Major concerns	No concerns	No concerns	Low	["Within-study bias", Imprecision"]
luma_25mg:Ola_teza_iva_600mg_50mg_300mg	0	Some concerns	Low risk	No concerns	Major concerns	No concerns	No concerns	Low	["Within-study bias", Imprecision"]
luma_50mg:Ola_teza_iva_600mg_50mg_300mg	0	Some concerns	Low risk	No concerns	Major concerns	No concerns	No concerns	Low	["Within-study bias", Imprecision"]
luma_iva_200mg_150mg:Ola_teza_iva_600mg_50mg_300mg	0	No concerns	Low risk	No concerns	No concerns	Major concerns	No concerns	Low	["Heterogeneity"]
luma_iva_200mg_250mg:Ola_teza_iva_600mg_50mg_300mg	0	No concerns	Low risk	No concerns	No concerns	Major concerns	No concerns	Low	["Heterogeneity"]
luma_iva_400mg_250mg:Ola_teza_iva_600mg_50mg_300mg	0	No concerns	Low risk	No concerns	No concerns	Major concerns	No concerns	Low	["Heterogeneity"]
luma_iva_600mg_250mg:Ola_teza_iva_600mg_50mg_300mg	0	No concerns	Low risk	No concerns	Major concerns	No concerns	No concerns	Low	["Imprecision"]
Ola_teza_iva_600mg_50mg_300mg:vanza_teza_deuti_10mg_100mg_150mg	0	No concerns	Low risk	No concerns	Major concerns	No concerns	No concerns	Low	["Imprecision"]
Ola_teza_iva_600mg_50mg_300mg:vanza_teza_deuti_20mg_100mg_150mg	0	No concerns	Low risk	No concerns	Major concerns	No concerns	No concerns	Low	["Imprecision"]
Ola_teza_iva_600mg_50mg_300mg:vanza_teza_deuti_5mg_100mg_150mg	0	No concerns	Low risk	No concerns	Major concerns	No concerns	No concerns	Low	["Imprecision"]
Ola_teza_iva_600mg_50mg_300mg:vanza_teza_iva_5mg_100mg_150mg	0	No concerns	Low risk	No concerns	Major concerns	No concerns	No concerns	Low	["Imprecision"]
Placebo:Teza_iva_50mg_300mg	0	No concerns	Low risk	No concerns	Major concerns	No concerns	No concerns	Low	["Imprecision"]
Teza_iva_100mg_150mg:Teza_iva_100mg_50mg	0	No concerns	Low risk	No concerns	No concerns	Major concerns	No concerns	Low	["Heterogeneity"]
Teza_iva_100mg_150mg:Teza_iva_50mg_150mg	0	No concerns	Low risk	No concerns	No concerns	Major concerns	No concerns	Low	["Heterogeneity"]

Teza_iva_100mg_150mg:Teza_iva_50mg_300mg	0	No concerns	Low risk	No concerns	Major concerns	No concerns	No concerns	Low	["Imprecision"]
Teza_iva_100mg_150mg:VX152_teza_iva_100mg_100mg_150 mg	0	No concerns	Low risk	No concerns	Major concerns	No concerns	No concerns	Low	["Imprecision"]
luma_100mg:Teza_iva_100mg_150mg	0	Some concerns	Low risk	No concerns	Major concerns	No concerns	No concerns	Low	["Within-study bias", Imprecision"]
luma_200mg:Teza_iva_100mg_150mg	0	Some concerns	Low risk	No concerns	Major concerns	No concerns	No concerns	Low	["Within-study bias", Imprecision"]
luma_25mg:Teza_iva_100mg_150mg	0	Some concerns	Low risk	No concerns	Major concerns	No concerns	No concerns	Low	["Within-study bias", Imprecision"]
luma_50mg:Teza_iva_100mg_150mg	0	Some concerns	Low risk	No concerns	Major concerns	No concerns	No concerns	Low	["Within-study bias", Imprecision"]
luma_iva_200mg_150mg:Teza_iva_100mg_150mg	0	No concerns	Low risk	No concerns	Major concerns	No concerns	No concerns	Low	["Imprecision"]
luma_iva_200mg_250mg:Teza_iva_100mg_150mg	0	No concerns	Low risk	No concerns	No concerns	Major concerns	No concerns	Low	["Heterogeneity"]
luma_iva_400mg_250mg:Teza_iva_100mg_150mg	0	No concerns	Low risk	No concerns	No concerns	Major concerns	No concerns	Low	["Heterogeneity"]
luma_iva_600mg_250mg:Teza_iva_100mg_150mg	0	No concerns	Low risk	No concerns	Major concerns	No concerns	No concerns	Low	["Imprecision"]
Teza_iva_100mg_150mg:vanza_teza_deuti_10mg_100mg_150 mg	0	No concerns	Low risk	No concerns	No concerns	No concerns	No concerns	High	NA
Teza_iva_100mg_150mg:vanza_teza_deuti_5mg_100mg_150m g	0	No concerns	Low risk	No concerns	Major concerns	No concerns	No concerns	Low	["Imprecision"]
Teza_iva_100mg_150mg:vanza_teza_iva_5mg_100mg_150mg	0	No concerns	Low risk	No concerns	Major concerns	No concerns	No concerns	Low	["Imprecision"]
Teza_iva_100mg_50mg:Teza_iva_50mg_300mg	0	No concerns	Low risk	No concerns	Major concerns	No concerns	No concerns	Low	["Imprecision"]
Teza_iva_100mg_50mg:VX152_teza_iva_100mg_100mg_150 mg	0	No concerns	Low risk	No concerns	No concerns	Major concerns	No concerns	Low	["Heterogeneity"]

Teza_iva_100mg_50mg:VX152_teza_iva_200mg_100mg_150mg	0	No concerns	Low risk	No concerns	No concerns	No concerns	No concerns	High	NA
Teza_iva_100mg_50mg:VX152_teza_iva_300mg_100mg_150mg	0	No concerns	Low risk	No concerns	No concerns	No concerns	No concerns	High	NA
luma_100mg:Teza_iva_100mg_50mg	0	Some concerns	Low risk	No concerns	Major concerns	No concerns	No concerns	Low	["Within-study bias", Imprecision"]
luma_200mg:Teza_iva_100mg_50mg	0	Some concerns	Low risk	No concerns	Major concerns	No concerns	No concerns	Low	["Within-study bias", Imprecision"]
luma_25mg:Teza_iva_100mg_50mg	0	Some concerns	Low risk	No concerns	Major concerns	No concerns	No concerns	Low	["Within-study bias", Imprecision"]
luma_50mg:Teza_iva_100mg_50mg	0	Some concerns	Low risk	No concerns	Major concerns	No concerns	No concerns	Low	["Within-study bias", Imprecision"]
luma_iva_200mg_150mg:Teza_iva_100mg_50mg	0	No concerns	Low risk	No concerns	Major concerns	No concerns	No concerns	Low	["Imprecision"]
luma_iva_200mg_250mg:Teza_iva_100mg_50mg	0	Some concerns	Low risk	No concerns	Major concerns	No concerns	No concerns	Low	["Within-study bias", Imprecision"]
luma_iva_400mg_250mg:Teza_iva_100mg_50mg	0	Some concerns	Low risk	No concerns	Major concerns	No concerns	No concerns	Low	["Within-study bias", Imprecision"]
luma_iva_600mg_250mg:Teza_iva_100mg_50mg	0	Some concerns	Low risk	No concerns	Major concerns	No concerns	No concerns	Low	["Within-study bias", Imprecision"]
Teza_iva_100mg_50mg:vanza_teza_deuti_10mg_100mg_150mg	0	No concerns	Low risk	No concerns	No concerns	No concerns	No concerns	High	NA
Teza_iva_100mg_50mg:vanza_teza_deuti_20mg_100mg_150mg	0	No concerns	Low risk	No concerns	No concerns	No concerns	No concerns	Low	NA
Teza_iva_100mg_50mg:vanza_teza_deuti_5mg_100mg_150mg	0	No concerns	Low risk	No concerns	Major concerns	No concerns	No concerns	Low	["Imprecision"]
Teza_iva_100mg_50mg:vanza_teza_iva_5mg_100mg_150mg	0	Some concerns	Low risk	No concerns	Major concerns	No concerns	No concerns	Low	["Within-study bias", Imprecision"]

Teza_iva_50mg_150mg:Teza_iva_50mg_300mg	0	No concerns	Low risk	No concerns	Major concerns	No concerns	No concerns	Low	["Imprecision"]
Teza_iva_50mg_150mg:VX152_teza_iva_100mg_100mg_150 mg	0	No concerns	Low risk	No concerns	No concerns	Major concerns	No concerns	Low	["Heterogeneity"]
Teza_iva_50mg_150mg:VX152_teza_iva_200mg_100mg_150 mg	0	No concerns	Low risk	No concerns	No concerns	No concerns	No concerns	High	NA
Teza_iva_50mg_150mg:VX152_teza_iva_300mg_100mg_150 mg	0	No concerns	Low risk	No concerns	No concerns	No concerns	No concerns	High	NA
luma_100mg:Teza_iva_50mg_150mg	0	Some concerns	Low risk	No concerns	Major concerns	No concerns	No concerns	Low	["Within-study bias", Imprecision"]
luma_200mg:Teza_iva_50mg_150mg	0	Some concerns	Low risk	No concerns	Major concerns	No concerns	No concerns	Low	["Within-study bias", Imprecision"]
luma_25mg:Teza_iva_50mg_150mg	0	Some concerns	Low risk	No concerns	Major concerns	No concerns	No concerns	Low	["Within-study bias", Imprecision"]
luma_50mg:Teza_iva_50mg_150mg	0	Some concerns	Low risk	No concerns	Major concerns	No concerns	No concerns	Low	["Within-study bias", Imprecision"]
luma_iva_200mg_150mg:Teza_iva_50mg_150mg	0	No concerns	Low risk	No concerns	Major concerns	No concerns	No concerns	Low	["Imprecision"]
luma_iva_200mg_250mg:Teza_iva_50mg_150mg	0	Some concerns	Low risk	No concerns	Major concerns	No concerns	No concerns	Low	["Within-study bias", Imprecision"]
luma_iva_400mg_250mg:Teza_iva_50mg_150mg	0	Some concerns	Low risk	No concerns	Major concerns	No concerns	No concerns	Low	["Within-study bias", Imprecision"]
luma_iva_600mg_250mg:Teza_iva_50mg_150mg	0	Some concerns	Low risk	No concerns	Major concerns	No concerns	No concerns	Low	["Within-study bias", Imprecision"]
Teza_iva_50mg_150mg:vanza_teza_deuti_10mg_100mg_150m g	0	No concerns	Low risk	No concerns	No concerns	No concerns	No concerns	High	NA
Teza_iva_50mg_150mg:vanza_teza_deuti_20mg_100mg_150m g	0	No concerns	Low risk	No concerns	No concerns	No concerns	No concerns	High	NA

Teza_iva_50mg_150mg:vanza_teza_deuti_5mg_100mg_150mg	0	No concerns	Low risk	No concerns	Major concerns	No concerns	No concerns	Low	["Imprecision"]
Teza_iva_50mg_150mg:vanza_teza_iva_5mg_100mg_150mg	0	Some concerns	Low risk	No concerns	Major concerns	No concerns	No concerns	Low	["Within-study bias", Imprecision"]
Teza_iva_50mg_300mg:VX152_teza_iva_100mg_100mg_150 mg	0	No concerns	Low risk	No concerns	No concerns	Major concerns	No concerns	Low	["Heterogeneity"]
Teza_iva_50mg_300mg:VX152_teza_iva_200mg_100mg_150 mg	0	No concerns	Low risk	No concerns	No concerns	Major concerns	No concerns	Low	["Heterogeneity"]
Teza_iva_50mg_300mg:VX152_teza_iva_300mg_100mg_150 mg	0	No concerns	Low risk	No concerns	No concerns	Major concerns	No concerns	Low	["Heterogeneity"]
luma_100mg:Teza_iva_50mg_300mg	0	No concerns	Low risk	No concerns	Major concerns	No concerns	No concerns	Low	["Imprecision"]
luma_200mg:Teza_iva_50mg_300mg	0	No concerns	Low risk	No concerns	Major concerns	No concerns	No concerns	Low	["Imprecision"]
luma_25mg:Teza_iva_50mg_300mg	0	No concerns	Low risk	No concerns	Major concerns	No concerns	No concerns	Low	["Imprecision"]
luma_50mg:Teza_iva_50mg_300mg	0	No concerns	Low risk	No concerns	Major concerns	No concerns	No concerns	Low	["Imprecision"]
luma_iva_200mg_150mg:Teza_iva_50mg_300mg	0	No concerns	Low risk	No concerns	Major concerns	No concerns	No concerns	Low	["Imprecision"]
luma_iva_200mg_250mg:Teza_iva_50mg_300mg	0	No concerns	Low risk	No concerns	Major concerns	No concerns	No concerns	Low	["Imprecision"]
luma_iva_400mg_250mg:Teza_iva_50mg_300mg	0	No concerns	Low risk	No concerns	Major concerns	No concerns	No concerns	Low	["Imprecision"]
luma_iva_600mg_250mg:Teza_iva_50mg_300mg	0	No concerns	Low risk	No concerns	Major concerns	No concerns	No concerns	Low	["Imprecision"]
Teza_iva_50mg_300mg:vanza_teza_deuti_10mg_100mg_150m g	0	No concerns	Low risk	No concerns	No concerns	No concerns	No concerns	High	NA
Teza_iva_50mg_300mg:vanza_teza_deuti_20mg_100mg_150m g	0	No concerns	Low risk	No concerns	No concerns	No concerns	No concerns	High	NA
Teza_iva_50mg_300mg:vanza_teza_deuti_5mg_100mg_150mg	0	No concerns	Low risk	No concerns	Major concerns	No concerns	No concerns	Low	["Imprecision"]

Teza_iva_50mg_300mg:vanza_teza_iva_5mg_100mg_150mg	0	No concerns	Low risk	No concerns	Major concerns	No concerns	No concerns	Low	["Imprecision"]
luma_100mg:VX152_teza_iva_100mg_100mg_150mg	0	No concerns	Low risk	No concerns	Major concerns	No concerns	No concerns	Low	["Imprecision"]
luma_200mg:VX152_teza_iva_100mg_100mg_150mg	0	No concerns	Low risk	No concerns	Major concerns	No concerns	No concerns	Low	["Imprecision"]
luma_25mg:VX152_teza_iva_100mg_100mg_150mg	0	No concerns	Low risk	No concerns	Major concerns	No concerns	No concerns	Low	["Imprecision"]
luma_50mg:VX152_teza_iva_100mg_100mg_150mg	0	No concerns	Low risk	No concerns	Major concerns	No concerns	No concerns	Low	["Imprecision"]
luma_iva_200mg_150mg:VX152_teza_iva_100mg_100mg_150mg	0	No concerns	Low risk	No concerns	Major concerns	No concerns	No concerns	Low	["Imprecision"]
luma_iva_200mg_250mg:VX152_teza_iva_100mg_100mg_150mg	0	No concerns	Low risk	No concerns	Major concerns	No concerns	No concerns	Low	["Imprecision"]
luma_iva_400mg_250mg:VX152_teza_iva_100mg_100mg_150mg	0	No concerns	Low risk	No concerns	No concerns	Major concerns	No concerns	Low	["Heterogeneity"]
luma_iva_600mg_250mg:VX152_teza_iva_100mg_100mg_150mg	0	No concerns	Low risk	No concerns	Major concerns	No concerns	No concerns	Low	["Imprecision"]
vanza_teza_deuti_10mg_100mg_150mg:VX152_teza_iva_100mg_100mg_150mg	0	No concerns	Low risk	No concerns	No concerns	Major concerns	No concerns	Low	["Heterogeneity"]
vanza_teza_deuti_20mg_100mg_150mg:VX152_teza_iva_100mg_100mg_150mg	0	No concerns	Low risk	No concerns	Major concerns	No concerns	No concerns	Low	["Imprecision"]
vanza_teza_deuti_5mg_100mg_150mg:VX152_teza_iva_100mg_100mg_150mg	0	No concerns	Low risk	No concerns	Major concerns	No concerns	No concerns	Low	["Imprecision"]
vanza_teza_iva_5mg_100mg_150mg:VX152_teza_iva_100mg_100mg_150mg	0	No concerns	Low risk	No concerns	Major concerns	No concerns	No concerns	Low	["Imprecision"]
luma_100mg:VX152_teza_iva_200mg_100mg_150mg	0	No concerns	Low risk	No concerns	Major concerns	No concerns	No concerns	Low	["Imprecision"]
luma_200mg:VX152_teza_iva_200mg_100mg_150mg	0	No concerns	Low risk	No concerns	Major concerns	No concerns	No concerns	Low	["Imprecision"]
luma_25mg:VX152_teza_iva_200mg_100mg_150mg	0	No concerns	Low risk	No concerns	Major concerns	No concerns	No concerns	Low	["Imprecision"]
luma_50mg:VX152_teza_iva_200mg_100mg_150mg	0	No concerns	Low risk	No concerns	Major concerns	No concerns	No concerns	Low	["Imprecision"]

luma_iva_200mg_150mg:VX152_teza_iva_200mg_100mg_150mg	0	No concerns	Low risk	No concerns	No concerns	Major concerns	No concerns	Low	["Heterogeneity"]
luma_iva_200mg_250mg:VX152_teza_iva_200mg_100mg_150mg	0	No concerns	Low risk	No concerns	No concerns	No concerns	No concerns	High	NA
luma_iva_400mg_250mg:VX152_teza_iva_200mg_100mg_150mg	0	No concerns	Low risk	No concerns	No concerns	No concerns	No concerns	High	NA
luma_iva_600mg_250mg:VX152_teza_iva_200mg_100mg_150mg	0	No concerns	Low risk	No concerns	No concerns	Major concerns	No concerns	Low	["Heterogeneity"]
vanza_teza_deuti_10mg_100mg_150mg:VX152_teza_iva_200mg_100mg_150mg	0	No concerns	Low risk	No concerns	Major concerns	No concerns	No concerns	Low	["Imprecision"]
vanza_teza_deuti_20mg_100mg_150mg:VX152_teza_iva_200mg_100mg_150mg	0	No concerns	Low risk	No concerns	Major concerns	No concerns	No concerns	Low	["Imprecision"]
vanza_teza_deuti_5mg_100mg_150mg:VX152_teza_iva_200mg_100mg_150mg	0	No concerns	Low risk	No concerns	Major concerns	No concerns	No concerns	Low	["Imprecision"]
vanza_teza_iva_5mg_100mg_150mg:VX152_teza_iva_200mg_100mg_150mg	0	No concerns	Low risk	No concerns	Major concerns	No concerns	No concerns	Low	["Imprecision"]
luma_100mg:VX152_teza_iva_300mg_100mg_150mg	0	No concerns	Low risk	No concerns	Major concerns	No concerns	No concerns	Low	["Imprecision"]
luma_200mg:VX152_teza_iva_300mg_100mg_150mg	0	No concerns	Low risk	No concerns	Major concerns	No concerns	No concerns	Low	["Imprecision"]
luma_25mg:VX152_teza_iva_300mg_100mg_150mg	0	No concerns	Low risk	No concerns	Major concerns	No concerns	No concerns	Low	["Imprecision"]
luma_50mg:VX152_teza_iva_300mg_100mg_150mg	0	No concerns	Low risk	No concerns	Major concerns	No concerns	No concerns	Low	["Imprecision"]
luma_iva_200mg_150mg:VX152_teza_iva_300mg_100mg_150mg	0	No concerns	Low risk	No concerns	No concerns	Major concerns	No concerns	Low	["Heterogeneity"]
luma_iva_200mg_250mg:VX152_teza_iva_300mg_100mg_150mg	0	No concerns	Low risk	No concerns	No concerns	No concerns	No concerns	High	NA
luma_iva_400mg_250mg:VX152_teza_iva_300mg_100mg_150mg	0	No concerns	Low risk	No concerns	No concerns	No concerns	No concerns	High	NA
luma_iva_600mg_250mg:VX152_teza_iva_300mg_100mg_150mg	0	No concerns	Low risk	No concerns	No concerns	Major concerns	No concerns	Low	["Heterogeneity"]
vanza_teza_deuti_10mg_100mg_150mg:VX152_teza_iva_300mg_100mg_150mg	0	No concerns	Low risk	No concerns	Major concerns	No concerns	No concerns	Low	["Imprecision"]

vanza_teza_deuti_20mg_100mg_150mg:VX152_teza_iva_300mg_100mg_150mg	0	No concerns	Low risk	No concerns	Major concerns	No concerns	No concerns	Low	["Imprecision"]
vanza_teza_deuti_5mg_100mg_150mg:VX152_teza_iva_300mg_100mg_150mg	0	No concerns	Low risk	No concerns	Major concerns	No concerns	No concerns	Low	["Imprecision"]
vanza_teza_iva_5mg_100mg_150mg:VX152_teza_iva_300mg_100mg_150mg	0	No concerns	Low risk	No concerns	Major concerns	No concerns	No concerns	Low	["Imprecision"]
luma_100mg:luma_iva_200mg_150mg	0	Some concerns	Low risk	No concerns	Major concerns	No concerns	No concerns	Low	["Within-study bias", Imprecision"]
luma_100mg:luma_iva_200mg_250mg	0	Some concerns	Low risk	No concerns	Major concerns	No concerns	No concerns	Low	["Within-study bias", Imprecision"]
luma_100mg:luma_iva_400mg_250mg	0	Some concerns	Low risk	No concerns	Major concerns	No concerns	No concerns	Low	["Within-study bias", Imprecision"]
luma_100mg:luma_iva_600mg_250mg	0	Some concerns	Low risk	No concerns	Major concerns	No concerns	No concerns	Low	["Within-study bias", Imprecision"]
luma_100mg:vanza_teza_deuti_10mg_100mg_150mg	0	No concerns	Low risk	No concerns	Major concerns	No concerns	No concerns	Low	["Imprecision"]
luma_100mg:vanza_teza_deuti_20mg_100mg_150mg	0	No concerns	Low risk	No concerns	Major concerns	No concerns	No concerns	Low	["Imprecision"]
luma_100mg:vanza_teza_deuti_5mg_100mg_150mg	0	No concerns	Low risk	No concerns	Major concerns	No concerns	No concerns	Low	["Imprecision"]
luma_100mg:vanza_teza_iva_5mg_100mg_150mg	0	Some concerns	Low risk	No concerns	Major concerns	No concerns	No concerns	Low	["Within-study bias", Imprecision"]
luma_200mg:luma_iva_200mg_150mg	0	Some concerns	Low risk	No concerns	Major concerns	No concerns	No concerns	Low	["Within-study bias", Imprecision"]
luma_200mg:luma_iva_200mg_250mg	0	Some concerns	Low risk	No concerns	Major concerns	No concerns	No concerns	Low	["Within-study bias", Imprecision"]
luma_200mg:luma_iva_400mg_250mg	0	Some concerns	Low risk	No concerns	Major concerns	No concerns	No concerns	Low	["Within-study bias", Imprecision"]

luma_200mg:luma_iva_600mg_250mg	0	Some concerns	Low risk	No concerns	Major concerns	No concerns	No concerns	Low	["Within-study bias", Imprecision"]
luma_200mg:vanza_teza_deuti_10mg_100mg_150mg	0	No concerns	Low risk	No concerns	Major concerns	No concerns	No concerns	Low	["Imprecision"]
luma_200mg:vanza_teza_deuti_20mg_100mg_150mg	0	No concerns	Low risk	No concerns	Major concerns	No concerns	No concerns	Low	["Imprecision"]
luma_200mg:vanza_teza_deuti_5mg_100mg_150mg	0	No concerns	Low risk	No concerns	Major concerns	No concerns	No concerns	Low	["Imprecision"]
luma_200mg:vanza_teza_iva_5mg_100mg_150mg	0	Some concerns	Low risk	No concerns	Major concerns	No concerns	No concerns	Low	["Within-study bias", Imprecision"]
luma_25mg:luma_iva_200mg_150mg	0	Some concerns	Low risk	No concerns	Major concerns	No concerns	No concerns	Low	["Within-study bias", Imprecision"]
luma_25mg:luma_iva_200mg_250mg	0	Some concerns	Low risk	No concerns	Major concerns	No concerns	No concerns	Low	["Within-study bias", Imprecision"]
luma_25mg:luma_iva_400mg_250mg	0	Some concerns	Low risk	No concerns	Major concerns	No concerns	No concerns	Low	["Within-study bias", Imprecision"]
luma_25mg:luma_iva_600mg_250mg	0	Some concerns	Low risk	No concerns	Major concerns	No concerns	No concerns	Low	["Within-study bias", Imprecision"]
luma_25mg:vanza_teza_deuti_10mg_100mg_150mg	0	No concerns	Low risk	No concerns	Major concerns	No concerns	No concerns	Low	["Imprecision"]
luma_25mg:vanza_teza_deuti_20mg_100mg_150mg	0	No concerns	Low risk	No concerns	Major concerns	No concerns	No concerns	Low	["Imprecision"]
luma_25mg:vanza_teza_deuti_5mg_100mg_150mg	0	No concerns	Low risk	No concerns	Major concerns	No concerns	No concerns	Low	["Imprecision"]
luma_25mg:vanza_teza_iva_5mg_100mg_150mg	0	Some concerns	Low risk	No concerns	Major concerns	No concerns	No concerns	Low	["Within-study bias", Imprecision"]
luma_50mg:luma_iva_200mg_150mg	0	Some concerns	Low risk	No concerns	Major concerns	No concerns	No concerns	Low	["Within-study bias", Imprecision"]

luma_50mg:luma_iva_200mg_250mg	0	Some concerns	Low risk	No concerns	Major concerns	No concerns	No concerns	Low	["Within-study bias", Imprecision"]
luma_50mg:luma_iva_400mg_250mg	0	Some concerns	Low risk	No concerns	Major concerns	No concerns	No concerns	Low	["Within-study bias", Imprecision"]
luma_50mg:luma_iva_600mg_250mg	0	Some concerns	Low risk	No concerns	Major concerns	No concerns	No concerns	Low	["Within-study bias", Imprecision"]
luma_50mg:vanza_teza_deuti_10mg_100mg_150mg	0	No concerns	Low risk	No concerns	Major concerns	No concerns	No concerns	Low	["Imprecision"]
luma_50mg:vanza_teza_deuti_20mg_100mg_150mg	0	No concerns	Low risk	No concerns	Major concerns	No concerns	No concerns	Low	["Imprecision"]
luma_50mg:vanza_teza_deuti_5mg_100mg_150mg	0	No concerns	Low risk	No concerns	Major concerns	No concerns	No concerns	Low	["Imprecision"]
luma_50mg:vanza_teza_iva_5mg_100mg_150mg	0	Some concerns	Low risk	No concerns	Major concerns	No concerns	No concerns	Low	["Within-study bias", Imprecision"]
luma_iva_200mg_150mg:luma_iva_400mg_250mg	0	No concerns	Low risk	No concerns	Major concerns	No concerns	No concerns	Low	["Imprecision"]
luma_iva_200mg_150mg:luma_iva_600mg_250mg	0	No concerns	Low risk	No concerns	Major concerns	No concerns	No concerns	Low	["Imprecision"]
luma_iva_200mg_150mg:vanza_teza_deuti_10mg_100mg_150mg	0	No concerns	Low risk	No concerns	No concerns	No concerns	No concerns	High	NA
luma_iva_200mg_150mg:vanza_teza_deuti_20mg_100mg_150mg	0	No concerns	Low risk	No concerns	No concerns	Major concerns	No concerns	Low	["Heterogeneity"]
luma_iva_200mg_150mg:vanza_teza_deuti_5mg_100mg_150mg	0	No concerns	Low risk	No concerns	Major concerns	No concerns	No concerns	Low	["Imprecision"]
luma_iva_200mg_150mg:vanza_teza_iva_5mg_100mg_150mg	0	No concerns	Low risk	No concerns	Major concerns	No concerns	No concerns	Low	["Imprecision"]
luma_iva_200mg_250mg:vanza_teza_deuti_10mg_100mg_150mg	0	No concerns	Low risk	No concerns	No concerns	No concerns	No concerns	High	NA
luma_iva_200mg_250mg:vanza_teza_deuti_20mg_100mg_150mg	0	No concerns	Low risk	No concerns	No concerns	No concerns	No concerns	High	NA

luma_iva_200mg_250mg:vanza_teza_deuti_5mg_100mg_150mg	0	No concerns	Low risk	No concerns	Major concerns	No concerns	No concerns	Low	["Imprecision"]
luma_iva_200mg_250mg:vanza_teza_iva_5mg_100mg_150mg	0	No concerns	Low risk	No concerns	Major concerns	No concerns	No concerns	Low	["Imprecision"]
luma_iva_400mg_250mg:vanza_teza_deuti_10mg_100mg_150mg	0	No concerns	Low risk	No concerns	No concerns	No concerns	No concerns	High	NA
luma_iva_400mg_250mg:vanza_teza_deuti_20mg_100mg_150mg	0	No concerns	Low risk	No concerns	No concerns	No concerns	No concerns	High	NA
luma_iva_400mg_250mg:vanza_teza_deuti_5mg_100mg_150mg	0	No concerns	Low risk	No concerns	Major concerns	No concerns	No concerns	Low	["Imprecision"]
luma_iva_400mg_250mg:vanza_teza_iva_5mg_100mg_150mg	0	No concerns	Low risk	No concerns	Major concerns	No concerns	No concerns	Low	["Imprecision"]
luma_iva_600mg_250mg:vanza_teza_deuti_10mg_100mg_150mg	0	No concerns	Low risk	No concerns	No concerns	Major concerns	No concerns	Low	["Heterogeneity"]
luma_iva_600mg_250mg:vanza_teza_deuti_20mg_100mg_150mg	0	No concerns	Low risk	No concerns	No concerns	Major concerns	No concerns	Low	["Heterogeneity"]
luma_iva_600mg_250mg:vanza_teza_deuti_5mg_100mg_150mg	0	No concerns	Low risk	No concerns	Major concerns	No concerns	No concerns	Low	["Imprecision"]
luma_iva_600mg_250mg:vanza_teza_iva_5mg_100mg_150mg	0	No concerns	Low risk	No concerns	Major concerns	No concerns	No concerns	Low	["Imprecision"]
vanza_teza_deuti_10mg_100mg_150mg:vanza_teza_iva_5mg_100mg_150mg	0	No concerns	Low risk	No concerns	Major concerns	No concerns	No concerns	Low	["Imprecision"]
vanza_teza_deuti_20mg_100mg_150mg:vanza_teza_iva_5mg_100mg_150mg	0	No concerns	Low risk	No concerns	Major concerns	No concerns	No concerns	Low	["Imprecision"]
vanza_teza_deuti_5mg_100mg_150mg:vanza_teza_iva_5mg_100mg_150mg	0	No concerns	Low risk	No concerns	Major concerns	No concerns	No concerns	Low	["Imprecision"]

eTable 8 Certainty ratings of ppFEV$_1$ for adults treated for greater than 8 weeks using CINEMA framework

Comparison	Number of studies	Within-study bias	Reporting bias	Indirectness	Imprecision	Heterogeneity	Incoherence	Confidence rating	Reason(s) for downgrading
Mixed evidence									
Elexa_teza_iva_200mg_100mg_150mg:Placebo	1	No concerns	Low risk	No concerns	No concerns	No concerns	No concerns	High	NA
Elexa_teza_iva_200mg_100mg_150mg:Teza_iva_100mg_150mg	1	No concerns	Low risk	No concerns	No concerns	No concerns	No concerns	High	NA
Elexa_teza_iva_200mg_100mg_150mg:Vanza_teza_deuti_20mg_100mg_250mg	4	No concerns	Low risk	No concerns	Major concerns	No concerns	No concerns	Low	["Imprecision"]
Iva_150mg:Placebo	1	No concerns	Low risk	No concerns	Major concerns	No concerns	No concerns	Low	["Imprecision"]
Placebo:Teza_iva_100mg_150mg	3	No concerns	Low risk	No concerns	No concerns	No concerns	No concerns	High	NA
Placebo:Teza_iva_50mg_150mg	1	Some concerns	Low risk	No concerns	Major concerns	No concerns	No concerns	Low	["Within-study bias","Imprecision"]
luma_iva_400mg_250mg:Placebo	2	No concerns	Low risk	No concerns	No concerns	No concerns	No concerns	High	NA
luma_iva_600mg_250mg:Placebo	2	No concerns	Low risk	No concerns	No concerns	No concerns	No concerns	High	NA
luma_iva_400mg_250mg:luma_iva_600mg_250mg	3	No concerns	Low risk	No concerns	Major concerns	No concerns	No concerns	Low	["Imprecision"]
Indirect evidence									
Elexa_teza_iva_200mg_100mg_150mg:Iva_150mg	0	No concerns	Low risk	No concerns	No concerns	No concerns	No concerns	High	NA
Elexa_teza_iva_200mg_100mg_150mg:Teza_iva_50mg_150mg	0	No concerns	Low risk	No concerns	No concerns	No concerns	No concerns	High	NA
Elexa_teza_iva_200mg_100mg_150mg:luma_iva_400mg_250mg	0	No concerns	Low risk	No concerns	No concerns	No concerns	No concerns	High	NA
Elexa_teza_iva_200mg_100mg_150mg:luma_iva_600mg_250mg	0	No concerns	Low risk	No concerns	No concerns	No concerns	No concerns	High	NA
Iva_150mg:Teza_iva_100mg_150mg	0	No concerns	Low risk	No concerns	Major concerns	No concerns	No concerns	Low	["Imprecision"]

Iva_150mg:Teza_iva_50mg_150mg	0	Some concerns	Low risk	No concerns	Major concerns	No concerns	No concerns	Low	["Within-study bias","Imprecision"]
Iva_150mg:luma_iva_400mg_250mg	0	No concerns	Low risk	No concerns	Major concerns	No concerns	No concerns	Low	["Imprecision"]
Iva_150mg:luma_iva_600mg_250mg	0	No concerns	Low risk	No concerns	Major concerns	No concerns	No concerns	Low	["Imprecision"]
Iva_150mg:vanza_teza_deuti_20mg_100mg_250mg	0	No concerns	Low risk	No concerns	No concerns	No concerns	No concerns	High	NA
Placebo:vanza_teza_deuti_20mg_100mg_250mg	0	No concerns	Low risk	No concerns	No concerns	No concerns	No concerns	High	NA
Teza_iva_100mg_150mg:Teza_iva_50mg_150mg	0	Some concerns	Low risk	No concerns	Major concerns	No concerns	No concerns	Low	["Within-study bias","Imprecision"]
luma_iva_400mg_250mg:Teza_iva_100mg_150mg	0	No concerns	Low risk	No concerns	Major concerns	No concerns	No concerns	Low	["Imprecision"]
luma_iva_600mg_250mg:Teza_iva_100mg_150mg	0	No concerns	Low risk	No concerns	Major concerns	No concerns	No concerns	Low	["Imprecision"]
Teza_iva_100mg_150mg:vanza_teza_deuti_20mg_100mg_250mg	0	No concerns	Low risk	No concerns	No concerns	No concerns	No concerns	High	NA
luma_iva_400mg_250mg:Teza_iva_50mg_150mg	0	No concerns	Low risk	No concerns	Major concerns	No concerns	No concerns	Low	["Imprecision"]
luma_iva_600mg_250mg:Teza_iva_50mg_150mg	0	No concerns	Low risk	No concerns	Major concerns	No concerns	No concerns	Low	["Imprecision"]
Teza_iva_50mg_150mg:vanza_teza_deuti_20mg_100mg_250mg	0	No concerns	Low risk	No concerns	No concerns	No concerns	No concerns	High	NA
luma_iva_400mg_250mg:vanza_teza_deuti_20mg_100mg_250mg	0	No concerns	Low risk	No concerns	No concerns	No concerns	No concerns	High	NA
luma_iva_600mg_250mg:vanza_teza_deuti_20mg_100mg_250mg	0	No concerns	Low risk	No concerns	No concerns	No concerns	No concerns	High	NA

eTable 9 Certainty ratings of sweat chloride for adults treated for 4 to 8 weeks using CINEMA framework

Comparison	Number of studies	Within-study bias	Reporting bias	Indirectness	Imprecision	Heterogeneity	Incoherence	Confidence rating	Reason(s) for downgrading
**Mixed evidence**									
Elexa_teza_iva_100mg_100mg_150mg:Elexa_teza_iva_200mg_100mg_150mg	1	No concerns	Low risk	No concerns	Major concerns	No concerns	No concerns	Low	["Imprecision"]
Elexa_teza_iva_100mg_100mg_150mg:Elexa_teza_iva_50mg_100mg_150mg	1	No concerns	Low risk	No concerns	Major concerns	No concerns	No concerns	Low	["Imprecision"]
Elexa_teza_iva_100mg_100mg_150mg:Placebo	1	No concerns	Low risk	No concerns	No concerns	No concerns	No concerns	High	NA
Elexa_teza_iva_200mg_100mg_150mg:Elexa_teza_iva_50mg_100mg_150mg	1	No concerns	Low risk	No concerns	Major concerns	No concerns	No concerns	Low	["Imprecision"]
Elexa_teza_iva_200mg_100mg_150mg:Iva_150mg	1	No concerns	Low risk	No concerns	No concerns	No concerns	No concerns	High	NA
Elexa_teza_iva_200mg_100mg_150mg:Placebo	2	No concerns	Low risk	No concerns	No concerns	No concerns	No concerns	High	NA
Elexa_teza_iva_200mg_100mg_150mg:Teza_iva_100mg_150mg	3	No concerns	Low risk	No concerns	No concerns	No concerns	No concerns	High	NA
Elexa_teza_iva_50mg_100mg_150mg:Placebo	1	No concerns	Low risk	No concerns	No concerns	No concerns	No concerns	High	NA
GLPG2222_100mg:GLPG2222_200mg	1	No concerns	Low risk	No concerns	Major concerns	No concerns	No concerns	Low	["Imprecision"]
GLPG2222_100mg:GLPG2222_400mg	1	No concerns	Low risk	No concerns	Major concerns	No concerns	No concerns	Low	["Imprecision"]
GLPG2222_100mg:GLPG2222_50mg	1	No concerns	Low risk	No concerns	Major concerns	No concerns	No concerns	Low	["Imprecision"]
GLPG2222_100mg:Placebo	1	No concerns	Low risk	No concerns	Major concerns	No concerns	No concerns	Low	["Imprecision"]
GLPG2222_150mg:GLPG2222_300mg	1	No concerns	Low risk	No concerns	Major concerns	No concerns	No concerns	Low	["Imprecision"]

GLPG2222_150mg:Placebo	1	No concerns	Low risk	No concerns	Major concerns	No concerns	No concerns	Low	["Imprecision"]
GLPG2222_200mg:GLPG2222_400mg	1	No concerns	Low risk	No concerns	Major concerns	No concerns	No concerns	Low	["Imprecision"]
GLPG2222_200mg:GLPG2222_50mg	1	No concerns	Low risk	No concerns	Major concerns	No concerns	No concerns	Low	["Imprecision"]
GLPG2222_200mg:Placebo	1	No concerns	Low risk	No concerns	No concerns	Major concerns	No concerns	Low	["Heterogeneity"]
GLPG2222_300mg:Placebo	1	No concerns	Low risk	No concerns	Major concerns	No concerns	No concerns	Low	["Imprecision"]
GLPG2222_400mg:GLPG2222_50mg	1	No concerns	Low risk	No concerns	Major concerns	No concerns	No concerns	Low	["Imprecision"]
GLPG2222_400mg:Placebo	1	No concerns	Low risk	No concerns	Major concerns	No concerns	No concerns	Low	["Imprecision"]
GLPG2222_50mg:Placebo	1	No concerns	Low risk	No concerns	Major concerns	No concerns	No concerns	Low	["Imprecision"]
GLPG2737_75mg:Placebo	1	No concerns	Low risk	No concerns	Major concerns	No concerns	No concerns	Low	["Imprecision"]
Iva_150mg:Teza_iva_100mg_150mg	1	No concerns	Low risk	No concerns	Major concerns	No concerns	Major concerns	Very Low	["Imprecision"|" Incoherence"]
Ola_teza_iva_600mg_50mg_300mg:Placebo	1	No concerns	Low risk	No concerns	No concerns	No concerns	No concerns	High	NA
Ola_teza_iva_600mg_50mg_300mg:Teza_iva_50mg_300mg	1	No concerns	Low risk	No concerns	No concerns	No concerns	No concerns	High	NA
Placebo:Teza_iva_100mg_150mg	2	No concerns	Low risk	No concerns	Major concerns	No concerns	No concerns	Low	["Imprecision"]
Placebo:Teza_iva_100mg_50mg	1	Some concerns	Low risk	No concerns	Major concerns	No concerns	No concerns	Low	["Within-study bias"|"Imprecision"]
Placebo:Teza_iva_50mg_150mg	1	Some concerns	Low risk	No concerns	Major concerns	No concerns	No concerns	Low	["Within-study bias"|"Imprecision"]
Placebo:VX152_teza_iva_100mg_100mg_150mg	1	No concerns	Low risk	No concerns	Major concerns	No concerns	Major concerns	Very Low	["Imprecision"|" Incoherence"]

Placebo:VX152_teza_iva_200mg_100mg_150mg	1	No concerns	Low risk	No concerns	Major concerns	No concerns	Major concerns	Very Low	["Imprecision"|" Incoherence"]
Placebo:VX152_teza_iva_300mg_100mg_150mg	1	No concerns	Low risk	No concerns	No concerns	No concerns	Major concerns	Low	["Incoherence"]
luma_100mg:Placebo	1	Some concerns	Low risk	No concerns	Major concerns	No concerns	No concerns	Low	["Within-study bias"|"Imprecision"]
luma_200mg:Placebo	1	Some concerns	Low risk	No concerns	Major concerns	No concerns	No concerns	Low	["Within-study bias"|"Imprecision"]
luma_25mg:Placebo	1	Some concerns	Low risk	No concerns	Major concerns	No concerns	No concerns	Low	["Within-study bias"|"Imprecision"]
luma_50mg:Placebo	1	Some concerns	Low risk	No concerns	Major concerns	No concerns	No concerns	Low	["Within-study bias"|"Imprecision"]
luma_iva_200mg_150mg:Placebo	1	No concerns	Low risk	No concerns	Major concerns	No concerns	No concerns	Low	["Imprecision"]
luma_iva_200mg_250mg:Placebo	2	No concerns	Low risk	No concerns	Major concerns	No concerns	No concerns	Low	["Imprecision"]
luma_iva_400mg_250mg:Placebo	2	No concerns	Low risk	No concerns	No concerns	Major concerns	No concerns	Low	["Heterogeneity"]
luma_iva_600mg_250mg:Placebo	1	No concerns	Low risk	No concerns	Major concerns	No concerns	No concerns	Low	["Imprecision"]
Placebo:vanza_teza_deuti_10mg_100mg_150mg	1	No concerns	Low risk	No concerns	No concerns	No concerns	No concerns	High	NA
Placebo:vanza_teza_deuti_20mg_100mg_150mg	1	No concerns	Low risk	No concerns	No concerns	No concerns	No concerns	High	NA
Placebo:vanza_teza_deuti_5mg_100mg_150mg	1	No concerns	Low risk	No concerns	No concerns	No concerns	No concerns	High	NA
Placebo:vanza_teza_iva_5mg_100mg_150mg	1	No concerns	Low risk	No concerns	No concerns	No concerns	No concerns	High	NA
Teza_iva_100mg_150mg:VX152_teza_iva_200mg_100mg_150 mg	1	No concerns	Low risk	No concerns	Major concerns	No concerns	Major concerns	Very Low	["Imprecision"|" Incoherence"]
Teza_iva_100mg_150mg:VX152_teza_iva_300mg_100mg_150 mg	1	No concerns	Low risk	No concerns	No concerns	Major concerns	No concerns	Low	["Heterogeneity"]
Teza_iva_100mg_150mg:vanza_teza_deuti_20mg_100mg_150m g	1	No concerns	Low risk	No concerns	No concerns	No concerns	No concerns	High	NA

Teza_iva_100mg_50mg:Teza_iva_50mg_150mg	1	Some concerns	Low risk	No concerns	Major concerns	No concerns	No concerns	Low	["Within-study bias"|"Imprecision"]
VX152_teza_iva_100mg_100mg_150mg:VX152_teza_iva_200mg_100mg_150mg	1	No concerns	Low risk	No concerns	Major concerns	No concerns	Major concerns	Very Low	["Imprecision"|"Incoherence"]
VX152_teza_iva_100mg_100mg_150mg:VX152_teza_iva_300mg_100mg_150mg	1	No concerns	Low risk	No concerns	Major concerns	No concerns	No concerns	Low	["Imprecision"]
VX152_teza_iva_200mg_100mg_150mg:VX152_teza_iva_300mg_100mg_150mg	1	No concerns	Low risk	No concerns	Major concerns	No concerns	No concerns	Low	["Imprecision"]
luma_100mg:luma_200mg	1	Some concerns	Low risk	No concerns	Major concerns	No concerns	No concerns	Low	["Within-study bias"|"Imprecision"]
luma_100mg:luma_25mg	1	Some concerns	Low risk	No concerns	Major concerns	No concerns	No concerns	Low	["Within-study bias"|"Imprecision"]
luma_100mg:luma_50mg	1	Some concerns	Low risk	No concerns	Major concerns	No concerns	No concerns	Low	["Within-study bias"|"Imprecision"]
luma_200mg:luma_25mg	1	Some concerns	Low risk	No concerns	Major concerns	No concerns	No concerns	Low	["Within-study bias"|"Imprecision"]
luma_200mg:luma_50mg	1	Some concerns	Low risk	No concerns	Major concerns	No concerns	No concerns	Low	["Within-study bias"|"Imprecision"]
luma_25mg:luma_50mg	1	Some concerns	Low risk	No concerns	Major concerns	No concerns	No concerns	Low	["Within-study bias"|"Imprecision"]
luma_iva_200mg_150mg:luma_iva_200mg_250mg	1	No concerns	Low risk	No concerns	Major concerns	No concerns	No concerns	Low	["Imprecision"]
luma_iva_200mg_250mg:luma_iva_400mg_250mg	1	No concerns	Low risk	No concerns	Major concerns	No concerns	No concerns	Low	["Imprecision"]
luma_iva_200mg_250mg:luma_iva_600mg_250mg	1	No concerns	Low risk	No concerns	Major concerns	No concerns	No concerns	Low	["Imprecision"]
luma_iva_400mg_250mg:luma_iva_600mg_250mg	1	No concerns	Low risk	No concerns	Major concerns	No concerns	No concerns	Low	["Imprecision"]
vanza_teza_deuti_10mg_100mg_150mg:vanza_teza_deuti_20mg_100mg_150mg	1	No concerns	Low risk	No concerns	Major concerns	No concerns	No concerns	Low	["Imprecision"]
vanza_teza_deuti_10mg_100mg_150mg:vanza_teza_deuti_5mg_100mg_150mg	1	No concerns	Low risk	No concerns	Major concerns	No concerns	No concerns	Low	["Imprecision"]
vanza_teza_deuti_20mg_100mg_150mg:vanza_teza_deuti_5mg_100mg_150mg	1	No concerns	Low risk	No concerns	Major concerns	No concerns	No concerns	Low	["Imprecision"]

Indirect evidence									
Elexa_teza_iva_100mg_100mg_150mg:GLPG2222_100mg	0	No concerns	Low risk	No concerns	No concerns	No concerns	No concerns	High	NA
Elexa_teza_iva_100mg_100mg_150mg:GLPG2222_150mg	0	No concerns	Low risk	No concerns	No concerns	Major concerns	No concerns	Low	["Heterogeneity"]
Elexa_teza_iva_100mg_100mg_150mg:GLPG2222_200mg	0	No concerns	Low risk	No concerns	Major concerns	No concerns	No concerns	Low	["Imprecision"]
Elexa_teza_iva_100mg_100mg_150mg:GLPG2222_300mg	0	No concerns	Low risk	No concerns	Major concerns	No concerns	No concerns	Low	["Imprecision"]
Elexa_teza_iva_100mg_100mg_150mg:GLPG2222_400mg	0	No concerns	Low risk	No concerns	No concerns	Major concerns	No concerns	Low	["Heterogeneity"]
Elexa_teza_iva_100mg_100mg_150mg:GLPG2222_50mg	0	No concerns	Low risk	No concerns	No concerns	No concerns	No concerns	High	NA
Elexa_teza_iva_100mg_100mg_150mg:GLPG2737_75mg	0	No concerns	Low risk	No concerns	Major concerns	No concerns	No concerns	Low	["Imprecision"]
Elexa_teza_iva_100mg_100mg_150mg:Iva_150mg	0	No concerns	Low risk	No concerns	No concerns	No concerns	No concerns	High	NA
Elexa_teza_iva_100mg_100mg_150mg:Ola_teza_iva_600mg_50 mg_300mg	0	No concerns	Low risk	No concerns	Major concerns	No concerns	No concerns	Low	["Imprecision"]
Elexa_teza_iva_100mg_100mg_150mg:Teza_iva_100mg_150mg	0	No concerns	Low risk	No concerns	No concerns	No concerns	No concerns	High	NA
Elexa_teza_iva_100mg_100mg_150mg:Teza_iva_100mg_50mg	0	No concerns	Low risk	No concerns	No concerns	No concerns	No concerns	High	NA
Elexa_teza_iva_100mg_100mg_150mg:Teza_iva_50mg_150mg	0	No concerns	Low risk	No concerns	No concerns	No concerns	No concerns	High	NA
Elexa_teza_iva_100mg_100mg_150mg:Teza_iva_50mg_300mg	0	No concerns	Low risk	No concerns	No concerns	Major concerns	No concerns	Low	["Heterogeneity"]
Elexa_teza_iva_100mg_100mg_150mg:VX152_teza_iva_100mg _100mg_150mg	0	No concerns	Low risk	No concerns	No concerns	Major concerns	No concerns	Low	["Heterogeneity"]
Elexa_teza_iva_100mg_100mg_150mg:VX152_teza_iva_200mg _100mg_150mg	0	No concerns	Low risk	No concerns	No concerns	Major concerns	No concerns	Low	["Heterogeneity"]
Elexa_teza_iva_100mg_100mg_150mg:VX152_teza_iva_300mg _100mg_150mg	0	No concerns	Low risk	No concerns	Major concerns	No concerns	No concerns	Low	["Imprecision"]

Elexa_teza_iva_100mg_100mg_150mg:luma_100mg	0	No concerns	Low risk	No concerns	No concerns	No concerns	No concerns	High	NA
Elexa_teza_iva_100mg_100mg_150mg:luma_200mg	0	No concerns	Low risk	No concerns	No concerns	No concerns	No concerns	High	NA
Elexa_teza_iva_100mg_100mg_150mg:luma_25mg	0	No concerns	Low risk	No concerns	No concerns	No concerns	No concerns	High	NA
Elexa_teza_iva_100mg_100mg_150mg:luma_50mg	0	No concerns	Low risk	No concerns	No concerns	No concerns	No concerns	High	NA
Elexa_teza_iva_100mg_100mg_150mg:luma_iva_200mg_150mg	0	No concerns	Low risk	No concerns	No concerns	No concerns	No concerns	High	NA
Elexa_teza_iva_100mg_100mg_150mg:luma_iva_200mg_250mg	0	No concerns	Low risk	No concerns	No concerns	No concerns	No concerns	High	NA
Elexa_teza_iva_100mg_100mg_150mg:luma_iva_400mg_250mg	0	No concerns	Low risk	No concerns	No concerns	Major concerns	No concerns	Low	["Heterogeneity"]
Elexa_teza_iva_100mg_100mg_150mg:luma_iva_600mg_250mg	0	No concerns	Low risk	No concerns	No concerns	Major concerns	No concerns	Low	["Heterogeneity"]
Elexa_teza_iva_100mg_100mg_150mg:vanza_teza_deuti_10mg_100mg_150mg	0	No concerns	Low risk	No concerns	Major concerns	No concerns	No concerns	Low	["Imprecision"]
Elexa_teza_iva_100mg_100mg_150mg:vanza_teza_deuti_20mg_100mg_150mg	0	No concerns	Low risk	No concerns	No concerns	Major concerns	No concerns	Low	["Heterogeneity"]
Elexa_teza_iva_100mg_100mg_150mg:vanza_teza_deuti_5mg_100mg_150mg	0	No concerns	Low risk	No concerns	Major concerns	No concerns	No concerns	Low	["Imprecision"]
Elexa_teza_iva_100mg_100mg_150mg:vanza_teza_iva_5mg_100mg_150mg	0	No concerns	Low risk	No concerns	Major concerns	No concerns	No concerns	Low	["Imprecision"]
Elexa_teza_iva_200mg_100mg_150mg:GLPG2222_100mg	0	No concerns	Low risk	No concerns	No concerns	No concerns	No concerns	High	NA
Elexa_teza_iva_200mg_100mg_150mg:GLPG2222_150mg	0	No concerns	Low risk	No concerns	No concerns	No concerns	No concerns	High	NA
Elexa_teza_iva_200mg_100mg_150mg:GLPG2222_200mg	0	No concerns	Low risk	No concerns	No concerns	No concerns	No concerns	High	NA
Elexa_teza_iva_200mg_100mg_150mg:GLPG2222_300mg	0	No concerns	Low risk	No concerns	No concerns	Major concerns	No concerns	Low	["Heterogeneity"]
Elexa_teza_iva_200mg_100mg_150mg:GLPG2222_400mg	0	No concerns	Low risk	No concerns	No concerns	No concerns	No concerns	High	NA

Elexa_teza_iva_200mg_100mg_150mg:GLPG2222_50mg	0	No concerns	Low risk	No concerns	No concerns	No concerns	No concerns	High	NA
Elexa_teza_iva_200mg_100mg_150mg:GLPG2737_75mg	0	No concerns	Low risk	No concerns	No concerns	Major concerns	No concerns	Low	["Heterogeneity"]
Elexa_teza_iva_200mg_100mg_150mg:Ola_teza_iva_600mg_50mg_300mg	0	No concerns	Low risk	No concerns	Major concerns	No concerns	No concerns	Low	["Imprecision"]
Elexa_teza_iva_200mg_100mg_150mg:Teza_iva_100mg_50mg	0	No concerns	Low risk	No concerns	No concerns	No concerns	No concerns	High	NA
Elexa_teza_iva_200mg_100mg_150mg:Teza_iva_50mg_150mg	0	No concerns	Low risk	No concerns	No concerns	No concerns	No concerns	High	NA
Elexa_teza_iva_200mg_100mg_150mg:Teza_iva_50mg_300mg	0	No concerns	Low risk	No concerns	No concerns	No concerns	No concerns	High	NA
Elexa_teza_iva_200mg_100mg_150mg:VX152_teza_iva_100mg_100mg_150mg	0	No concerns	Low risk	No concerns	No concerns	No concerns	No concerns	High	NA
Elexa_teza_iva_200mg_100mg_150mg:VX152_teza_iva_200mg_100mg_150mg	0	No concerns	Low risk	No concerns	No concerns	No concerns	No concerns	High	NA
Elexa_teza_iva_200mg_100mg_150mg:VX152_teza_iva_300mg_100mg_150mg	0	No concerns	Low risk	No concerns	No concerns	Major concerns	No concerns	Low	["Heterogeneity"]
bamoca_teza_iva_240mg_100mg_150mg:Elexa_teza_iva_200mg_100mg_150mg	0	No concerns	Low risk	No concerns	Major concerns	No concerns	No concerns	Low	["Imprecision"]
bamoca_teza_iva_400mg_100mg_150mg:Elexa_teza_iva_200mg_100mg_150mg	0	No concerns	Low risk	No concerns	No concerns	Major concerns	No concerns	Low	["Heterogeneity"]
bamoca_teza_iva_80mg_100mg_150mg:Elexa_teza_iva_200mg_100mg_150mg	0	No concerns	Low risk	No concerns	Major concerns	No concerns	No concerns	Low	["Imprecision"]
Elexa_teza_iva_200mg_100mg_150mg:luma_100mg	0	Some concerns	Low risk	No concerns	No concerns	No concerns	No concerns	Moderate	["Within-study bias"]
Elexa_teza_iva_200mg_100mg_150mg:luma_200mg	0	Some concerns	Low risk	No concerns	No concerns	No concerns	No concerns	Moderate	["Within-study bias"]
Elexa_teza_iva_200mg_100mg_150mg:luma_25mg	0	Some concerns	Low risk	No concerns	No concerns	No concerns	No concerns	Moderate	["Within-study bias"]
Elexa_teza_iva_200mg_100mg_150mg:luma_50mg	0	Some concerns	Low risk	No concerns	No concerns	No concerns	No concerns	Moderate	["Within-study bias"]
Elexa_teza_iva_200mg_100mg_150mg:luma_iva_200mg_150mg	0	No concerns	Low risk	No concerns	No concerns	No concerns	No concerns	High	NA

Elexa_teza_iva_200mg_100mg_150mg:luma_iva_200mg_250mg	0	No concerns	Low risk	No concerns	No concerns	No concerns	No concerns	High	NA
Elexa_teza_iva_200mg_100mg_150mg:luma_iva_400mg_250mg	0	No concerns	Low risk	No concerns	No concerns	No concerns	No concerns	High	NA
Elexa_teza_iva_200mg_100mg_150mg:luma_iva_600mg_250mg	0	No concerns	Low risk	No concerns	No concerns	No concerns	No concerns	High	NA
Elexa_teza_iva_200mg_100mg_150mg:vanza_teza_deuti_10mg_100mg_150mg	0	No concerns	Low risk	No concerns	Major concerns	No concerns	No concerns	Low	["Imprecision"]
Elexa_teza_iva_200mg_100mg_150mg:vanza_teza_deuti_20mg_100mg_150mg	0	No concerns	Low risk	No concerns	Major concerns	No concerns	No concerns	Low	["Imprecision"]
Elexa_teza_iva_200mg_100mg_150mg:vanza_teza_deuti_5mg_100mg_150mg	0	No concerns	Low risk	No concerns	Major concerns	No concerns	No concerns	Low	["Imprecision"]
Elexa_teza_iva_200mg_100mg_150mg:vanza_teza_iva_5mg_100mg_150mg	0	No concerns	Low risk	No concerns	Major concerns	No concerns	No concerns	Low	["Imprecision"]
Elexa_teza_iva_50mg_100mg_150mg:GLPG2222_100mg	0	No concerns	Low risk	No concerns	No concerns	No concerns	No concerns	High	NA
Elexa_teza_iva_50mg_100mg_150mg:GLPG2222_150mg	0	No concerns	Low risk	No concerns	No concerns	Major concerns	No concerns	Low	["Heterogeneity"]
Elexa_teza_iva_50mg_100mg_150mg:GLPG2222_200mg	0	No concerns	Low risk	No concerns	No concerns	Major concerns	No concerns	Low	["Heterogeneity"]
Elexa_teza_iva_50mg_100mg_150mg:GLPG2222_300mg	0	No concerns	Low risk	No concerns	No concerns	Major concerns	No concerns	Low	["Heterogeneity"]
Elexa_teza_iva_50mg_100mg_150mg:GLPG2222_400mg	0	No concerns	Low risk	No concerns	No concerns	No concerns	No concerns	High	NA
Elexa_teza_iva_50mg_100mg_150mg:GLPG2222_50mg	0	No concerns	Low risk	No concerns	No concerns	No concerns	No concerns	High	NA
Elexa_teza_iva_50mg_100mg_150mg:GLPG2737_75mg	0	No concerns	Low risk	No concerns	No concerns	Major concerns	No concerns	Low	["Heterogeneity"]
Elexa_teza_iva_50mg_100mg_150mg:Iva_150mg	0	No concerns	Low risk	No concerns	No concerns	No concerns	No concerns	High	NA
Elexa_teza_iva_50mg_100mg_150mg:Ola_teza_iva_600mg_50mg_300mg	0	No concerns	Low risk	No concerns	Major concerns	No concerns	No concerns	Low	["Imprecision"]
Elexa_teza_iva_50mg_100mg_150mg:Teza_iva_100mg_150mg	0	No concerns	Low risk	No concerns	No concerns	No concerns	No concerns	High	NA

Elexa_teza_iva_50mg_100mg_150mg:Teza_iva_100mg_50mg	0	No concerns	Low risk	No concerns	No concerns	No concerns	No concerns	High	NA
Elexa_teza_iva_50mg_100mg_150mg:Teza_iva_50mg_150mg	0	No concerns	Low risk	No concerns	No concerns	No concerns	No concerns	High	NA
Elexa_teza_iva_50mg_100mg_150mg:Teza_iva_50mg_300mg	0	No concerns	Low risk	No concerns	No concerns	No concerns	No concerns	High	NA
Elexa_teza_iva_50mg_100mg_150mg:VX152_teza_iva_100mg_100mg_150mg	0	No concerns	Low risk	No concerns	No concerns	No concerns	No concerns	High	NA
Elexa_teza_iva_50mg_100mg_150mg:VX152_teza_iva_200mg_100mg_150mg	0	No concerns	Low risk	No concerns	No concerns	No concerns	No concerns	High	NA
Elexa_teza_iva_50mg_100mg_150mg:VX152_teza_iva_300mg_100mg_150mg	0	No concerns	Low risk	No concerns	Major concerns	No concerns	No concerns	Low	["Imprecision"]
Elexa_teza_iva_50mg_100mg_150mg:luma_100mg	0	No concerns	Low risk	No concerns	No concerns	No concerns	No concerns	High	NA
Elexa_teza_iva_50mg_100mg_150mg:luma_200mg	0	No concerns	Low risk	No concerns	No concerns	No concerns	No concerns	High	NA
Elexa_teza_iva_50mg_100mg_150mg:luma_25mg	0	No concerns	Low risk	No concerns	No concerns	No concerns	No concerns	High	NA
Elexa_teza_iva_50mg_100mg_150mg:luma_50mg	0	No concerns	Low risk	No concerns	No concerns	No concerns	No concerns	High	NA
Elexa_teza_iva_50mg_100mg_150mg:luma_iva_200mg_150mg	0	No concerns	Low risk	No concerns	No concerns	No concerns	No concerns	High	NA
Elexa_teza_iva_50mg_100mg_150mg:luma_iva_200mg_250mg	0	No concerns	Low risk	No concerns	No concerns	No concerns	No concerns	High	NA
Elexa_teza_iva_50mg_100mg_150mg:luma_iva_400mg_250mg	0	No concerns	Low risk	No concerns	No concerns	No concerns	No concerns	High	NA
Elexa_teza_iva_50mg_100mg_150mg:luma_iva_600mg_250mg	0	No concerns	Low risk	No concerns	No concerns	No concerns	No concerns	High	NA
Elexa_teza_iva_50mg_100mg_150mg:vanza_teza_deuti_10mg_100mg_150mg	0	No concerns	Low risk	No concerns	Major concerns	No concerns	No concerns	Low	["Imprecision"]
Elexa_teza_iva_50mg_100mg_150mg:vanza_teza_deuti_20mg_100mg_150mg	0	No concerns	Low risk	No concerns	Major concerns	No concerns	No concerns	Low	["Imprecision"]
Elexa_teza_iva_50mg_100mg_150mg:vanza_teza_deuti_5mg_100mg_150mg	0	No concerns	Low risk	No concerns	Major concerns	No concerns	No concerns	Low	["Imprecision"]

Elexa_teza_iva_50mg_100mg_150mg:vanza_teza_iva_5mg_100mg_150mg	0	No concerns	Low risk	No concerns	Major concerns	No concerns	No concerns	Low	["Imprecision"]
GLPG2222_100mg:GLPG2222_150mg	0	No concerns	Low risk	No concerns	Major concerns	No concerns	No concerns	Low	["Imprecision"]
GLPG2222_100mg:GLPG2222_300mg	0	No concerns	Low risk	No concerns	Major concerns	No concerns	No concerns	Low	["Imprecision"]
GLPG2222_100mg:GLPG2737_75mg	0	No concerns	Low risk	No concerns	Major concerns	No concerns	No concerns	Low	["Imprecision"]
GLPG2222_100mg:Iva_150mg	0	No concerns	Low risk	No concerns	Major concerns	No concerns	No concerns	High	["Imprecision"]
GLPG2222_100mg:Ola_teza_iva_600mg_50mg_300mg	0	No concerns	Low risk	No concerns	No concerns	No concerns	No concerns	High	NA
GLPG2222_100mg:Teza_iva_100mg_150mg	0	No concerns	Low risk	No concerns	Major concerns	No concerns	No concerns	Low	["Imprecision"]
GLPG2222_100mg:Teza_iva_100mg_50mg	0	No concerns	Low risk	No concerns	Major concerns	No concerns	No concerns	Low	["Imprecision"]
GLPG2222_100mg:Teza_iva_50mg_150mg	0	No concerns	Low risk	No concerns	Major concerns	No concerns	No concerns	Low	["Imprecision"]
GLPG2222_100mg:Teza_iva_50mg_300mg	0	No concerns	Low risk	No concerns	Major concerns	No concerns	No concerns	Low	["Imprecision"]
GLPG2222_100mg:VX152_teza_iva_100mg_100mg_150mg	0	No concerns	Low risk	No concerns	Major concerns	No concerns	No concerns	Low	["Imprecision"]
GLPG2222_100mg:VX152_teza_iva_200mg_100mg_150mg	0	No concerns	Low risk	No concerns	Major concerns	No concerns	No concerns	Low	["Imprecision"]
GLPG2222_100mg:VX152_teza_iva_300mg_100mg_150mg	0	No concerns	Low risk	No concerns	Major concerns	No concerns	No concerns	Low	["Imprecision"]
GLPG2222_100mg:luma_100mg	0	No concerns	Low risk	No concerns	Major concerns	No concerns	No concerns	Low	["Imprecision"]
GLPG2222_100mg:luma_200mg	0	Some concerns	Low risk	No concerns	Major concerns	No concerns	No concerns	Low	["Within-study bias"|"Imprecision"]
GLPG2222_100mg:luma_25mg	0	No concerns	Low risk	No concerns	Major concerns	No concerns	No concerns	Low	["Imprecision"]
GLPG2222_100mg:luma_50mg	0	No concerns	Low risk	No concerns	Major concerns	No concerns	No concerns	Low	["Imprecision"]

GLPG2222_100mg:luma_iva_200mg_150mg	0	No concerns	Low risk	No concerns	Major concerns	No concerns	No concerns	Low	["Imprecision"]
GLPG2222_100mg:luma_iva_200mg_250mg	0	No concerns	Low risk	No concerns	Major concerns	No concerns	No concerns	Low	["Imprecision"]
GLPG2222_100mg:luma_iva_400mg_250mg	0	No concerns	Low risk	No concerns	Major concerns	No concerns	No concerns	Low	["Imprecision"]
GLPG2222_100mg:luma_iva_600mg_250mg	0	No concerns	Low risk	No concerns	Major concerns	No concerns	No concerns	Low	["Imprecision"]
GLPG2222_100mg:vanza_teza_deuti_10mg_100mg_150mg	0	No concerns	Low risk	No concerns	No concerns	No concerns	No concerns	High	NA
GLPG2222_100mg:vanza_teza_deuti_20mg_100mg_150mg	0	No concerns	Low risk	No concerns	No concerns	No concerns	No concerns	High	NA
GLPG2222_100mg:vanza_teza_deuti_5mg_100mg_150mg	0	No concerns	Low risk	No concerns	No concerns	No concerns	No concerns	High	NA
GLPG2222_100mg:vanza_teza_iva_5mg_100mg_150mg	0	No concerns	Low risk	No concerns	No concerns	No concerns	No concerns	High	NA
GLPG2222_150mg:GLPG2222_200mg	0	No concerns	Low risk	No concerns	Major concerns	No concerns	No concerns	Low	["Imprecision"]
GLPG2222_150mg:GLPG2222_400mg	0	No concerns	Low risk	No concerns	Major concerns	No concerns	No concerns	Low	["Imprecision"]
GLPG2222_150mg:GLPG2222_50mg	0	No concerns	Low risk	No concerns	Major concerns	No concerns	No concerns	Low	["Imprecision"]
GLPG2222_150mg:GLPG2737_75mg	0	No concerns	Low risk	No concerns	Major concerns	No concerns	No concerns	Low	["Imprecision"]
GLPG2222_150mg:Iva_150mg	0	No concerns	Low risk	No concerns	Major concerns	No concerns	No concerns	Low	["Imprecision"]
GLPG2222_150mg:Ola_teza_iva_600mg_50mg_300mg	0	No concerns	Low risk	No concerns	No concerns	Major concerns	No concerns	Low	["Heterogeneity"]
GLPG2222_150mg:Teza_iva_100mg_150mg	0	No concerns	Low risk	No concerns	Major concerns	No concerns	No concerns	Low	["Imprecision"]
GLPG2222_150mg:Teza_iva_100mg_50mg	0	No concerns	Low risk	No concerns	Major concerns	No concerns	No concerns	Low	["Imprecision"]
GLPG2222_150mg:Teza_iva_50mg_150mg	0	No concerns	Low risk	No concerns	Major concerns	No concerns	No concerns	Low	["Imprecision"]

GLPG2222_150mg:Teza_iva_50mg_300mg	0	No concerns	Low risk	No concerns	Major concerns	No concerns	No concerns	Low	["Imprecision"]
GLPG2222_150mg:VX152_teza_iva_100mg_100mg_150mg	0	No concerns	Low risk	No concerns	Major concerns	No concerns	No concerns	Low	["Imprecision"]
GLPG2222_150mg:VX152_teza_iva_200mg_100mg_150mg	0	No concerns	Low risk	No concerns	Major concerns	No concerns	No concerns	Low	["Imprecision"]
GLPG2222_150mg:VX152_teza_iva_300mg_100mg_150mg	0	No concerns	Low risk	No concerns	Major concerns	No concerns	No concerns	Low	["Imprecision"]
GLPG2222_150mg:luma_100mg	0	Some concerns	Low risk	No concerns	Major concerns	No concerns	No concerns	Low	["Within-study bias"|"Imprecision"]
GLPG2222_150mg:luma_200mg	0	Some concerns	Low risk	No concerns	Major concerns	No concerns	No concerns	Low	["Within-study bias"|"Imprecision"]
GLPG2222_150mg:luma_25mg	0	Some concerns	Low risk	No concerns	Major concerns	No concerns	No concerns	Low	["Within-study bias"|"Imprecision"]
GLPG2222_150mg:luma_50mg	0	Some concerns	Low risk	No concerns	Major concerns	No concerns	No concerns	Low	["Within-study bias"|"Imprecision"]
GLPG2222_150mg:luma_iva_200mg_150mg	0	No concerns	Low risk	No concerns	Major concerns	No concerns	No concerns	Low	["Imprecision"]
GLPG2222_150mg:luma_iva_200mg_250mg	0	No concerns	Low risk	No concerns	Major concerns	No concerns	No concerns	Low	["Imprecision"]
GLPG2222_150mg:luma_iva_400mg_250mg	0	No concerns	Low risk	No concerns	Major concerns	No concerns	No concerns	Low	["Imprecision"]
GLPG2222_150mg:luma_iva_600mg_250mg	0	No concerns	Low risk	No concerns	Major concerns	No concerns	No concerns	Low	["Imprecision"]
GLPG2222_150mg:vanza_teza_deuti_10mg_100mg_150mg	0	No concerns	Low risk	No concerns	No concerns	No concerns	No concerns	High	NA
GLPG2222_150mg:vanza_teza_deuti_20mg_100mg_150mg	0	No concerns	Low risk	No concerns	No concerns	No concerns	No concerns	High	NA
GLPG2222_150mg:vanza_teza_deuti_5mg_100mg_150mg	0	No concerns	Low risk	No concerns	No concerns	No concerns	No concerns	High	NA
GLPG2222_150mg:vanza_teza_iva_5mg_100mg_150mg	0	No concerns	Low risk	No concerns	No concerns	No concerns	No concerns	High	NA
GLPG2222_200mg:GLPG2222_300mg	0	No concerns	Low risk	No concerns	Major concerns	No concerns	No concerns	Low	["Imprecision"]

GLPG2222_200mg:GLPG2737_75mg	0	No concerns	Low risk	No concerns	Major concerns	No concerns	No concerns	Low	["Imprecision"]
GLPG2222_200mg:Iva_150mg	0	No concerns	Low risk	No concerns	Major concerns	No concerns	No concerns	Low	["Imprecision"]
GLPG2222_200mg:Ola_teza_iva_600mg_50mg_300mg	0	No concerns	Low risk	No concerns	Major concerns	No concerns	No concerns	Low	["Imprecision"]
GLPG2222_200mg:Teza_iva_100mg_150mg	0	No concerns	Low risk	No concerns	Major concerns	No concerns	No concerns	Low	["Imprecision"]
GLPG2222_200mg:Teza_iva_100mg_50mg	0	No concerns	Low risk	No concerns	Major concerns	No concerns	No concerns	Low	["Imprecision"]
GLPG2222_200mg:Teza_iva_50mg_150mg	0	No concerns	Low risk	No concerns	Major concerns	No concerns	No concerns	Low	["Imprecision"]
GLPG2222_200mg:Teza_iva_50mg_300mg	0	No concerns	Low risk	No concerns	Major concerns	No concerns	No concerns	Low	["Imprecision"]
GLPG2222_200mg:VX152_teza_iva_100mg_100mg_150mg	0	No concerns	Low risk	No concerns	Major concerns	No concerns	No concerns	Low	["Imprecision"]
GLPG2222_200mg:VX152_teza_iva_200mg_100mg_150mg	0	No concerns	Low risk	No concerns	Major concerns	No concerns	No concerns	Low	["Imprecision"]
GLPG2222_200mg:VX152_teza_iva_300mg_100mg_150mg	0	No concerns	Low risk	No concerns	Major concerns	No concerns	No concerns	Low	["Imprecision"]
GLPG2222_200mg:luma_100mg	0	No concerns	Low risk	No concerns	Major concerns	No concerns	No concerns	Low	["Imprecision"]
GLPG2222_200mg:luma_200mg	0	Some concerns	Low risk	No concerns	Major concerns	No concerns	No concerns	Low	["Imprecision"]
GLPG2222_200mg:luma_25mg	0	No concerns	Low risk	No concerns	Major concerns	No concerns	No concerns	Low	["Imprecision"]
GLPG2222_200mg:luma_50mg	0	No concerns	Low risk	No concerns	Major concerns	No concerns	No concerns	Low	["Imprecision"]
GLPG2222_200mg:luma_iva_200mg_150mg	0	No concerns	Low risk	No concerns	Major concerns	No concerns	No concerns	Low	["Imprecision"]
GLPG2222_200mg:luma_iva_200mg_250mg	0	No concerns	Low risk	No concerns	Major concerns	No concerns	No concerns	Low	["Imprecision"]
GLPG2222_200mg:luma_iva_400mg_250mg	0	No concerns	Low risk	No concerns	Major concerns	No concerns	No concerns	Low	["Imprecision"]

GLPG2222_200mg:luma_iva_600mg_250mg	0	No concerns	Low risk	No concerns	Major concerns	No concerns	No concerns	Low	["Imprecision"]
GLPG2222_200mg:vanza_teza_deuti_10mg_100mg_150mg	0	No concerns	Low risk	No concerns	No concerns	No concerns	No concerns	High	NA
GLPG2222_200mg:vanza_teza_deuti_20mg_100mg_150mg	0	No concerns	Low risk	No concerns	No concerns	No concerns	No concerns	High	NA
GLPG2222_200mg:vanza_teza_deuti_5mg_100mg_150mg	0	No concerns	Low risk	No concerns	No concerns	No concerns	No concerns	High	NA
GLPG2222_200mg:vanza_teza_iva_5mg_100mg_150mg	0	No concerns	Low risk	No concerns	No concerns	No concerns	No concerns	High	NA
GLPG2222_300mg:GLPG2222_400mg	0	No concerns	Low risk	No concerns	Major concerns	No concerns	No concerns	Low	["Imprecision"]
GLPG2222_300mg:GLPG2222_50mg	0	No concerns	Low risk	No concerns	Major concerns	No concerns	No concerns	Low	["Imprecision"]
GLPG2222_300mg:GLPG2737_75mg	0	No concerns	Low risk	No concerns	Major concerns	No concerns	No concerns	Low	["Imprecision"]
GLPG2222_300mg:Iva_150mg	0	No concerns	Low risk	No concerns	Major concerns	No concerns	No concerns	Low	["Imprecision"]
GLPG2222_300mg:Ola_teza_iva_600mg_50mg_300mg	0	No concerns	Low risk	No concerns	Major concerns	No concerns	No concerns	Low	["Imprecision"]
GLPG2222_300mg:Teza_iva_100mg_150mg	0	No concerns	Low risk	No concerns	Major concerns	No concerns	No concerns	Low	["Imprecision"]
GLPG2222_300mg:Teza_iva_100mg_50mg	0	No concerns	Low risk	No concerns	Major concerns	No concerns	No concerns	Low	["Imprecision"]
GLPG2222_300mg:Teza_iva_50mg_150mg	0	No concerns	Low risk	No concerns	Major concerns	No concerns	No concerns	Low	["Imprecision"]
GLPG2222_300mg:Teza_iva_50mg_300mg	0	No concerns	Low risk	No concerns	Major concerns	No concerns	No concerns	Low	["Imprecision"]
GLPG2222_300mg:VX152_teza_iva_100mg_100mg_150mg	0	No concerns	Low risk	No concerns	Major concerns	No concerns	No concerns	Low	["Imprecision"]
GLPG2222_300mg:VX152_teza_iva_200mg_100mg_150mg	0	No concerns	Low risk	No concerns	Major concerns	No concerns	No concerns	Low	["Imprecision"]
GLPG2222_300mg:VX152_teza_iva_300mg_100mg_150mg	0	No concerns	Low risk	No concerns	Major concerns	No concerns	No concerns	Low	["Imprecision"]

GLPG2222_300mg:luma_100mg	0	Some concerns	Low risk	No concerns	Major concerns	No concerns	No concerns	Low	["Within-study bias"|"Imprecision"]
GLPG2222_300mg:luma_200mg	0	Some concerns	Low risk	No concerns	Major concerns	No concerns	No concerns	Low	["Within-study bias"|"Imprecision"]
GLPG2222_300mg:luma_25mg	0	Some concerns	Low risk	No concerns	Major concerns	No concerns	No concerns	Low	["Within-study bias"|"Imprecision"]
GLPG2222_300mg:luma_50mg	0	Some concerns	Low risk	No concerns	Major concerns	No concerns	No concerns	Low	["Within-study bias"|"Imprecision"]
GLPG2222_300mg:luma_iva_200mg_150mg	0	No concerns	Low risk	No concerns	Major concerns	No concerns	No concerns	Low	["Imprecision"]
GLPG2222_300mg:luma_iva_200mg_250mg	0	No concerns	Low risk	No concerns	Major concerns	No concerns	No concerns	Low	["Imprecision"]
GLPG2222_300mg:luma_iva_400mg_250mg	0	No concerns	Low risk	No concerns	Major concerns	No concerns	No concerns	Low	["Imprecision"]
GLPG2222_300mg:luma_iva_600mg_250mg	0	No concerns	Low risk	No concerns	Major concerns	No concerns	No concerns	Low	["Imprecision"]
GLPG2222_300mg:vanza_teza_deuti_10mg_100mg_150mg	0	No concerns	Low risk	No concerns	No concerns	No concerns	No concerns	High	NA
GLPG2222_300mg:vanza_teza_deuti_20mg_100mg_150mg	0	No concerns	Low risk	No concerns	No concerns	No concerns	No concerns	High	NA
GLPG2222_300mg:vanza_teza_deuti_5mg_100mg_150mg	0	No concerns	Low risk	No concerns	No concerns	Major concerns	No concerns	Low	["Heterogeneity"]
GLPG2222_300mg:vanza_teza_iva_5mg_100mg_150mg	0	No concerns	Low risk	No concerns	No concerns	No concerns	No concerns	High	NA
GLPG2222_400mg:GLPG2737_75mg	0	No concerns	Low risk	No concerns	Major concerns	No concerns	No concerns	Low	["Imprecision"]
GLPG2222_400mg:Iva_150mg	0	No concerns	Low risk	No concerns	Major concerns	No concerns	No concerns	Low	["Imprecision"]
GLPG2222_400mg:Ola_teza_iva_600mg_50mg_300mg	0	No concerns	Low risk	No concerns	No concerns	No concerns	No concerns	High	NA
GLPG2222_400mg:Teza_iva_100mg_150mg	0	No concerns	Low risk	No concerns	Major concerns	No concerns	No concerns	Low	["Imprecision"]
GLPG2222_400mg:Teza_iva_100mg_50mg	0	No concerns	Low risk	No concerns	Major concerns	No concerns	No concerns	Low	["Imprecision"]

GLPG2222_400mg:Teza_iva_50mg_150mg	0	No concerns	Low risk	No concerns	Major concerns	No concerns	No concerns	Low	["Imprecision"]
GLPG2222_400mg:Teza_iva_50mg_300mg	0	No concerns	Low risk	No concerns	Major concerns	No concerns	No concerns	Low	["Imprecision"]
GLPG2222_400mg:VX152_teza_iva_100mg_100mg_150mg	0	No concerns	Low risk	No concerns	Major concerns	No concerns	No concerns	Low	["Imprecision"]
GLPG2222_400mg:VX152_teza_iva_200mg_100mg_150mg	0	No concerns	Low risk	No concerns	Major concerns	No concerns	No concerns	Low	["Imprecision"]
GLPG2222_400mg:VX152_teza_iva_300mg_100mg_150mg	0	No concerns	Low risk	No concerns	Major concerns	No concerns	No concerns	Low	["Imprecision"]
GLPG2222_400mg:luma_100mg	0	Some concerns	Low risk	No concerns	Major concerns	No concerns	No concerns	Low	["Within-study bias"|"Imprecision"]
GLPG2222_400mg:luma_200mg	0	Some concerns	Low risk	No concerns	Major concerns	No concerns	No concerns	Low	["Within-study bias"|"Imprecision"]
GLPG2222_400mg:luma_25mg	0	Some concerns	Low risk	No concerns	Major concerns	No concerns	No concerns	Low	["Within-study bias"|"Imprecision"]
GLPG2222_400mg:luma_50mg	0	No concerns	Low risk	No concerns	Major concerns	No concerns	No concerns	Low	["Imprecision"]
GLPG2222_400mg:luma_iva_200mg_150mg	0	No concerns	Low risk	No concerns	Major concerns	No concerns	No concerns	Low	["Imprecision"]
GLPG2222_400mg:luma_iva_200mg_250mg	0	No concerns	Low risk	No concerns	Major concerns	No concerns	No concerns	Low	["Imprecision"]
GLPG2222_400mg:luma_iva_400mg_250mg	0	No concerns	Low risk	No concerns	Major concerns	No concerns	No concerns	Low	["Imprecision"]
GLPG2222_400mg:luma_iva_600mg_250mg	0	No concerns	Low risk	No concerns	Major concerns	No concerns	No concerns	Low	["Imprecision"]
GLPG2222_400mg:vanza_teza_deuti_10mg_100mg_150mg	0	No concerns	Low risk	No concerns	No concerns	No concerns	No concerns	High	NA
GLPG2222_400mg:vanza_teza_deuti_20mg_100mg_150mg	0	No concerns	Low risk	No concerns	No concerns	No concerns	No concerns	High	NA
GLPG2222_400mg:vanza_teza_deuti_5mg_100mg_150mg	0	No concerns	Low risk	No concerns	No concerns	No concerns	No concerns	High	NA
GLPG2222_400mg:vanza_teza_iva_5mg_100mg_150mg	0	No concerns	Low risk	No concerns	No concerns	No concerns	No concerns	High	NA

GLPG2222_50mg:GLPG2737_75mg	0	No concerns	Low risk	No concerns	Major concerns	No concerns	No concerns	High	["Imprecision"]
GLPG2222_50mg:Iva_150mg	0	No concerns	Low risk	No concerns	Major concerns	No concerns	No concerns	High	["Imprecision"]
GLPG2222_50mg:Ola_teza_iva_600mg_50mg_300mg	0	No concerns	Low risk	No concerns	No concerns	No concerns	No concerns	High	NA
GLPG2222_50mg:Teza_iva_100mg_150mg	0	No concerns	Low risk	No concerns	Major concerns	No concerns	No concerns	Low	["Imprecision"]
GLPG2222_50mg:Teza_iva_100mg_50mg	0	No concerns	Low risk	No concerns	Major concerns	No concerns	No concerns	Low	["Imprecision"]
GLPG2222_50mg:Teza_iva_50mg_150mg	0	No concerns	Low risk	No concerns	Major concerns	No concerns	No concerns	Low	["Imprecision"]
GLPG2222_50mg:Teza_iva_50mg_300mg	0	No concerns	Low risk	No concerns	Major concerns	No concerns	No concerns	Low	["Imprecision"]
GLPG2222_50mg:VX152_teza_iva_100mg_100mg_150mg	0	No concerns	Low risk	No concerns	Major concerns	No concerns	No concerns	Low	["Imprecision"]
GLPG2222_50mg:VX152_teza_iva_200mg_100mg_150mg	0	No concerns	Low risk	No concerns	Major concerns	No concerns	No concerns	Low	["Imprecision"]
GLPG2222_50mg:VX152_teza_iva_300mg_100mg_150mg	0	No concerns	Low risk	No concerns	Major concerns	No concerns	No concerns	Low	["Imprecision"]
GLPG2222_50mg:luma_100mg	0	Some concerns	Low risk	No concerns	Major concerns	No concerns	No concerns	Low	["Within-study bias"|"Imprecision"]
GLPG2222_50mg:luma_200mg	0	Some concerns	Low risk	No concerns	Major concerns	No concerns	No concerns	Low	["Within-study bias"|"Imprecision"]
GLPG2222_50mg:luma_25mg	0	Some concerns	Low risk	No concerns	Major concerns	No concerns	No concerns	Low	["Within-study bias"|"Imprecision"]
GLPG2222_50mg:luma_50mg	0	No concerns	Low risk	No concerns	Major concerns	No concerns	No concerns	Low	["Imprecision"]
GLPG2222_50mg:luma_iva_200mg_150mg	0	No concerns	Low risk	No concerns	Major concerns	No concerns	No concerns	Low	["Imprecision"]
GLPG2222_50mg:luma_iva_200mg_250mg	0	No concerns	Low risk	No concerns	Major concerns	No concerns	No concerns	Low	["Imprecision"]
GLPG2222_50mg:luma_iva_400mg_250mg	0	No concerns	Low risk	No concerns	Major concerns	No concerns	No concerns	Low	["Imprecision"]

GLPG2222_50mg:luma_iva_600mg_250mg	0	No concerns	Low risk	No concerns	Major concerns	No concerns	No concerns	Low	["Imprecision"]
GLPG2222_50mg:vanza_teza_deuti_10mg_100mg_150mg	0	No concerns	Low risk	No concerns	No concerns	No concerns	No concerns	High	NA
GLPG2222_50mg:vanza_teza_deuti_20mg_100mg_150mg	0	No concerns	Low risk	No concerns	No concerns	No concerns	No concerns	High	NA
GLPG2222_50mg:vanza_teza_deuti_5mg_100mg_150mg	0	No concerns	Low risk	No concerns	No concerns	No concerns	No concerns	High	NA
GLPG2222_50mg:vanza_teza_iva_5mg_100mg_150mg	0	No concerns	Low risk	No concerns	No concerns	No concerns	No concerns	High	NA
GLPG2737_75mg:Iva_150mg	0	No concerns	Low risk	No concerns	Major concerns	No concerns	No concerns	Low	["Imprecision"]
GLPG2737_75mg:Ola_teza_iva_600mg_50mg_300mg	0	No concerns	Low risk	No concerns	Major concerns	No concerns	No concerns	Low	["Imprecision"]
GLPG2737_75mg:Teza_iva_100mg_150mg	0	No concerns	Low risk	No concerns	Major concerns	No concerns	No concerns	Low	["Imprecision"]
GLPG2737_75mg:Teza_iva_100mg_50mg	0	Some concerns	Low risk	No concerns	Major concerns	No concerns	No concerns	Low	["Within-study bias"|"Imprecision"]
GLPG2737_75mg:Teza_iva_50mg_150mg	0	Some concerns	Low risk	No concerns	Major concerns	No concerns	No concerns	Low	["Within-study bias"|"Imprecision"]
GLPG2737_75mg:Teza_iva_50mg_300mg	0	No concerns	Low risk	No concerns	Major concerns	No concerns	No concerns	Low	["Imprecision"]
GLPG2737_75mg:VX152_teza_iva_100mg_100mg_150mg	0	No concerns	Low risk	No concerns	Major concerns	No concerns	No concerns	Low	["Imprecision"]
GLPG2737_75mg:VX152_teza_iva_200mg_100mg_150mg	0	No concerns	Low risk	No concerns	Major concerns	No concerns	No concerns	Low	["Imprecision"]
GLPG2737_75mg:VX152_teza_iva_300mg_100mg_150mg	0	No concerns	Low risk	No concerns	Major concerns	No concerns	No concerns	Low	["Imprecision"]
GLPG2737_75mg:luma_100mg	0	Some concerns	Low risk	No concerns	Major concerns	No concerns	No concerns	Low	["Within-study bias"|"Imprecision"]
GLPG2737_75mg:luma_200mg	0	Some concerns	Low risk	No concerns	Major concerns	No concerns	No concerns	Low	["Within-study bias"|"Imprecision"]
GLPG2737_75mg:luma_25mg	0	Some concerns	Low risk	No concerns	Major concerns	No concerns	No concerns	Low	["Within-study bias"|"Imprecision"]

GLPG2737_75mg:luma_50mg	0	Some concerns	Low risk	No concerns	Major concerns	No concerns	No concerns	Low	["Within-study bias"|"Imprecision"]
GLPG2737_75mg:luma_iva_200mg_150mg	0	No concerns	Low risk	No concerns	Major concerns	No concerns	No concerns	Low	["Imprecision"]
GLPG2737_75mg:luma_iva_200mg_250mg	0	No concerns	Low risk	No concerns	Major concerns	No concerns	No concerns	Low	["Imprecision"]
GLPG2737_75mg:luma_iva_400mg_250mg	0	No concerns	Low risk	No concerns	Major concerns	No concerns	No concerns	Low	["Imprecision"]
GLPG2737_75mg:luma_iva_600mg_250mg	0	No concerns	Low risk	No concerns	Major concerns	No concerns	No concerns	Low	["Imprecision"]
GLPG2737_75mg:vanza_teza_deuti_10mg_100mg_150mg	0	No concerns	Low risk	No concerns	No concerns	No concerns	No concerns	High	NA
GLPG2737_75mg:vanza_teza_deuti_20mg_100mg_150mg	0	No concerns	Low risk	No concerns	No concerns	No concerns	No concerns	High	NA
GLPG2737_75mg:vanza_teza_deuti_5mg_100mg_150mg	0	No concerns	Low risk	No concerns	No concerns	Major concerns	No concerns	Low	["Heterogeneity"]
GLPG2737_75mg:vanza_teza_iva_5mg_100mg_150mg	0	No concerns	Low risk	No concerns	No concerns	Major concerns	No concerns	Low	["Heterogeneity"]
Iva_150mg:Ola_teza_iva_600mg_50mg_300mg	0	No concerns	Low risk	No concerns	No concerns	No concerns	No concerns	High	NA
Iva_150mg:Placebo	0	No concerns	Low risk	No concerns	Major concerns	No concerns	No concerns	Low	["Imprecision"]
Iva_150mg:Teza_iva_100mg_50mg	0	Some concerns	Low risk	No concerns	Major concerns	No concerns	No concerns	Low	["Within-study bias"|"Imprecision"]
Iva_150mg:Teza_iva_50mg_150mg	0	Some concerns	Low risk	No concerns	Major concerns	No concerns	No concerns	Low	["Within-study bias"|"Imprecision"]
Iva_150mg:Teza_iva_50mg_300mg	0	No concerns	Low risk	No concerns	Major concerns	No concerns	No concerns	Low	["Imprecision"]
Iva_150mg:VX152_teza_iva_100mg_100mg_150mg	0	No concerns	Low risk	No concerns	Major concerns	No concerns	No concerns	Low	["Imprecision"]
Iva_150mg:VX152_teza_iva_200mg_100mg_150mg	0	No concerns	Low risk	No concerns	Major concerns	No concerns	No concerns	Low	["Imprecision"]
Iva_150mg:VX152_teza_iva_300mg_100mg_150mg	0	No concerns	Low risk	No concerns	Major concerns	No concerns	No concerns	Low	["Imprecision"]

Iva_150mg:luma_100mg	0	Some concerns	Low risk	No concerns	Major concerns	No concerns	No concerns	Low	["Within-study bias"|"Imprecision"]
Iva_150mg:luma_200mg	0	Some concerns	Low risk	No concerns	Major concerns	No concerns	No concerns	Low	["Within-study bias"|"Imprecision"]
Iva_150mg:luma_25mg	0	Some concerns	Low risk	No concerns	Major concerns	No concerns	No concerns	Low	["Within-study bias"|"Imprecision"]
Iva_150mg:luma_50mg	0	Some concerns	Low risk	No concerns	Major concerns	No concerns	No concerns	Low	["Within-study bias"|"Imprecision"]
Iva_150mg:luma_iva_200mg_150mg	0	No concerns	Low risk	No concerns	Major concerns	No concerns	No concerns	Low	["Imprecision"]
Iva_150mg:luma_iva_200mg_250mg	0	No concerns	Low risk	No concerns	Major concerns	No concerns	No concerns	Low	["Imprecision"]
Iva_150mg:luma_iva_400mg_250mg	0	No concerns	Low risk	No concerns	Major concerns	No concerns	No concerns	Low	["Imprecision"]
Iva_150mg:luma_iva_600mg_250mg	0	No concerns	Low risk	No concerns	Major concerns	No concerns	No concerns	Low	["Imprecision"]
Iva_150mg:vanza_teza_deuti_10mg_100mg_150mg	0	No concerns	Low risk	No concerns	No concerns	No concerns	No concerns	High	NA
Iva_150mg:vanza_teza_deuti_20mg_100mg_150mg	0	No concerns	Low risk	No concerns	No concerns	No concerns	No concerns	High	NA
Iva_150mg:vanza_teza_deuti_5mg_100mg_150mg	0	No concerns	Low risk	No concerns	No concerns	No concerns	No concerns	High	NA
Iva_150mg:vanza_teza_iva_5mg_100mg_150mg	0	No concerns	Low risk	No concerns	No concerns	No concerns	No concerns	High	NA
Ola_teza_iva_600mg_50mg_300mg:Teza_iva_100mg_150mg	0	No concerns	Low risk	No concerns	No concerns	No concerns	No concerns	High	NA
Ola_teza_iva_600mg_50mg_300mg:Teza_iva_100mg_50mg	0	Some concerns	Low risk	No concerns	No concerns	No concerns	No concerns	Moderate	["Within-study bias"]
Ola_teza_iva_600mg_50mg_300mg:Teza_iva_50mg_150mg	0	Some concerns	Low risk	No concerns	No concerns	No concerns	No concerns	Moderate	["Within-study bias"]
Ola_teza_iva_600mg_50mg_300mg:VX152_teza_iva_100mg_100mg_150mg	0	No concerns	Low risk	No concerns	No concerns	Major concerns	No concerns	Low	["Heterogeneity"]
Ola_teza_iva_600mg_50mg_300mg:VX152_teza_iva_200mg_100mg_150mg	0	No concerns	Low risk	No concerns	No concerns	Major concerns	No concerns	Low	["Heterogeneity"]

Ola_teza_iva_600mg_50mg_300mg:VX152_teza_iva_300mg_100mg_150mg	0	No concerns	Low risk	No concerns	Major concerns	No concerns	No concerns	Low	["Imprecision"]
luma_100mg:Ola_teza_iva_600mg_50mg_300mg	0	Some concerns	Low risk	No concerns	No concerns	No concerns	No concerns	Moderate	["Within-study bias"]
luma_200mg:Ola_teza_iva_600mg_50mg_300mg	0	Some concerns	Low risk	No concerns	No concerns	No concerns	No concerns	Moderate	["Within-study bias"]
luma_25mg:Ola_teza_iva_600mg_50mg_300mg	0	Some concerns	Low risk	No concerns	No concerns	No concerns	No concerns	Moderate	["Within-study bias"]
luma_50mg:Ola_teza_iva_600mg_50mg_300mg	0	Some concerns	Low risk	No concerns	No concerns	No concerns	No concerns	Moderate	["Within-study bias"]
luma_iva_200mg_150mg:Ola_teza_iva_600mg_50mg_300mg	0	No concerns	Low risk	No concerns	No concerns	No concerns	No concerns	High	NA
luma_iva_200mg_250mg:Ola_teza_iva_600mg_50mg_300mg	0	No concerns	Low risk	No concerns	No concerns	No concerns	No concerns	High	NA
luma_iva_400mg_250mg:Ola_teza_iva_600mg_50mg_300mg	0	No concerns	Low risk	No concerns	No concerns	Major concerns	No concerns	Low	["Heterogeneity"]
luma_iva_600mg_250mg:Ola_teza_iva_600mg_50mg_300mg	0	No concerns	Low risk	No concerns	No concerns	Major concerns	No concerns	Low	["Heterogeneity"]
Ola_teza_iva_600mg_50mg_300mg:vanza_teza_deuti_10mg_100mg_150mg	0	No concerns	Low risk	No concerns	Major concerns	No concerns	No concerns	Low	["Imprecision"]
Ola_teza_iva_600mg_50mg_300mg:vanza_teza_deuti_20mg_100mg_150mg	0	No concerns	Low risk	No concerns	Major concerns	No concerns	No concerns	Low	["Imprecision"]
Ola_teza_iva_600mg_50mg_300mg:vanza_teza_deuti_5mg_100mg_150mg	0	No concerns	Low risk	No concerns	Major concerns	No concerns	No concerns	Low	["Imprecision"]
Ola_teza_iva_600mg_50mg_300mg:vanza_teza_iva_5mg_100mg_150mg	0	No concerns	Low risk	No concerns	Major concerns	No concerns	No concerns	Low	["Imprecision"]
Placebo:Teza_iva_50mg_300mg	0	No concerns	Low risk	No concerns	Major concerns	No concerns	No concerns	Low	["Imprecision"]
Teza_iva_100mg_150mg:Teza_iva_100mg_50mg	0	No concerns	Low risk	No concerns	Major concerns	No concerns	No concerns	Low	["Imprecision"]
Teza_iva_100mg_150mg:Teza_iva_50mg_150mg	0	No concerns	Low risk	No concerns	Major concerns	No concerns	No concerns	Low	["Imprecision"]
Teza_iva_100mg_150mg:Teza_iva_50mg_300mg	0	No concerns	Low risk	No concerns	Major concerns	No concerns	No concerns	Low	["Imprecision"]

Teza_iva_100mg_150mg:VX152_teza_iva_100mg_100mg_150 mg	0	No concerns	Low risk	No concerns	Major concerns	No concerns	No concerns	Low	["Imprecision"]
luma_100mg:Teza_iva_100mg_150mg	0	Some concerns	Low risk	No concerns	Major concerns	No concerns	No concerns	Low	["Within-study bias"|"Imprecision"]
luma_200mg:Teza_iva_100mg_150mg	0	Some concerns	Low risk	No concerns	Major concerns	No concerns	No concerns	Low	["Within-study bias"|"Imprecision"]
luma_25mg:Teza_iva_100mg_150mg	0	Some concerns	Low risk	No concerns	Major concerns	No concerns	No concerns	Low	["Within-study bias"|"Imprecision"]
luma_50mg:Teza_iva_100mg_150mg	0	Some concerns	Low risk	No concerns	Major concerns	No concerns	No concerns	Low	["Within-study bias"|"Imprecision"]
luma_iva_200mg_150mg:Teza_iva_100mg_150mg	0	No concerns	Low risk	No concerns	Major concerns	No concerns	No concerns	Low	["Imprecision"]
luma_iva_200mg_250mg:Teza_iva_100mg_150mg	0	No concerns	Low risk	No concerns	Major concerns	No concerns	No concerns	Low	["Imprecision"]
luma_iva_400mg_250mg:Teza_iva_100mg_150mg	0	No concerns	Low risk	No concerns	Major concerns	No concerns	No concerns	Low	["Imprecision"]
luma_iva_600mg_250mg:Teza_iva_100mg_150mg	0	No concerns	Low risk	No concerns	Major concerns	No concerns	No concerns	Low	["Imprecision"]
Teza_iva_100mg_150mg:vanza_teza_deuti_10mg_100mg_150m g	0	No concerns	Low risk	No concerns	No concerns	No concerns	No concerns	High	NA
Teza_iva_100mg_150mg:vanza_teza_deuti_5mg_100mg_150mg	0	No concerns	Low risk	No concerns	No concerns	No concerns	No concerns	High	NA
Teza_iva_100mg_150mg:vanza_teza_iva_5mg_100mg_150mg	0	No concerns	Low risk	No concerns	No concerns	No concerns	No concerns	High	NA
Teza_iva_100mg_50mg:Teza_iva_50mg_300mg	0	No concerns	Low risk	No concerns	Major concerns	No concerns	No concerns	Low	["Imprecision"]
Teza_iva_100mg_50mg:VX152_teza_iva_100mg_100mg_150m g	0	No concerns	Low risk	No concerns	Major concerns	No concerns	No concerns	Low	["Imprecision"]
Teza_iva_100mg_50mg:VX152_teza_iva_200mg_100mg_150m g	0	No concerns	Low risk	No concerns	Major concerns	No concerns	No concerns	Low	["Imprecision"]
Teza_iva_100mg_50mg:VX152_teza_iva_300mg_100mg_150m g	0	No concerns	Low risk	No concerns	Major concerns	No concerns	No concerns	Low	["Imprecision"]
luma_100mg:Teza_iva_100mg_50mg	0	Some concerns	Low risk	No concerns	Major concerns	No concerns	No concerns	Low	["Within-study bias"|"Imprecision"]

luma_200mg:Teza_iva_100mg_50mg	0	Some concerns	Low risk	No concerns	Major concerns	No concerns	No concerns	Low	["Within-study bias"|"Imprecision"]
luma_25mg:Teza_iva_100mg_50mg	0	Some concerns	Low risk	No concerns	Major concerns	No concerns	No concerns	Low	["Within-study bias"|"Imprecision"]
luma_50mg:Teza_iva_100mg_50mg	0	Some concerns	Low risk	No concerns	Major concerns	No concerns	No concerns	Low	["Within-study bias"|"Imprecision"]
luma_iva_200mg_150mg:Teza_iva_100mg_50mg	0	No concerns	Low risk	No concerns	Major concerns	No concerns	No concerns	Low	["Imprecision"]
luma_iva_200mg_250mg:Teza_iva_100mg_50mg	0	Some concerns	Low risk	No concerns	Major concerns	No concerns	No concerns	Low	["Within-study bias"|"Imprecision"]
luma_iva_400mg_250mg:Teza_iva_100mg_50mg	0	Some concerns	Low risk	No concerns	Major concerns	No concerns	No concerns	Low	["Within-study bias"|"Imprecision"]
luma_iva_600mg_250mg:Teza_iva_100mg_50mg	0	No concerns	Low risk	No concerns	Major concerns	No concerns	No concerns	Low	["Imprecision"]
Teza_iva_100mg_50mg:vanza_teza_deuti_10mg_100mg_150mg	0	No concerns	Low risk	No concerns	No concerns	No concerns	No concerns	High	NA
Teza_iva_100mg_50mg:vanza_teza_deuti_20mg_100mg_150mg	0	No concerns	Low risk	No concerns	No concerns	No concerns	No concerns	High	NA
Teza_iva_100mg_50mg:vanza_teza_deuti_5mg_100mg_150mg	0	No concerns	Low risk	No concerns	No concerns	No concerns	No concerns	High	NA
Teza_iva_100mg_50mg:vanza_teza_iva_5mg_100mg_150mg	0	Some concerns	Low risk	No concerns	No concerns	No concerns	No concerns	Moderate	["Within-study bias"]
Teza_iva_50mg_150mg:Teza_iva_50mg_300mg	0	No concerns	Low risk	No concerns	Major concerns	No concerns	No concerns	Low	["Imprecision"]
Teza_iva_50mg_150mg:VX152_teza_iva_100mg_100mg_150mg	0	No concerns	Low risk	No concerns	Major concerns	No concerns	No concerns	Low	["Imprecision"]
Teza_iva_50mg_150mg:VX152_teza_iva_200mg_100mg_150mg	0	No concerns	Low risk	No concerns	Major concerns	No concerns	No concerns	Low	["Imprecision"]
Teza_iva_50mg_150mg:VX152_teza_iva_300mg_100mg_150mg	0	No concerns	Low risk	No concerns	Major concerns	No concerns	No concerns	Low	["Imprecision"]
luma_100mg:Teza_iva_50mg_150mg	0	Some concerns	Low risk	No concerns	Major concerns	No concerns	No concerns	Low	["Within-study bias"|"Imprecision"]
luma_200mg:Teza_iva_50mg_150mg	0	Some concerns	Low risk	No concerns	Major concerns	No concerns	No concerns	Low	["Within-study bias"|"Imprecision"]

luma_25mg:Teza_iva_50mg_150mg	0	Some concerns	Low risk	No concerns	Major concerns	No concerns	No concerns	Low	["Within-study bias"|"Imprecision"]
luma_50mg:Teza_iva_50mg_150mg	0	Some concerns	Low risk	No concerns	Major concerns	No concerns	No concerns	Low	["Within-study bias"|"Imprecision"]
luma_iva_200mg_150mg:Teza_iva_50mg_150mg	0	No concerns	Low risk	No concerns	Major concerns	No concerns	No concerns	Low	["Imprecision"]
luma_iva_200mg_250mg:Teza_iva_50mg_150mg	0	Some concerns	Low risk	No concerns	Major concerns	No concerns	No concerns	Low	["Within-study bias"|"Imprecision"]
luma_iva_400mg_250mg:Teza_iva_50mg_150mg	0	Some concerns	Low risk	No concerns	Major concerns	No concerns	No concerns	Low	["Within-study bias"|"Imprecision"]
luma_iva_600mg_250mg:Teza_iva_50mg_150mg	0	No concerns	Low risk	No concerns	Major concerns	No concerns	No concerns	Low	["Imprecision"]
Teza_iva_50mg_150mg:vanza_teza_deuti_10mg_100mg_150mg	0	No concerns	Low risk	No concerns	No concerns	No concerns	No concerns	High	NA
Teza_iva_50mg_150mg:vanza_teza_deuti_20mg_100mg_150mg	0	No concerns	Low risk	No concerns	No concerns	No concerns	No concerns	High	NA
Teza_iva_50mg_150mg:vanza_teza_deuti_5mg_100mg_150mg	0	No concerns	Low risk	No concerns	No concerns	No concerns	No concerns	High	NA
Teza_iva_50mg_150mg:vanza_teza_iva_5mg_100mg_150mg	0	Some concerns	Low risk	No concerns	No concerns	No concerns	No concerns	Moderate	["Within-study bias"]
Teza_iva_50mg_300mg:VX152_teza_iva_100mg_100mg_150mg	0	No concerns	Low risk	No concerns	Major concerns	No concerns	No concerns	Low	["Imprecision"]
Teza_iva_50mg_300mg:VX152_teza_iva_200mg_100mg_150mg	0	No concerns	Low risk	No concerns	Major concerns	No concerns	No concerns	Low	["Imprecision"]
Teza_iva_50mg_300mg:VX152_teza_iva_300mg_100mg_150mg	0	No concerns	Low risk	No concerns	Major concerns	No concerns	No concerns	Low	["Imprecision"]
luma_100mg:Teza_iva_50mg_300mg	0	No concerns	Low risk	No concerns	Major concerns	No concerns	No concerns	Low	["Imprecision"]
luma_200mg:Teza_iva_50mg_300mg	0	No concerns	Low risk	No concerns	Major concerns	No concerns	No concerns	Low	["Imprecision"]
luma_25mg:Teza_iva_50mg_300mg	0	No concerns	Low risk	No concerns	Major concerns	No concerns	No concerns	Low	["Imprecision"]
luma_50mg:Teza_iva_50mg_300mg	0	No concerns	Low risk	No concerns	Major concerns	No concerns	No concerns	Low	["Imprecision"]

luma_iva_200mg_150mg:Teza_iva_50mg_300mg	0	No concerns	Low risk	No concerns	Major concerns	No concerns	No concerns	Low	["Imprecision"]
luma_iva_200mg_250mg:Teza_iva_50mg_300mg	0	No concerns	Low risk	No concerns	Major concerns	No concerns	No concerns	Low	["Imprecision"]
luma_iva_400mg_250mg:Teza_iva_50mg_300mg	0	No concerns	Low risk	No concerns	Major concerns	No concerns	No concerns	Low	["Imprecision"]
luma_iva_600mg_250mg:Teza_iva_50mg_300mg	0	No concerns	Low risk	No concerns	Major concerns	No concerns	No concerns	Low	["Imprecision"]
Teza_iva_50mg_300mg:vanza_teza_deuti_10mg_100mg_150mg	0	No concerns	Low risk	No concerns	No concerns	No concerns	No concerns	High	NA
Teza_iva_50mg_300mg:vanza_teza_deuti_20mg_100mg_150mg	0	No concerns	Low risk	No concerns	No concerns	No concerns	No concerns	High	NA
Teza_iva_50mg_300mg:vanza_teza_deuti_5mg_100mg_150mg	0	No concerns	Low risk	No concerns	No concerns	No concerns	No concerns	High	NA
Teza_iva_50mg_300mg:vanza_teza_iva_5mg_100mg_150mg	0	No concerns	Low risk	No concerns	No concerns	No concerns	No concerns	High	NA
luma_100mg:VX152_teza_iva_100mg_100mg_150mg	0	No concerns	Low risk	No concerns	Major concerns	No concerns	No concerns	Low	["Imprecision"]
luma_200mg:VX152_teza_iva_100mg_100mg_150mg	0	No concerns	Low risk	No concerns	Major concerns	No concerns	No concerns	Low	["Imprecision"]
luma_25mg:VX152_teza_iva_100mg_100mg_150mg	0	No concerns	Low risk	No concerns	Major concerns	No concerns	No concerns	Low	["Imprecision"]
luma_50mg:VX152_teza_iva_100mg_100mg_150mg	0	No concerns	Low risk	No concerns	Major concerns	No concerns	No concerns	Low	["Imprecision"]
luma_iva_200mg_150mg:VX152_teza_iva_100mg_100mg_150 mg	0	No concerns	Low risk	No concerns	Major concerns	No concerns	No concerns	Low	["Imprecision"]
luma_iva_200mg_250mg:VX152_teza_iva_100mg_100mg_150 mg	0	No concerns	Low risk	No concerns	Major concerns	No concerns	No concerns	Low	["Imprecision"]
luma_iva_400mg_250mg:VX152_teza_iva_100mg_100mg_150 mg	0	No concerns	Low risk	No concerns	Major concerns	No concerns	No concerns	Low	["Imprecision"]
luma_iva_600mg_250mg:VX152_teza_iva_100mg_100mg_150 mg	0	No concerns	Low risk	No concerns	Major concerns	No concerns	No concerns	Low	["Imprecision"]
vanza_teza_deuti_10mg_100mg_150mg:VX152_teza_iva_100m g_100mg_150mg	0	No concerns	Low risk	No concerns	No concerns	No concerns	No concerns	High	NA

vanza_teza_deuti_20mg_100mg_150mg:VX152_teza_iva_100mg_100mg_150mg	0	No concerns	Low risk	No concerns	No concerns	No concerns	No concerns	High	NA
vanza_teza_deuti_5mg_100mg_150mg:VX152_teza_iva_100mg_100mg_150mg	0	No concerns	Low risk	No concerns	No concerns	No concerns	No concerns	High	NA
vanza_teza_iva_5mg_100mg_150mg:VX152_teza_iva_100mg_100mg_150mg	0	No concerns	Low risk	No concerns	No concerns	No concerns	No concerns	High	NA
luma_100mg:VX152_teza_iva_200mg_100mg_150mg	0	No concerns	Low risk	No concerns	Major concerns	No concerns	No concerns	Low	["Imprecision"]
luma_200mg:VX152_teza_iva_200mg_100mg_150mg	0	No concerns	Low risk	No concerns	Major concerns	No concerns	No concerns	Low	["Imprecision"]
luma_25mg:VX152_teza_iva_200mg_100mg_150mg	0	No concerns	Low risk	No concerns	Major concerns	No concerns	No concerns	Low	["Imprecision"]
luma_50mg:VX152_teza_iva_200mg_100mg_150mg	0	No concerns	Low risk	No concerns	Major concerns	No concerns	No concerns	Low	["Imprecision"]
luma_iva_200mg_150mg:VX152_teza_iva_200mg_100mg_150mg	0	No concerns	Low risk	No concerns	Major concerns	No concerns	No concerns	Low	["Imprecision"]
luma_iva_200mg_250mg:VX152_teza_iva_200mg_100mg_150mg	0	No concerns	Low risk	No concerns	Major concerns	No concerns	No concerns	Low	["Imprecision"]
luma_iva_400mg_250mg:VX152_teza_iva_200mg_100mg_150mg	0	No concerns	Low risk	No concerns	Major concerns	No concerns	No concerns	Low	["Imprecision"]
luma_iva_600mg_250mg:VX152_teza_iva_200mg_100mg_150mg	0	No concerns	Low risk	No concerns	Major concerns	No concerns	No concerns	Low	["Imprecision"]
vanza_teza_deuti_10mg_100mg_150mg:VX152_teza_iva_200mg_100mg_150mg	0	No concerns	Low risk	No concerns	No concerns	No concerns	No concerns	High	NA
vanza_teza_deuti_20mg_100mg_150mg:VX152_teza_iva_200mg_100mg_150mg	0	No concerns	Low risk	No concerns	No concerns	No concerns	No concerns	High	NA
vanza_teza_deuti_5mg_100mg_150mg:VX152_teza_iva_200mg_100mg_150mg	0	No concerns	Low risk	No concerns	No concerns	No concerns	No concerns	High	NA
vanza_teza_iva_5mg_100mg_150mg:VX152_teza_iva_200mg_100mg_150mg	0	No concerns	Low risk	No concerns	No concerns	No concerns	No concerns	High	NA
luma_100mg:VX152_teza_iva_300mg_100mg_150mg	0	No concerns	Low risk	No concerns	Major concerns	No concerns	No concerns	Low	["Imprecision"]
luma_200mg:VX152_teza_iva_300mg_100mg_150mg	0	No concerns	Low risk	No concerns	Major concerns	No concerns	No concerns	Low	["Imprecision"]

luma_25mg:VX152_teza_iva_300mg_100mg_150mg	0	No concerns	Low risk	No concerns	No concerns	Major concerns	No concerns	Low	["Heterogeneity"]
luma_50mg:VX152_teza_iva_300mg_100mg_150mg	0	No concerns	Low risk	No concerns	No concerns	Major concerns	No concerns	Low	["Heterogeneity"]
luma_iva_200mg_150mg:VX152_teza_iva_300mg_100mg_150 mg	0	No concerns	Low risk	No concerns	No concerns	Major concerns	No concerns	Low	["Heterogeneity"]
luma_iva_200mg_250mg:VX152_teza_iva_300mg_100mg_150 mg	0	No concerns	Low risk	No concerns	Major concerns	No concerns	No concerns	Low	["Imprecision"]
luma_iva_400mg_250mg:VX152_teza_iva_300mg_100mg_150 mg	0	No concerns	Low risk	No concerns	Major concerns	No concerns	No concerns	Low	["Imprecision"]
luma_iva_600mg_250mg:VX152_teza_iva_300mg_100mg_150 mg	0	No concerns	Low risk	No concerns	Major concerns	No concerns	No concerns	Low	["Imprecision"]
vanza_teza_deuti_10mg_100mg_150mg:VX152_teza_iva_300m g_100mg_150mg	0	No concerns	Low risk	No concerns	No concerns	No concerns	No concerns	High	NA
vanza_teza_deuti_20mg_100mg_150mg:VX152_teza_iva_300m g_100mg_150mg	0	No concerns	Low risk	No concerns	No concerns	No concerns	No concerns	High	NA
vanza_teza_deuti_5mg_100mg_150mg:VX152_teza_iva_300mg _100mg_150mg	0	No concerns	Low risk	No concerns	No concerns	Major concerns	No concerns	Low	["Heterogeneity"]
vanza_teza_iva_5mg_100mg_150mg:VX152_teza_iva_300mg_1 00mg_150mg	0	No concerns	Low risk	No concerns	No concerns	Major concerns	No concerns	Low	["Heterogeneity"]
luma_100mg:luma_iva_200mg_150mg	0	Some concerns	Low risk	No concerns	Major concerns	No concerns	No concerns	Low	["Within-study bias"|"Imprecision"]
luma_100mg:luma_iva_200mg_250mg	0	Some concerns	Low risk	No concerns	Major concerns	No concerns	No concerns	Low	["Within-study bias"|"Imprecision"]
luma_100mg:luma_iva_400mg_250mg	0	Some concerns	Low risk	No concerns	Major concerns	No concerns	No concerns	Low	["Within-study bias"|"Imprecision"]
luma_100mg:luma_iva_600mg_250mg	0	Some concerns	Low risk	No concerns	Major concerns	No concerns	No concerns	Low	["Within-study bias"|"Imprecision"]
luma_100mg:vanza_teza_deuti_10mg_100mg_150mg	0	No concerns	Low risk	No concerns	No concerns	No concerns	No concerns	High	NA
luma_100mg:vanza_teza_deuti_20mg_100mg_150mg	0	No concerns	Low risk	No concerns	No concerns	No concerns	No concerns	High	NA
luma_100mg:vanza_teza_deuti_5mg_100mg_150mg	0	No concerns	Low risk	No concerns	No concerns	No concerns	No concerns	High	NA

luma_100mg:vanza_teza_iva_5mg_100mg_150mg	0	Some concerns	Low risk	No concerns	No concerns	No concerns	No concerns	Moderate	["Within-study bias"]
luma_200mg:luma_iva_200mg_150mg	0	Some concerns	Low risk	No concerns	Major concerns	No concerns	No concerns	Low	["Within-study bias"|"Imprecision"]
luma_200mg:luma_iva_200mg_250mg	0	Some concerns	Low risk	No concerns	Major concerns	No concerns	No concerns	Low	["Within-study bias"|"Imprecision"]
luma_200mg:luma_iva_400mg_250mg	0	Some concerns	Low risk	No concerns	Major concerns	No concerns	No concerns	Low	["Within-study bias"|"Imprecision"]
luma_200mg:luma_iva_600mg_250mg	0	Some concerns	Low risk	No concerns	Major concerns	No concerns	No concerns	Low	["Within-study bias"|"Imprecision"]
luma_200mg:vanza_teza_deuti_10mg_100mg_150mg	0	No concerns	Low risk	No concerns	No concerns	No concerns	No concerns	High	NA
luma_200mg:vanza_teza_deuti_20mg_100mg_150mg	0	No concerns	Low risk	No concerns	No concerns	No concerns	No concerns	High	NA
luma_200mg:vanza_teza_deuti_5mg_100mg_150mg	0	No concerns	Low risk	No concerns	No concerns	No concerns	No concerns	High	NA
luma_200mg:vanza_teza_iva_5mg_100mg_150mg	0	Some concerns	Low risk	No concerns	No concerns	No concerns	No concerns	Moderate	["Within-study bias"]
luma_25mg:luma_iva_200mg_150mg	0	Some concerns	Low risk	No concerns	Major concerns	No concerns	No concerns	Low	["Within-study bias"|"Imprecision"]
luma_25mg:luma_iva_200mg_250mg	0	Some concerns	Low risk	No concerns	Major concerns	No concerns	No concerns	Low	["Within-study bias"|"Imprecision"]
luma_25mg:luma_iva_400mg_250mg	0	Some concerns	Low risk	No concerns	Major concerns	No concerns	No concerns	Low	["Within-study bias"|"Imprecision"]
luma_25mg:luma_iva_600mg_250mg	0	Some concerns	Low risk	No concerns	Major concerns	No concerns	No concerns	Low	["Within-study bias"|"Imprecision"]
luma_25mg:vanza_teza_deuti_10mg_100mg_150mg	0	No concerns	Low risk	No concerns	No concerns	No concerns	No concerns	High	NA
luma_25mg:vanza_teza_deuti_20mg_100mg_150mg	0	No concerns	Low risk	No concerns	No concerns	No concerns	No concerns	High	NA
luma_25mg:vanza_teza_deuti_5mg_100mg_150mg	0	No concerns	Low risk	No concerns	No concerns	No concerns	No concerns	High	NA
luma_25mg:vanza_teza_iva_5mg_100mg_150mg	0	Some concerns	Low risk	No concerns	No concerns	No concerns	No concerns	Moderate	["Within-study bias"]

luma_50mg:luma_iva_200mg_150mg	0	Some concerns	Low risk	No concerns	Major concerns	No concerns	No concerns	Low	["Within-study bias"|"Imprecision"]
luma_50mg:luma_iva_200mg_250mg	0	Some concerns	Low risk	No concerns	Major concerns	No concerns	No concerns	Low	["Within-study bias"|"Imprecision"]
luma_50mg:luma_iva_400mg_250mg	0	Some concerns	Low risk	No concerns	Major concerns	No concerns	No concerns	Low	["Within-study bias"|"Imprecision"]
luma_50mg:luma_iva_600mg_250mg	0	Some concerns	Low risk	No concerns	Major concerns	No concerns	No concerns	Low	["Within-study bias"|"Imprecision"]
luma_50mg:vanza_teza_deuti_10mg_100mg_150mg	0	No concerns	Low risk	No concerns	No concerns	No concerns	No concerns	High	NA
luma_50mg:vanza_teza_deuti_20mg_100mg_150mg	0	No concerns	Low risk	No concerns	No concerns	No concerns	No concerns	High	NA
luma_50mg:vanza_teza_deuti_5mg_100mg_150mg	0	No concerns	Low risk	No concerns	No concerns	No concerns	No concerns	High	NA
luma_50mg:vanza_teza_iva_5mg_100mg_150mg	0	Some concerns	Low risk	No concerns	No concerns	No concerns	No concerns	Moderate	["Within-study bias"]
luma_iva_200mg_150mg:luma_iva_400mg_250mg	0	No concerns	Low risk	No concerns	Major concerns	No concerns	No concerns	Low	["Imprecision"]
luma_iva_200mg_150mg:luma_iva_600mg_250mg	0	No concerns	Low risk	No concerns	Major concerns	No concerns	No concerns	Low	["Imprecision"]
luma_iva_200mg_150mg:vanza_teza_deuti_10mg_100mg_150mg	0	No concerns	Low risk	No concerns	No concerns	No concerns	No concerns	High	NA
luma_iva_200mg_150mg:vanza_teza_deuti_20mg_100mg_150mg	0	No concerns	Low risk	No concerns	No concerns	No concerns	No concerns	High	NA
luma_iva_200mg_150mg:vanza_teza_deuti_5mg_100mg_150mg	0	No concerns	Low risk	No concerns	No concerns	No concerns	No concerns	High	NA
luma_iva_200mg_150mg:vanza_teza_iva_5mg_100mg_150mg	0	No concerns	Low risk	No concerns	No concerns	No concerns	No concerns	High	NA
luma_iva_200mg_250mg:vanza_teza_deuti_10mg_100mg_150mg	0	No concerns	Low risk	No concerns	No concerns	No concerns	No concerns	High	NA
luma_iva_200mg_250mg:vanza_teza_deuti_20mg_100mg_150mg	0	No concerns	Low risk	No concerns	No concerns	No concerns	No concerns	High	NA
luma_iva_200mg_250mg:vanza_teza_deuti_5mg_100mg_150mg	0	No concerns	Low risk	No concerns	No concerns	No concerns	No concerns	High	NA

luma_iva_200mg_250mg:vanza_teza_iva_5mg_100mg_150mg	0	No concerns	Low risk	No concerns	No concerns	No concerns	No concerns	High	NA
luma_iva_400mg_250mg:vanza_teza_deuti_10mg_100mg_150mg	0	No concerns	Low risk	No concerns	No concerns	No concerns	No concerns	High	NA
luma_iva_400mg_250mg:vanza_teza_deuti_20mg_100mg_150mg	0	No concerns	Low risk	No concerns	No concerns	No concerns	No concerns	High	NA
luma_iva_400mg_250mg:vanza_teza_deuti_5mg_100mg_150mg	0	No concerns	Low risk	No concerns	No concerns	No concerns	No concerns	High	NA
luma_iva_400mg_250mg:vanza_teza_iva_5mg_100mg_150mg	0	No concerns	Low risk	No concerns	No concerns	No concerns	No concerns	High	NA
luma_iva_600mg_250mg:vanza_teza_deuti_10mg_100mg_150mg	0	No concerns	Low risk	No concerns	No concerns	No concerns	No concerns	High	NA
luma_iva_600mg_250mg:vanza_teza_deuti_20mg_100mg_150mg	0	No concerns	Low risk	No concerns	No concerns	No concerns	No concerns	High	NA
luma_iva_600mg_250mg:vanza_teza_deuti_5mg_100mg_150mg	0	No concerns	Low risk	No concerns	No concerns	No concerns	No concerns	High	NA
luma_iva_600mg_250mg:vanza_teza_iva_5mg_100mg_150mg	0	No concerns	Low risk	No concerns	No concerns	No concerns	No concerns	High	NA
vanza_teza_deuti_10mg_100mg_150mg:vanza_teza_iva_5mg_100mg_150mg	0	No concerns	Low risk	No concerns	Major concerns	No concerns	No concerns	Low	["Imprecision"]
vanza_teza_deuti_20mg_100mg_150mg:vanza_teza_iva_5mg_100mg_150mg	0	No concerns	Low risk	No concerns	Major concerns	No concerns	No concerns	Low	["Imprecision"]
vanza_teza_deuti_5mg_100mg_150mg:vanza_teza_iva_5mg_100mg_150mg	0	No concerns	Low risk	No concerns	Major concerns	No concerns	No concerns	Low	["Imprecision"]

eTable 10 Certainty ratings of sweat chloride for adults treated for greater than 8 weeks using CINEMA framework

Comparison	Number of studies	Within-study bias	Reporting bias	Indirectness	Imprecision	Heterogeneity	Incoherence	Confidence rating	Reason(s) for downgrading
Mixed evidence									
Elexa_teza_iva_200mg_100mg_150mg:Placebo	1	No concerns	Low risk	No concerns	No concerns	No concerns	No concerns	High	NA
Elexa_teza_iva_200mg_100mg_150mg:Teza_iva_100mg_150mg	1	No concerns	Low risk	No concerns	No concerns	No concerns	No concerns	High	NA
Elexa_teza_iva_200mg_100mg_150mg:vanza_teza_deuti_20mg_100mg_250mg	4	No concerns	Low risk	No concerns	No concerns	Major concerns	No concerns	Low	["Heterogeneity"]
Iva_150mg:Placebo	1	No concerns	Low risk	No concerns	Major concerns	No concerns	No concerns	Low	["Imprecision"]
Placebo:Teza_iva_100mg_150mg	3	Some concerns	Low risk	No concerns	No concerns	Major concerns	No concerns	Low	["Within-study bias","Heterogeneity"]
Placebo:Teza_iva_50mg_150mg	1	Some concerns	Low risk	No concerns	No concerns	Major concerns	No concerns	Low	["Within-study bias","Heterogeneity"]
Indirect evidence									
Elexa_teza_iva_200mg_100mg_150mg:Iva_150mg	0	No concerns	Low risk	No concerns	No concerns	No concerns	No concerns	High	NA
Elexa_teza_iva_200mg_100mg_150mg:Teza_iva_50mg_150mg	0	Some concerns	Low risk	No concerns	No concerns	No concerns	No concerns	Moderate	["Within-study bias"]
Iva_150mg:Teza_iva_100mg_150mg	0	No concerns	Low risk	No concerns	Major concerns	No concerns	No concerns	Low	["Imprecision"]
Iva_150mg:Teza_iva_50mg_150mg	0	Some concerns	Low risk	No concerns	Major concerns	No concerns	No concerns	Low	["Within-study bias","Imprecision"]
Iva_150mg:vanza_teza_deuti_20mg_100mg_250mg	0	No concerns	Low risk	No concerns	No concerns	No concerns	No concerns	High	NA
Placebo:vanza_teza_deuti_20mg_100mg_250mg	0	No concerns	Low risk	No concerns	No concerns	No concerns	No concerns	High	NA
Teza_iva_100mg_150mg:Teza_iva_50mg_150mg	0	Some concerns	Low risk	No concerns	Major concerns	No concerns	No concerns	Low	["Within-study bias","Imprecision"]
Teza_iva_100mg_150mg:vanza_teza_deuti_20mg_100mg_250mg	0	No concerns	Low risk	No concerns	No concerns	No concerns	No concerns	High	NA
Teza_iva_50mg_150mg:vanza_teza_deuti_20mg_100mg_250mg	0	No concerns	Low risk	No concerns	No concerns	No concerns	No concerns	High	NA

eTable 11 Certainty ratings of CFQ-R for adults treated for 4 to 8 weeks using CINEMA framework

Comparison	Number of studies	Within-study bias	Reporting bias	Indirectness	Imprecision	Heterogeneity	Incoherence	Confidence rating	Reason(s) for downgrading
Mixed evidence									
Elexa_teza_iva_100mg_100mg_150mg:Elexa_teza_iva_200mg_100mg_150mg	1	No concerns	Low risk	No concerns	No concerns	No concerns	No concerns	High	NA
Elexa_teza_iva_100mg_100mg_150mg:Elexa_teza_iva_50mg_100mg_150mg	1	No concerns	Low risk	No concerns	Major concerns	No concerns	No concerns	Low	[Imprecision]
Elexa_teza_iva_100mg_100mg_150mg:Placebo	1	No concerns	Low risk	No concerns	No concerns	No concerns	No concerns	High	NA
Elexa_teza_iva_200mg_100mg_150mg:Elexa_teza_iva_50mg_100mg_150mg	1	No concerns	Low risk	No concerns	Major concerns	No concerns	No concerns	Low	[Imprecision]
Elexa_teza_iva_200mg_100mg_150mg:Iva_150mg	1	No concerns	Low risk	No concerns	No concerns	No concerns	No concerns	High	NA
Elexa_teza_iva_200mg_100mg_150mg:Placebo	2	No concerns	Low risk	No concerns	No concerns	No concerns	No concerns	High	NA
Elexa_teza_iva_200mg_100mg_150mg:Teza_iva_100mg_150mg	3	No concerns	Low risk	No concerns	No concerns	No concerns	No concerns	High	NA
Elexa_teza_iva_50mg_100mg_150mg:Placebo	1	No concerns	Low risk	No concerns	No concerns	No concerns	No concerns	High	NA
GLPG2222_100mg:GLPG2222_200mg	1	No concerns	Low risk	No concerns	Major concerns	No concerns	No concerns	Low	[Imprecision]
GLPG2222_100mg:GLPG2222_400mg	1	No concerns	Low risk	No concerns	Major concerns	No concerns	No concerns	Low	[Imprecision]
GLPG2222_100mg:GLPG2222_50mg	1	No concerns	Low risk	No concerns	Major concerns	No concerns	No concerns	Low	[Imprecision]
GLPG2222_100mg:Placebo	1	No concerns	Low risk	No concerns	Major concerns	No concerns	No concerns	Low	[Imprecision]
GLPG2222_150mg:GLPG2222_300mg	1	No concerns	Low risk	No concerns	Major concerns	No concerns	No concerns	Low	[Imprecision]
GLPG2222_150mg:Placebo	1	No concerns	Low risk	No concerns	Major concerns	No concerns	No concerns	Low	[Imprecision]
GLPG2222_200mg:GLPG2222_400mg	1	No concerns	Low risk	No concerns	Major concerns	No concerns	No concerns	Low	[Imprecision]
GLPG2222_200mg:GLPG2222_50mg	1	No concerns	Low risk	No concerns	Major concerns	No concerns	No concerns	Low	[Imprecision]

GLPG2222_200mg:Placebo	1	No concerns	Low risk	No concerns	Major concerns	No concerns	No concerns	Low	[Imprecision]
GLPG2222_300mg:Placebo	1	No concerns	Low risk	No concerns	Major concerns	No concerns	No concerns	Low	[Imprecision]
GLPG2222_400mg:GLPG2222_50mg	1	No concerns	Low risk	No concerns	Major concerns	No concerns	No concerns	Low	[Imprecision]
GLPG2222_400mg:Placebo	1	No concerns	Low risk	No concerns	Major concerns	No concerns	No concerns	Low	[Imprecision]
GLPG2222_50mg:Placebo	1	No concerns	Low risk	No concerns	Major concerns	No concerns	No concerns	Low	[Imprecision]
GLPG2737_75mg:Placebo	1	No concerns	Low risk	No concerns	Major concerns	No concerns	No concerns	Low	[Imprecision]
Iva_150mg:Teza_iva_100mg_150mg	1	No concerns	Low risk	No concerns	Major concerns	No concerns	No concerns	Low	[Imprecision]
Ola_teza_iva_600mg_50mg_300mg:Placebo	1	No concerns	Low risk	No concerns	No concerns	No concerns	No concerns	High	NA
Ola_teza_iva_600mg_50mg_300mg:Teza_iva_50mg_300mg	1	No concerns	Low risk	No concerns	No concerns	No concerns	No concerns	High	NA
Placebo:Teza_iva_100mg_150mg	2	No concerns	Low risk	No concerns	No concerns	No concerns	No concerns	High	NA
Placebo:VX152_teza_iva_100mg_100mg_150 mg	1	No concerns	Low risk	No concerns	Major concerns	No concerns	No concerns	Low	[Imprecision]
Placebo:VX152_teza_iva_200mg_100mg_150 mg	1	No concerns	Low risk	No concerns	No concerns	No concerns	Major concerns	Low	[Incoherence]
Placebo:VX152_teza_iva_300mg_100mg_150 mg	1	No concerns	Low risk	No concerns	No concerns	No concerns	No concerns	High	NA
luma_100mg:Placebo	1	Some concerns	Low risk	No concerns	Major concerns	No concerns	No concerns	Low	[Within-study bias, Imprecision]
luma_200mg:Placebo	1	Some concerns	Low risk	No concerns	Major concerns	No concerns	No concerns	Low	[Within-study bias|Imprecision]
luma_25mg:Placebo	1	Some concerns	Low risk	No concerns	Major concerns	No concerns	No concerns	Low	[Within-study bias|Imprecision]
luma_50mg:Placebo	1	Some concerns	Low risk	No concerns	No concerns	Major concerns	No concerns	Low	[Within-study bias|Heterogeneity]
luma_iva_200mg_250mg:Placebo	1	No concerns	Low risk	No concerns	No concerns	No concerns	No concerns	High	NA
luma_iva_400mg_250mg:Placebo	2	Some concerns	Low risk	No concerns	No concerns	Major concerns	No concerns	Low	[Within-study bias|Heterogeneity]

luma_iva_600mg_250mg:Placebo	1	No concerns	Low risk	No concerns	Major concerns	No concerns	No concerns	Low	[Imprecision]
Placebo:vanza_teza_deuti_10mg_100mg_150mg	1	No concerns	Low risk	No concerns	No concerns	No concerns	No concerns	High	NA
Placebo:vanza_teza_deuti_20mg_100mg_150mg	1	No concerns	Low risk	No concerns	No concerns	No concerns	No concerns	High	NA
Placebo:vanza_teza_deuti_5mg_100mg_150mg	1	No concerns	Low risk	No concerns	Major concerns	No concerns	No concerns	Low	[Imprecision]
Teza_iva_100mg_150mg:VX152_teza_iva_200mg_100mg_150mg	1	No concerns	Low risk	No concerns	No concerns	No concerns	No concerns	High	NA
Teza_iva_100mg_150mg:VX152_teza_iva_300mg_100mg_150mg	1	No concerns	Low risk	No concerns	No concerns	No concerns	No concerns	High	NA
Teza_iva_100mg_150mg:vanza_teza_deuti_20mg_100mg_150mg	1	No concerns	Low risk	No concerns	No concerns	No concerns	No concerns	High	NA
VX152_teza_iva_100mg_100mg_150mg:VX152_teza_iva_200mg_100mg_150mg	1	No concerns	Low risk	No concerns	Major concerns	No concerns	No concerns	Low	[Imprecision]
VX152_teza_iva_100mg_100mg_150mg:VX152_teza_iva_300mg_100mg_150mg	1	No concerns	Low risk	No concerns	Major concerns	No concerns	No concerns	Low	[Imprecision]
VX152_teza_iva_200mg_100mg_150mg:VX152_teza_iva_300mg_100mg_150mg	1	No concerns	Low risk	No concerns	Major concerns	No concerns	No concerns	Low	[Imprecision]
luma_100mg:luma_200mg	1	Some concerns	Low risk	No concerns	Major concerns	No concerns	No concerns	Low	[Within-study bias|Imprecision]
luma_100mg:luma_25mg	1	Some concerns	Low risk	No concerns	Major concerns	No concerns	No concerns	Low	[Within-study bias|Imprecision]
luma_100mg:luma_50mg	1	Some concerns	Low risk	No concerns	Major concerns	No concerns	No concerns	Low	[Within-study bias|Imprecision]
luma_200mg:luma_25mg	1	Some concerns	Low risk	No concerns	Major concerns	No concerns	No concerns	Low	[Within-study bias|Imprecision]
luma_200mg:luma_50mg	1	Some concerns	Low risk	No concerns	Major concerns	No concerns	No concerns	Low	[Within-study bias|Imprecision]
luma_25mg:luma_50mg	1	Some concerns	Low risk	No concerns	Major concerns	No concerns	No concerns	Low	[Within-study bias|Imprecision]
luma_iva_200mg_250mg:luma_iva_400mg_250mg	1	No concerns	Low risk	No concerns	Major concerns	No concerns	No concerns	Low	[Imprecision]
luma_iva_200mg_250mg:luma_iva_600mg_250mg	1	No concerns	Low risk	No concerns	Major concerns	No concerns	No concerns	Low	[Imprecision]

luma_iva_400mg_250mg:luma_iva_600mg_250mg	1	No concerns	Low risk	No concerns	Major concerns	No concerns	No concerns	Low	[Imprecision]
vanza_teza_deuti_10mg_100mg_150mg:vanza_teza_deuti_20mg_100mg_150mg	1	No concerns	Low risk	No concerns	Major concerns	No concerns	No concerns	Low	[Imprecision]
vanza_teza_deuti_10mg_100mg_150mg:vanza_teza_deuti_5mg_100mg_150mg	1	No concerns	Low risk	No concerns	Major concerns	No concerns	No concerns	Low	[Imprecision]
vanza_teza_deuti_20mg_100mg_150mg:vanza_teza_deuti_5mg_100mg_150mg	1	No concerns	Low risk	No concerns	Major concerns	No concerns	No concerns	Low	[Imprecision]
Indirect evidence									
Elexa_teza_iva_100mg_100mg_150mg:GLPG2222_100mg	0	No concerns	Low risk	No concerns	Major concerns	No concerns	No concerns	Low	[Imprecision]
Elexa_teza_iva_100mg_100mg_150mg:GLPG2222_150mg	0	No concerns	Low risk	No concerns	Major concerns	No concerns	No concerns	Low	[Imprecision]
Elexa_teza_iva_100mg_100mg_150mg:GLPG2222_200mg	0	No concerns	Low risk	No concerns	Major concerns	No concerns	No concerns	Low	[Imprecision]
Elexa_teza_iva_100mg_100mg_150mg:GLPG2222_300mg	0	No concerns	Low risk	No concerns	Major concerns	No concerns	No concerns	Low	[Imprecision]
Elexa_teza_iva_100mg_100mg_150mg:GLPG2222_400mg	0	No concerns	Low risk	No concerns	Major concerns	No concerns	No concerns	Low	[Imprecision]
Elexa_teza_iva_100mg_100mg_150mg:GLPG2222_50mg	0	No concerns	Low risk	No concerns	Major concerns	No concerns	No concerns	Low	[Imprecision]
Elexa_teza_iva_100mg_100mg_150mg:GLPG2737_75mg	0	No concerns	Low risk	No concerns	Major concerns	No concerns	No concerns	Low	[Imprecision]
Elexa_teza_iva_100mg_100mg_150mg:Iva_150mg	0	No concerns	Low risk	No concerns	Major concerns	No concerns	No concerns	Low	[Imprecision]
Elexa_teza_iva_100mg_100mg_150mg:Ola_teza_iva_600mg_50mg_300mg	0	No concerns	Low risk	No concerns	Major concerns	No concerns	No concerns	Low	[Imprecision]
Elexa_teza_iva_100mg_100mg_150mg:Teza_iva_100mg_150mg	0	No concerns	Low risk	No concerns	Major concerns	No concerns	No concerns	Low	[Imprecision]
Elexa_teza_iva_100mg_100mg_150mg:Teza_iva_50mg_300mg	0	No concerns	Low risk	No concerns	Major concerns	No concerns	No concerns	Low	[Imprecision]
Elexa_teza_iva_100mg_100mg_150mg:VX152_teza_iva_100mg_100mg_150mg	0	No concerns	Low risk	No concerns	Major concerns	No concerns	No concerns	Low	[Imprecision]
Elexa_teza_iva_100mg_100mg_150mg:VX152_teza_iva_200mg_100mg_150mg	0	No concerns	Low risk	No concerns	Major concerns	No concerns	No concerns	Low	[Imprecision]
Elexa_teza_iva_100mg_100mg_150mg:VX152_teza_iva_300mg_100mg_150mg	0	No concerns	Low risk	No concerns	Major concerns	No concerns	No concerns	Low	[Imprecision]

Elexa_teza_iva_100mg_100mg_150mg:luma_100mg	0	No concerns	Low risk	No concerns	No concerns	No concerns	No concerns	High	NA
Elexa_teza_iva_100mg_100mg_150mg:luma_200mg	0	No concerns	Low risk	No concerns	No concerns	Major concerns	No concerns	Low	[Heterogeneity]
Elexa_teza_iva_100mg_100mg_150mg:luma_25mg	0	No concerns	Low risk	No concerns	No concerns	No concerns	No concerns	High	NA
Elexa_teza_iva_100mg_100mg_150mg:luma_50mg	0	No concerns	Low risk	No concerns	No concerns	No concerns	No concerns	High	NA
Elexa_teza_iva_100mg_100mg_150mg:luma_iva_200mg_250mg	0	No concerns	Low risk	No concerns	Major concerns	No concerns	No concerns	Low	[Imprecision]
Elexa_teza_iva_100mg_100mg_150mg:luma_iva_400mg_250mg	0	No concerns	Low risk	No concerns	Major concerns	No concerns	No concerns	Low	[Imprecision]
Elexa_teza_iva_100mg_100mg_150mg:luma_iva_600mg_250mg	0	No concerns	Low risk	No concerns	Major concerns	No concerns	No concerns	Low	[Imprecision]
Elexa_teza_iva_100mg_100mg_150mg:vanza_teza_deuti_10mg_100mg_150mg	0	No concerns	Low risk	No concerns	Major concerns	No concerns	No concerns	Low	[Imprecision]
Elexa_teza_iva_100mg_100mg_150mg:vanza_teza_deuti_20mg_100mg_150mg	0	No concerns	Low risk	No concerns	No concerns	No concerns	No concerns	High	NA
Elexa_teza_iva_100mg_100mg_150mg:vanza_teza_deuti_5mg_100mg_150mg	0	No concerns	Low risk	No concerns	Major concerns	No concerns	No concerns	Low	[Imprecision]
Elexa_teza_iva_200mg_100mg_150mg:GLPG2222_100mg	0	No concerns	Low risk	No concerns	No concerns	No concerns	No concerns	High	NA
Elexa_teza_iva_200mg_100mg_150mg:GLPG2222_150mg	0	No concerns	Low risk	No concerns	No concerns	No concerns	No concerns	High	NA
Elexa_teza_iva_200mg_100mg_150mg:GLPG2222_200mg	0	No concerns	Low risk	No concerns	No concerns	Major concerns	No concerns	Low	[Heterogeneity]
Elexa_teza_iva_200mg_100mg_150mg:GLPG2222_300mg	0	No concerns	Low risk	No concerns	No concerns	No concerns	No concerns	High	NA
Elexa_teza_iva_200mg_100mg_150mg:GLPG2222_400mg	0	No concerns	Low risk	No concerns	No concerns	No concerns	No concerns	High	NA
Elexa_teza_iva_200mg_100mg_150mg:GLPG2222_50mg	0	No concerns	Low risk	No concerns	No concerns	No concerns	No concerns	High	NA
Elexa_teza_iva_200mg_100mg_150mg:GLPG2737_75mg	0	No concerns	Low risk	No concerns	No concerns	No concerns	No concerns	High	NA
Elexa_teza_iva_200mg_100mg_150mg:Ola_teza_iva_600mg_50mg_300mg	0	No concerns	Low risk	No concerns	Major concerns	No concerns	No concerns	Low	[Imprecision]
Elexa_teza_iva_200mg_100mg_150mg:Teza_iva_50mg_300mg	0	No concerns	Low risk	No concerns	No concerns	No concerns	No concerns	High	NA

Elexa_teza_iva_200mg_100mg_150mg:VX152_teza_iva_100mg_100mg_150mg	0	No concerns	Low risk	No concerns	Major concerns	No concerns	No concerns	Low	[Imprecision]
Elexa_teza_iva_200mg_100mg_150mg:VX152_teza_iva_200mg_100mg_150mg	0	No concerns	Low risk	No concerns	Major concerns	No concerns	No concerns	Low	[Imprecision]
Elexa_teza_iva_200mg_100mg_150mg:VX152_teza_iva_300mg_100mg_150mg	0	No concerns	Low risk	No concerns	Major concerns	No concerns	No concerns	Low	[Imprecision]
Elexa_teza_iva_200mg_100mg_150mg:luma_100mg	0	Some concerns	Low risk	No concerns	No concerns	No concerns	No concerns	Moderate	[Within-study bias]
Elexa_teza_iva_200mg_100mg_150mg:luma_200mg	0	Some concerns	Low risk	No concerns	No concerns	No concerns	No concerns	Moderate	[Within-study bias]
Elexa_teza_iva_200mg_100mg_150mg:luma_25mg	0	Some concerns	Low risk	No concerns	No concerns	No concerns	No concerns	Moderate	[Within-study bias]
Elexa_teza_iva_200mg_100mg_150mg:luma_50mg	0	Some concerns	Low risk	No concerns	No concerns	No concerns	No concerns	Moderate	[Within-study bias]
Elexa_teza_iva_200mg_100mg_150mg:luma_iva_200mg_250mg	0	No concerns	Low risk	No concerns	Major concerns	No concerns	No concerns	Low	[Imprecision]
Elexa_teza_iva_200mg_100mg_150mg:luma_iva_400mg_250mg	0	No concerns	Low risk	No concerns	No concerns	No concerns	No concerns	High	NA
Elexa_teza_iva_200mg_100mg_150mg:luma_iva_600mg_250mg	0	No concerns	Low risk	No concerns	No concerns	No concerns	No concerns	High	NA
Elexa_teza_iva_200mg_100mg_150mg:vanza_teza_deuti_10mg_100mg_150mg	0	No concerns	Low risk	No concerns	Major concerns	No concerns	No concerns	Low	[Imprecision]
Elexa_teza_iva_200mg_100mg_150mg:vanza_teza_deuti_20mg_100mg_150mg	0	No concerns	Low risk	No concerns	Major concerns	No concerns	No concerns	Low	[Imprecision]
Elexa_teza_iva_200mg_100mg_150mg:vanza_teza_deuti_5mg_100mg_150mg	0	No concerns	Low risk	No concerns	Major concerns	No concerns	No concerns	Low	[Imprecision]
Elexa_teza_iva_50mg_100mg_150mg:GLPG2222_100mg	0	No concerns	Low risk	No concerns	Major concerns	No concerns	No concerns	Low	[Imprecision]
Elexa_teza_iva_50mg_100mg_150mg:GLPG2222_150mg	0	No concerns	Low risk	No concerns	No concerns	No concerns	No concerns	High	NA
Elexa_teza_iva_50mg_100mg_150mg:GLPG2222_200mg	0	No concerns	Low risk	No concerns	Major concerns	No concerns	No concerns	Low	[Imprecision]
Elexa_teza_iva_50mg_100mg_150mg:GLPG2222_300mg	0	No concerns	Low risk	No concerns	No concerns	No concerns	No concerns	High	NA
Elexa_teza_iva_50mg_100mg_150mg:GLPG2222_400mg	0	No concerns	Low risk	No concerns	No concerns	Major concerns	No concerns	Low	[Heterogeneity]
Elexa_teza_iva_50mg_100mg_150mg:GLPG2222_50mg	0	No concerns	Low risk	No concerns	Major concerns	No concerns	No concerns	Low	[Imprecision]

Elexa_teza_iva_50mg_100mg_150mg:GLPG2737_75mg	0	No concerns	Low risk	No concerns	No concerns	Major concerns	No concerns	Low	[Heterogeneity]
Elexa_teza_iva_50mg_100mg_150mg:Iva_150mg	0	No concerns	Low risk	No concerns	No concerns	No concerns	No concerns	High	NA
Elexa_teza_iva_50mg_100mg_150mg:Ola_teza_iva_600mg_50mg_300mg	0	No concerns	Low risk	No concerns	Major concerns	No concerns	No concerns	Low	[Imprecision]
Elexa_teza_iva_50mg_100mg_150mg:Teza_iva_100mg_150mg	0	No concerns	Low risk	No concerns	No concerns	No concerns	No concerns	High	NA
Elexa_teza_iva_50mg_100mg_150mg:Teza_iva_50mg_300mg	0	No concerns	Low risk	No concerns	No concerns	Major concerns	No concerns	Low	[Heterogeneity]
Elexa_teza_iva_50mg_100mg_150mg:VX152_teza_iva_100mg_100mg_150mg	0	No concerns	Low risk	No concerns	Major concerns	No concerns	No concerns	Low	[Imprecision]
Elexa_teza_iva_50mg_100mg_150mg:VX152_teza_iva_200mg_100mg_150mg	0	No concerns	Low risk	No concerns	Major concerns	No concerns	No concerns	Low	[Imprecision]
Elexa_teza_iva_50mg_100mg_150mg:VX152_teza_iva_300mg_100mg_150mg	0	No concerns	Low risk	No concerns	Major concerns	No concerns	No concerns	Low	[Imprecision]
Elexa_teza_iva_50mg_100mg_150mg:luma_100mg	0	No concerns	Low risk	No concerns	No concerns	No concerns	No concerns	High	NA
Elexa_teza_iva_50mg_100mg_150mg:luma_200mg	0	No concerns	Low risk	No concerns	No concerns	No concerns	No concerns	High	NA
Elexa_teza_iva_50mg_100mg_150mg:luma_25mg	0	No concerns	Low risk	No concerns	No concerns	No concerns	No concerns	High	NA
Elexa_teza_iva_50mg_100mg_150mg:luma_50mg	0	No concerns	Low risk	No concerns	No concerns	No concerns	No concerns	High	NA
Elexa_teza_iva_50mg_100mg_150mg:luma_iva_200mg_250mg	0	No concerns	Low risk	No concerns	Major concerns	No concerns	No concerns	Low	[Imprecision]
Elexa_teza_iva_50mg_100mg_150mg:luma_iva_400mg_250mg	0	No concerns	Low risk	No concerns	Major concerns	No concerns	No concerns	Low	[Imprecision]
Elexa_teza_iva_50mg_100mg_150mg:luma_iva_600mg_250mg	0	No concerns	Low risk	No concerns	Major concerns	No concerns	No concerns	Low	[Imprecision]
Elexa_teza_iva_50mg_100mg_150mg:vanza_teza_deuti_10mg_100mg_150mg	0	No concerns	Low risk	No concerns	Major concerns	No concerns	No concerns	Low	[Imprecision]
Elexa_teza_iva_50mg_100mg_150mg:vanza_teza_deuti_20mg_100mg_150mg	0	No concerns	Low risk	No concerns	No concerns	Major concerns	No concerns	Low	[Heterogeneity]
Elexa_teza_iva_50mg_100mg_150mg:vanza_teza_deuti_5mg_100mg_150mg	0	No concerns	Low risk	No concerns	Major concerns	No concerns	No concerns	Low	[Imprecision]
GLPG2222_100mg:GLPG2222_150mg	0	No concerns	Low risk	No concerns	Major concerns	No concerns	No concerns	Low	[Imprecision]

GLPG2222_100mg:GLPG2222_300mg	0	No concerns	Low risk	No concerns	Major concerns	No concerns	No concerns	Low	[Imprecision]
GLPG2222_100mg:GLPG2737_75mg	0	No concerns	Low risk	No concerns	Major concerns	No concerns	No concerns	Low	[Imprecision]
GLPG2222_100mg:Iva_150mg	0	No concerns	Low risk	No concerns	Major concerns	No concerns	No concerns	Low	[Imprecision]
GLPG2222_100mg:Ola_teza_iva_600mg_50mg_300mg	0	No concerns	Low risk	No concerns	Major concerns	No concerns	No concerns	Low	[Imprecision]
GLPG2222_100mg:Teza_iva_100mg_150mg	0	No concerns	Low risk	No concerns	Major concerns	No concerns	No concerns	Low	[Imprecision]
GLPG2222_100mg:Teza_iva_50mg_300mg	0	No concerns	Low risk	No concerns	Major concerns	No concerns	No concerns	Low	[Imprecision]
GLPG2222_100mg:VX152_teza_iva_100mg_100mg_150mg	0	No concerns	Low risk	No concerns	Major concerns	No concerns	No concerns	Low	[Imprecision]
GLPG2222_100mg:VX152_teza_iva_200mg_100mg_150mg	0	No concerns	Low risk	No concerns	No concerns	Major concerns	No concerns	Low	[Heterogeneity]
GLPG2222_100mg:VX152_teza_iva_300mg_100mg_150mg	0	No concerns	Low risk	No concerns	No concerns	Major concerns	No concerns	Low	[Heterogeneity]
GLPG2222_100mg:luma_100mg	0	Some concerns	Low risk	No concerns	Major concerns	No concerns	No concerns	Low	[Within-study bias|Imprecision]
GLPG2222_100mg:luma_200mg	0	No concerns	Low risk	No concerns	Major concerns	No concerns	No concerns	Low	[Imprecision]
GLPG2222_100mg:luma_25mg	0	No concerns	Low risk	No concerns	Major concerns	No concerns	No concerns	Low	[Imprecision]
GLPG2222_100mg:luma_50mg	0	No concerns	Low risk	No concerns	Major concerns	No concerns	No concerns	Low	[Imprecision]
GLPG2222_100mg:luma_iva_200mg_250mg	0	No concerns	Low risk	No concerns	Major concerns	No concerns	No concerns	Low	[Imprecision]
GLPG2222_100mg:luma_iva_400mg_250mg	0	No concerns	Low risk	No concerns	Major concerns	No concerns	No concerns	Low	[Imprecision]
GLPG2222_100mg:luma_iva_600mg_250mg	0	No concerns	Low risk	No concerns	Major concerns	No concerns	No concerns	Low	[Imprecision]
GLPG2222_100mg:vanza_teza_deuti_10mg_100mg_150mg	0	No concerns	Low risk	No concerns	Major concerns	No concerns	No concerns	Low	[Imprecision]
GLPG2222_100mg:vanza_teza_deuti_20mg_100mg_150mg	0	No concerns	Low risk	No concerns	No concerns	No concerns	No concerns	High	NA

GLPG2222_100mg:vanza_teza_deuti_5mg_100mg_150mg	0	No concerns	Low risk	No concerns	Major concerns	No concerns	No concerns	Low	[Imprecision]
GLPG2222_150mg:GLPG2222_200mg	0	No concerns	Low risk	No concerns	Major concerns	No concerns	No concerns	Low	[Imprecision]
GLPG2222_150mg:GLPG2222_400mg	0	No concerns	Low risk	No concerns	Major concerns	No concerns	No concerns	Low	[Imprecision]
GLPG2222_150mg:GLPG2222_50mg	0	No concerns	Low risk	No concerns	Major concerns	No concerns	No concerns	Low	[Imprecision]
GLPG2222_150mg:GLPG2737_75mg	0	No concerns	Low risk	No concerns	Major concerns	No concerns	No concerns	Low	[Imprecision]
GLPG2222_150mg:Iva_150mg	0	No concerns	Low risk	No concerns	Major concerns	No concerns	No concerns	Low	[Imprecision]
GLPG2222_150mg:Ola_teza_iva_600mg_50mg_300mg	0	No concerns	Low risk	No concerns	No concerns	No concerns	No concerns	High	NA
GLPG2222_150mg:Teza_iva_100mg_150mg	0	No concerns	Low risk	No concerns	Major concerns	No concerns	No concerns	Low	[Imprecision]
GLPG2222_150mg:Teza_iva_50mg_300mg	0	No concerns	Low risk	No concerns	Major concerns	No concerns	No concerns	Low	[Imprecision]
GLPG2222_150mg:VX152_teza_iva_100mg_100mg_150mg	0	No concerns	Low risk	No concerns	Major concerns	No concerns	No concerns	Low	[Imprecision]
GLPG2222_150mg:VX152_teza_iva_200mg_100mg_150mg	0	No concerns	Low risk	No concerns	No concerns	No concerns	No concerns	High	NA
GLPG2222_150mg:VX152_teza_iva_300mg_100mg_150mg	0	No concerns	Low risk	No concerns	No concerns	No concerns	No concerns	High	NA
GLPG2222_150mg:luma_100mg	0	Some concerns	Low risk	No concerns	Major concerns	No concerns	No concerns	Low	[Within-study bias|Imprecision]
GLPG2222_150mg:luma_200mg	0	Some concerns	Low risk	No concerns	Major concerns	No concerns	No concerns	Low	[Within-study bias|Imprecision]
GLPG2222_150mg:luma_25mg	0	Some concerns	Low risk	No concerns	Major concerns	No concerns	No concerns	Low	[Within-study bias|Imprecision]
GLPG2222_150mg:luma_50mg	0	Some concerns	Low risk	No concerns	Major concerns	No concerns	No concerns	Low	[Within-study bias|Imprecision]
GLPG2222_150mg:luma_iva_200mg_250mg	0	No concerns	Low risk	No concerns	Major concerns	No concerns	No concerns	Low	[Imprecision]
GLPG2222_150mg:luma_iva_400mg_250mg	0	No concerns	Low risk	No concerns	Major concerns	No concerns	No concerns	Low	[Imprecision]

GLPG2222_150mg:luma_iva_600mg_250mg	0	No concerns	Low risk	No concerns	Major concerns	No concerns	No concerns	Low	[Imprecision]
GLPG2222_150mg:vanza_teza_deuti_10mg_100mg_150mg	0	No concerns	Low risk	No concerns	No concerns	Major concerns	No concerns	Low	[Heterogeneity]
GLPG2222_150mg:vanza_teza_deuti_20mg_100mg_150mg	0	No concerns	Low risk	No concerns	No concerns	No concerns	No concerns	High	NA
GLPG2222_150mg:vanza_teza_deuti_5mg_100mg_150mg	0	No concerns	Low risk	No concerns	Major concerns	No concerns	No concerns	Low	[Imprecision]
GLPG2222_200mg:GLPG2222_300mg	0	No concerns	Low risk	No concerns	Major concerns	No concerns	No concerns	Low	[Imprecision]
GLPG2222_200mg:GLPG2737_75mg	0	No concerns	Low risk	No concerns	Major concerns	No concerns	No concerns	Low	[Imprecision]
GLPG2222_200mg:Iva_150mg	0	No concerns	Low risk	No concerns	Major concerns	No concerns	No concerns	Low	[Imprecision]
GLPG2222_200mg:Ola_teza_iva_600mg_50mg_300mg	0	No concerns	Low risk	No concerns	Major concerns	No concerns	No concerns	Low	[Imprecision]
GLPG2222_200mg:Teza_iva_100mg_150mg	0	No concerns	Low risk	No concerns	Major concerns	No concerns	No concerns	Low	[Imprecision]
GLPG2222_200mg:Teza_iva_50mg_300mg	0	No concerns	Low risk	No concerns	Major concerns	No concerns	No concerns	Low	[Imprecision]
GLPG2222_200mg:VX152_teza_iva_100mg_100mg_150mg	0	No concerns	Low risk	No concerns	Major concerns	No concerns	No concerns	Low	[Imprecision]
GLPG2222_200mg:VX152_teza_iva_200mg_100mg_150mg	0	No concerns	Low risk	No concerns	Major concerns	No concerns	No concerns	Low	[Imprecision]
GLPG2222_200mg:VX152_teza_iva_300mg_100mg_150mg	0	No concerns	Low risk	No concerns	Major concerns	No concerns	No concerns	Low	[Imprecision]
GLPG2222_200mg:luma_100mg	0	Some concerns	Low risk	No concerns	No concerns	Major concerns	No concerns	Low	[Within-study bias|Heterogeneity]
GLPG2222_200mg:luma_200mg	0	Some concerns	Low risk	No concerns	Major concerns	No concerns	No concerns	Low	[Within-study bias|Imprecision]
GLPG2222_200mg:luma_25mg	0	Some concerns	Low risk	No concerns	No concerns	No concerns	No concerns	Moderate	[Within-study bias]
GLPG2222_200mg:luma_50mg	0	Some concerns	Low risk	No concerns	No concerns	No concerns	No concerns	Moderate	[Within-study bias]
GLPG2222_200mg:luma_iva_200mg_250mg	0	No concerns	Low risk	No concerns	Major concerns	No concerns	No concerns	Low	[Imprecision]

GLPG2222_200mg:luma_iva_400mg_250mg	0	No concerns	Low risk	No concerns	Major concerns	No concerns	No concerns	Low	[Imprecision]
GLPG2222_200mg:luma_iva_600mg_250mg	0	No concerns	Low risk	No concerns	Major concerns	No concerns	No concerns	Low	[Imprecision]
GLPG2222_200mg:vanza_teza_deuti_10mg_100mg_150mg	0	No concerns	Low risk	No concerns	Major concerns	No concerns	No concerns	Low	[Imprecision]
GLPG2222_200mg:vanza_teza_deuti_20mg_100mg_150mg	0	No concerns	Low risk	No concerns	No concerns	No concerns	No concerns	High	NA
GLPG2222_200mg:vanza_teza_deuti_5mg_100mg_150mg	0	No concerns	Low risk	No concerns	Major concerns	No concerns	No concerns	Low	[Imprecision]
GLPG2222_300mg:GLPG2222_400mg	0	No concerns	Low risk	No concerns	Major concerns	No concerns	No concerns	Low	[Imprecision]
GLPG2222_300mg:GLPG2222_50mg	0	No concerns	Low risk	No concerns	Major concerns	No concerns	No concerns	Low	[Imprecision]
GLPG2222_300mg:GLPG2737_75mg	0	No concerns	Low risk	No concerns	Major concerns	No concerns	No concerns	Low	[Imprecision]
GLPG2222_300mg:Iva_150mg	0	No concerns	Low risk	No concerns	Major concerns	No concerns	No concerns	Low	[Imprecision]
GLPG2222_300mg:Ola_teza_iva_600mg_50mg_300mg	0	No concerns	Low risk	No concerns	No concerns	No concerns	No concerns	High	NA
GLPG2222_300mg:Teza_iva_100mg_150mg	0	No concerns	Low risk	No concerns	Major concerns	No concerns	No concerns	Low	[Imprecision]
GLPG2222_300mg:Teza_iva_50mg_300mg	0	No concerns	Low risk	No concerns	Major concerns	No concerns	No concerns	Low	[Imprecision]
GLPG2222_300mg:VX152_teza_iva_100mg_100mg_150mg	0	No concerns	Low risk	No concerns	Major concerns	No concerns	No concerns	Low	[Imprecision]
GLPG2222_300mg:VX152_teza_iva_200mg_100mg_150mg	0	No concerns	Low risk	No concerns	No concerns	No concerns	No concerns	High	NA
GLPG2222_300mg:VX152_teza_iva_300mg_100mg_150mg	0	No concerns	Low risk	No concerns	No concerns	No concerns	No concerns	High	NA
GLPG2222_300mg:luma_100mg	0	Some concerns	Low risk	No concerns	Major concerns	No concerns	No concerns	Low	[Within-study bias|Imprecision]
GLPG2222_300mg:luma_200mg	0	Some concerns	Low risk	No concerns	Major concerns	No concerns	No concerns	Low	[Within-study bias|Imprecision]
GLPG2222_300mg:luma_25mg	0	Some concerns	Low risk	No concerns	Major concerns	No concerns	No concerns	Low	[Within-study bias|Imprecision]

GLPG2222_300mg:luma_50mg	0	Some concerns	Low risk	No concerns	Major concerns	No concerns	No concerns	Low	[Within-study bias|Imprecision]
GLPG2222_300mg:luma_iva_200mg_250mg	0	No concerns	Low risk	No concerns	Major concerns	No concerns	No concerns	Low	[Imprecision]
GLPG2222_300mg:luma_iva_400mg_250mg	0	No concerns	Low risk	No concerns	Major concerns	No concerns	No concerns	Low	[Imprecision]
GLPG2222_300mg:luma_iva_600mg_250mg	0	No concerns	Low risk	No concerns	Major concerns	No concerns	No concerns	Low	[Imprecision]
GLPG2222_300mg:vanza_teza_deuti_10mg_100mg_150mg	0	No concerns	Low risk	No concerns	No concerns	Major concerns	No concerns	Low	[Heterogeneity]
GLPG2222_300mg:vanza_teza_deuti_20mg_100mg_150mg	0	No concerns	Low risk	No concerns	No concerns	No concerns	No concerns	High	NA
GLPG2222_300mg:vanza_teza_deuti_5mg_100mg_150mg	0	No concerns	Low risk	No concerns	Major concerns	No concerns	No concerns	Low	[Imprecision]
GLPG2222_400mg:GLPG2737_75mg	0	No concerns	Low risk	No concerns	Major concerns	No concerns	No concerns	Low	[Imprecision]
GLPG2222_400mg:Iva_150mg	0	No concerns	Low risk	No concerns	Major concerns	No concerns	No concerns	Low	[Imprecision]
GLPG2222_400mg:Ola_teza_iva_600mg_50mg_300mg	0	No concerns	Low risk	No concerns	No concerns	Major concerns	No concerns	Low	[Heterogeneity]
GLPG2222_400mg:Teza_iva_100mg_150mg	0	No concerns	Low risk	No concerns	Major concerns	No concerns	No concerns	Low	[Imprecision]
GLPG2222_400mg:Teza_iva_50mg_300mg	0	No concerns	Low risk	No concerns	Major concerns	No concerns	No concerns	Low	[Imprecision]
GLPG2222_400mg:VX152_teza_iva_100mg_100mg_150mg	0	No concerns	Low risk	No concerns	Major concerns	No concerns	No concerns	Low	[Imprecision]
GLPG2222_400mg:VX152_teza_iva_200mg_100mg_150mg	0	No concerns	Low risk	No concerns	No concerns	Major concerns	No concerns	Low	[Heterogeneity]
GLPG2222_400mg:VX152_teza_iva_300mg_100mg_150mg	0	No concerns	Low risk	No concerns	No concerns	Major concerns	No concerns	Low	[Heterogeneity]
GLPG2222_400mg:luma_100mg	0	Some concerns	Low risk	No concerns	Major concerns	No concerns	No concerns	Low	[Within-study bias|Imprecision]
GLPG2222_400mg:luma_200mg	0	Some concerns	Low risk	No concerns	Major concerns	No concerns	No concerns	Low	[Within-study bias|Imprecision]
GLPG2222_400mg:luma_25mg	0	Some concerns	Low risk	No concerns	Major concerns	No concerns	No concerns	Low	[Within-study bias|Imprecision]

GLPG2222_400mg:luma_50mg	0	Some concerns	Low risk	No concerns	Major concerns	No concerns	No concerns	Low	[Within-study bias|Imprecision]
GLPG2222_400mg:luma_iva_200mg_250mg	0	No concerns	Low risk	No concerns	Major concerns	No concerns	No concerns	Low	[Imprecision]
GLPG2222_400mg:luma_iva_400mg_250mg	0	No concerns	Low risk	No concerns	Major concerns	No concerns	No concerns	Low	[Imprecision]
GLPG2222_400mg:luma_iva_600mg_250mg	0	No concerns	Low risk	No concerns	Major concerns	No concerns	No concerns	Low	[Imprecision]
GLPG2222_400mg:vanza_teza_deuti_10mg_100mg_150mg	0	No concerns	Low risk	No concerns	Major concerns	No concerns	No concerns	Low	[Imprecision]
GLPG2222_400mg:vanza_teza_deuti_20mg_100mg_150mg	0	No concerns	Low risk	No concerns	No concerns	No concerns	No concerns	High	NA
GLPG2222_400mg:vanza_teza_deuti_5mg_100mg_150mg	0	No concerns	Low risk	No concerns	Major concerns	No concerns	No concerns	Low	[Imprecision]
GLPG2222_50mg:GLPG2737_75mg	0	No concerns	Low risk	No concerns	Major concerns	No concerns	No concerns	Low	[Imprecision]
GLPG2222_50mg:Iva_150mg	0	No concerns	Low risk	No concerns	Major concerns	No concerns	No concerns	Low	[Imprecision]
GLPG2222_50mg:Ola_teza_iva_600mg_50mg_300mg	0	No concerns	Low risk	No concerns	Major concerns	No concerns	No concerns	Low	[Imprecision]
GLPG2222_50mg:Teza_iva_100mg_150mg	0	No concerns	Low risk	No concerns	Major concerns	No concerns	No concerns	Low	[Imprecision]
GLPG2222_50mg:Teza_iva_50mg_300mg	0	No concerns	Low risk	No concerns	Major concerns	No concerns	No concerns	Low	[Imprecision]
GLPG2222_50mg:VX152_teza_iva_100mg_100mg_150mg	0	No concerns	Low risk	No concerns	Major concerns	No concerns	No concerns	Low	[Imprecision]
GLPG2222_50mg:VX152_teza_iva_200mg_100mg_150mg	0	No concerns	Low risk	No concerns	No concerns	Major concerns	No concerns	Low	[Heterogeneity]
GLPG2222_50mg:VX152_teza_iva_300mg_100mg_150mg	0	No concerns	Low risk	No concerns	Major concerns	No concerns	No concerns	Low	[Imprecision]
GLPG2222_50mg:luma_100mg	0	No concerns	Low risk	No concerns	Major concerns	No concerns	No concerns	Low	[Imprecision]
GLPG2222_50mg:luma_200mg	0	No concerns	Low risk	No concerns	Major concerns	No concerns	No concerns	Low	[Imprecision]
GLPG2222_50mg:luma_25mg	0	No concerns	Low risk	No concerns	Major concerns	No concerns	No concerns	Low	[Imprecision]

GLPG2222_50mg:luma_50mg	0	No concerns	Low risk	No concerns	Major concerns	No concerns	No concerns	Low	[Imprecision]
GLPG2222_50mg:luma_iva_200mg_250mg	0	No concerns	Low risk	No concerns	Major concerns	No concerns	No concerns	Low	[Imprecision]
GLPG2222_50mg:luma_iva_400mg_250mg	0	No concerns	Low risk	No concerns	Major concerns	No concerns	No concerns	Low	[Imprecision]
GLPG2222_50mg:luma_iva_600mg_250mg	0	No concerns	Low risk	No concerns	Major concerns	No concerns	No concerns	Low	[Imprecision]
GLPG2222_50mg:vanza_teza_deuti_10mg_100 mg_150mg	0	No concerns	Low risk	No concerns	Major concerns	No concerns	No concerns	Low	[Imprecision]
GLPG2222_50mg:vanza_teza_deuti_20mg_100 mg_150mg	0	No concerns	Low risk	No concerns	No concerns	No concerns	No concerns	High	NA
GLPG2222_50mg:vanza_teza_deuti_5mg_100 mg_150mg	0	No concerns	Low risk	No concerns	Major concerns	No concerns	No concerns	Low	[Imprecision]
GLPG2737_75mg:Iva_150mg	0	No concerns	Low risk	No concerns	Major concerns	No concerns	No concerns	Low	[Imprecision]
GLPG2737_75mg:Ola_teza_iva_600mg_50mg _300mg	0	No concerns	Low risk	No concerns	No concerns	Major concerns	No concerns	Low	[Heterogeneity]
GLPG2737_75mg:Teza_iva_100mg_150mg	0	No concerns	Low risk	No concerns	Major concerns	No concerns	No concerns	Low	[Imprecision]
GLPG2737_75mg:Teza_iva_50mg_300mg	0	No concerns	Low risk	No concerns	Major concerns	No concerns	No concerns	Low	[Imprecision]
GLPG2737_75mg:VX152_teza_iva_100mg_10 0mg_150mg	0	No concerns	Low risk	No concerns	Major concerns	No concerns	No concerns	Low	[Imprecision]
GLPG2737_75mg:VX152_teza_iva_200mg_10 0mg_150mg	0	No concerns	Low risk	No concerns	No concerns	Major concerns	No concerns	Low	[Heterogeneity]
GLPG2737_75mg:VX152_teza_iva_300mg_10 0mg_150mg	0	No concerns	Low risk	No concerns	No concerns	Major concerns	No concerns	Low	[Heterogeneity]
GLPG2737_75mg:luma_100mg	0	Some concerns	Low risk	No concerns	Major concerns	No concerns	No concerns	Low	[Within-study bias|Imprecision]
GLPG2737_75mg:luma_200mg	0	Some concerns	Low risk	No concerns	Major concerns	No concerns	No concerns	Low	[Within-study bias|Imprecision]
GLPG2737_75mg:luma_25mg	0	Some concerns	Low risk	No concerns	Major concerns	No concerns	No concerns	Low	[Within-study bias|Imprecision]
GLPG2737_75mg:luma_50mg	0	Some concerns	Low risk	No concerns	Major concerns	No concerns	No concerns	Low	[Within-study bias|Imprecision]

GLPG2737_75mg:luma_iva_200mg_250mg	0	No concerns	Low risk	No concerns	Major concerns	No concerns	No concerns	Low	[Imprecision]
GLPG2737_75mg:luma_iva_400mg_250mg	0	No concerns	Low risk	No concerns	Major concerns	No concerns	No concerns	Low	[Imprecision]
GLPG2737_75mg:luma_iva_600mg_250mg	0	No concerns	Low risk	No concerns	Major concerns	No concerns	No concerns	Low	[Imprecision]
GLPG2737_75mg:vanza_teza_deuti_10mg_100mg_150mg	0	No concerns	Low risk	No concerns	Major concerns	No concerns	No concerns	Low	[Imprecision]
GLPG2737_75mg:vanza_teza_deuti_20mg_100mg_150mg	0	No concerns	Low risk	No concerns	No concerns	No concerns	No concerns	High	NA
GLPG2737_75mg:vanza_teza_deuti_5mg_100mg_150mg	0	No concerns	Low risk	No concerns	Major concerns	No concerns	No concerns	Low	[Imprecision]
Iva_150mg:Ola_teza_iva_600mg_50mg_300mg	0	No concerns	Low risk	No concerns	No concerns	Major concerns	No concerns	Low	[Heterogeneity]
Iva_150mg:Placebo	0	No concerns	Low risk	No concerns	Major concerns	No concerns	No concerns	Low	[Imprecision]
Iva_150mg:Teza_iva_50mg_300mg	0	No concerns	Low risk	No concerns	Major concerns	No concerns	No concerns	Low	[Imprecision]
Iva_150mg:VX152_teza_iva_100mg_100mg_150mg	0	No concerns	Low risk	No concerns	Major concerns	No concerns	No concerns	Low	[Imprecision]
Iva_150mg:VX152_teza_iva_200mg_100mg_150mg	0	No concerns	Low risk	No concerns	No concerns	No concerns	No concerns	High	NA
Iva_150mg:VX152_teza_iva_300mg_100mg_150mg	0	No concerns	Low risk	No concerns	No concerns	No concerns	No concerns	High	NA
Iva_150mg:luma_100mg	0	Some concerns	Low risk	No concerns	Major concerns	No concerns	No concerns	Low	[Within-study bias|Imprecision]
Iva_150mg:luma_200mg	0	Some concerns	Low risk	No concerns	Major concerns	No concerns	No concerns	Low	[Within-study bias|Imprecision]
Iva_150mg:luma_25mg	0	Some concerns	Low risk	No concerns	No concerns	No concerns	No concerns	Moderate	[Within-study bias]
Iva_150mg:luma_50mg	0	Some concerns	Low risk	No concerns	No concerns	No concerns	No concerns	Moderate	[Within-study bias]
Iva_150mg:luma_iva_200mg_250mg	0	No concerns	Low risk	No concerns	Major concerns	No concerns	No concerns	Low	[Imprecision]
Iva_150mg:luma_iva_400mg_250mg	0	No concerns	Low risk	No concerns	Major concerns	No concerns	No concerns	Low	[Imprecision]

Iva_150mg:luma_iva_600mg_250mg	0	No concerns	Low risk	No concerns	Major concerns	No concerns	No concerns	Low	[Imprecision]
Iva_150mg:vanza_teza_deuti_10mg_100mg_150mg	0	No concerns	Low risk	No concerns	Major concerns	No concerns	No concerns	Low	[Imprecision]
Iva_150mg:vanza_teza_deuti_20mg_100mg_150mg	0	No concerns	Low risk	No concerns	No concerns	No concerns	No concerns	High	NA
Iva_150mg:vanza_teza_deuti_5mg_100mg_150mg	0	No concerns	Low risk	No concerns	Major concerns	No concerns	No concerns	Low	[Imprecision]
Ola_teza_iva_600mg_50mg_300mg:Teza_iva_100mg_150mg	0	No concerns	Low risk	No concerns	No concerns	Major concerns	No concerns	Low	[Heterogeneity]
Ola_teza_iva_600mg_50mg_300mg:VX152_teza_iva_100mg_100mg_150mg	0	No concerns	Low risk	No concerns	Major concerns	No concerns	No concerns	Low	[Imprecision]
Ola_teza_iva_600mg_50mg_300mg:VX152_teza_iva_200mg_100mg_150mg	0	No concerns	Low risk	No concerns	Major concerns	No concerns	No concerns	Low	[Imprecision]
Ola_teza_iva_600mg_50mg_300mg:VX152_teza_iva_300mg_100mg_150mg	0	No concerns	Low risk	No concerns	Major concerns	No concerns	No concerns	Low	[Imprecision]
luma_100mg:Ola_teza_iva_600mg_50mg_300mg	0	Some concerns	Low risk	No concerns	No concerns	No concerns	No concerns	Moderate	[Within-study bias]
luma_200mg:Ola_teza_iva_600mg_50mg_300mg	0	Some concerns	Low risk	No concerns	No concerns	No concerns	No concerns	Moderate	[Within-study bias]
luma_25mg:Ola_teza_iva_600mg_50mg_300mg	0	Some concerns	Low risk	No concerns	No concerns	No concerns	No concerns	Moderate	[Within-study bias]
luma_50mg:Ola_teza_iva_600mg_50mg_300mg	0	Some concerns	Low risk	No concerns	No concerns	No concerns	No concerns	Moderate	[Within-study bias]
luma_iva_200mg_250mg:Ola_teza_iva_600mg_50mg_300mg	0	No concerns	Low risk	No concerns	Major concerns	No concerns	No concerns	Low	[Imprecision]
luma_iva_400mg_250mg:Ola_teza_iva_600mg_50mg_300mg	0	No concerns	Low risk	No concerns	Major concerns	No concerns	No concerns	Low	[Imprecision]
luma_iva_600mg_250mg:Ola_teza_iva_600mg_50mg_300mg	0	No concerns	Low risk	No concerns	Major concerns	No concerns	No concerns	Low	[Imprecision]
Ola_teza_iva_600mg_50mg_300mg:vanza_teza_deuti_10mg_100mg_150mg	0	No concerns	Low risk	No concerns	Major concerns	No concerns	No concerns	Low	[Imprecision]
Ola_teza_iva_600mg_50mg_300mg:vanza_teza_deuti_20mg_100mg_150mg	0	No concerns	Low risk	No concerns	Major concerns	No concerns	No concerns	Low	[Imprecision]
Ola_teza_iva_600mg_50mg_300mg:vanza_teza_deuti_5mg_100mg_150mg	0	No concerns	Low risk	No concerns	Major concerns	No concerns	No concerns	Low	[Imprecision]
Placebo:Teza_iva_50mg_300mg	0	No concerns	Low risk	No concerns	Major concerns	No concerns	No concerns	Low	[Imprecision]

Teza_iva_100mg_150mg:Teza_iva_50mg_300mg	0	No concerns	Low risk	No concerns	Major concerns	No concerns	No concerns	Low	[Imprecision]
Teza_iva_100mg_150mg:VX152_teza_iva_100mg_100mg_150mg	0	No concerns	Low risk	No concerns	Major concerns	No concerns	No concerns	Low	[Imprecision]
luma_100mg:Teza_iva_100mg_150mg	0	Some concerns	Low risk	No concerns	Major concerns	No concerns	No concerns	Low	[Within-study bias|Imprecision]
luma_200mg:Teza_iva_100mg_150mg	0	Some concerns	Low risk	No concerns	Major concerns	No concerns	No concerns	Low	[Within-study bias|Imprecision]
luma_25mg:Teza_iva_100mg_150mg	0	Some concerns	Low risk	No concerns	No concerns	No concerns	No concerns	Moderate	[Within-study bias]
luma_50mg:Teza_iva_100mg_150mg	0	Some concerns	Low risk	No concerns	No concerns	No concerns	No concerns	Moderate	[Within-study bias]
luma_iva_200mg_250mg:Teza_iva_100mg_150mg	0	No concerns	Low risk	No concerns	Major concerns	No concerns	No concerns	Low	[Imprecision]
luma_iva_400mg_250mg:Teza_iva_100mg_150mg	0	No concerns	Low risk	No concerns	Major concerns	No concerns	No concerns	Low	[Imprecision]
luma_iva_600mg_250mg:Teza_iva_100mg_150mg	0	No concerns	Low risk	No concerns	Major concerns	No concerns	No concerns	Low	[Imprecision]
Teza_iva_100mg_150mg:vanza_teza_deuti_10mg_100mg_150mg	0	No concerns	Low risk	No concerns	Major concerns	No concerns	No concerns	Low	[Imprecision]
Teza_iva_100mg_150mg:vanza_teza_deuti_5mg_100mg_150mg	0	No concerns	Low risk	No concerns	Major concerns	No concerns	No concerns	Low	[Imprecision]
Teza_iva_50mg_300mg:VX152_teza_iva_100mg_100mg_150mg	0	No concerns	Low risk	No concerns	Major concerns	No concerns	No concerns	Low	[Imprecision]
Teza_iva_50mg_300mg:VX152_teza_iva_200mg_100mg_150mg	0	No concerns	Low risk	No concerns	No concerns	Major concerns	No concerns	Low	[Heterogeneity]
Teza_iva_50mg_300mg:VX152_teza_iva_300mg_100mg_150mg	0	No concerns	Low risk	No concerns	No concerns	Major concerns	No concerns	Low	[Heterogeneity]
luma_100mg:Teza_iva_50mg_300mg	0	No concerns	Low risk	No concerns	Major concerns	No concerns	No concerns	Low	[Imprecision]
luma_200mg:Teza_iva_50mg_300mg	0	No concerns	Low risk	No concerns	Major concerns	No concerns	No concerns	Low	[Imprecision]
luma_25mg:Teza_iva_50mg_300mg	0	No concerns	Low risk	No concerns	Major concerns	No concerns	No concerns	Low	[Imprecision]
luma_50mg:Teza_iva_50mg_300mg	0	No concerns	Low risk	No concerns	Major concerns	No concerns	No concerns	Low	[Imprecision]

luma_iva_200mg_250mg:Teza_iva_50mg_300mg	0	No concerns	Low risk	No concerns	Major concerns	No concerns	No concerns	Low	[Imprecision]
luma_iva_400mg_250mg:Teza_iva_50mg_300mg	0	No concerns	Low risk	No concerns	Major concerns	No concerns	No concerns	Low	[Imprecision]
luma_iva_600mg_250mg:Teza_iva_50mg_300mg	0	No concerns	Low risk	No concerns	Major concerns	No concerns	No concerns	Low	[Imprecision]
Teza_iva_50mg_300mg:vanza_teza_deuti_10mg_100mg_150mg	0	No concerns	Low risk	No concerns	Major concerns	No concerns	No concerns	Low	[Imprecision]
Teza_iva_50mg_300mg:vanza_teza_deuti_20mg_100mg_150mg	0	No concerns	Low risk	No concerns	No concerns	No concerns	No concerns	High	NA
Teza_iva_50mg_300mg:vanza_teza_deuti_5mg_100mg_150mg	0	No concerns	Low risk	No concerns	Major concerns	No concerns	No concerns	Low	[Imprecision]
luma_100mg:VX152_teza_iva_100mg_100mg_150mg	0	No concerns	Low risk	No concerns	Major concerns	No concerns	No concerns	Low	[Imprecision]
luma_200mg:VX152_teza_iva_100mg_100mg_150mg	0	No concerns	Low risk	No concerns	Major concerns	No concerns	No concerns	Low	[Imprecision]
luma_25mg:VX152_teza_iva_100mg_100mg_150mg	0	No concerns	Low risk	No concerns	Major concerns	No concerns	No concerns	Low	[Imprecision]
luma_50mg:VX152_teza_iva_100mg_100mg_150mg	0	No concerns	Low risk	No concerns	Major concerns	No concerns	No concerns	Low	[Imprecision]
luma_iva_200mg_250mg:VX152_teza_iva_100mg_100mg_150mg	0	No concerns	Low risk	No concerns	Major concerns	No concerns	No concerns	Low	[Imprecision]
luma_iva_400mg_250mg:VX152_teza_iva_100mg_100mg_150mg	0	No concerns	Low risk	No concerns	Major concerns	No concerns	No concerns	Low	[Imprecision]
luma_iva_600mg_250mg:VX152_teza_iva_100mg_100mg_150mg	0	No concerns	Low risk	No concerns	Major concerns	No concerns	No concerns	Low	[Imprecision]
vanza_teza_deuti_10mg_100mg_150mg:VX152_teza_iva_100mg_100mg_150mg	0	No concerns	Low risk	No concerns	Major concerns	No concerns	No concerns	Low	[Imprecision]
vanza_teza_deuti_20mg_100mg_150mg:VX152_teza_iva_100mg_100mg_150mg	0	No concerns	Low risk	No concerns	No concerns	Major concerns	No concerns	Low	[Heterogeneity]
vanza_teza_deuti_5mg_100mg_150mg:VX152_teza_iva_100mg_100mg_150mg	0	No concerns	Low risk	No concerns	Major concerns	No concerns	No concerns	Low	[Imprecision]
luma_100mg:VX152_teza_iva_200mg_100mg_150mg	0	No concerns	Low risk	No concerns	No concerns	No concerns	No concerns	High	NA
luma_200mg:VX152_teza_iva_200mg_100mg_150mg	0	No concerns	Low risk	No concerns	No concerns	No concerns	No concerns	High	NA
luma_25mg:VX152_teza_iva_200mg_100mg_150mg	0	No concerns	Low risk	No concerns	No concerns	No concerns	No concerns	High	NA

luma_50mg:VX152_teza_iva_200mg_100mg_150mg	0	No concerns	Low risk	No concerns	No concerns	No concerns	No concerns	High	NA
luma_iva_200mg_250mg:VX152_teza_iva_200mg_100mg_150mg	0	No concerns	Low risk	No concerns	Major concerns	No concerns	No concerns	Low	[Imprecision]
luma_iva_400mg_250mg:VX152_teza_iva_200mg_100mg_150mg	0	No concerns	Low risk	No concerns	Major concerns	No concerns	No concerns	Low	[Imprecision]
luma_iva_600mg_250mg:VX152_teza_iva_200mg_100mg_150mg	0	No concerns	Low risk	No concerns	Major concerns	No concerns	No concerns	Low	[Imprecision]
vanza_teza_deuti_10mg_100mg_150mg:VX152_teza_iva_200mg_100mg_150mg	0	No concerns	Low risk	No concerns	Major concerns	No concerns	No concerns	Low	[Imprecision]
vanza_teza_deuti_20mg_100mg_150mg:VX152_teza_iva_200mg_100mg_150mg	0	No concerns	Low risk	No concerns	Major concerns	No concerns	No concerns	Low	[Imprecision]
vanza_teza_deuti_5mg_100mg_150mg:VX152_teza_iva_200mg_100mg_150mg	0	No concerns	Low risk	No concerns	Major concerns	No concerns	No concerns	Low	[Imprecision]
luma_100mg:VX152_teza_iva_300mg_100mg_150mg	0	No concerns	Low risk	No concerns	No concerns	No concerns	No concerns	High	NA
luma_200mg:VX152_teza_iva_300mg_100mg_150mg	0	No concerns	Low risk	No concerns	No concerns	No concerns	No concerns	High	NA
luma_25mg:VX152_teza_iva_300mg_100mg_150mg	0	No concerns	Low risk	No concerns	No concerns	No concerns	No concerns	High	NA
luma_50mg:VX152_teza_iva_300mg_100mg_150mg	0	No concerns	Low risk	No concerns	No concerns	No concerns	No concerns	High	NA
luma_iva_200mg_250mg:VX152_teza_iva_300mg_100mg_150mg	0	No concerns	Low risk	No concerns	Major concerns	No concerns	No concerns	Low	[Imprecision]
luma_iva_400mg_250mg:VX152_teza_iva_300mg_100mg_150mg	0	No concerns	Low risk	No concerns	Major concerns	No concerns	No concerns	Low	[Imprecision]
luma_iva_600mg_250mg:VX152_teza_iva_300mg_100mg_150mg	0	No concerns	Low risk	No concerns	Major concerns	No concerns	No concerns	Low	[Imprecision]
vanza_teza_deuti_10mg_100mg_150mg:VX152_teza_iva_300mg_100mg_150mg	0	No concerns	Low risk	No concerns	Major concerns	No concerns	No concerns	Low	[Imprecision]
vanza_teza_deuti_20mg_100mg_150mg:VX152_teza_iva_300mg_100mg_150mg	0	No concerns	Low risk	No concerns	Major concerns	No concerns	No concerns	Low	[Imprecision]
vanza_teza_deuti_5mg_100mg_150mg:VX152_teza_iva_300mg_100mg_150mg	0	No concerns	Low risk	No concerns	Major concerns	No concerns	No concerns	Low	[Imprecision]
luma_100mg:luma_iva_200mg_250mg	0	Some concerns	Low risk	No concerns	No concerns	No concerns	No concerns	Moderate	[Within-study bias]
luma_100mg:luma_iva_400mg_250mg	0	Some concerns	Low risk	No concerns	No concerns	No concerns	No concerns	Moderate	[Within-study bias]

luma_100mg:luma_iva_600mg_250mg	0	Some concerns	Low risk	No concerns	Major concerns	No concerns	No concerns	Low	[Imprecision]
luma_100mg:vanza_teza_deuti_10mg_100mg_150mg	0	No concerns	Low risk	No concerns	No concerns	No concerns	No concerns	High	NA
luma_100mg:vanza_teza_deuti_20mg_100mg_150mg	0	No concerns	Low risk	No concerns	No concerns	No concerns	No concerns	High	NA
luma_100mg:vanza_teza_deuti_5mg_100mg_150mg	0	No concerns	Low risk	No concerns	No concerns	Major concerns	No concerns	Low	[Heterogeneity]
luma_200mg:luma_iva_200mg_250mg	0	Some concerns	Low risk	No concerns	No concerns	Major concerns	No concerns	Low	[Within-study bias|Heterogeneity]
luma_200mg:luma_iva_400mg_250mg	0	Some concerns	Low risk	No concerns	Major concerns	No concerns	No concerns	Low	[Within-study bias|Imprecision]
luma_200mg:luma_iva_600mg_250mg	0	Some concerns	Low risk	No concerns	Major concerns	No concerns	No concerns	Low	[Within-study bias|Imprecision]
luma_200mg:vanza_teza_deuti_10mg_100mg_150mg	0	No concerns	Low risk	No concerns	No concerns	No concerns	No concerns	High	NA
luma_200mg:vanza_teza_deuti_20mg_100mg_150mg	0	No concerns	Low risk	No concerns	No concerns	No concerns	No concerns	High	NA
luma_200mg:vanza_teza_deuti_5mg_100mg_150mg	0	No concerns	Low risk	No concerns	Major concerns	No concerns	No concerns	Low	[Imprecision]
luma_25mg:luma_iva_200mg_250mg	0	Some concerns	Low risk	No concerns	No concerns	No concerns	No concerns	Moderate	[Within-study bias]
luma_25mg:luma_iva_400mg_250mg	0	Some concerns	Low risk	No concerns	No concerns	No concerns	No concerns	Moderate	[Within-study bias]
luma_25mg:luma_iva_600mg_250mg	0	Some concerns	Low risk	No concerns	Major concerns	No concerns	No concerns	Low	[Within-study bias|Imprecision]
luma_25mg:vanza_teza_deuti_10mg_100mg_150mg	0	No concerns	Low risk	No concerns	No concerns	No concerns	No concerns	High	NA
luma_25mg:vanza_teza_deuti_20mg_100mg_150mg	0	No concerns	Low risk	No concerns	No concerns	No concerns	No concerns	High	NA
luma_25mg:vanza_teza_deuti_5mg_100mg_150mg	0	No concerns	Low risk	No concerns	No concerns	No concerns	No concerns	High	NA
luma_50mg:luma_iva_200mg_250mg	0	Some concerns	Low risk	No concerns	No concerns	No concerns	No concerns	Moderate	[Within-study bias]
luma_50mg:luma_iva_400mg_250mg	0	Some concerns	Low risk	No concerns	No concerns	No concerns	No concerns	Moderate	[Within-study bias]

luma_50mg:luma_iva_600mg_250mg	0	Some concerns	Low risk	No concerns	No concerns	Major concerns	No concerns	Low	[Within-study bias|Heterogeneity]
luma_50mg:vanza_teza_deuti_10mg_100mg_150mg	0	No concerns	Low risk	No concerns	No concerns	No concerns	No concerns	High	NA
luma_50mg:vanza_teza_deuti_20mg_100mg_150mg	0	No concerns	Low risk	No concerns	No concerns	No concerns	No concerns	High	NA
luma_50mg:vanza_teza_deuti_5mg_100mg_150mg	0	No concerns	Low risk	No concerns	No concerns	No concerns	No concerns	High	NA
luma_iva_200mg_250mg:vanza_teza_deuti_10mg_100mg_150mg	0	No concerns	Low risk	No concerns	Major concerns	No concerns	No concerns	Low	[Imprecision]
luma_iva_200mg_250mg:vanza_teza_deuti_20mg_100mg_150mg	0	No concerns	Low risk	No concerns	Major concerns	No concerns	No concerns	Low	[Imprecision]
luma_iva_200mg_250mg:vanza_teza_deuti_5mg_100mg_150mg	0	No concerns	Low risk	No concerns	Major concerns	No concerns	No concerns	Low	[Imprecision]
luma_iva_400mg_250mg:vanza_teza_deuti_10mg_100mg_150mg	0	No concerns	Low risk	No concerns	Major concerns	No concerns	No concerns	Low	[Imprecision]
luma_iva_400mg_250mg:vanza_teza_deuti_20mg_100mg_150mg	0	No concerns	Low risk	No concerns	No concerns	No concerns	No concerns	High	NA
luma_iva_400mg_250mg:vanza_teza_deuti_5mg_100mg_150mg	0	No concerns	Low risk	No concerns	Major concerns	No concerns	No concerns	Low	[Imprecision]
luma_iva_600mg_250mg:vanza_teza_deuti_10mg_100mg_150mg	0	No concerns	Low risk	No concerns	Major concerns	No concerns	No concerns	Low	[Imprecision]
luma_iva_600mg_250mg:vanza_teza_deuti_20mg_100mg_150mg	0	No concerns	Low risk	No concerns	No concerns	No concerns	No concerns	High	NA
luma_iva_600mg_250mg:vanza_teza_deuti_5mg_100mg_150mg	0	No concerns	Low risk	No concerns	Major concerns	No concerns	No concerns	Low	[Imprecision]

eTable 12 Certainty ratings of CFQ-R for adults treated greater than 8 weeks using CINEMA framework

Comparison	Number of studies	Within-study bias	Reporting bias	Indirectness	Imprecision	Heterogeneity	Incoherence	Confidence rating	Reason(s) for downgrading
Mixed evidence									
Elexa_teza_iva_200mg_100mg_150mg:Placebo	1	No concerns	Low risk	No concerns	No concerns	No concerns	No concerns	High	NA
Elexa_teza_iva_200mg_100mg_150mg:Teza_iva_100mg_150mg	1	No concerns	Low risk	No concerns	No concerns	No concerns	No concerns	High	NA
Elexa_teza_iva_200mg_100mg_150mg: Vanza_teza_deuti_20mg_100mg_250mg	2	No concerns	Low risk	No concerns	Major concerns	No concerns	No concerns	Low	["Imprecision"]
Iva_150mg:Placebo	1	No concerns	Low risk	No concerns	Major concerns	No concerns	No concerns	Low	["Imprecision"]
Placebo:Teza_iva_100mg_150mg	3	No concerns	Low risk	No concerns	No concerns	Major concerns	No concerns	Low	["Heterogeneity"]
Placebo:Teza_iva_50mg_150mg	1	Some concerns	Low risk	No concerns	Major concerns	No concerns	No concerns	Low	["Within-study bias","Imprecision"]
luma_iva_400mg_250mg:Placebo	2	No concerns	Low risk	No concerns	Major concerns	No concerns	No concerns	Low	["Imprecision"]
luma_iva_600mg_250mg:Placebo	2	No concerns	Low risk	No concerns	Major concerns	No concerns	No concerns	Low	["Imprecision"]
luma_iva_400mg_250mg:luma_iva_600mg_250mg	3	No concerns	Low risk	No concerns	Major concerns	No concerns	No concerns	Low	["Imprecision"]
Indirect evidence									
Elexa_teza_iva_200mg_100mg_150mg:Iva_150mg	0	No concerns	Low risk	No concerns	No concerns	No concerns	No concerns	High	NA
Elexa_teza_iva_200mg_100mg_150mg:Teza_iva_50mg_150mg	0	No concerns	Low risk	No concerns	Major concerns	No concerns	No concerns	Low	["Imprecision"]
Elexa_teza_iva_200mg_100mg_150mg:luma_iva_400mg_250mg	0	No concerns	Low risk	No concerns	No concerns	No concerns	No concerns	High	NA
Elexa_teza_iva_200mg_100mg_150mg:luma_iva_600mg_250mg	0	No concerns	Low risk	No concerns	No concerns	No concerns	No concerns	High	NA
Iva_150mg:Teza_iva_100mg_150mg	0	No concerns	Low risk	No concerns	Major concerns	No concerns	No concerns	Low	["Imprecision"]
Iva_150mg:Teza_iva_50mg_150mg	0	Some concerns	Low risk	No concerns	Major concerns	No concerns	No concerns	Low	["Within-study bias","Imprecision"]

Iva_150mg: Vanza_teza_deuti_20mg_100mg_250mg	0	No concerns	Low risk	No concerns	No concerns	No concerns	No concerns	High	NA
Iva_150mg:luma_iva_400mg_250mg	0	No concerns	Low risk	No concerns	Major concerns	No concerns	No concerns	Low	["Imprecision"]
Iva_150mg:luma_iva_600mg_250mg	0	No concerns	Low risk	No concerns	Major concerns	No concerns	No concerns	Low	["Imprecision"]
Placebo: Vanza_teza_deuti_20mg_100mg_250mg	0	No concerns	Low risk	No concerns	No concerns	No concerns	No concerns	High	NA
Teza_iva_100mg_150mg:Teza_iva_50mg_150mg	0	Some concerns	Low risk	No concerns	Major concerns	No concerns	No concerns	Low	["Within-study bias","Imprecision"]
Vanza_teza_iva_20mg_100mg_250mg:Teza_iva_100mg_150mg	0	No concerns	Low risk	No concerns	No concerns	No concerns	No concerns	High	NA
luma_iva_400mg_250mg:Teza_iva_100mg_150mg	0	No concerns	Low risk	No concerns	Major concerns	No concerns	No concerns	Low	["Imprecision"]
luma_iva_600mg_250mg:Teza_iva_100mg_150mg	0	No concerns	Low risk	No concerns	Major concerns	No concerns	No concerns	Low	["Imprecision"]
Vanza_teza_deuti_240mg_100mg_150mg:Teza_iva_50mg_150mg	0	No concerns	Low risk	No concerns	Major concerns	No concerns	No concerns	Low	["Imprecision"]
luma_iva_400mg_250mg:Teza_iva_50mg_150mg	0	No concerns	Low risk	No concerns	Major concerns	No concerns	No concerns	Low	["Imprecision"]
luma_iva_600mg_250mg:Teza_iva_50mg_150mg	0	No concerns	Low risk	No concerns	Major concerns	No concerns	No concerns	Low	["Imprecision"]
Vanza_teza_deuti_20mg_100mg_250mg:luma_iva_400mg_250mg	0	No concerns	Low risk	No concerns	No concerns	No concerns	No concerns	High	NA
Vamoca_teza_deuti_20mg_100mg_150mg:luma_iva_600mg_250mg	0	No concerns	Low risk	No concerns	No concerns	No concerns	No concerns	High	NA

eTable 13 Certainty ratings of Serious adverse events for adults treated for 4 to 8 weeks using CINEMA framework

Comparison	Number of studies	Within-study bias	Reporting bias	Indirectness	Imprecision	Heterogeneity	Incoherence	Confidence rating	Reason(s) for downgrading
Mixed evidence									
Elexa_teza_iva_100mg_100mg_150mg:Elexa_teza_iva_200mg_100mg_150mg	1	No concerns	Low risk	No concerns	Major concerns	No concerns	No concerns	Low	[Imprecision]
Elexa_teza_iva_100mg_100mg_150mg:Elexa_teza_iva_50mg_100mg_150mg	1	No concerns	Low risk	No concerns	Major concerns	No concerns	No concerns	Low	[Imprecision]
Elexa_teza_iva_100mg_100mg_150mg:Placebo	1	No concerns	Low risk	No concerns	Major concerns	No concerns	No concerns	Low	[Imprecision]
Elexa_teza_iva_200mg_100mg_150mg:Elexa_teza_iva_50mg_100mg_150mg	1	No concerns	Low risk	No concerns	Major concerns	No concerns	No concerns	Low	[Imprecision]
Elexa_teza_iva_200mg_100mg_150mg:Placebo	1	No concerns	Low risk	No concerns	Major concerns	No concerns	No concerns	Low	[Imprecision]
Elexa_teza_iva_200mg_100mg_150mg:Teza_iva_100mg_150mg	2	No concerns	Low risk	No concerns	Major concerns	No concerns	No concerns	Low	[Imprecision]
Elexa_teza_iva_50mg_100mg_150mg:Placebo	1	No concerns	Low risk	No concerns	Major concerns	No concerns	No concerns	Low	[Imprecision]
GLPG2222_100mg:GLPG2222_200mg	1	No concerns	Low risk	No concerns	Major concerns	No concerns	No concerns	Low	[Imprecision]
GLPG2222_100mg:GLPG2222_400mg	1	No concerns	Low risk	No concerns	Major concerns	No concerns	No concerns	Low	[Imprecision]
GLPG2222_100mg:GLPG2222_50mg	1	No concerns	Low risk	No concerns	Major concerns	No concerns	No concerns	Low	[Imprecision]
GLPG2222_100mg:Placebo	1	No concerns	Low risk	No concerns	Major concerns	No concerns	No concerns	Low	[Imprecision]
GLPG2222_200mg:GLPG2222_400mg	1	No concerns	Low risk	No concerns	Major concerns	No concerns	No concerns	Low	[Imprecision]
GLPG2222_200mg:GLPG2222_50mg	1	No concerns	Low risk	No concerns	Major concerns	No concerns	No concerns	Low	[Imprecision]
GLPG2222_200mg:Placebo	1	No concerns	Low risk	No concerns	Major concerns	No concerns	No concerns	Low	[Imprecision]
GLPG2222_400mg:GLPG2222_50mg	1	No concerns	Low risk	No concerns	Major concerns	No concerns	No concerns	Low	[Imprecision]
GLPG2222_400mg:Placebo	1	No concerns	Low risk	No concerns	Major concerns	No concerns	No concerns	Low	[Imprecision]
GLPG2222_50mg:Placebo	1	No concerns	Low risk	No concerns	Major concerns	No concerns	No concerns	Low	[Imprecision]
Iva_150mg:Placebo	1	No concerns	Low risk	No concerns	Major concerns	No concerns	No concerns	Low	[Imprecision]
Iva_150mg:Teza_iva_100mg_150mg	2	No concerns	Low risk	No concerns	Major concerns	No concerns	No concerns	Low	[Imprecision]
Ola_teza_iva_200mg_100mg_150mg:Ola_teza_iva_200mg_50mg_150mg	1	No concerns	Low risk	No concerns	Major concerns	No concerns	No concerns	Low	[Imprecision]

Ola_teza_iva_200mg_100mg_150mg:Ola_teza_iva_600mg_50mg_300mg	1	No concerns	Low risk	No concerns	Major concerns	No concerns	No concerns	Low	[Imprecision]
Ola_teza_iva_200mg_100mg_150mg:Placebo	1	No concerns	Low risk	No concerns	Major concerns	No concerns	No concerns	Low	[Imprecision]
Ola_teza_iva_200mg_50mg_150mg:Ola_teza_iva_600mg_50mg_300mg	1	No concerns	Low risk	No concerns	Major concerns	No concerns	No concerns	Low	[Imprecision]
Ola_teza_iva_200mg_50mg_150mg:Placebo	1	No concerns	Low risk	No concerns	Major concerns	No concerns	No concerns	Low	[Imprecision]
Ola_teza_iva_600mg_50mg_300mg:Placebo	1	No concerns	Low risk	No concerns	Major concerns	No concerns	No concerns	Low	[Imprecision]
Ola_teza_iva_600mg_50mg_300mg:Teza_iva_50mg_300mg	1	No concerns	Low risk	No concerns	Major concerns	No concerns	No concerns	Low	[Imprecision]
Placebo:Teza_iva_100mg_150mg	4	No concerns	Low risk	No concerns	Major concerns	Major concerns	No concerns	Very Low	[Imprecision| Heterogeneity]
Placebo:VX152_teza_iva_100mg_100mg_150mg	1	No concerns	Low risk	No concerns	Major concerns	No concerns	No concerns	Low	[Imprecision]
Placebo:VX152_teza_iva_200mg_100mg_150mg	1	No concerns	Low risk	No concerns	Major concerns	No concerns	No concerns	Low	[Imprecision]
Placebo:VX152_teza_iva_300mg_100mg_150mg	1	No concerns	Low risk	No concerns	Major concerns	No concerns	No concerns	Low	[Imprecision]
luma_100mg:Placebo	1	Some concerns	Low risk	No concerns	Major concerns	No concerns	No concerns	Low	[Within-study bias| Imprecision]
luma_200mg:Placebo	1	Some concerns	Low risk	No concerns	Major concerns	No concerns	No concerns	Low	[Within-study bias| Imprecision]
luma_25mg:Placebo	1	Some concerns	Low risk	No concerns	Major concerns	No concerns	No concerns	Low	[Within-study bias| Imprecision]
luma_50mg:Placebo	1	Some concerns	Low risk	No concerns	Major concerns	No concerns	No concerns	Low	[Within-study bias| Imprecision]
luma_iva_200mg_250mg:Placebo	1	No concerns	Low risk	No concerns	Major concerns	No concerns	No concerns	Low	[Imprecision]
luma_iva_400mg_250mg:Placebo	2	Some concerns	Low risk	No concerns	Major concerns	No concerns	No concerns	Low	[Within-study bias| Imprecision]
luma_iva_600mg_250mg:Placebo	1	No concerns	Low risk	No concerns	Major concerns	No concerns	No concerns	Low	[Imprecision]
Placebo:vanza_teza_deuti_10mg_100mg_150mg	1	No concerns	Low risk	No concerns	Major concerns	No concerns	No concerns	Low	[Imprecision]
Placebo:vanza_teza_deuti_20mg_100mg_150mg	1	No concerns	Low risk	No concerns	Major concerns	No concerns	No concerns	Low	[Imprecision]
Placebo:vanza_teza_deuti_5mg_100mg_150mg	1	No concerns	Low risk	No concerns	Major concerns	No concerns	No concerns	Low	[Imprecision]
Teza_iva_100mg_150mg:Teza_iva_50mg_150mg	1	Some concerns	Low risk	No concerns	Major concerns	No concerns	No concerns	Low	[Within-study bias| Imprecision]
VX152_teza_iva_100mg_100mg_150mg:VX152_teza_iva_200mg_100mg_150mg	1	No concerns	Low risk	No concerns	Major concerns	No concerns	No concerns	Low	[Imprecision]

VX152_teza_iva_100mg_100mg_150mg:VX152_teza_iva_300mg_100mg_150mg	1	No concerns	Low risk	No concerns	Major concerns	No concerns	No concerns	Low	[Imprecision]
VX152_teza_iva_200mg_100mg_150mg:VX152_teza_iva_300mg_100mg_150mg	1	No concerns	Low risk	No concerns	Major concerns	No concerns	No concerns	Low	[Imprecision]
luma_100mg:luma_200mg	1	Some concerns	Low risk	No concerns	Major concerns	No concerns	No concerns	Low	[Within-study bias| Imprecision]
luma_100mg:luma_25mg	1	Some concerns	Low risk	No concerns	Major concerns	No concerns	No concerns	Low	[Within-study bias| Imprecision]
luma_100mg:luma_50mg	1	Some concerns	Low risk	No concerns	Major concerns	No concerns	No concerns	Low	[Within-study bias| Imprecision]
luma_200mg:luma_25mg	1	Some concerns	Low risk	No concerns	Major concerns	No concerns	No concerns	Low	[Within-study bias| Imprecision]
luma_200mg:luma_50mg	1	Some concerns	Low risk	No concerns	Major concerns	No concerns	No concerns	Low	[Within-study bias| Imprecision]
luma_25mg:luma_50mg	1	Some concerns	Low risk	No concerns	Major concerns	No concerns	No concerns	Low	[Within-study bias| Imprecision]
luma_iva_200mg_250mg:luma_iva_400mg_250mg	1	No concerns	Low risk	No concerns	Major concerns	No concerns	Major concerns	Very Low	[Imprecision|Incoherence]
luma_iva_200mg_250mg:luma_iva_600mg_250mg	1	No concerns	Low risk	No concerns	Major concerns	No concerns	No concerns	Low	[Imprecision]
luma_iva_400mg_250mg:luma_iva_600mg_250mg	1	No concerns	Low risk	No concerns	Major concerns	No concerns	No concerns	Low	[Imprecision]
vanza_teza_deuti_10mg_100mg_150mg:vanza_teza_deuti_20mg_100mg_150mg	1	No concerns	Low risk	No concerns	Major concerns	No concerns	No concerns	Low	[Imprecision]
vanza_teza_deuti_10mg_100mg_150mg:vanza_teza_deuti_5mg_100mg_150mg	1	No concerns	Low risk	No concerns	Major concerns	No concerns	No concerns	Low	[Imprecision]
vanza_teza_deuti_20mg_100mg_150mg:vanza_teza_deuti_5mg_100mg_150mg	1	No concerns	Low risk	No concerns	Major concerns	No concerns	No concerns	Low	[Imprecision]
Indirect evidence									
Elexa_teza_iva_100mg_100mg_150mg:GLPG2222_100mg	0	No concerns	Low risk	No concerns	Major concerns	No concerns	No concerns	Low	[Imprecision]
Elexa_teza_iva_100mg_100mg_150mg:GLPG2222_200mg	0	No concerns	Low risk	No concerns	Major concerns	No concerns	No concerns	Low	[Imprecision]
Elexa_teza_iva_100mg_100mg_150mg:GLPG2222_400mg	0	No concerns	Low risk	No concerns	Major concerns	No concerns	No concerns	Low	[Imprecision]
Elexa_teza_iva_100mg_100mg_150mg:GLPG2222_50mg	0	No concerns	Low risk	No concerns	Major concerns	No concerns	No concerns	Low	[Imprecision]

Elexa_teza_iva_100mg_100mg_150mg:Iva_150mg	0	No concerns	Low risk	No concerns	Major concerns	No concerns	No concerns	Low	[Imprecision]
Elexa_teza_iva_100mg_100mg_150mg:Ola_teza_iva_200mg_100mg_150mg	0	No concerns	Low risk	No concerns	Major concerns	No concerns	No concerns	Low	[Imprecision]
Elexa_teza_iva_100mg_100mg_150mg:Ola_teza_iva_200mg_50mg_150mg	0	No concerns	Low risk	No concerns	Major concerns	No concerns	No concerns	Low	[Imprecision]
Elexa_teza_iva_100mg_100mg_150mg:Ola_teza_iva_600mg_50mg_300mg	0	No concerns	Low risk	No concerns	Major concerns	No concerns	No concerns	Low	[Imprecision]
Elexa_teza_iva_100mg_100mg_150mg:Teza_iva_100mg_150mg	0	No concerns	Low risk	No concerns	Major concerns	No concerns	No concerns	Low	[Imprecision]
Elexa_teza_iva_100mg_100mg_150mg:Teza_iva_50mg_150mg	0	No concerns	Low risk	No concerns	Major concerns	No concerns	No concerns	Low	[Imprecision]
Elexa_teza_iva_100mg_100mg_150mg:Teza_iva_50mg_300mg	0	No concerns	Low risk	No concerns	Major concerns	No concerns	No concerns	Low	[Imprecision]
Elexa_teza_iva_100mg_100mg_150mg:VX152_teza_iva_100mg_100mg_150mg	0	No concerns	Low risk	No concerns	Major concerns	No concerns	No concerns	Low	[Imprecision]
Elexa_teza_iva_100mg_100mg_150mg:VX152_teza_iva_200mg_100mg_150mg	0	No concerns	Low risk	No concerns	Major concerns	No concerns	No concerns	Low	[Imprecision]
Elexa_teza_iva_100mg_100mg_150mg:VX152_teza_iva_300mg_100mg_150mg	0	No concerns	Low risk	No concerns	Major concerns	No concerns	No concerns	Low	[Imprecision]
Elexa_teza_iva_100mg_100mg_150mg:luma_100mg	0	Some concerns	Low risk	No concerns	Major concerns	No concerns	No concerns	Low	[Within-study bias| Imprecision]
Elexa_teza_iva_100mg_100mg_150mg:luma_200mg	0	Some concerns	Low risk	No concerns	Major concerns	No concerns	No concerns	Low	[Within-study bias| Imprecision]
Elexa_teza_iva_100mg_100mg_150mg:luma_25mg	0	Some concerns	Low risk	No concerns	Major concerns	No concerns	No concerns	Low	[Within-study bias| Imprecision]
Elexa_teza_iva_100mg_100mg_150mg:luma_50mg	0	Some concerns	Low risk	No concerns	Major concerns	No concerns	No concerns	Low	[Within-study bias| Imprecision]
Elexa_teza_iva_100mg_100mg_150mg:luma_iva_200mg_250mg	0	No concerns	Low risk	No concerns	Major concerns	No concerns	No concerns	Low	[Imprecision]
Elexa_teza_iva_100mg_100mg_150mg:luma_iva_400mg_250mg	0	No concerns	Low risk	No concerns	Major concerns	No concerns	No concerns	Low	[Imprecision]
Elexa_teza_iva_100mg_100mg_150mg:luma_iva_600mg_250mg	0	No concerns	Low risk	No concerns	Major concerns	No concerns	No concerns	Low	[Imprecision]
Elexa_teza_iva_100mg_100mg_150mg:vanza_teza_deuti_10mg_100mg_150mg	0	No concerns	Low risk	No concerns	Major concerns	No concerns	No concerns	Low	[Imprecision]

Elexa_teza_iva_100mg_100mg_150mg:vanza_teza_deuti_20mg_100mg_150mg	0	No concerns	Low risk	No concerns	Major concerns	No concerns	No concerns	Low	[Imprecision]
Elexa_teza_iva_100mg_100mg_150mg:vanza_teza_deuti_5mg_100mg_150mg	0	No concerns	Low risk	No concerns	Major concerns	No concerns	No concerns	Low	[Imprecision]
Elexa_teza_iva_200mg_100mg_150mg:GLPG2222_100mg	0	No concerns	Low risk	No concerns	Major concerns	No concerns	No concerns	Low	[Imprecision]
Elexa_teza_iva_200mg_100mg_150mg:GLPG2222_200mg	0	No concerns	Low risk	No concerns	Major concerns	No concerns	No concerns	Low	[Imprecision]
Elexa_teza_iva_200mg_100mg_150mg:GLPG2222_400mg	0	No concerns	Low risk	No concerns	Major concerns	No concerns	No concerns	Low	[Imprecision]
Elexa_teza_iva_200mg_100mg_150mg:GLPG2222_50mg	0	No concerns	Low risk	No concerns	Major concerns	No concerns	No concerns	Low	[Imprecision]
Elexa_teza_iva_200mg_100mg_150mg:Iva_150mg	0	No concerns	Low risk	No concerns	Major concerns	No concerns	No concerns	Low	[Imprecision]
Elexa_teza_iva_200mg_100mg_150mg:Ola_teza_iva_200mg_100mg_150mg	0	No concerns	Low risk	No concerns	Major concerns	No concerns	No concerns	Low	[Imprecision]
Elexa_teza_iva_200mg_100mg_150mg:Ola_teza_iva_200mg_50mg_150mg	0	No concerns	Low risk	No concerns	Major concerns	No concerns	No concerns	Low	[Imprecision]
Elexa_teza_iva_200mg_100mg_150mg:Ola_teza_iva_600mg_50mg_300mg	0	No concerns	Low risk	No concerns	Major concerns	No concerns	No concerns	Low	[Imprecision]
Elexa_teza_iva_200mg_100mg_150mg:Teza_iva_50mg_150mg	0	No concerns	Low risk	No concerns	Major concerns	No concerns	No concerns	Low	[Imprecision]
Elexa_teza_iva_200mg_100mg_150mg:Teza_iva_50mg_300mg	0	No concerns	Low risk	No concerns	No concerns	Major concerns	No concerns	Low	[Heterogeneity]
Elexa_teza_iva_200mg_100mg_150mg:VX152_teza_iva_100mg_100mg_150mg	0	No concerns	Low risk	No concerns	Major concerns	No concerns	No concerns	Low	[Imprecision]
Elexa_teza_iva_200mg_100mg_150mg:VX152_teza_iva_200mg_100mg_150mg	0	No concerns	Low risk	No concerns	Major concerns	No concerns	No concerns	Low	[Imprecision]
Elexa_teza_iva_200mg_100mg_150mg:VX152_teza_iva_300mg_100mg_150mg	0	No concerns	Low risk	No concerns	Major concerns	No concerns	No concerns	Low	[Imprecision]
Elexa_teza_iva_200mg_100mg_150mg:luma_100mg	0	No concerns	Low risk	No concerns	Major concerns	No concerns	No concerns	Low	[Imprecision]
Elexa_teza_iva_200mg_100mg_150mg:luma_200mg	0	No concerns	Low risk	No concerns	Major concerns	No concerns	No concerns	Low	[Imprecision]
Elexa_teza_iva_200mg_100mg_150mg:luma_25mg	0	No concerns	Low risk	No concerns	No concerns	No concerns	No concerns	High	NA
Elexa_teza_iva_200mg_100mg_150mg:luma_50mg	0	No concerns	Low risk	No concerns	Major concerns	No concerns	No concerns	Low	[Imprecision]

Elexa_teza_iva_200mg_100mg_150mg:luma_iva_200mg_250mg	0	No concerns	Low risk	No concerns	Major concerns	No concerns	No concerns	Low	[Imprecision]
Elexa_teza_iva_200mg_100mg_150mg:luma_iva_400mg_250mg	0	No concerns	Low risk	No concerns	Major concerns	No concerns	No concerns	Low	[Imprecision]
Elexa_teza_iva_200mg_100mg_150mg:luma_iva_600mg_250mg	0	No concerns	Low risk	No concerns	Major concerns	No concerns	No concerns	Low	[Imprecision]
Elexa_teza_iva_200mg_100mg_150mg:vanza_teza_deuti_10mg_100mg_150mg	0	No concerns	Low risk	No concerns	Major concerns	No concerns	No concerns	Low	[Imprecision]
Elexa_teza_iva_200mg_100mg_150mg:vanza_teza_deuti_20mg_100mg_150mg	0	No concerns	Low risk	No concerns	Major concerns	No concerns	No concerns	Low	[Imprecision]
Elexa_teza_iva_200mg_100mg_150mg:vanza_teza_deuti_5mg_100mg_150mg	0	No concerns	Low risk	No concerns	Major concerns	No concerns	No concerns	Low	[Imprecision]
Elexa_teza_iva_50mg_100mg_150mg:GLPG2222_100mg	0	No concerns	Low risk	No concerns	Major concerns	No concerns	No concerns	Low	[Imprecision]
Elexa_teza_iva_50mg_100mg_150mg:GLPG2222_200mg	0	No concerns	Low risk	No concerns	Major concerns	No concerns	No concerns	Low	[Imprecision]
Elexa_teza_iva_50mg_100mg_150mg:GLPG2222_400mg	0	No concerns	Low risk	No concerns	Major concerns	No concerns	No concerns	Low	[Imprecision]
Elexa_teza_iva_50mg_100mg_150mg:GLPG2222_50mg	0	No concerns	Low risk	No concerns	Major concerns	No concerns	No concerns	Low	[Imprecision]
Elexa_teza_iva_50mg_100mg_150mg:Iva_150mg	0	No concerns	Low risk	No concerns	Major concerns	No concerns	No concerns	Low	[Imprecision]
Elexa_teza_iva_50mg_100mg_150mg:Ola_teza_iva_200mg_100mg_150mg	0	No concerns	Low risk	No concerns	Major concerns	No concerns	No concerns	Low	[Imprecision]
Elexa_teza_iva_50mg_100mg_150mg:Ola_teza_iva_200mg_50mg_150mg	0	No concerns	Low risk	No concerns	Major concerns	No concerns	No concerns	Low	[Imprecision]
Elexa_teza_iva_50mg_100mg_150mg:Ola_teza_iva_600mg_50mg_300mg	0	No concerns	Low risk	No concerns	Major concerns	No concerns	No concerns	Low	[Imprecision]
Elexa_teza_iva_50mg_100mg_150mg:Teza_iva_100mg_150mg	0	No concerns	Low risk	No concerns	Major concerns	No concerns	No concerns	Low	[Imprecision]
Elexa_teza_iva_50mg_100mg_150mg:Teza_iva_50mg_150mg	0	No concerns	Low risk	No concerns	Major concerns	No concerns	No concerns	Low	[Imprecision]
Elexa_teza_iva_50mg_100mg_150mg:Teza_iva_50mg_300mg	0	No concerns	Low risk	No concerns	Major concerns	No concerns	No concerns	Low	[Imprecision]
Elexa_teza_iva_50mg_100mg_150mg:VX152_teza_iva_100mg_100mg_150mg	0	No concerns	Low risk	No concerns	Major concerns	No concerns	No concerns	Low	[Imprecision]
Elexa_teza_iva_50mg_100mg_150mg:VX152_teza_iva_200mg_100mg_150mg	0	No concerns	Low risk	No concerns	Major concerns	No concerns	No concerns	Low	[Imprecision]

Elexa_teza_iva_50mg_100mg_150mg:VX152_teza_iva_300mg_100mg_150mg	0	No concerns	Low risk	No concerns	Major concerns	No concerns	No concerns	Low	[Imprecision]
Elexa_teza_iva_50mg_100mg_150mg:luma_100mg	0	Some concerns	Low risk	No concerns	Major concerns	No concerns	No concerns	Low	[Within-study bias| Imprecision]
Elexa_teza_iva_50mg_100mg_150mg:luma_200mg	0	Some concerns	Low risk	No concerns	Major concerns	No concerns	No concerns	Low	[Within-study bias| Imprecision]
Elexa_teza_iva_50mg_100mg_150mg:luma_25mg	0	No concerns	Low risk	No concerns	Major concerns	No concerns	No concerns	Low	[Imprecision]
Elexa_teza_iva_50mg_100mg_150mg:luma_50mg	0	Some concerns	Low risk	No concerns	Major concerns	No concerns	No concerns	Low	[Within-study bias| Imprecision]
Elexa_teza_iva_50mg_100mg_150mg:luma_iva_200mg_250mg	0	No concerns	Low risk	No concerns	Major concerns	No concerns	No concerns	Low	[Imprecision]
Elexa_teza_iva_50mg_100mg_150mg:luma_iva_400mg_250mg	0	No concerns	Low risk	No concerns	Major concerns	No concerns	No concerns	Low	[Imprecision]
Elexa_teza_iva_50mg_100mg_150mg:luma_iva_600mg_250mg	0	No concerns	Low risk	No concerns	Major concerns	No concerns	No concerns	Low	[Imprecision]
Elexa_teza_iva_50mg_100mg_150mg:vanza_teza_deuti_10mg_100mg_150mg	0	No concerns	Low risk	No concerns	Major concerns	No concerns	No concerns	Low	[Imprecision]
Elexa_teza_iva_50mg_100mg_150mg:vanza_teza_deuti_20mg_100mg_150mg	0	No concerns	Low risk	No concerns	Major concerns	No concerns	No concerns	Low	[Imprecision]
Elexa_teza_iva_50mg_100mg_150mg:vanza_teza_deuti_5mg_100mg_150mg	0	No concerns	Low risk	No concerns	Major concerns	No concerns	No concerns	Low	[Imprecision]
GLPG2222_100mg:Iva_150mg	0	No concerns	Low risk	No concerns	Major concerns	No concerns	No concerns	Low	[Imprecision]
GLPG2222_100mg:Ola_teza_iva_200mg_100mg_150mg	0	No concerns	Low risk	No concerns	Major concerns	No concerns	No concerns	Low	[Imprecision]
GLPG2222_100mg:Ola_teza_iva_200mg_50mg_150mg	0	No concerns	Low risk	No concerns	Major concerns	No concerns	No concerns	Low	[Imprecision]
GLPG2222_100mg:Ola_teza_iva_600mg_50mg_300mg	0	No concerns	Low risk	No concerns	Major concerns	No concerns	No concerns	Low	[Imprecision]
GLPG2222_100mg:Teza_iva_100mg_150mg	0	No concerns	Low risk	No concerns	Major concerns	No concerns	No concerns	Low	[Imprecision]
GLPG2222_100mg:Teza_iva_50mg_150mg	0	No concerns	Low risk	No concerns	Major concerns	No concerns	No concerns	Low	[Imprecision]
GLPG2222_100mg:Teza_iva_50mg_300mg	0	No concerns	Low risk	No concerns	Major concerns	No concerns	No concerns	Low	[Imprecision]
GLPG2222_100mg:VX152_teza_iva_100mg_100mg_150mg	0	No concerns	Low risk	No concerns	Major concerns	No concerns	No concerns	Low	[Imprecision]
GLPG2222_100mg:VX152_teza_iva_200mg_100mg_150mg	0	No concerns	Low risk	No concerns	Major concerns	No concerns	No concerns	Low	[Imprecision]

GLPG2222_100mg:VX152_teza_iva_300mg_100mg_150mg	0	No concerns	Low risk	No concerns	Major concerns	No concerns	No concerns	Low	[Imprecision]
GLPG2222_100mg:luma_100mg	0	Some concerns	Low risk	No concerns	Major concerns	No concerns	No concerns	Low	[Within-study bias| Imprecision]
GLPG2222_100mg:luma_200mg	0	Some concerns	Low risk	No concerns	Major concerns	No concerns	No concerns	Low	[Within-study bias| Imprecision]
GLPG2222_100mg:luma_25mg	0	Some concerns	Low risk	No concerns	Major concerns	No concerns	No concerns	Low	[Within-study bias| Imprecision]
GLPG2222_100mg:luma_50mg	0	Some concerns	Low risk	No concerns	Major concerns	No concerns	No concerns	Low	[Within-study bias| Imprecision]
GLPG2222_100mg:luma_iva_200mg_250mg	0	No concerns	Low risk	No concerns	Major concerns	No concerns	No concerns	Low	[Imprecision]
GLPG2222_100mg:luma_iva_400mg_250mg	0	No concerns	Low risk	No concerns	Major concerns	No concerns	No concerns	Low	[Imprecision]
GLPG2222_100mg:luma_iva_600mg_250mg	0	No concerns	Low risk	No concerns	Major concerns	No concerns	No concerns	Low	[Imprecision]
GLPG2222_100mg:vanza_teza_deuti_10mg_100mg_150mg	0	No concerns	Low risk	No concerns	Major concerns	No concerns	No concerns	Low	[Imprecision]
GLPG2222_100mg:vanza_teza_deuti_20mg_100mg_150mg	0	No concerns	Low risk	No concerns	Major concerns	No concerns	No concerns	Low	[Imprecision]
GLPG2222_100mg:vanza_teza_deuti_5mg_100mg_150mg	0	No concerns	Low risk	No concerns	Major concerns	No concerns	No concerns	Low	[Imprecision]
GLPG2222_200mg:Iva_150mg	0	No concerns	Low risk	No concerns	Major concerns	No concerns	No concerns	Low	[Imprecision]
GLPG2222_200mg:Ola_teza_iva_200mg_100mg_150mg	0	No concerns	Low risk	No concerns	Major concerns	No concerns	No concerns	Low	[Imprecision]
GLPG2222_200mg:Ola_teza_iva_200mg_50mg_150mg	0	No concerns	Low risk	No concerns	Major concerns	No concerns	No concerns	Low	[Imprecision]
GLPG2222_200mg:Ola_teza_iva_600mg_50mg_300mg	0	No concerns	Low risk	No concerns	Major concerns	No concerns	No concerns	Low	[Imprecision]
GLPG2222_200mg:Teza_iva_100mg_150mg	0	No concerns	Low risk	No concerns	Major concerns	No concerns	No concerns	Low	[Imprecision]
GLPG2222_200mg:Teza_iva_50mg_150mg	0	No concerns	Low risk	No concerns	Major concerns	No concerns	No concerns	Low	[Imprecision]
GLPG2222_200mg:Teza_iva_50mg_300mg	0	No concerns	Low risk	No concerns	No concerns	Major concerns	No concerns	Low	[Heterogeneity]
GLPG2222_200mg:VX152_teza_iva_100mg_100mg_150mg	0	No concerns	Low risk	No concerns	Major concerns	No concerns	No concerns	Low	[Imprecision]
GLPG2222_200mg:VX152_teza_iva_200mg_100mg_150mg	0	No concerns	Low risk	No concerns	Major concerns	No concerns	No concerns	Low	[Imprecision]

GLPG2222_200mg:VX152_teza_iva_300mg_100mg_150mg	0	No concerns	Low risk	No concerns	Major concerns	No concerns	No concerns	Low	[Imprecision]
GLPG2222_200mg:luma_100mg	0	Some concerns	Low risk	No concerns	Major concerns	No concerns	No concerns	Low	[Within-study bias| Imprecision]
GLPG2222_200mg:luma_200mg	0	No concerns	Low risk	No concerns	Major concerns	No concerns	No concerns	Low	[Imprecision]
GLPG2222_200mg:luma_25mg	0	No concerns	Low risk	No concerns	Major concerns	No concerns	No concerns	Low	[Imprecision]
GLPG2222_200mg:luma_50mg	0	Some concerns	Low risk	No concerns	Major concerns	No concerns	No concerns	Low	[Within-study bias| Imprecision]
GLPG2222_200mg:luma_iva_200mg_250mg	0	No concerns	Low risk	No concerns	Major concerns	No concerns	No concerns	Low	[Imprecision]
GLPG2222_200mg:luma_iva_400mg_250mg	0	No concerns	Low risk	No concerns	Major concerns	No concerns	No concerns	Low	[Imprecision]
GLPG2222_200mg:luma_iva_600mg_250mg	0	No concerns	Low risk	No concerns	Major concerns	No concerns	No concerns	Low	[Imprecision]
GLPG2222_200mg:vanza_teza_deuti_10mg_100mg_150mg	0	No concerns	Low risk	No concerns	Major concerns	No concerns	No concerns	Low	[Imprecision]
GLPG2222_200mg:vanza_teza_deuti_20mg_100mg_150mg	0	No concerns	Low risk	No concerns	Major concerns	No concerns	No concerns	Low	[Imprecision]
GLPG2222_200mg:vanza_teza_deuti_5mg_100mg_150mg	0	No concerns	Low risk	No concerns	Major concerns	No concerns	No concerns	Low	[Imprecision]
GLPG2222_400mg:Iva_150mg	0	No concerns	Low risk	No concerns	Major concerns	No concerns	No concerns	Low	[Imprecision]
GLPG2222_400mg:Ola_teza_iva_200mg_100mg_150mg	0	No concerns	Low risk	No concerns	Major concerns	No concerns	No concerns	Low	[Imprecision]
GLPG2222_400mg:Ola_teza_iva_200mg_50mg_150mg	0	No concerns	Low risk	No concerns	Major concerns	No concerns	No concerns	Low	[Imprecision]
GLPG2222_400mg:Ola_teza_iva_600mg_50mg_300mg	0	No concerns	Low risk	No concerns	Major concerns	No concerns	No concerns	Low	[Imprecision]
GLPG2222_400mg:Teza_iva_100mg_150mg	0	No concerns	Low risk	No concerns	Major concerns	No concerns	No concerns	Low	[Imprecision]
GLPG2222_400mg:Teza_iva_50mg_150mg	0	No concerns	Low risk	No concerns	Major concerns	No concerns	No concerns	Low	[Imprecision]
GLPG2222_400mg:Teza_iva_50mg_300mg	0	No concerns	Low risk	No concerns	No concerns	Major concerns	No concerns	Low	[Heterogeneity]
GLPG2222_400mg:VX152_teza_iva_100mg_100mg_150mg	0	No concerns	Low risk	No concerns	Major concerns	No concerns	No concerns	Low	[Imprecision]
GLPG2222_400mg:VX152_teza_iva_200mg_100mg_150mg	0	No concerns	Low risk	No concerns	Major concerns	No concerns	No concerns	Low	[Imprecision]
GLPG2222_400mg:VX152_teza_iva_300mg_100mg_150mg	0	No concerns	Low risk	No concerns	Major concerns	No concerns	No concerns	Low	[Imprecision]

GLPG2222_400mg:luma_100mg	0	Some concerns	Low risk	No concerns	Major concerns	No concerns	No concerns	Low	[Within-study bias| Imprecision]
GLPG2222_400mg:luma_200mg	0	No concerns	Low risk	No concerns	Major concerns	No concerns	No concerns	Low	[Imprecision]
GLPG2222_400mg:luma_25mg	0	No concerns	Low risk	No concerns	Major concerns	No concerns	No concerns	Low	[Imprecision]
GLPG2222_400mg:luma_50mg	0	Some concerns	Low risk	No concerns	Major concerns	No concerns	No concerns	Low	[Within-study bias| Imprecision]
GLPG2222_400mg:luma_iva_200mg_250mg	0	No concerns	Low risk	No concerns	Major concerns	No concerns	No concerns	Low	[Imprecision]
GLPG2222_400mg:luma_iva_400mg_250mg	0	No concerns	Low risk	No concerns	Major concerns	No concerns	No concerns	Low	[Imprecision]
GLPG2222_400mg:luma_iva_600mg_250mg	0	No concerns	Low risk	No concerns	Major concerns	No concerns	No concerns	Low	[Imprecision]
GLPG2222_400mg:vanza_teza_deuti_10mg_100mg_150mg	0	No concerns	Low risk	No concerns	Major concerns	No concerns	No concerns	Low	[Imprecision]
GLPG2222_400mg:vanza_teza_deuti_20mg_100mg_150mg	0	No concerns	Low risk	No concerns	Major concerns	No concerns	No concerns	Low	[Imprecision]
GLPG2222_400mg:vanza_teza_deuti_5mg_100mg_150mg	0	No concerns	Low risk	No concerns	Major concerns	No concerns	No concerns	Low	[Imprecision]
GLPG2222_50mg:Iva_150mg	0	No concerns	Low risk	No concerns	Major concerns	No concerns	No concerns	Low	[Imprecision]
GLPG2222_50mg:Ola_teza_iva_200mg_100mg_150mg	0	No concerns	Low risk	No concerns	Major concerns	No concerns	No concerns	Low	[Imprecision]
GLPG2222_50mg:Ola_teza_iva_200mg_50mg_150mg	0	No concerns	Low risk	No concerns	Major concerns	No concerns	No concerns	Low	[Imprecision]
GLPG2222_50mg:Ola_teza_iva_600mg_50mg_300mg	0	No concerns	Low risk	No concerns	Major concerns	No concerns	No concerns	Low	[Imprecision]
GLPG2222_50mg:Teza_iva_100mg_150mg	0	No concerns	Low risk	No concerns	Major concerns	No concerns	No concerns	Low	[Imprecision]
GLPG2222_50mg:Teza_iva_50mg_150mg	0	No concerns	Low risk	No concerns	Major concerns	No concerns	No concerns	Low	[Imprecision]
GLPG2222_50mg:Teza_iva_50mg_300mg	0	No concerns	Low risk	No concerns	No concerns	Major concerns	No concerns	Low	[Heterogeneity]
GLPG2222_50mg:VX152_teza_iva_100mg_100mg_150mg	0	No concerns	Low risk	No concerns	Major concerns	No concerns	No concerns	Low	[Imprecision]
GLPG2222_50mg:VX152_teza_iva_200mg_100mg_150mg	0	No concerns	Low risk	No concerns	Major concerns	No concerns	No concerns	Low	[Imprecision]
GLPG2222_50mg:VX152_teza_iva_300mg_100mg_150mg	0	No concerns	Low risk	No concerns	Major concerns	No concerns	No concerns	Low	[Imprecision]
GLPG2222_50mg:luma_100mg	0	Some concerns	Low risk	No concerns	Major concerns	No concerns	No concerns	Low	[Within-study bias| Imprecision]

GLPG2222_50mg:luma_200mg	0	No concerns	Low risk	No concerns	Major concerns	No concerns	No concerns	Low	[Imprecision]
GLPG2222_50mg:luma_25mg	0	No concerns	Low risk	No concerns	Major concerns	No concerns	No concerns	Low	[Imprecision]
GLPG2222_50mg:luma_50mg	0	Some concerns	Low risk	No concerns	Major concerns	No concerns	No concerns	Low	[Within-study bias| Imprecision]
GLPG2222_50mg:luma_iva_200mg_250mg	0	No concerns	Low risk	No concerns	Major concerns	No concerns	No concerns	Low	[Imprecision]
GLPG2222_50mg:luma_iva_400mg_250mg	0	No concerns	Low risk	No concerns	Major concerns	No concerns	No concerns	Low	[Imprecision]
GLPG2222_50mg:luma_iva_600mg_250mg	0	No concerns	Low risk	No concerns	Major concerns	No concerns	No concerns	Low	[Imprecision]
GLPG2222_50mg:vanza_teza_deuti_10mg_100mg_150mg	0	No concerns	Low risk	No concerns	Major concerns	No concerns	No concerns	Low	[Imprecision]
GLPG2222_50mg:vanza_teza_deuti_20mg_100mg_150mg	0	No concerns	Low risk	No concerns	Major concerns	No concerns	No concerns	Low	[Imprecision]
GLPG2222_50mg:vanza_teza_deuti_5mg_100mg_150mg	0	No concerns	Low risk	No concerns	Major concerns	No concerns	No concerns	Low	[Imprecision]
Iva_150mg:Ola_teza_iva_200mg_100mg_150mg	0	No concerns	Low risk	No concerns	Major concerns	No concerns	No concerns	Low	[Imprecision]
Iva_150mg:Ola_teza_iva_200mg_50mg_150mg	0	No concerns	Low risk	No concerns	Major concerns	No concerns	No concerns	Low	[Imprecision]
Iva_150mg:Ola_teza_iva_600mg_50mg_300mg	0	No concerns	Low risk	No concerns	Major concerns	No concerns	No concerns	Low	[Imprecision]
Iva_150mg:Teza_iva_50mg_150mg	0	Some concerns	Low risk	No concerns	Major concerns	No concerns	No concerns	Low	[Within-study bias| Imprecision]
Iva_150mg:Teza_iva_50mg_300mg	0	No concerns	Low risk	No concerns	Major concerns	No concerns	No concerns	Low	[Imprecision]
Iva_150mg:VX152_teza_iva_100mg_100mg_150mg	0	No concerns	Low risk	No concerns	Major concerns	No concerns	No concerns	Low	[Imprecision]
Iva_150mg:VX152_teza_iva_200mg_100mg_150mg	0	No concerns	Low risk	No concerns	Major concerns	No concerns	No concerns	Low	[Imprecision]
Iva_150mg:VX152_teza_iva_300mg_100mg_150mg	0	No concerns	Low risk	No concerns	Major concerns	No concerns	No concerns	Low	[Imprecision]
Iva_150mg:luma_100mg	0	Some concerns	Low risk	No concerns	Major concerns	No concerns	No concerns	Low	[Within-study bias| Imprecision]
Iva_150mg:luma_200mg	0	Some concerns	Low risk	No concerns	Major concerns	No concerns	No concerns	Low	[Within-study bias| Imprecision]
Iva_150mg:luma_25mg	0	Some concerns	Low risk	No concerns	Major concerns	No concerns	No concerns	Low	[Within-study bias| Imprecision]
Iva_150mg:luma_50mg	0	Some concerns	Low risk	No concerns	Major concerns	No concerns	No concerns	Low	[Within-study bias| Imprecision]

Iva_150mg:luma_iva_200mg_250mg	0	No concerns	Low risk	No concerns	Major concerns	No concerns	No concerns	Low	[Imprecision]
Iva_150mg:luma_iva_400mg_250mg	0	No concerns	Low risk	No concerns	Major concerns	No concerns	No concerns	Low	[Imprecision]
Iva_150mg:luma_iva_600mg_250mg	0	No concerns	Low risk	No concerns	Major concerns	No concerns	No concerns	Low	[Imprecision]
Iva_150mg:vanza_teza_deuti_10mg_100mg_150mg	0	No concerns	Low risk	No concerns	Major concerns	No concerns	No concerns	Low	[Imprecision]
Iva_150mg:vanza_teza_deuti_20mg_100mg_150mg	0	No concerns	Low risk	No concerns	Major concerns	No concerns	No concerns	Low	[Imprecision]
Iva_150mg:vanza_teza_deuti_5mg_100mg_150mg	0	No concerns	Low risk	No concerns	Major concerns	No concerns	No concerns	Low	[Imprecision]
Ola_teza_iva_200mg_100mg_150mg:Teza_iva_100mg_150mg	0	No concerns	Low risk	No concerns	Major concerns	No concerns	No concerns	Low	[Imprecision]
Ola_teza_iva_200mg_100mg_150mg:Teza_iva_50mg_150mg	0	No concerns	Low risk	No concerns	Major concerns	No concerns	No concerns	Low	[Imprecision]
Ola_teza_iva_200mg_100mg_150mg:Teza_iva_50mg_300mg	0	No concerns	Low risk	No concerns	Major concerns	No concerns	No concerns	Low	[Imprecision]
Ola_teza_iva_200mg_100mg_150mg:VX152_teza_iva_100mg_100mg_150mg	0	No concerns	Low risk	No concerns	Major concerns	No concerns	No concerns	Low	[Imprecision]
Ola_teza_iva_200mg_100mg_150mg:VX152_teza_iva_200mg_100mg_150mg	0	No concerns	Low risk	No concerns	Major concerns	No concerns	No concerns	Low	[Imprecision]
Ola_teza_iva_200mg_100mg_150mg:VX152_teza_iva_300mg_100mg_150mg	0	No concerns	Low risk	No concerns	Major concerns	No concerns	No concerns	Low	[Imprecision]
luma_100mg:Ola_teza_iva_200mg_100mg_150mg	0	Some concerns	Low risk	No concerns	Major concerns	No concerns	No concerns	Low	[Within-study bias| Imprecision]
luma_200mg:Ola_teza_iva_200mg_100mg_150mg	0	Some concerns	Low risk	No concerns	Major concerns	No concerns	No concerns	Low	[Within-study bias| Imprecision]
luma_25mg:Ola_teza_iva_200mg_100mg_150mg	0	No concerns	Low risk	No concerns	Major concerns	No concerns	No concerns	Low	[Imprecision]
luma_50mg:Ola_teza_iva_200mg_100mg_150mg	0	Some concerns	Low risk	No concerns	Major concerns	No concerns	No concerns	Low	[Within-study bias| Imprecision]
luma_iva_200mg_250mg:Ola_teza_iva_200mg_100mg_150mg	0	No concerns	Low risk	No concerns	Major concerns	No concerns	No concerns	Low	[Imprecision]
luma_iva_400mg_250mg:Ola_teza_iva_200mg_100mg_150mg	0	No concerns	Low risk	No concerns	Major concerns	No concerns	No concerns	Low	[Imprecision]
luma_iva_600mg_250mg:Ola_teza_iva_200mg_100mg_150mg	0	No concerns	Low risk	No concerns	Major concerns	No concerns	No concerns	Low	[Imprecision]
Ola_teza_iva_200mg_100mg_150mg:vanza_teza_deuti_10mg_100mg_150mg	0	No concerns	Low risk	No concerns	Major concerns	No concerns	No concerns	Low	[Imprecision]

Ola_teza_iva_200mg_100mg_150mg:vanza_teza_deuti_20mg_100mg_150mg	0	No concerns	Low risk	No concerns	Major concerns	No concerns	No concerns	Low	[Imprecision]
Ola_teza_iva_200mg_100mg_150mg:vanza_teza_deuti_5mg_100mg_150mg	0	No concerns	Low risk	No concerns	Major concerns	No concerns	No concerns	Low	[Imprecision]
Ola_teza_iva_200mg_50mg_150mg:Teza_iva_100mg_150mg	0	No concerns	Low risk	No concerns	Major concerns	No concerns	No concerns	Low	[Imprecision]
Ola_teza_iva_200mg_50mg_150mg:Teza_iva_50mg_150mg	0	No concerns	Low risk	No concerns	Major concerns	No concerns	No concerns	Low	[Imprecision]
Ola_teza_iva_200mg_50mg_150mg:Teza_iva_50mg_300mg	0	No concerns	Low risk	No concerns	Major concerns	No concerns	No concerns	Low	[Imprecision]
Ola_teza_iva_200mg_50mg_150mg:VX152_teza_iva_100mg_100mg_150mg	0	No concerns	Low risk	No concerns	Major concerns	No concerns	No concerns	Low	[Imprecision]
Ola_teza_iva_200mg_50mg_150mg:VX152_teza_iva_200mg_100mg_150mg	0	No concerns	Low risk	No concerns	Major concerns	No concerns	No concerns	Low	[Imprecision]
Ola_teza_iva_200mg_50mg_150mg:VX152_teza_iva_300mg_100mg_150mg	0	No concerns	Low risk	No concerns	Major concerns	No concerns	No concerns	Low	[Imprecision]
luma_100mg:Ola_teza_iva_200mg_50mg_150mg	0	Some concerns	Low risk	No concerns	Major concerns	No concerns	No concerns	Low	[Within-study bias| Imprecision]
luma_200mg:Ola_teza_iva_200mg_50mg_150mg	0	Some concerns	Low risk	No concerns	Major concerns	No concerns	No concerns	Low	[Within-study bias| Imprecision]
luma_25mg:Ola_teza_iva_200mg_50mg_150mg	0	No concerns	Low risk	No concerns	Major concerns	No concerns	No concerns	Low	[Imprecision]
luma_50mg:Ola_teza_iva_200mg_50mg_150mg	0	Some concerns	Low risk	No concerns	Major concerns	No concerns	No concerns	Low	[Within-study bias| Imprecision]
luma_iva_200mg_250mg:Ola_teza_iva_200mg_50mg_150mg	0	No concerns	Low risk	No concerns	Major concerns	No concerns	No concerns	Low	[Imprecision]
luma_iva_400mg_250mg:Ola_teza_iva_200mg_50mg_150mg	0	No concerns	Low risk	No concerns	Major concerns	No concerns	No concerns	Low	[Imprecision]
luma_iva_600mg_250mg:Ola_teza_iva_200mg_50mg_150mg	0	No concerns	Low risk	No concerns	Major concerns	No concerns	No concerns	Low	[Imprecision]
Ola_teza_iva_200mg_50mg_150mg:vanza_teza_deuti_10mg_100mg_150mg	0	No concerns	Low risk	No concerns	Major concerns	No concerns	No concerns	Low	[Imprecision]
Ola_teza_iva_200mg_50mg_150mg:vanza_teza_deuti_20mg_100mg_150mg	0	No concerns	Low risk	No concerns	Major concerns	No concerns	No concerns	Low	[Imprecision]
Ola_teza_iva_200mg_50mg_150mg:vanza_teza_deuti_5mg_100mg_150mg	0	No concerns	Low risk	No concerns	Major concerns	No concerns	No concerns	Low	[Imprecision]
Ola_teza_iva_600mg_50mg_300mg:Teza_iva_100mg_150mg	0	No concerns	Low risk	No concerns	Major concerns	No concerns	No concerns	Low	[Imprecision]

Ola_teza_iva_600mg_50mg_300mg:Teza_iva_50mg_150mg	0	No concerns	Low risk	No concerns	Major concerns	No concerns	No concerns	Low	[Imprecision]
Ola_teza_iva_600mg_50mg_300mg:VX152_teza_iva_100mg_100mg_150mg	0	No concerns	Low risk	No concerns	Major concerns	No concerns	No concerns	Low	[Imprecision]
Ola_teza_iva_600mg_50mg_300mg:VX152_teza_iva_200mg_100mg_150mg	0	No concerns	Low risk	No concerns	Major concerns	No concerns	No concerns	Low	[Imprecision]
Ola_teza_iva_600mg_50mg_300mg:VX152_teza_iva_300mg_100mg_150mg	0	No concerns	Low risk	No concerns	Major concerns	No concerns	No concerns	Low	[Imprecision]
luma_100mg:Ola_teza_iva_600mg_50mg_300mg	0	Some concerns	Low risk	No concerns	Major concerns	No concerns	No concerns	Low	[Within-study bias| Imprecision]
luma_200mg:Ola_teza_iva_600mg_50mg_300mg	0	Some concerns	Low risk	No concerns	Major concerns	No concerns	No concerns	Low	[Within-study bias| Imprecision]
luma_25mg:Ola_teza_iva_600mg_50mg_300mg	0	Some concerns	Low risk	No concerns	Major concerns	No concerns	No concerns	Low	[Within-study bias| Imprecision]
luma_50mg:Ola_teza_iva_600mg_50mg_300mg	0	Some concerns	Low risk	No concerns	Major concerns	No concerns	No concerns	Low	[Within-study bias| Imprecision]
luma_iva_200mg_250mg:Ola_teza_iva_600mg_50mg_300mg	0	No concerns	Low risk	No concerns	Major concerns	No concerns	No concerns	Low	[Imprecision]
luma_iva_400mg_250mg:Ola_teza_iva_600mg_50mg_300mg	0	No concerns	Low risk	No concerns	Major concerns	No concerns	No concerns	Low	[Imprecision]
luma_iva_600mg_250mg:Ola_teza_iva_600mg_50mg_300mg	0	No concerns	Low risk	No concerns	Major concerns	No concerns	No concerns	Low	[Imprecision]
Ola_teza_iva_600mg_50mg_300mg:vanza_teza_deuti_10mg_100mg_150mg	0	No concerns	Low risk	No concerns	Major concerns	No concerns	No concerns	Low	[Imprecision]
Ola_teza_iva_600mg_50mg_300mg:vanza_teza_deuti_20mg_100mg_150mg	0	No concerns	Low risk	No concerns	Major concerns	No concerns	No concerns	Low	[Imprecision]
Ola_teza_iva_600mg_50mg_300mg:vanza_teza_deuti_5mg_100mg_150mg	0	No concerns	Low risk	No concerns	Major concerns	No concerns	No concerns	Low	[Imprecision]
Placebo:Teza_iva_50mg_150mg	0	Some concerns	Low risk	No concerns	Major concerns	No concerns	No concerns	Low	[Within-study bias| Imprecision]
Placebo:Teza_iva_50mg_300mg	0	No concerns	Low risk	No concerns	Major concerns	No concerns	No concerns	Low	[Imprecision]
Teza_iva_100mg_150mg:Teza_iva_50mg_300mg	0	No concerns	Low risk	No concerns	No concerns	Major concerns	No concerns	Low	[Heterogeneity]
Teza_iva_100mg_150mg:VX152_teza_iva_100mg_100mg_150mg	0	No concerns	Low risk	No concerns	Major concerns	No concerns	No concerns	Low	[Imprecision]

Teza_iva_100mg_150mg:VX152_teza_iva_200mg_100mg_150mg	0	No concerns	Low risk	No concerns	Major concerns	No concerns	No concerns	Low	[Imprecision]
Teza_iva_100mg_150mg:VX152_teza_iva_300mg_100mg_150mg	0	No concerns	Low risk	No concerns	Major concerns	No concerns	No concerns	Low	[Imprecision]
luma_100mg:Teza_iva_100mg_150mg	0	Some concerns	Low risk	No concerns	Major concerns	No concerns	No concerns	Low	[Within-study bias| Imprecision]
luma_200mg:Teza_iva_100mg_150mg	0	Some concerns	Low risk	No concerns	Major concerns	No concerns	No concerns	Low	[Within-study bias| Imprecision]
luma_25mg:Teza_iva_100mg_150mg	0	Some concerns	Low risk	No concerns	No concerns	Major concerns	No concerns	Low	[Within-study bias| Heterogeneity]
luma_50mg:Teza_iva_100mg_150mg	0	Some concerns	Low risk	No concerns	Major concerns	No concerns	No concerns	Low	[Within-study bias| Imprecision]
luma_iva_200mg_250mg:Teza_iva_100mg_150mg	0	No concerns	Low risk	No concerns	Major concerns	No concerns	No concerns	Low	[Imprecision]
luma_iva_400mg_250mg:Teza_iva_100mg_150mg	0	No concerns	Low risk	No concerns	Major concerns	No concerns	No concerns	Low	[Imprecision]
luma_iva_600mg_250mg:Teza_iva_100mg_150mg	0	No concerns	Low risk	No concerns	Major concerns	No concerns	No concerns	Low	[Imprecision]
Teza_iva_100mg_150mg:vanza_teza_deuti_10mg_100mg_150mg	0	No concerns	Low risk	No concerns	Major concerns	No concerns	No concerns	Low	[Imprecision]
Teza_iva_100mg_150mg:vanza_teza_deuti_20mg_100mg_150mg	0	No concerns	Low risk	No concerns	Major concerns	No concerns	No concerns	Low	[Imprecision]
Teza_iva_100mg_150mg:vanza_teza_deuti_5mg_100mg_150mg	0	No concerns	Low risk	No concerns	Major concerns	No concerns	No concerns	Low	[Imprecision]
Teza_iva_50mg_150mg:Teza_iva_50mg_300mg	0	No concerns	Low risk	No concerns	Major concerns	No concerns	No concerns	Low	[Imprecision]
Teza_iva_50mg_150mg:VX152_teza_iva_100mg_100mg_150mg	0	No concerns	Low risk	No concerns	Major concerns	No concerns	No concerns	Low	[Imprecision]
Teza_iva_50mg_150mg:VX152_teza_iva_200mg_100mg_150mg	0	No concerns	Low risk	No concerns	Major concerns	No concerns	No concerns	Low	[Imprecision]
Teza_iva_50mg_150mg:VX152_teza_iva_300mg_100mg_150mg	0	No concerns	Low risk	No concerns	Major concerns	No concerns	No concerns	Low	[Imprecision]
luma_100mg:Teza_iva_50mg_150mg	0	Some concerns	Low risk	No concerns	Major concerns	No concerns	No concerns	Low	[Within-study bias| Imprecision]
luma_200mg:Teza_iva_50mg_150mg	0	Some concerns	Low risk	No concerns	Major concerns	No concerns	No concerns	Low	[Within-study bias| Imprecision]
luma_25mg:Teza_iva_50mg_150mg	0	Some concerns	Low risk	No concerns	Major concerns	No concerns	No concerns	Low	[Within-study bias| Imprecision]

luma_50mg:Teza_iva_50mg_150mg	0	Some concerns	Low risk	No concerns	Major concerns	No concerns	No concerns	Low	[Within-study bias| Imprecision]
luma_iva_200mg_250mg:Teza_iva_50mg_150mg	0	No concerns	Low risk	No concerns	Major concerns	No concerns	No concerns	Low	[Imprecision]
luma_iva_400mg_250mg:Teza_iva_50mg_150mg	0	Some concerns	Low risk	No concerns	Major concerns	No concerns	No concerns	Low	[Within-study bias| Imprecision]
luma_iva_600mg_250mg:Teza_iva_50mg_150mg	0	No concerns	Low risk	No concerns	Major concerns	No concerns	No concerns	Low	[Imprecision]
Teza_iva_50mg_150mg:vanza_teza_deuti_10mg_100mg_150mg	0	No concerns	Low risk	No concerns	Major concerns	No concerns	No concerns	Low	[Imprecision]
Teza_iva_50mg_150mg:vanza_teza_deuti_20mg_100mg_150mg	0	No concerns	Low risk	No concerns	Major concerns	No concerns	No concerns	Low	[Imprecision]
Teza_iva_50mg_150mg:vanza_teza_deuti_5mg_100mg_150mg	0	No concerns	Low risk	No concerns	Major concerns	No concerns	No concerns	Low	[Imprecision]
Teza_iva_50mg_300mg:VX152_teza_iva_100mg_100mg_150mg	0	No concerns	Low risk	No concerns	Major concerns	No concerns	No concerns	Low	[Imprecision]
Teza_iva_50mg_300mg:VX152_teza_iva_200mg_100mg_150mg	0	No concerns	Low risk	No concerns	No concerns	Major concerns	No concerns	Low	[Heterogeneity]
Teza_iva_50mg_300mg:VX152_teza_iva_300mg_100mg_150mg	0	No concerns	Low risk	No concerns	Major concerns	No concerns	No concerns	Low	[Imprecision]
luma_100mg:Teza_iva_50mg_300mg	0	No concerns	Low risk	No concerns	Major concerns	No concerns	No concerns	Low	[Imprecision]
luma_200mg:Teza_iva_50mg_300mg	0	No concerns	Low risk	No concerns	Major concerns	No concerns	No concerns	Low	[Imprecision]
luma_25mg:Teza_iva_50mg_300mg	0	No concerns	Low risk	No concerns	Major concerns	No concerns	No concerns	Low	[Imprecision]
luma_50mg:Teza_iva_50mg_300mg	0	No concerns	Low risk	No concerns	Major concerns	No concerns	No concerns	Low	[Imprecision]
luma_iva_200mg_250mg:Teza_iva_50mg_300mg	0	No concerns	Low risk	No concerns	No concerns	Major concerns	No concerns	Low	[Heterogeneity]
luma_iva_400mg_250mg:Teza_iva_50mg_300mg	0	No concerns	Low risk	No concerns	Major concerns	No concerns	No concerns	Low	[Imprecision]
luma_iva_600mg_250mg:Teza_iva_50mg_300mg	0	No concerns	Low risk	No concerns	Major concerns	No concerns	No concerns	Low	[Imprecision]
Teza_iva_50mg_300mg:vanza_teza_deuti_10mg_100mg_150mg	0	No concerns	Low risk	No concerns	Major concerns	No concerns	No concerns	Low	[Imprecision]
Teza_iva_50mg_300mg:vanza_teza_deuti_20mg_100mg_150mg	0	No concerns	Low risk	No concerns	Major concerns	No concerns	No concerns	Low	[Imprecision]
Teza_iva_50mg_300mg:vanza_teza_deuti_5mg_100mg_150mg	0	No concerns	Low risk	No concerns	Major concerns	No concerns	No concerns	Low	[Imprecision]
luma_100mg:VX152_teza_iva_100mg_100mg_150mg	0	Some concerns	Low risk	No concerns	Major concerns	No concerns	No concerns	Low	[Within-study bias| Imprecision]

luma_200mg:VX152_teza_iva_100mg_100mg_150 mg	0	Some concerns	Low risk	No concerns	Major concerns	No concerns	No concerns	Low	[Within-study bias| Imprecision]
luma_25mg:VX152_teza_iva_100mg_100mg_150 mg	0	Some concerns	Low risk	No concerns	Major concerns	No concerns	No concerns	Low	[Within-study bias| Imprecision]
luma_50mg:VX152_teza_iva_100mg_100mg_150 mg	0	Some concerns	Low risk	No concerns	Major concerns	No concerns	No concerns	Low	[Within-study bias| Imprecision]
luma_iva_200mg_250mg:VX152_teza_iva_100mg _100mg_150mg	0	No concerns	Low risk	No concerns	Major concerns	No concerns	No concerns	Low	[Imprecision]
luma_iva_400mg_250mg:VX152_teza_iva_100mg _100mg_150mg	0	No concerns	Low risk	No concerns	Major concerns	No concerns	No concerns	Low	[Imprecision]
luma_iva_600mg_250mg:VX152_teza_iva_100mg _100mg_150mg	0	No concerns	Low risk	No concerns	Major concerns	No concerns	No concerns	Low	[Imprecision]
vanza_teza_deuti_10mg_100mg_150mg:VX152_te za_iva_100mg_100mg_150mg	0	No concerns	Low risk	No concerns	Major concerns	No concerns	No concerns	Low	[Imprecision]
vanza_teza_deuti_20mg_100mg_150mg:VX152_te za_iva_100mg_100mg_150mg	0	No concerns	Low risk	No concerns	Major concerns	No concerns	No concerns	Low	[Imprecision]
vanza_teza_deuti_5mg_100mg_150mg:VX152_tez a_iva_100mg_100mg_150mg	0	No concerns	Low risk	No concerns	Major concerns	No concerns	No concerns	Low	[Imprecision]
luma_100mg:VX152_teza_iva_200mg_100mg_150 mg	0	Some concerns	Low risk	No concerns	Major concerns	No concerns	No concerns	Low	[Within-study bias| Imprecision]
luma_200mg:VX152_teza_iva_200mg_100mg_150 mg	0	Some concerns	Low risk	No concerns	Major concerns	No concerns	No concerns	Low	[Within-study bias| Imprecision]
luma_25mg:VX152_teza_iva_200mg_100mg_150 mg	0	Some concerns	Low risk	No concerns	Major concerns	No concerns	No concerns	Low	[Within-study bias| Imprecision]
luma_50mg:VX152_teza_iva_200mg_100mg_150 mg	0	Some concerns	Low risk	No concerns	Major concerns	No concerns	No concerns	Low	[Within-study bias| Imprecision]
luma_iva_200mg_250mg:VX152_teza_iva_200mg _100mg_150mg	0	No concerns	Low risk	No concerns	Major concerns	No concerns	No concerns	Low	[Imprecision]
luma_iva_400mg_250mg:VX152_teza_iva_200mg _100mg_150mg	0	No concerns	Low risk	No concerns	Major concerns	No concerns	No concerns	Low	[Imprecision]
luma_iva_600mg_250mg:VX152_teza_iva_200mg _100mg_150mg	0	No concerns	Low risk	No concerns	Major concerns	No concerns	No concerns	Low	[Imprecision]
vanza_teza_deuti_10mg_100mg_150mg:VX152_te za_iva_200mg_100mg_150mg	0	No concerns	Low risk	No concerns	Major concerns	No concerns	No concerns	Low	[Imprecision]
vanza_teza_deuti_20mg_100mg_150mg:VX152_te za_iva_200mg_100mg_150mg	0	No concerns	Low risk	No concerns	Major concerns	No concerns	No concerns	Low	[Imprecision]

vanza_teza_deuti_5mg_100mg_150mg:VX152_teza_iva_200mg_100mg_150mg	0	No concerns	Low risk	No concerns	Major concerns	No concerns	No concerns	Low	[Imprecision]
luma_100mg:VX152_teza_iva_300mg_100mg_150mg	0	Some concerns	Low risk	No concerns	Major concerns	No concerns	No concerns	Low	[Within-study bias| Imprecision]
luma_200mg:VX152_teza_iva_300mg_100mg_150mg	0	Some concerns	Low risk	No concerns	Major concerns	No concerns	No concerns	Low	[Within-study bias| Imprecision]
luma_25mg:VX152_teza_iva_300mg_100mg_150mg	0	Some concerns	Low risk	No concerns	Major concerns	No concerns	No concerns	Low	[Within-study bias| Imprecision]
luma_50mg:VX152_teza_iva_300mg_100mg_150mg	0	Some concerns	Low risk	No concerns	Major concerns	No concerns	No concerns	Low	[Within-study bias| Imprecision]
luma_iva_200mg_250mg:VX152_teza_iva_300mg_100mg_150mg	0	No concerns	Low risk	No concerns	Major concerns	No concerns	No concerns	Low	[Imprecision]
luma_iva_400mg_250mg:VX152_teza_iva_300mg_100mg_150mg	0	No concerns	Low risk	No concerns	Major concerns	No concerns	No concerns	Low	[Imprecision]
luma_iva_600mg_250mg:VX152_teza_iva_300mg_100mg_150mg	0	No concerns	Low risk	No concerns	Major concerns	No concerns	No concerns	Low	[Imprecision]
vanza_teza_deuti_10mg_100mg_150mg:VX152_teza_iva_300mg_100mg_150mg	0	No concerns	Low risk	No concerns	Major concerns	No concerns	No concerns	Low	[Imprecision]
vanza_teza_deuti_20mg_100mg_150mg:VX152_teza_iva_300mg_100mg_150mg	0	No concerns	Low risk	No concerns	Major concerns	No concerns	No concerns	Low	[Imprecision]
vanza_teza_deuti_5mg_100mg_150mg:VX152_teza_iva_300mg_100mg_150mg	0	No concerns	Low risk	No concerns	Major concerns	No concerns	No concerns	Low	[Imprecision]
luma_100mg:luma_iva_200mg_250mg	0	Some concerns	Low risk	No concerns	Major concerns	No concerns	No concerns	Low	[Within-study bias| Imprecision]
luma_100mg:luma_iva_400mg_250mg	0	Some concerns	Low risk	No concerns	Major concerns	No concerns	No concerns	Low	[Within-study bias| Imprecision]
luma_100mg:luma_iva_600mg_250mg	0	Some concerns	Low risk	No concerns	Major concerns	No concerns	No concerns	Low	[Within-study bias| Imprecision]
luma_100mg:vanza_teza_deuti_10mg_100mg_150mg	0	Some concerns	Low risk	No concerns	Major concerns	No concerns	No concerns	Low	[Within-study bias| Imprecision]
luma_100mg:vanza_teza_deuti_20mg_100mg_150mg	0	Some concerns	Low risk	No concerns	Major concerns	No concerns	No concerns	Low	[Within-study bias| Imprecision]
luma_100mg:vanza_teza_deuti_5mg_100mg_150mg	0	Some concerns	Low risk	No concerns	Major concerns	No concerns	No concerns	Low	[Within-study bias| Imprecision]

luma_200mg:luma_iva_200mg_250mg	0	Some concerns	Low risk	No concerns	Major concerns	No concerns	No concerns	Low	[Within-study bias| Imprecision]
luma_200mg:luma_iva_400mg_250mg	0	Some concerns	Low risk	No concerns	Major concerns	No concerns	No concerns	Low	[Within-study bias| Imprecision]
luma_200mg:luma_iva_600mg_250mg	0	Some concerns	Low risk	No concerns	Major concerns	No concerns	No concerns	Low	[Within-study bias| Imprecision]
luma_200mg:vanza_teza_deuti_10mg_100mg_150 mg	0	Some concerns	Low risk	No concerns	Major concerns	No concerns	No concerns	Low	[Within-study bias| Imprecision]
luma_200mg:vanza_teza_deuti_20mg_100mg_150 mg	0	Some concerns	Low risk	No concerns	Major concerns	No concerns	No concerns	Low	[Within-study bias| Imprecision]
luma_200mg:vanza_teza_deuti_5mg_100mg_150m g	0	Some concerns	Low risk	No concerns	Major concerns	No concerns	No concerns	Low	[Within-study bias| Imprecision]
luma_25mg:luma_iva_200mg_250mg	0	Some concerns	Low risk	No concerns	No concerns	Major concerns	No concerns	Low	[Within-study bias| Heterogeneity]
luma_25mg:luma_iva_400mg_250mg	0	Some concerns	Low risk	No concerns	Major concerns	No concerns	No concerns	Low	[Within-study bias| Imprecision]
luma_25mg:luma_iva_600mg_250mg	0	Some concerns	Low risk	No concerns	Major concerns	No concerns	No concerns	Low	[Within-study bias| Imprecision]
luma_25mg:vanza_teza_deuti_10mg_100mg_150m g	0	Some concerns	Low risk	No concerns	Major concerns	No concerns	No concerns	Low	[Within-study bias| Imprecision]
luma_25mg:vanza_teza_deuti_20mg_100mg_150m g	0	Some concerns	Low risk	No concerns	Major concerns	No concerns	No concerns	Low	[Within-study bias| Imprecision]
luma_25mg:vanza_teza_deuti_5mg_100mg_150mg	0	Some concerns	Low risk	No concerns	Major concerns	No concerns	No concerns	Low	[Within-study bias| Imprecision]
luma_50mg:luma_iva_200mg_250mg	0	Some concerns	Low risk	No concerns	Major concerns	No concerns	No concerns	Low	[Within-study bias| Imprecision]
luma_50mg:luma_iva_400mg_250mg	0	Some concerns	Low risk	No concerns	Major concerns	No concerns	No concerns	Low	[Within-study bias| Imprecision]
luma_50mg:luma_iva_600mg_250mg	0	Some concerns	Low risk	No concerns	Major concerns	No concerns	No concerns	Low	[Within-study bias| Imprecision]
luma_50mg:vanza_teza_deuti_10mg_100mg_150m g	0	Some concerns	Low risk	No concerns	Major concerns	No concerns	No concerns	Low	[Within-study bias| Imprecision]

luma_50mg:vanza_teza_deuti_20mg_100mg_150mg	0	Some concerns	Low risk	No concerns	Major concerns	No concerns	No concerns	Low	[Within-study bias| Imprecision]
luma_50mg:vanza_teza_deuti_5mg_100mg_150mg	0	Some concerns	Low risk	No concerns	Major concerns	No concerns	No concerns	Low	[Within-study bias| Imprecision]
luma_iva_200mg_250mg:vanza_teza_deuti_10mg_100mg_150mg	0	No concerns	Low risk	No concerns	Major concerns	No concerns	No concerns	Low	[Imprecision]
luma_iva_200mg_250mg:vanza_teza_deuti_20mg_100mg_150mg	0	No concerns	Low risk	No concerns	Major concerns	No concerns	No concerns	Low	[Imprecision]
luma_iva_200mg_250mg:vanza_teza_deuti_5mg_100mg_150mg	0	No concerns	Low risk	No concerns	Major concerns	No concerns	No concerns	Low	[Imprecision]
luma_iva_400mg_250mg:vanza_teza_deuti_10mg_100mg_150mg	0	No concerns	Low risk	No concerns	Major concerns	No concerns	No concerns	Low	[Imprecision]
luma_iva_400mg_250mg:vanza_teza_deuti_20mg_100mg_150mg	0	No concerns	Low risk	No concerns	Major concerns	No concerns	No concerns	Low	[Imprecision]
luma_iva_400mg_250mg:vanza_teza_deuti_5mg_100mg_150mg	0	No concerns	Low risk	No concerns	Major concerns	No concerns	No concerns	Low	[Imprecision]
luma_iva_600mg_250mg:vanza_teza_deuti_10mg_100mg_150mg	0	No concerns	Low risk	No concerns	Major concerns	No concerns	No concerns	Low	[Imprecision]
luma_iva_600mg_250mg:vanza_teza_deuti_20mg_100mg_150mg	0	No concerns	Low risk	No concerns	Major concerns	No concerns	No concerns	Low	[Imprecision]
luma_iva_600mg_250mg:vanza_teza_deuti_5mg_100mg_150mg	0	No concerns	Low risk	No concerns	Major concerns	No concerns	No concerns	Low	[Imprecision]

eTable 14 Certainty ratings of Serious adverse events for adults treated for greater than 8 weeks using CINEMA framework

Comparison	Number of studies	Within-study bias	Reporting bias	Indirectness	Imprecision	Heterogeneity	Incoherence	Confidence rating	Reason(s) for downgrading
Mixed evidence									
Elexa_teza_iva_200mg_100mg_150mg:Placebo	1	No concerns	Low risk	No concerns	No concerns	Major concerns	No concerns	Low	["Heterogeneity"]
Elexa_teza_iva_200mg_100mg_150mg:Teza_iva_100mg_150mg	1	No concerns	Low risk	No concerns	Major concerns	No concerns	No concerns	Low	["Imprecision"]
Elexa_teza_iva_200mg_100mg_150mg:vanza_teza_deuti_20mg_100mg_250mg	1	No concerns	Low risk	No concerns	Major concerns	No concerns	No concerns	Low	["Imprecision"]
Iva_150mg:Placebo	1	No concerns	Low risk	No concerns	Major concerns	No concerns	No concerns	Low	["Imprecision"]
Placebo:Teza_iva_100mg_150mg	3	Some concerns	Low risk	No concerns	Major concerns	No concerns	No concerns	Low	["Within-study bias","Imprecision"]
Placebo:Teza_iva_50mg_150mg	1	Some concerns	Low risk	No concerns	Major concerns	No concerns	No concerns	Low	["Within-study bias","Imprecision"]
luma_iva_400mg_250mg:Placebo	2	No concerns	Low risk	No concerns	No concerns	No concerns	No concerns	High	NA
luma_iva_600mg_250mg:Placebo	2	No concerns	Low risk	No concerns	No concerns	Major concerns	No concerns	Low	["Heterogeneity"]
luma_iva_400mg_250mg:luma_iva_600mg_250mg	3	No concerns	Low risk	No concerns	No concerns	Major concerns	No concerns	Low	["Heterogeneity"]
Indirect evidence									
Elexa_teza_iva_200mg_100mg_150mg:Iva_150mg	0	No concerns	Low risk	No concerns	Major concerns	No concerns	No concerns	Low	["Imprecision"]
Elexa_teza_iva_200mg_100mg_150mg:Teza_iva_50mg_150mg	0	Some concerns	Low risk	No concerns	Major concerns	No concerns	No concerns	Low	["Within-study bias","Imprecision"]
Elexa_teza_iva_200mg_100mg_150mg:luma_iva_400mg_250mg	0	No concerns	Low risk	No concerns	Major concerns	No concerns	No concerns	Low	["Imprecision"]
Elexa_teza_iva_200mg_100mg_150mg:luma_iva_600mg_250mg	0	No concerns	Low risk	No concerns	Major concerns	No concerns	No concerns	Low	["Imprecision"]

Iva_150mg:Teza_iva_100mg_150mg	0	No concerns	Low risk	No concerns	Major concerns	No concerns	No concerns	Low	["Imprecision"]
Iva_150mg:Teza_iva_50mg_150mg	0	Some concerns	Low risk	No concerns	Major concerns	No concerns	No concerns	Low	["Within-study bias","Imprecision"]
Iva_150mg:luma_iva_400mg_250mg	0	No concerns	Low risk	No concerns	Major concerns	No concerns	No concerns	Low	["Imprecision"]
Iva_150mg:luma_iva_600mg_250mg	0	No concerns	Low risk	No concerns	Major concerns	No concerns	No concerns	Low	["Imprecision"]
Iva_150mg:vanza_teza_deuti_20mg_100mg_250mg	0	No concerns	Low risk	No concerns	Major concerns	No concerns	No concerns	Low	["Imprecision"]
Placebo:vanza_teza_deuti_20mg_100mg_250mg	0	No concerns	Low risk	No concerns	Major concerns	No concerns	No concerns	Low	["Imprecision"]
Teza_iva_100mg_150mg:Teza_iva_50mg_150mg	0	Some concerns	Low risk	No concerns	Major concerns	No concerns	No concerns	Low	["Within-study bias","Imprecision"]
luma_iva_400mg_250mg:Teza_iva_100mg_150mg	0	No concerns	Low risk	No concerns	Major concerns	No concerns	No concerns	Low	["Imprecision"]
luma_iva_600mg_250mg:Teza_iva_100mg_150mg	0	No concerns	Low risk	No concerns	Major concerns	No concerns	No concerns	Low	["Imprecision"]
Teza_iva_100mg_150mg:vanza_teza_deuti_20mg_100mg_250mg	0	No concerns	Low risk	No concerns	No concerns	Major concerns	No concerns	Low	["Within-study bias","Heterogeneity"]
luma_iva_400mg_250mg:Teza_iva_50mg_150mg	0	No concerns	Low risk	No concerns	Major concerns	No concerns	No concerns	Low	["Imprecision"]
luma_iva_600mg_250mg:Teza_iva_50mg_150mg	0	No concerns	Low risk	No concerns	Major concerns	No concerns	No concerns	Low	["Imprecision"]
Teza_iva_50mg_150mg:vanza_teza_deuti_20mg_100mg_250mg	0	No concerns	Low risk	No concerns	Major concerns	No concerns	No concerns	Low	["Imprecision"]
luma_iva_400mg_250mg:vanza_teza_deuti_20mg_100mg_250mg	0	No concerns	Low risk	No concerns	Major concerns	No concerns	No concerns	Low	["Imprecision"]
luma_iva_600mg_250mg:vanza_teza_deuti_20mg_100mg_250mg	0	No concerns	Low risk	No concerns	Major concerns	No concerns	No concerns	Low	["Imprecision"]

	D1	D2	D3	D4	D5	Overall
Flume et al	+	+	+	+	+	+
Heijerman et al	+	+	+	+	+	+
Mall et al	!	+	+	+	+	!
McKone et al	!	+	+	+	+	!
Middleton et al	+	+	+	+	+	+
Munck et al	!	+	+	+	+	!
Taylor et al	+	+	+	+	+	+
Ratjen et al	+	+	+	+	+	+
Schwarz et al	+	+	+	+	+	+
Sutharsan et al	+	+	+	+	+	+
Barry et al.	+	+	+	+	+	+
Uluer et al	+	+	+	+	+	+
Keating et al	+	+	+	+	+	+
Donaldson et al	!	+	+	+	+	!
Davies et al.	!	+	+	+	+	!
Clancy et al.	!	+	+	+	+	!
Boyle et al.	+	+	+	+	+	+
NCT02070744	!	+	+	+	!	!
NCT02508207	+	+	+	+	+	+
Rowe et al.	!	+	+	+	+	!
Koningsbruggen et al.	+	+	+	+	+	+
Bell et al.	+	+	+	+	+	+
Rowe et al.	+	+	+	+	+	+
NCT02951195	+	+	+	+	+	+
NCT02951182	+	+	+	+	+	+
NCT03150719	+	+	+	+	+	+
Wainright et al.	+	+	+	+	+	+
Konstan et al.	+	+	+	+	+	+
Keating et al.	+	+	+	+	+	+

+ Low risk
! Some concerns
- High risk

D1 Randomisation process
D2 Deviations from the intended i
D3 Missing outcome data
D4 Measurement of the outcome
D5 Selection of the reported resul

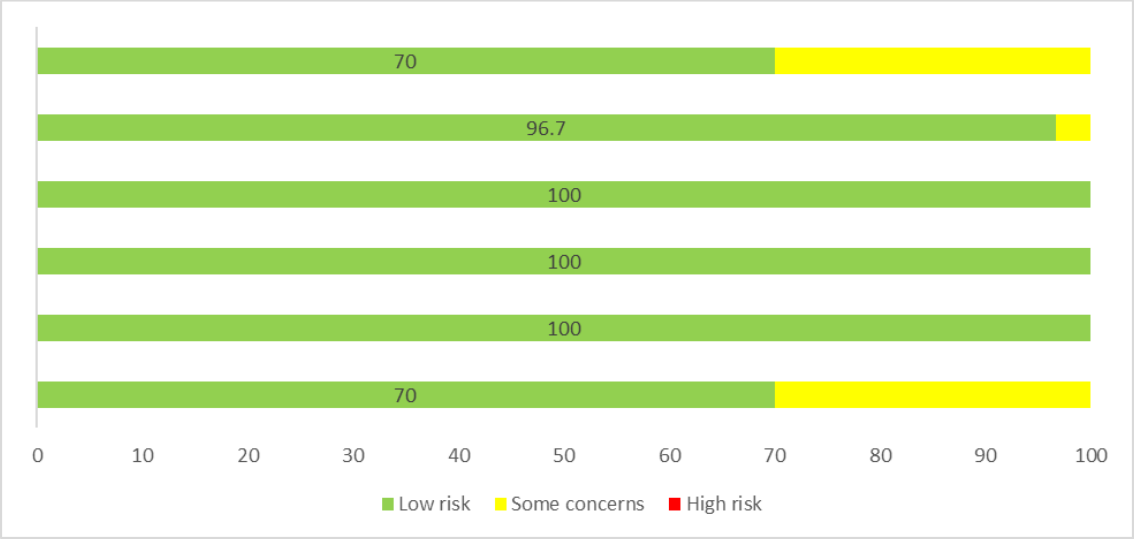

eFigure1 Summary of results from risk of bias (ROB-2) tool

A)

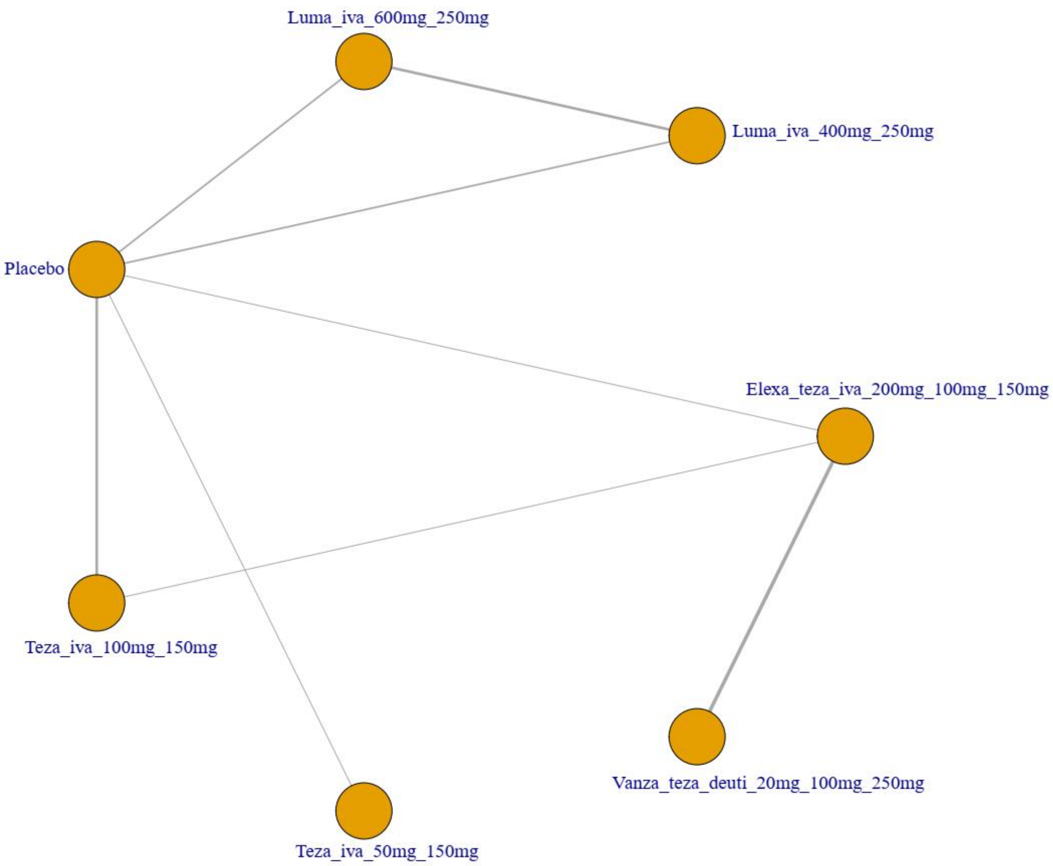
Luma_iva_600mg_250mg
Luma_iva_400mg_250mg
Placebo
Elexa_teza_iva_200mg_100mg_150mg
Teza_iva_100mg_150mg
Vanza_teza_deuti_20mg_100mg_250mg
Teza_iva_50mg_150mg

B)

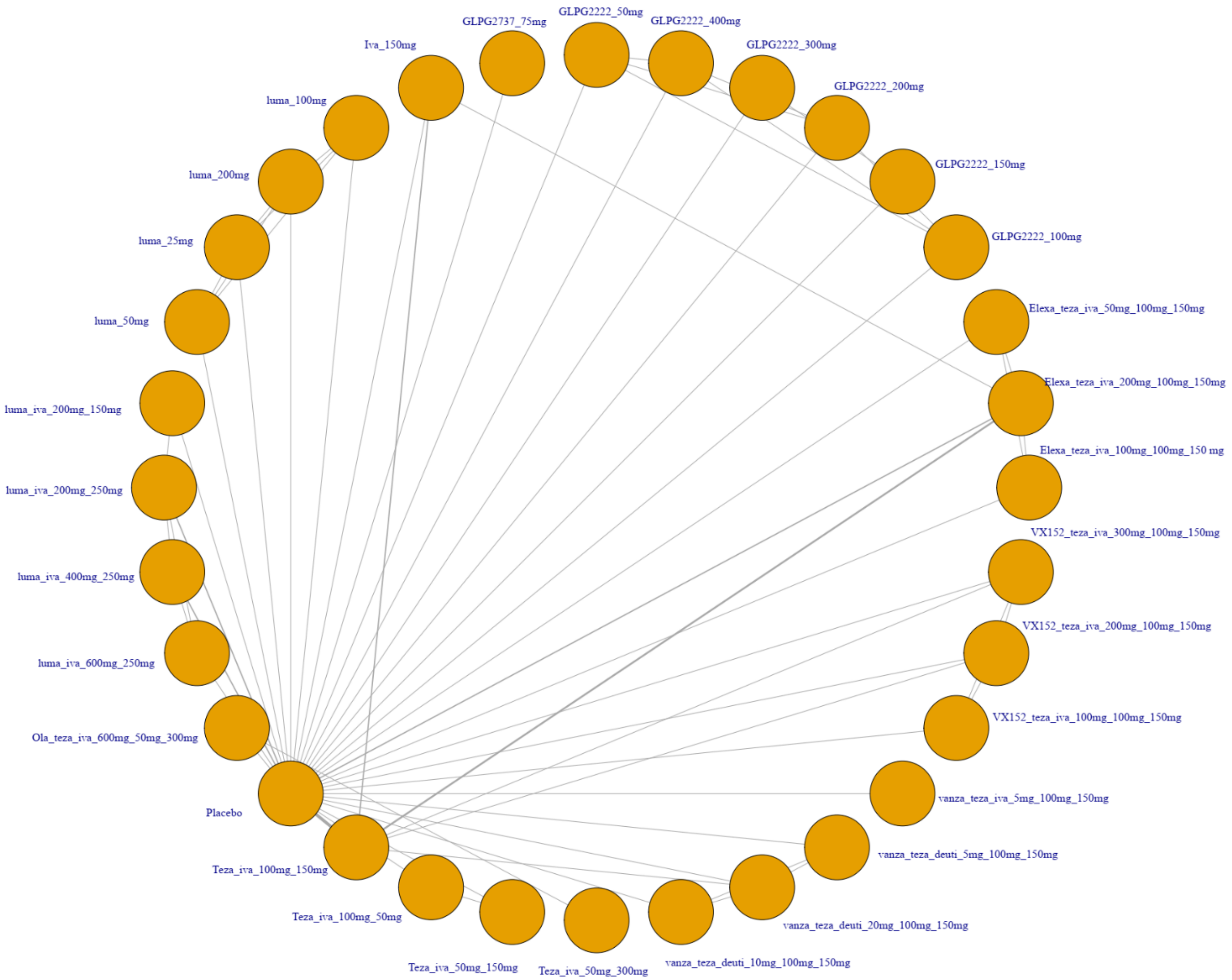

eFigure 2 Network diagrams for ppFEV$_1$ in : (A) Adults treated for greater than 8 weeks (excluding Flume et al); (B) Adults treated for 4 to 8 weeks

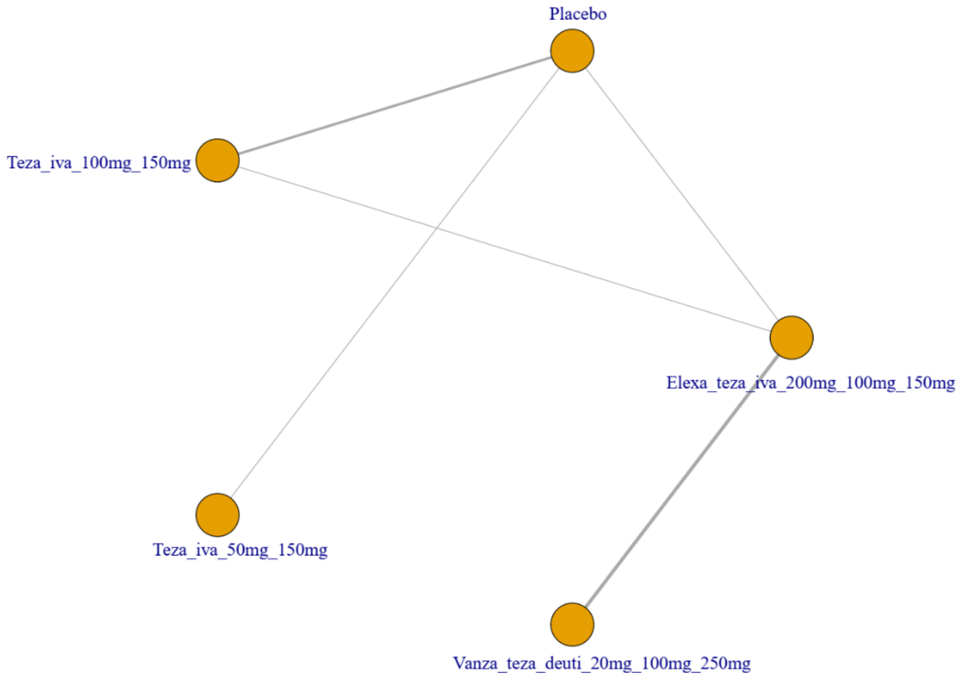
Placebo
Teza_iva_100mg_150mg
Elexa_teza_iva_200mg_100mg_150mg
Teza_iva_50mg_150mg
Vanza_teza_deuti_20mg_100mg_250mg

A)

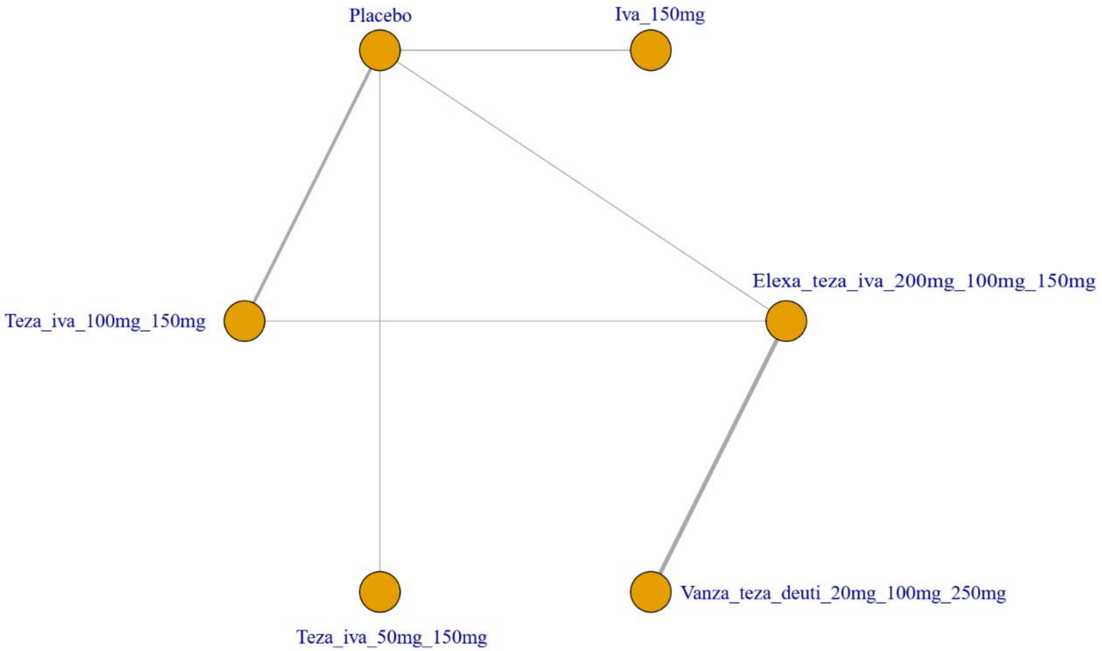
Placebo
Iva_150mg
Elexa_teza_iva_200mg_100mg_150mg
Teza_iva_100mg_150mg
Teza_iva_50mg_150mg
Vanza_teza_deuti_20mg_100mg_250mg

B)

C)

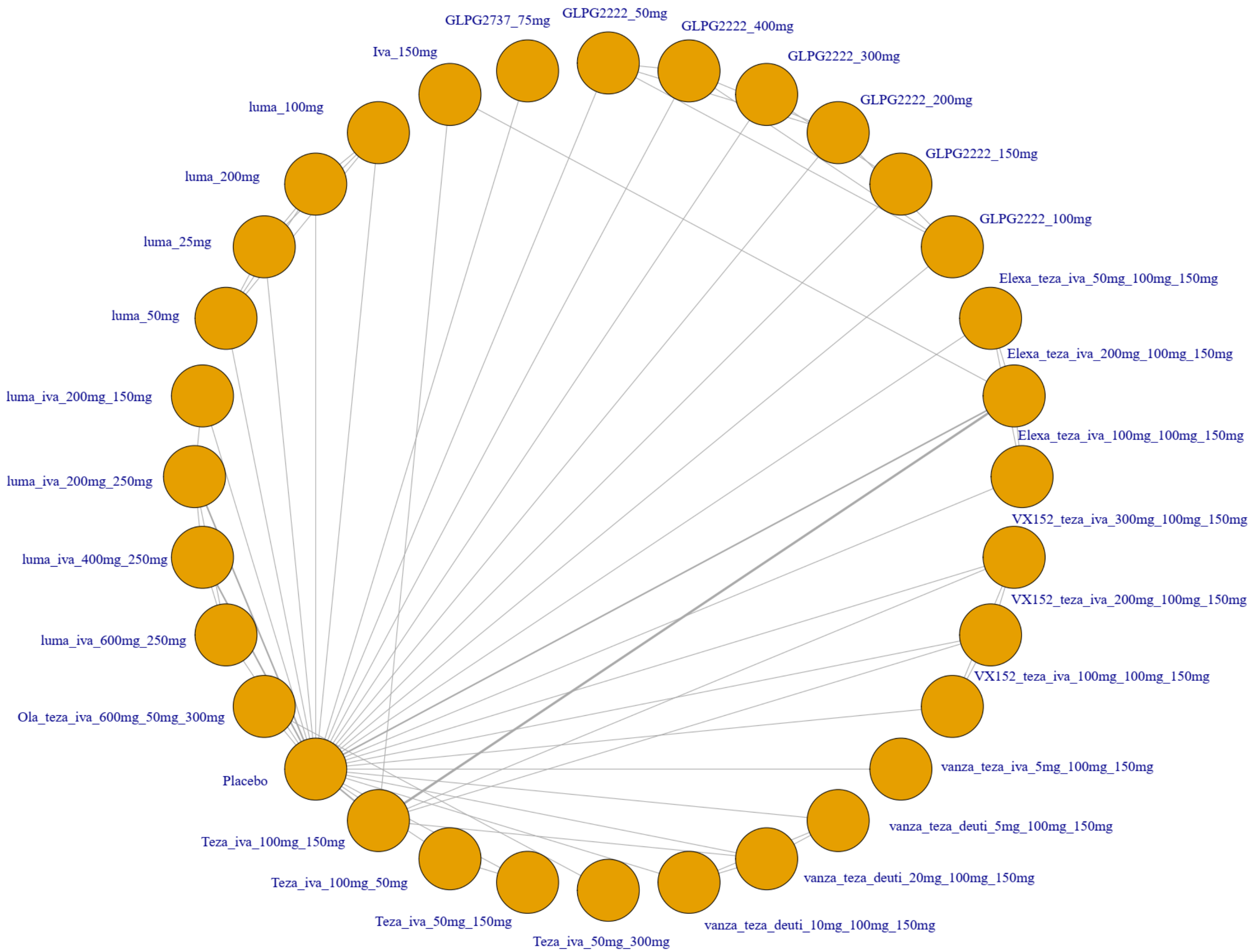

eFigure 3 Network diagrams for sweat chloride in : (A) Adults treated for greater than 8 weeks (excluding Flume et al.) (B) Adults treated for greater than 8 weeks C) Adults treated for 4 to 8 weeks

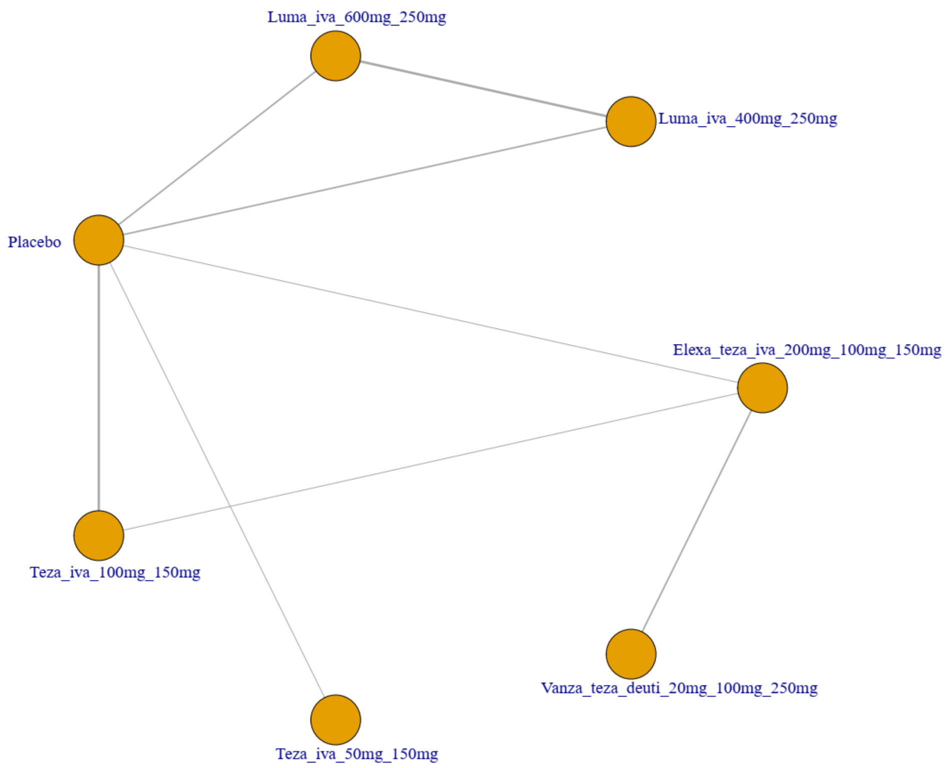
Luma_iva_600mg_250mg
Luma_iva_400mg_250mg
Placebo
Elexa_teza_iva_200mg_100mg_150mg
Teza_iva_100mg_150mg
Vanza_teza_deuti_20mg_100mg_250mg
Teza_iva_50mg_150mg

A)

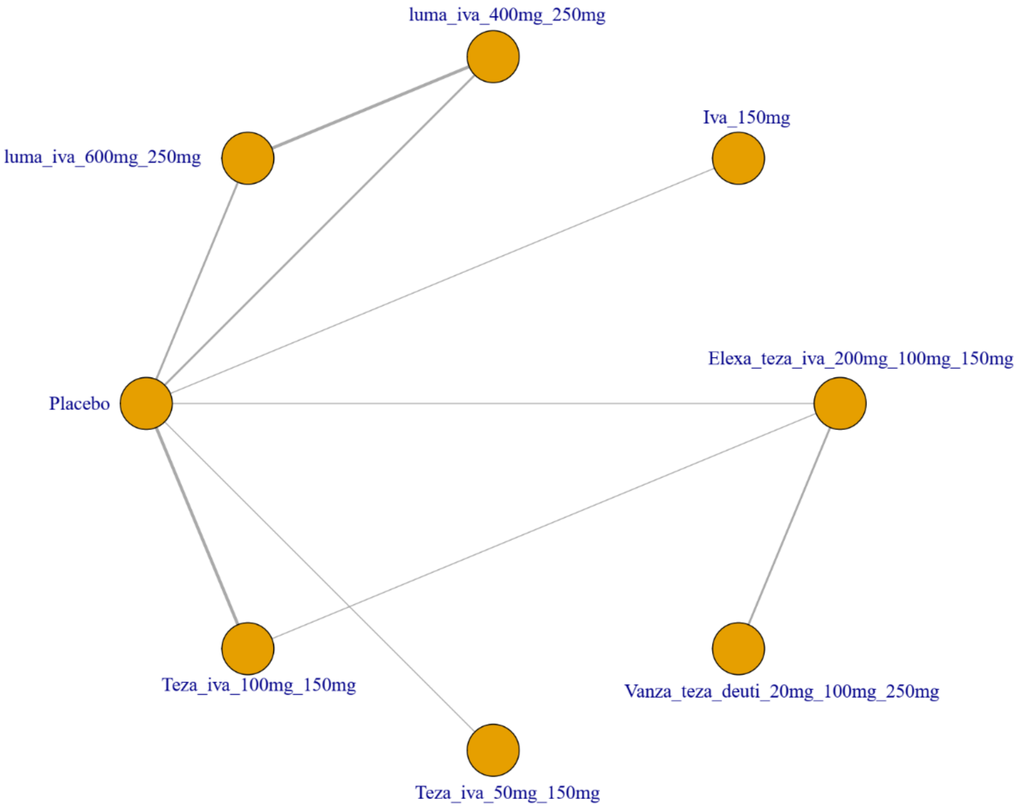
luma_iva_400mg_250mg
Iva_150mg
luma_iva_600mg_250mg
Placebo
Elexa_teza_iva_200mg_100mg_150mg
Teza_iva_100mg_150mg
Vanza_teza_deuti_20mg_100mg_250mg
Teza_iva_50mg_150mg

B)

C)

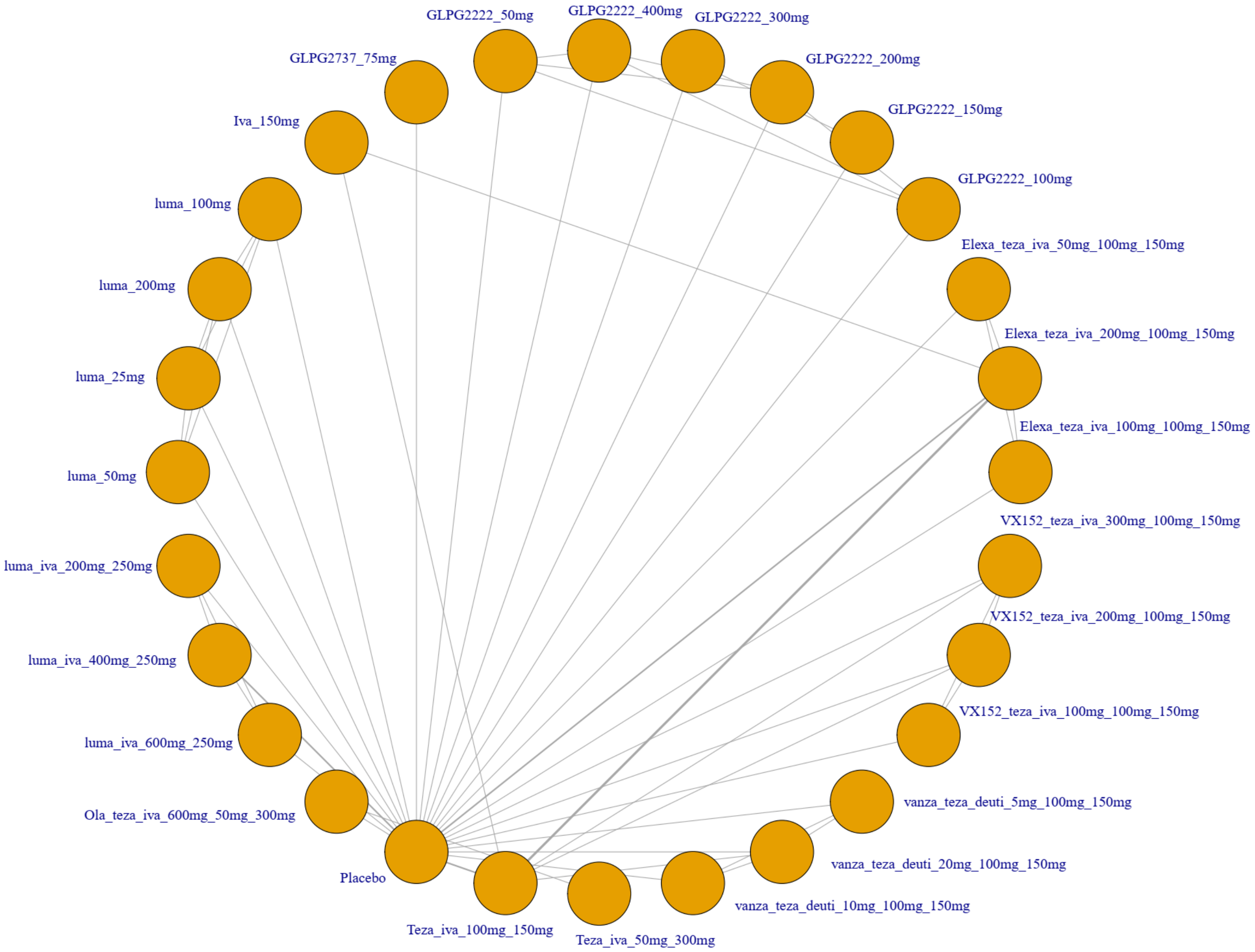

eFigure 4 Network diagrams for CFQ-R in : (A) Adults treated for greater than 8 weeks (excluding Flume et al.) (B) Adults treated for greater than 8 weeks C) Adults treated for 4 to 8 weeks

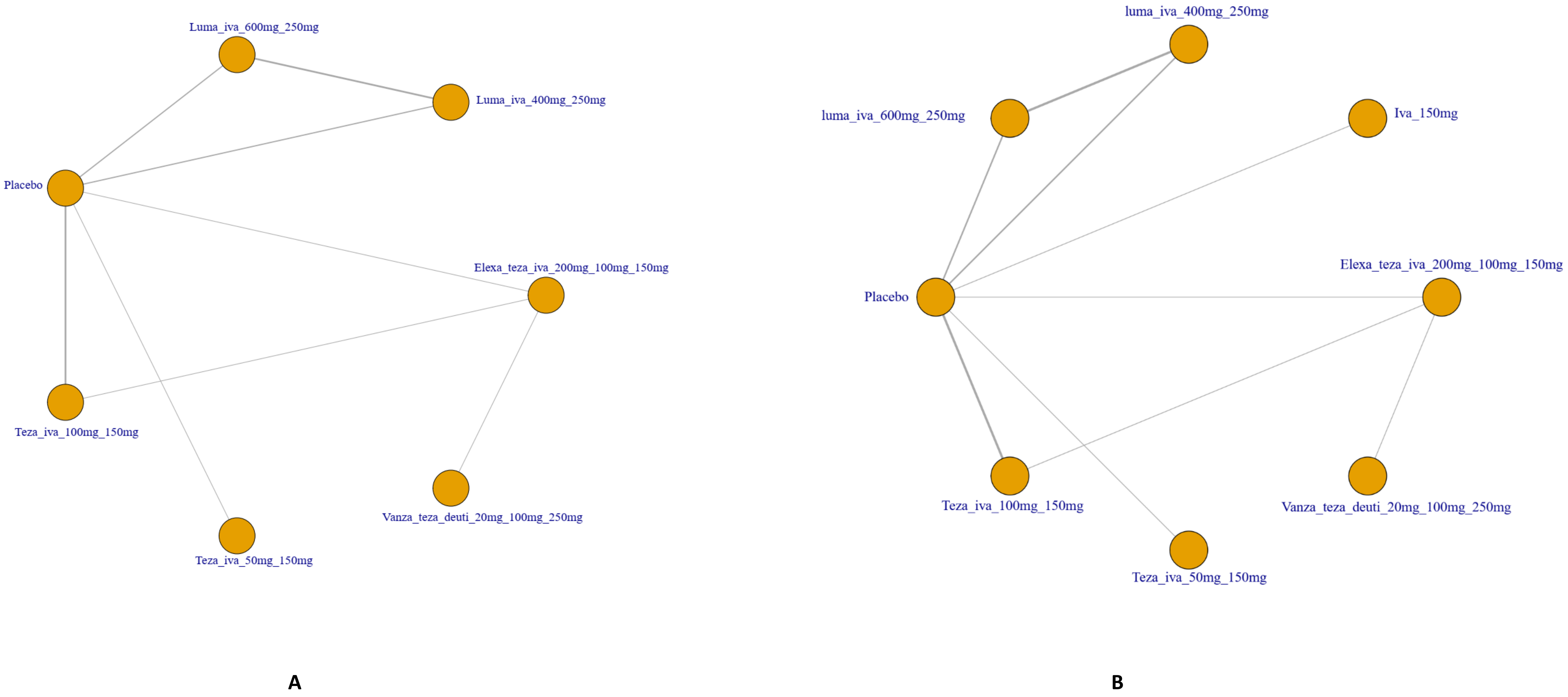

Luma_iva_600mg_250mg
Luma_iva_400mg_250mg
Placebo
Elexa_teza_iva_200mg_100mg_150mg
Teza_iva_100mg_150mg
Vanza_teza_deuti_20mg_100mg_250mg
Teza_iva_50mg_150mg
A
luma_iva_400mg_250mg
luma_iva_600mg_250mg
Iva_150mg
Placebo
Elexa_teza_iva_200mg_100mg_150mg
Teza_iva_100mg_150mg
Vanza_teza_deuti_20mg_100mg_250mg
Teza_iva_50mg_150mg
B

C)

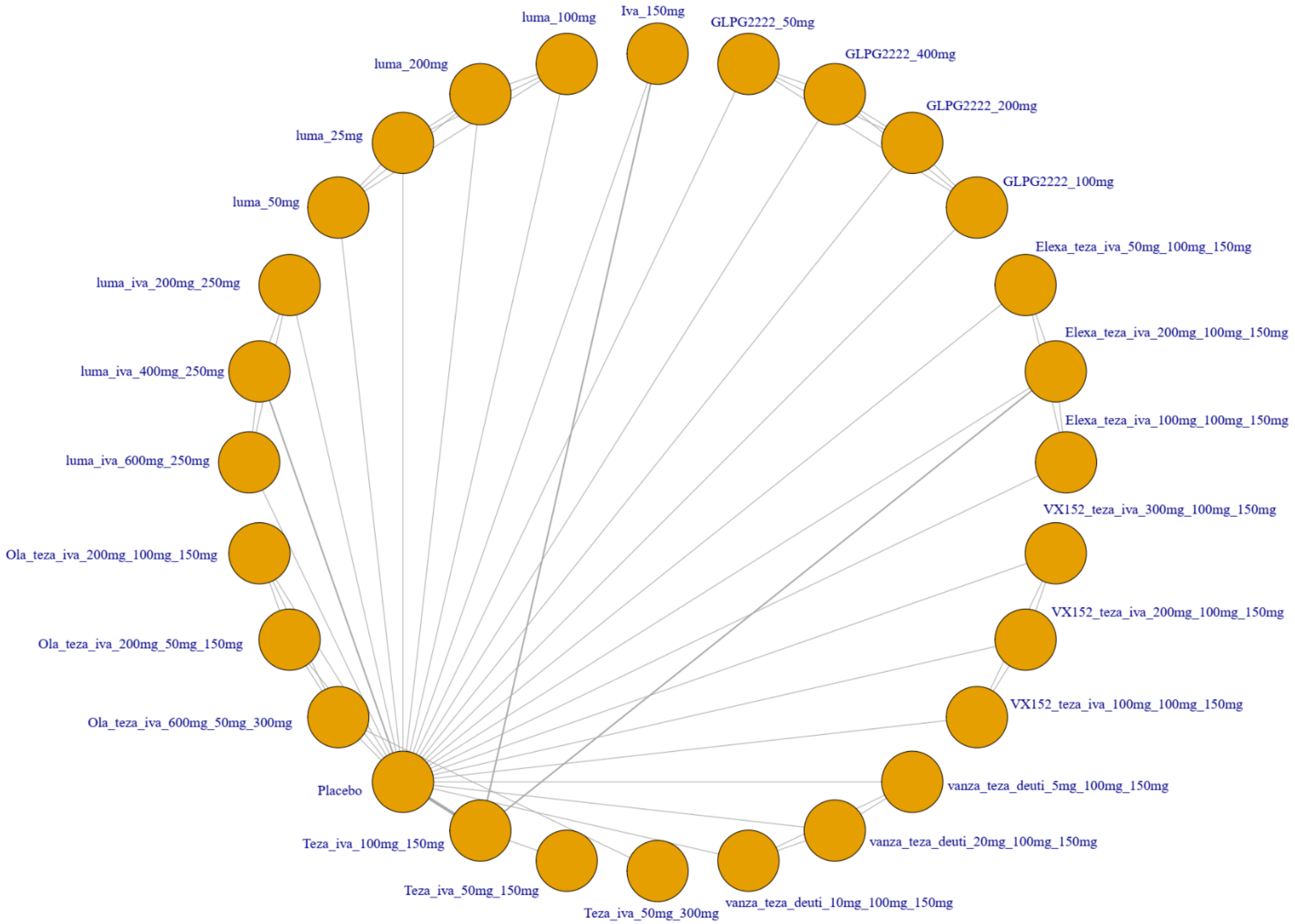

eFigure 5 Network diagrams for serious adverse events in : (A) Adults treated for greater than 8 weeks (excluding Flume et al.) (B) Adults treated for greater than 8 weeks (C) Adults treated for 4 to 8 weeks

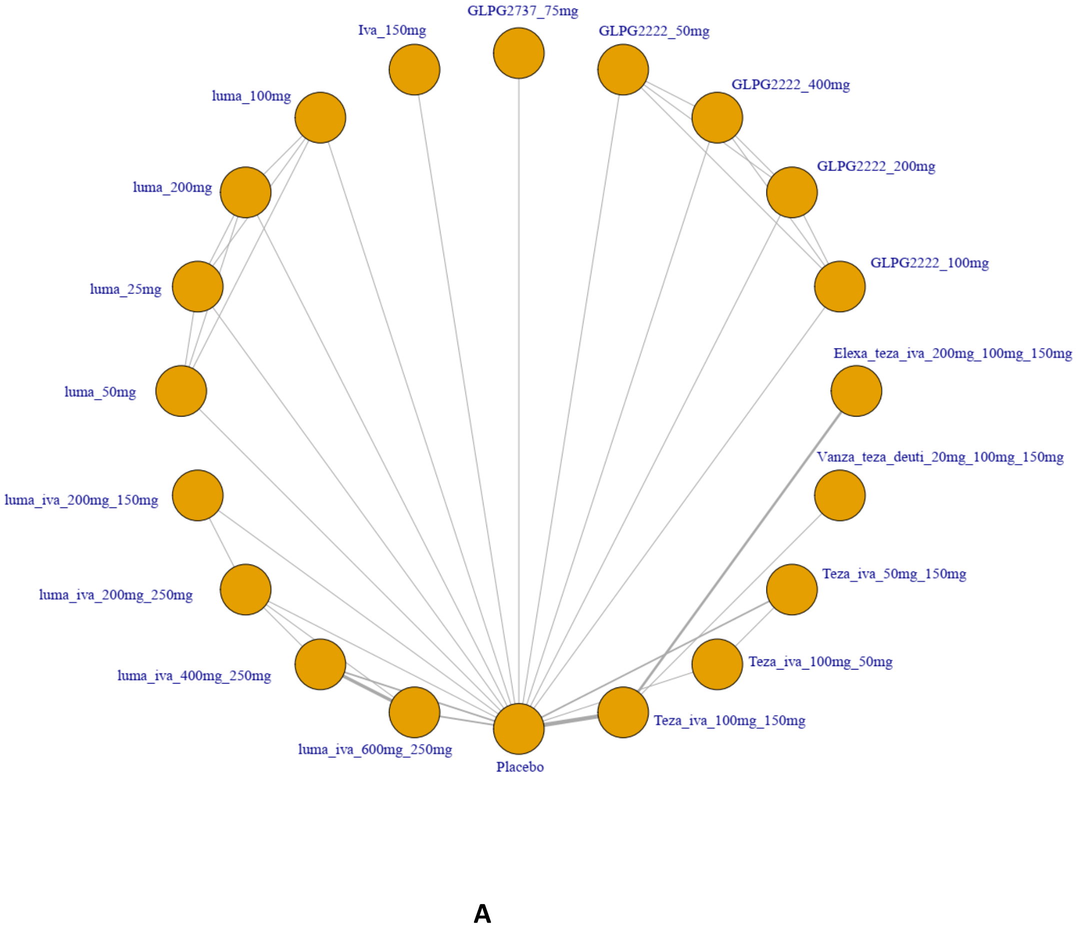

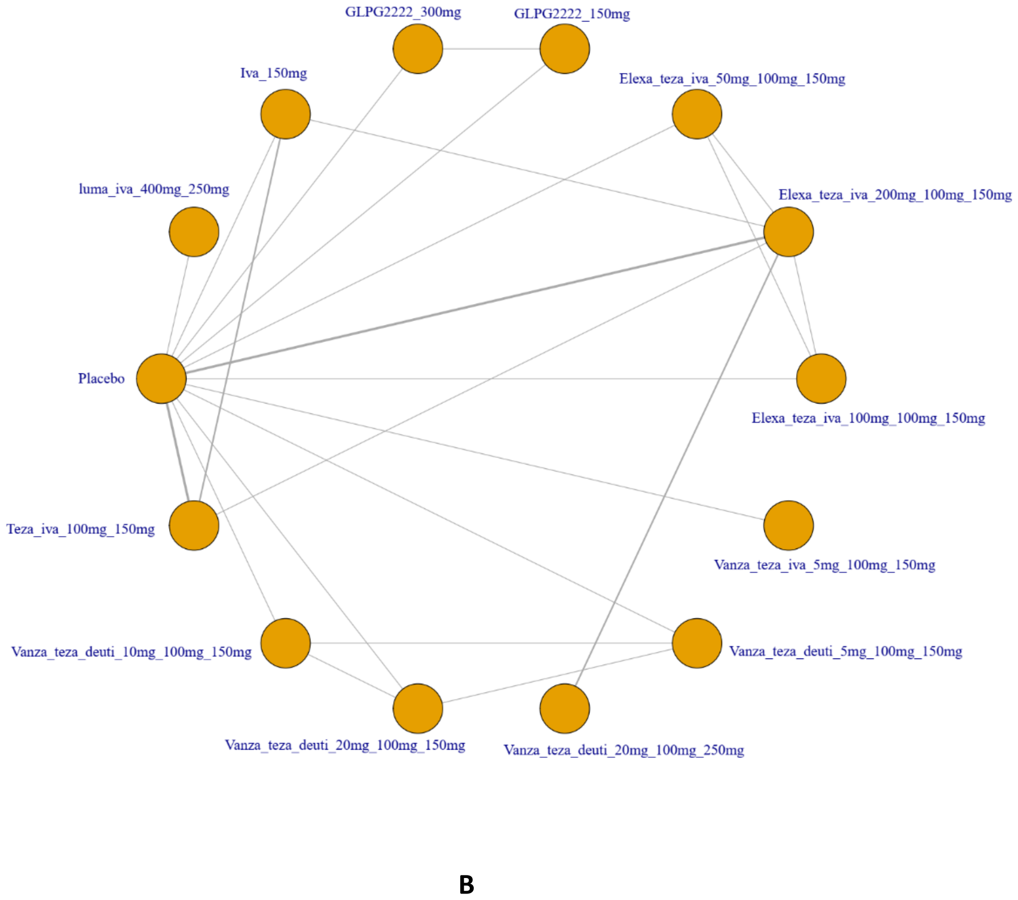

eFigure 5 Network diagrams for ppFEV1 in adults : (A) Homozygous to phe508del mutation ; (B) Heterozygous to phe508del mutation

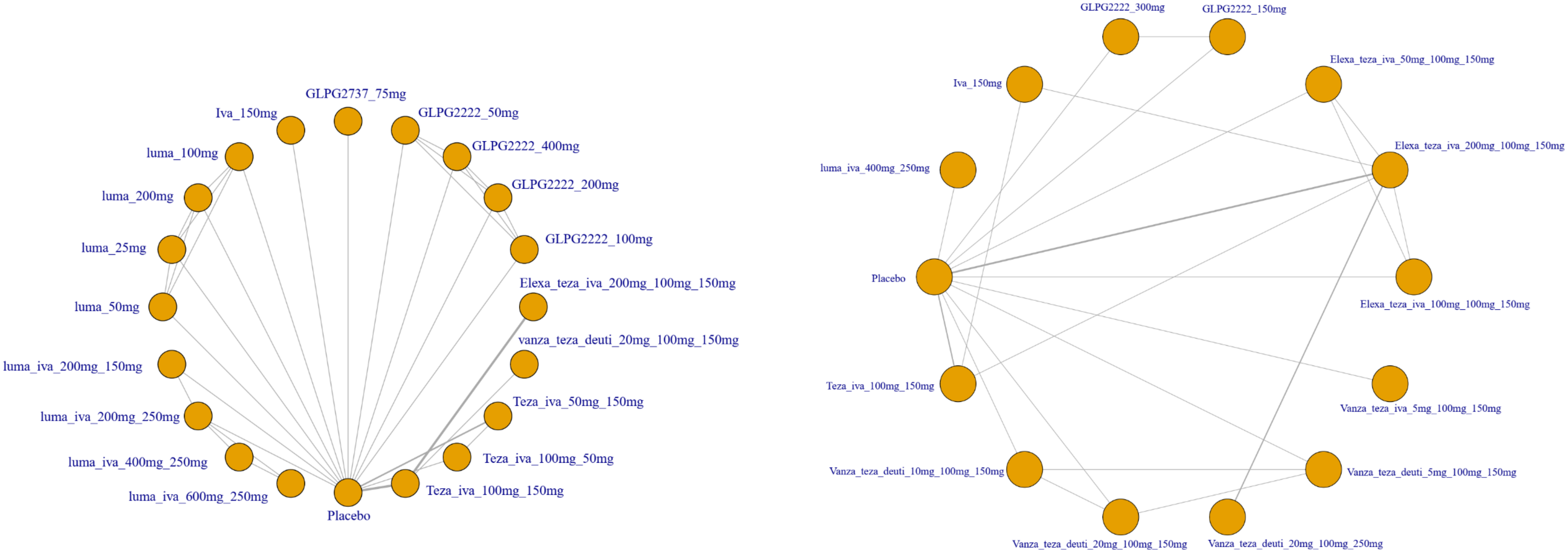

A **B**

eFigure 6 Network diagrams for sweat chloride in adults : (A) Homozygous to phe508del mutation ; (B) Heterozygous to phe508del mutation

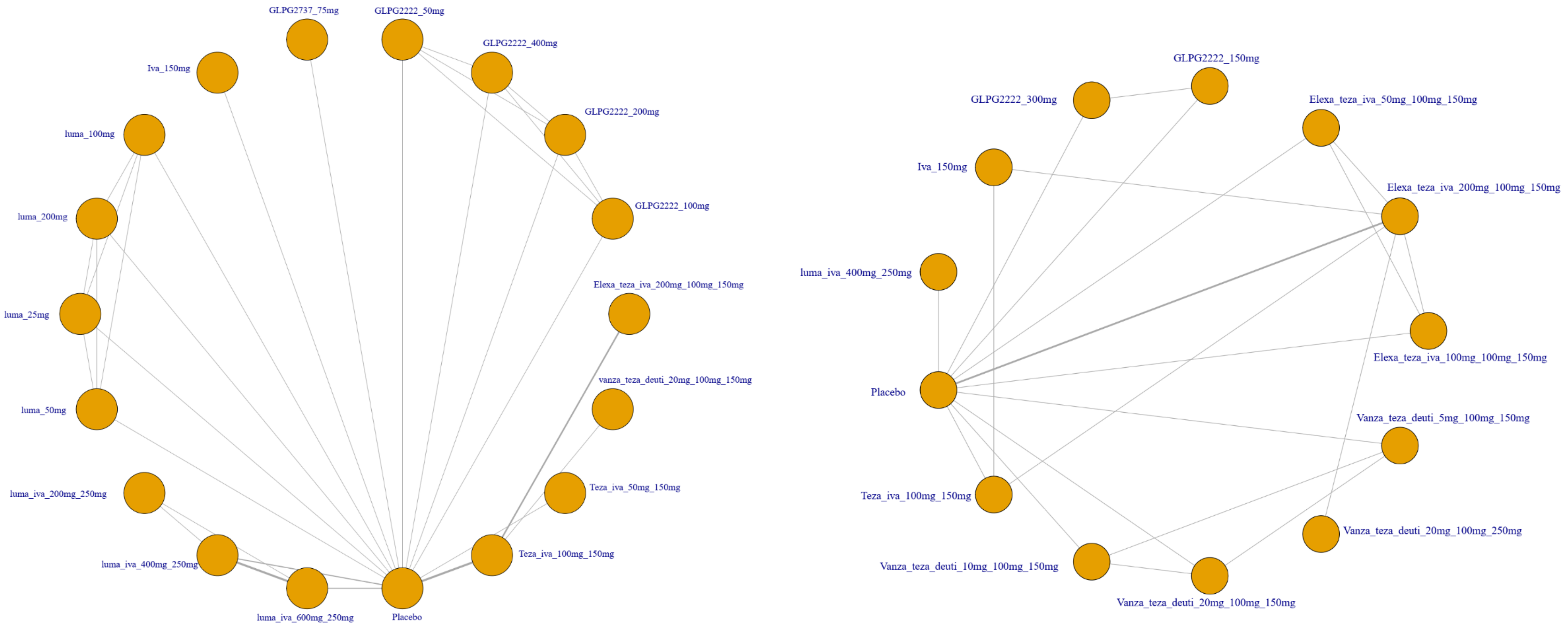

A **B**

eFigure 7 Network diagrams for CFQ-R in adults : (A) Homozygous to phe508del mutation ; (B) Heterozygous to phe508del mutation

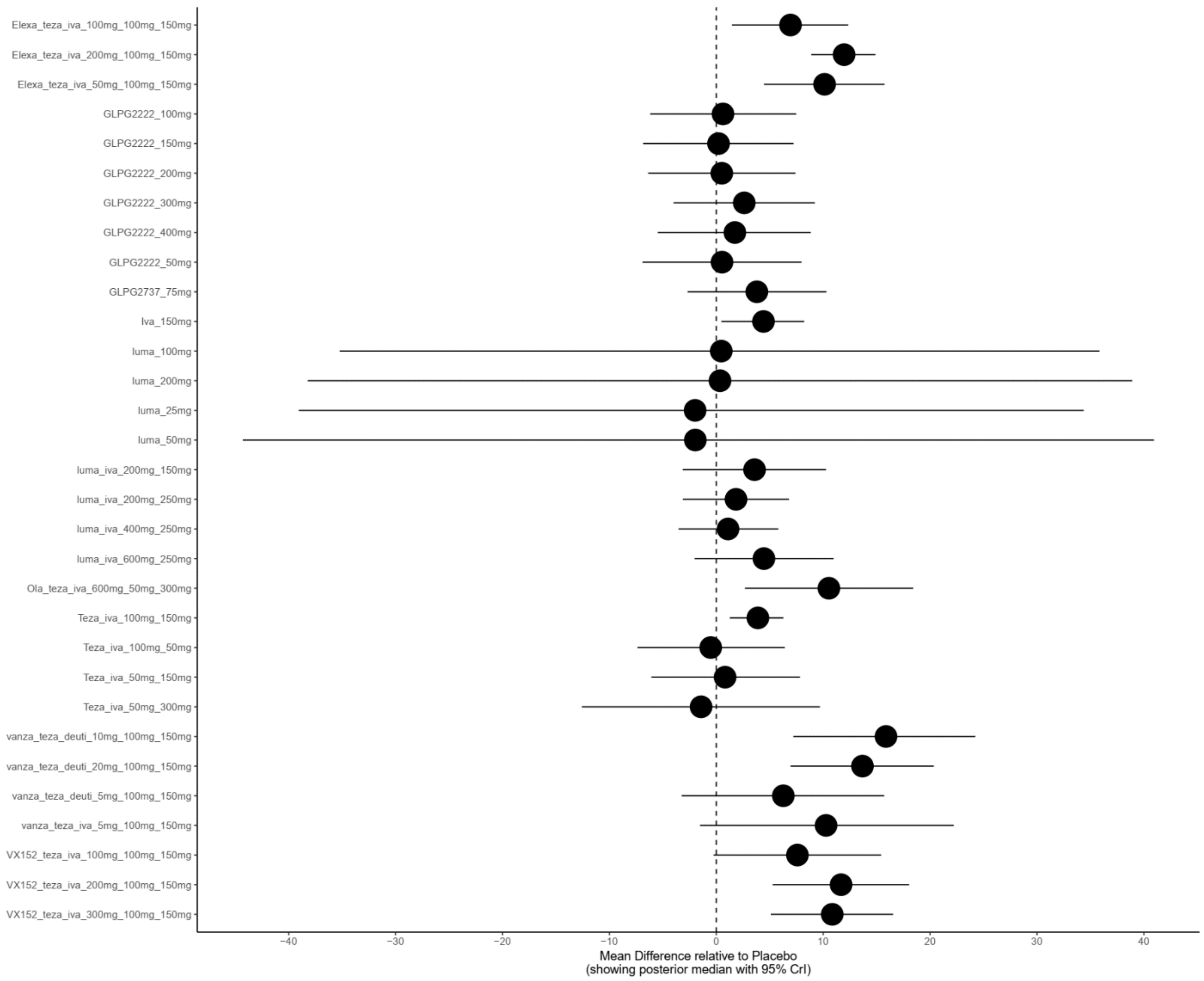

eFigure 8 Forest plot of ppFEV$_1$ in adults treated for 4 to 8 weeks

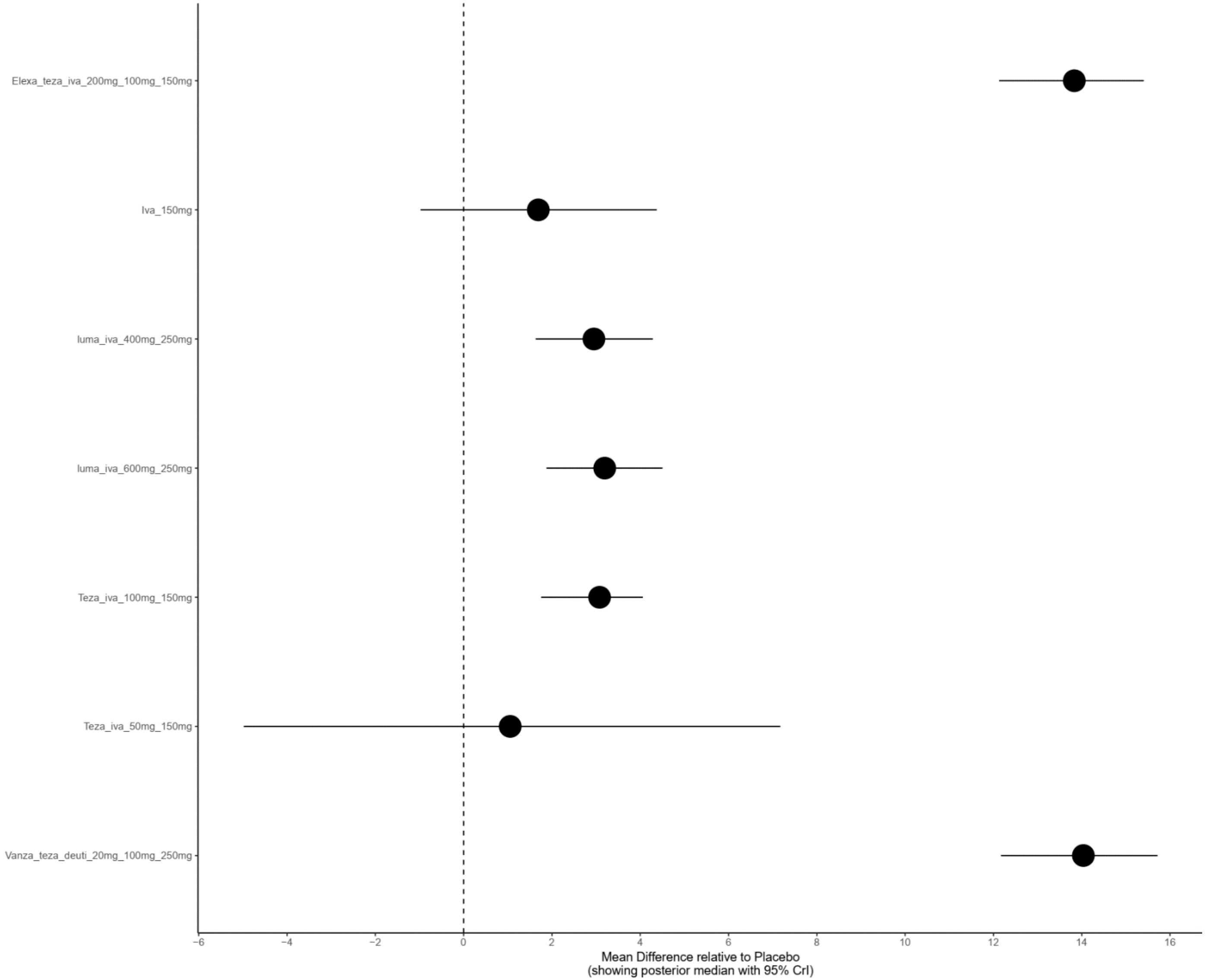

eFigure 9 Forest plot of $ppFEV_1$ in adults treated for greater than 8 weeks

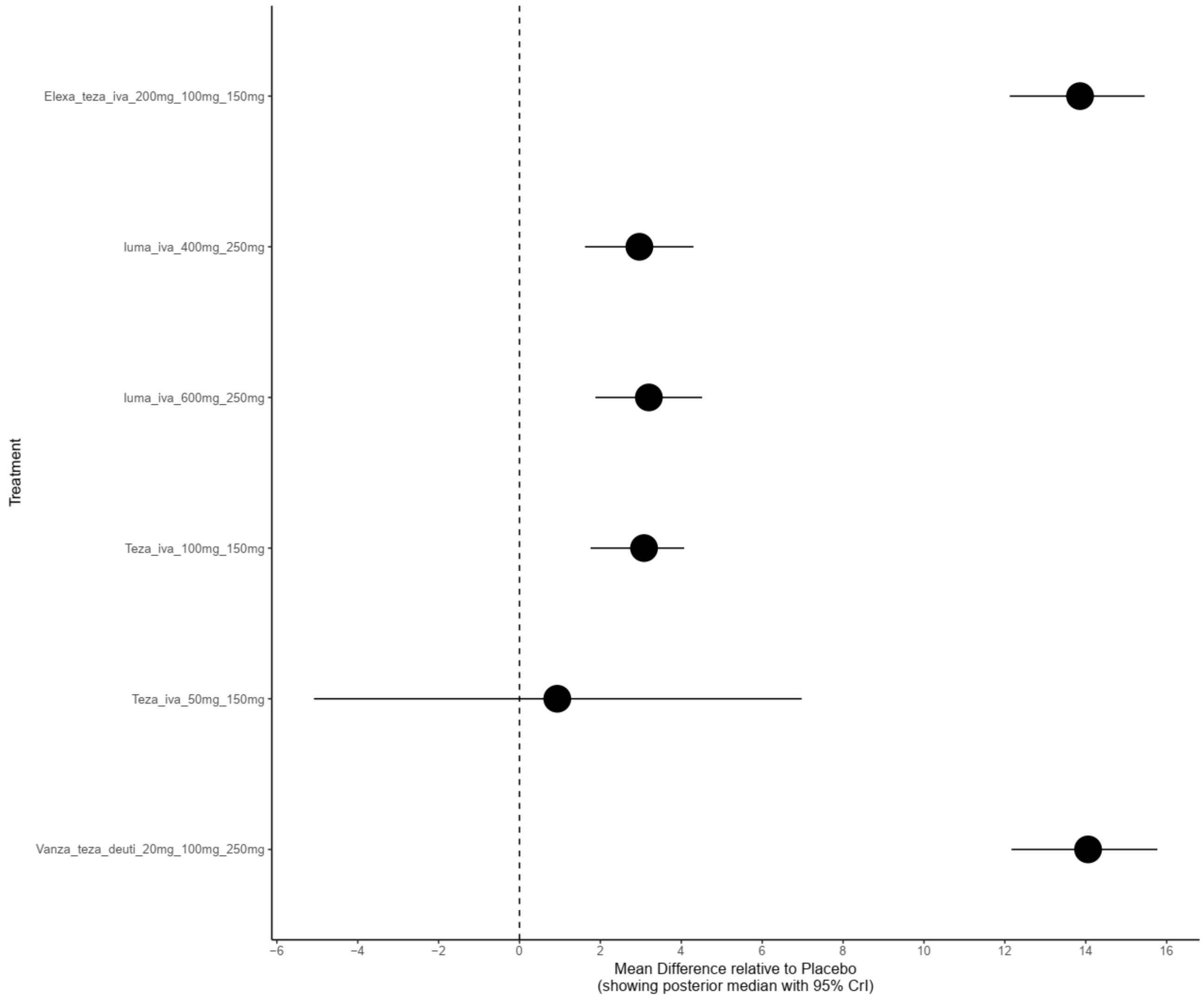

eFigure 10 Forest plot of ppFEV$_1$ in adults treated for greater than 8 weeks (excluding Flume et al.)

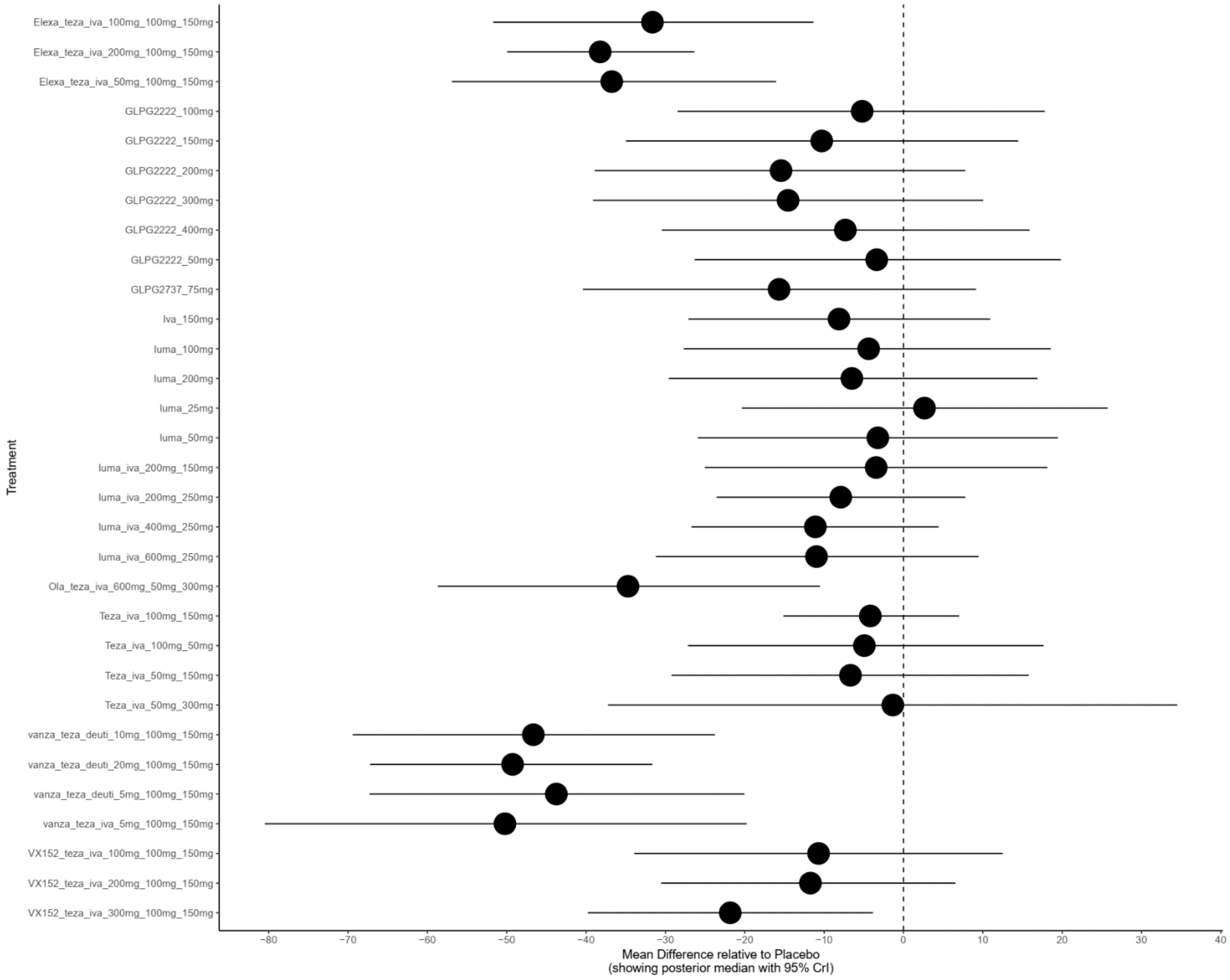

eFigure 11 Forest plot of sweat chloride in adults treated for 4 to 8 weeks

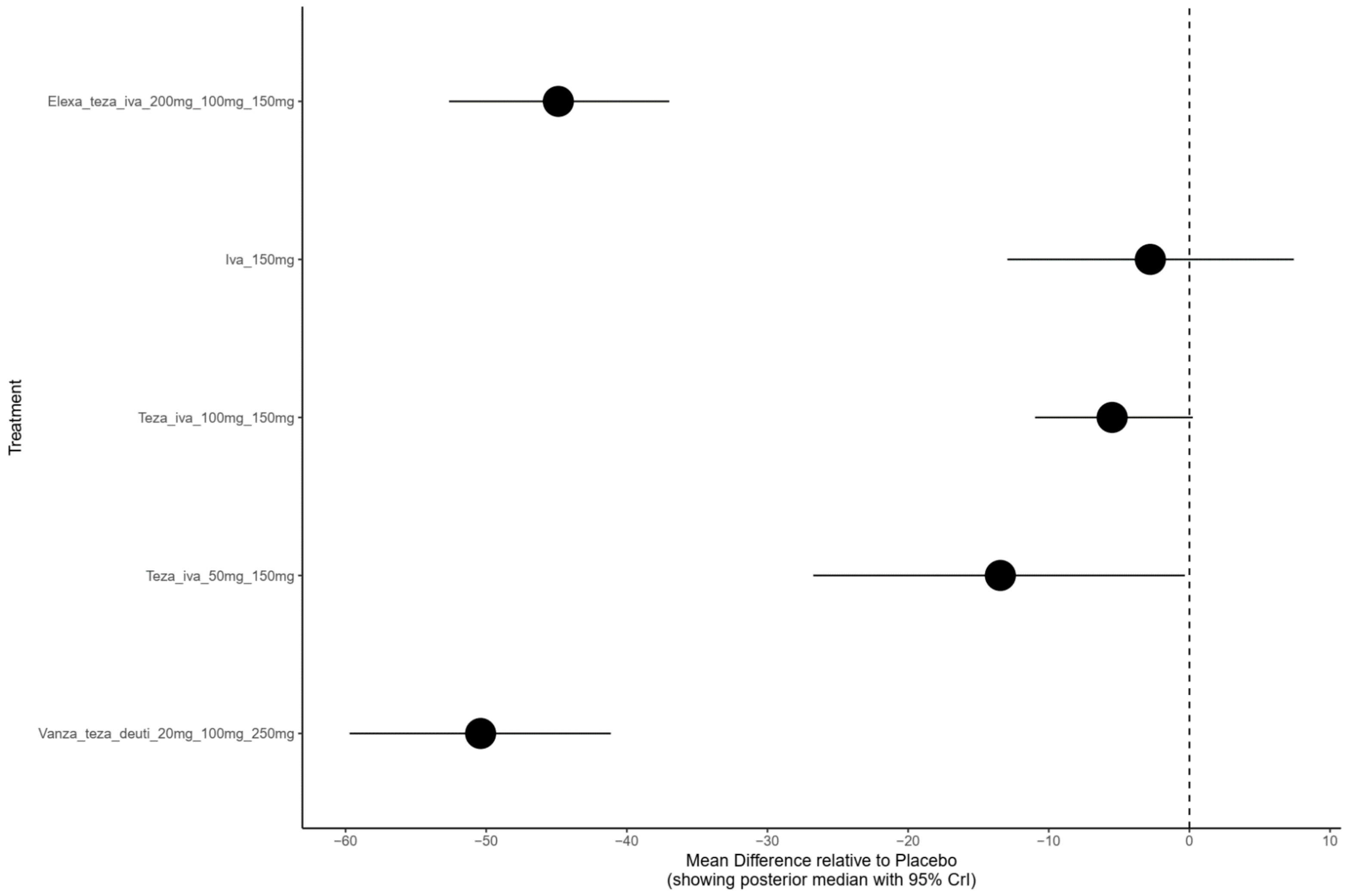

eFigure 12 Forest plot of sweat chloride in adults treated for greater than 8 weeks

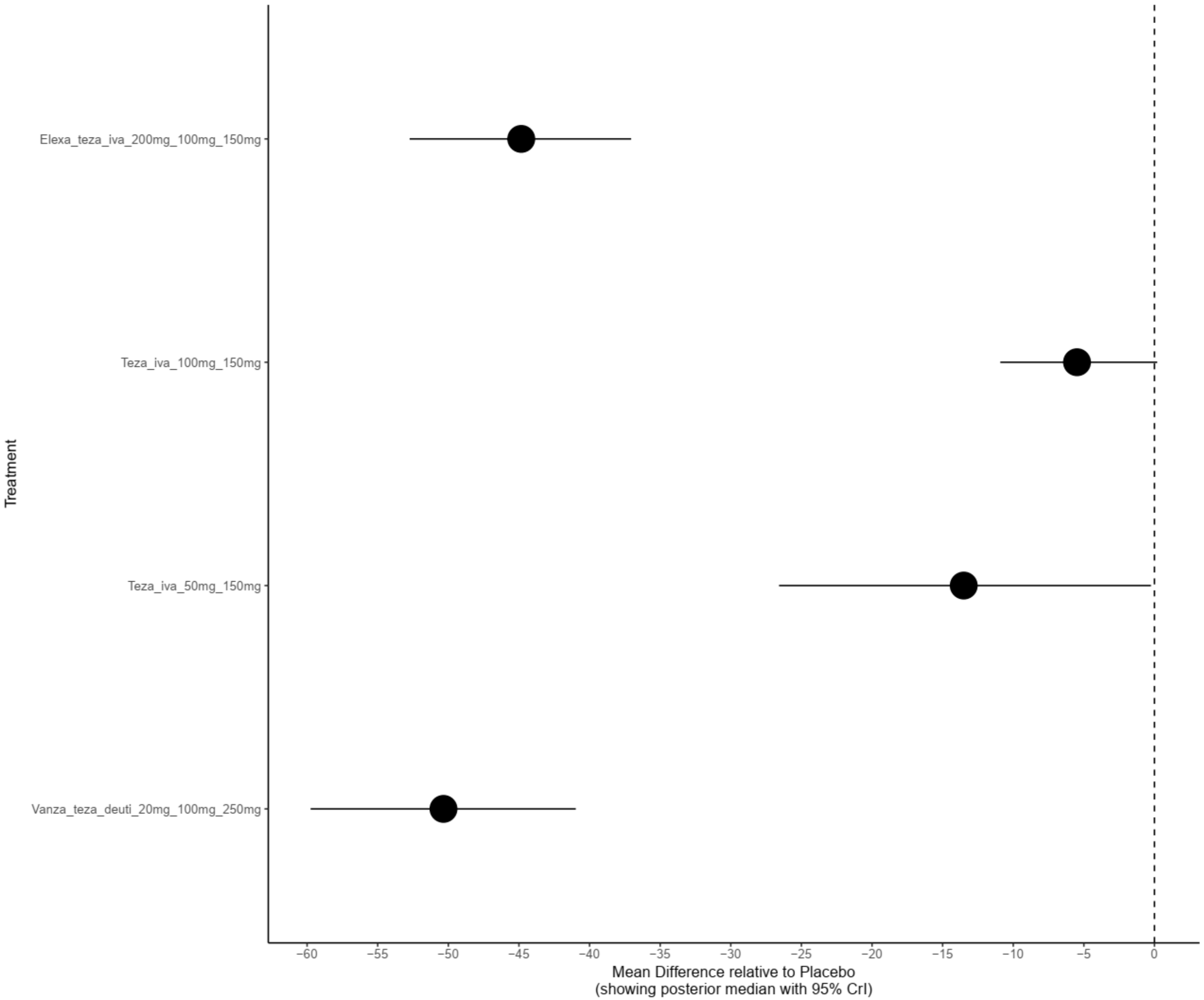

eFigure 13 Forest plot of sweat chloride in adults treated for greater than 8 weeks (excluding Flume et al.)

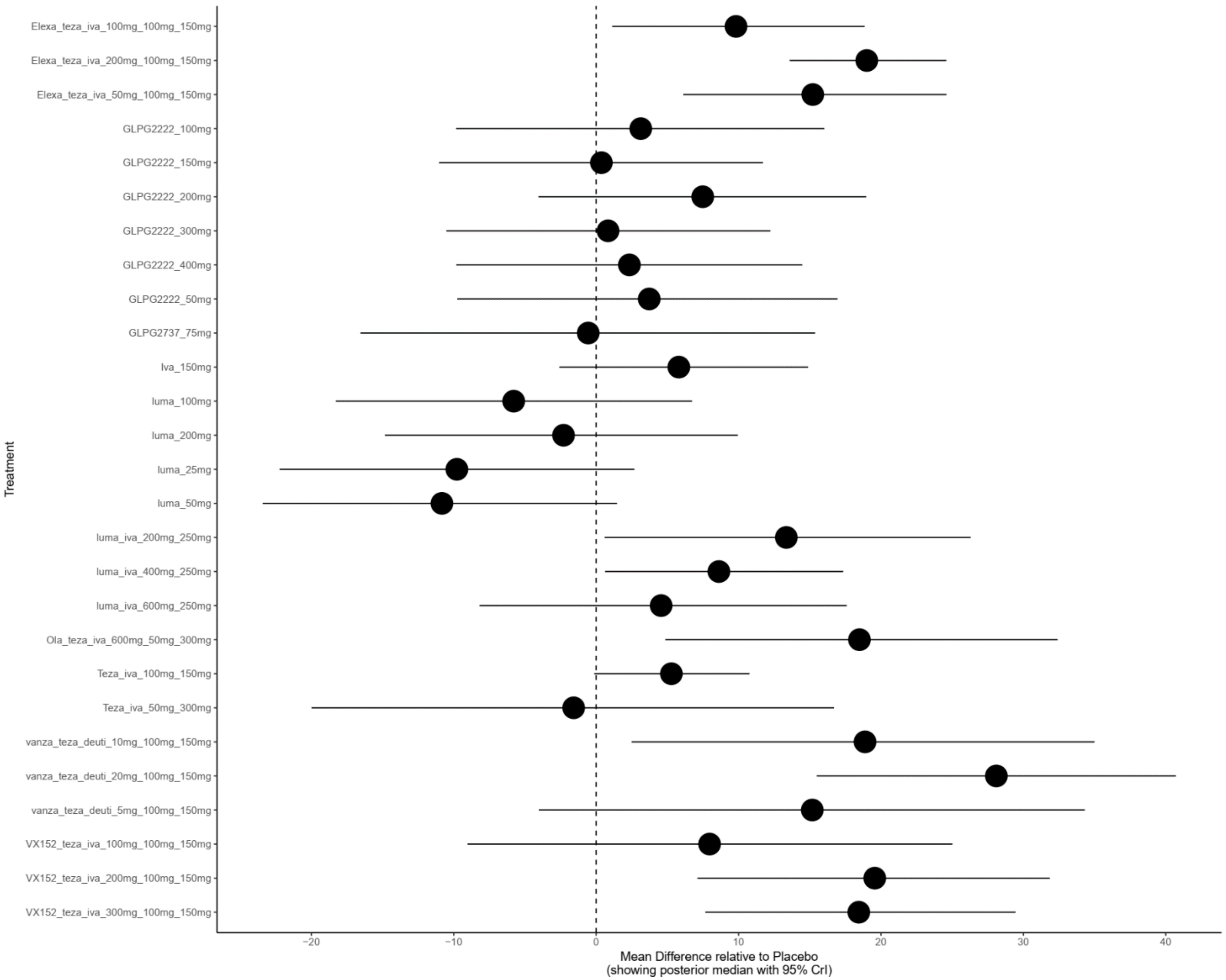

eFigure 14 Forest plot of CFQ-R in adults treated for 4 to 8 weeks

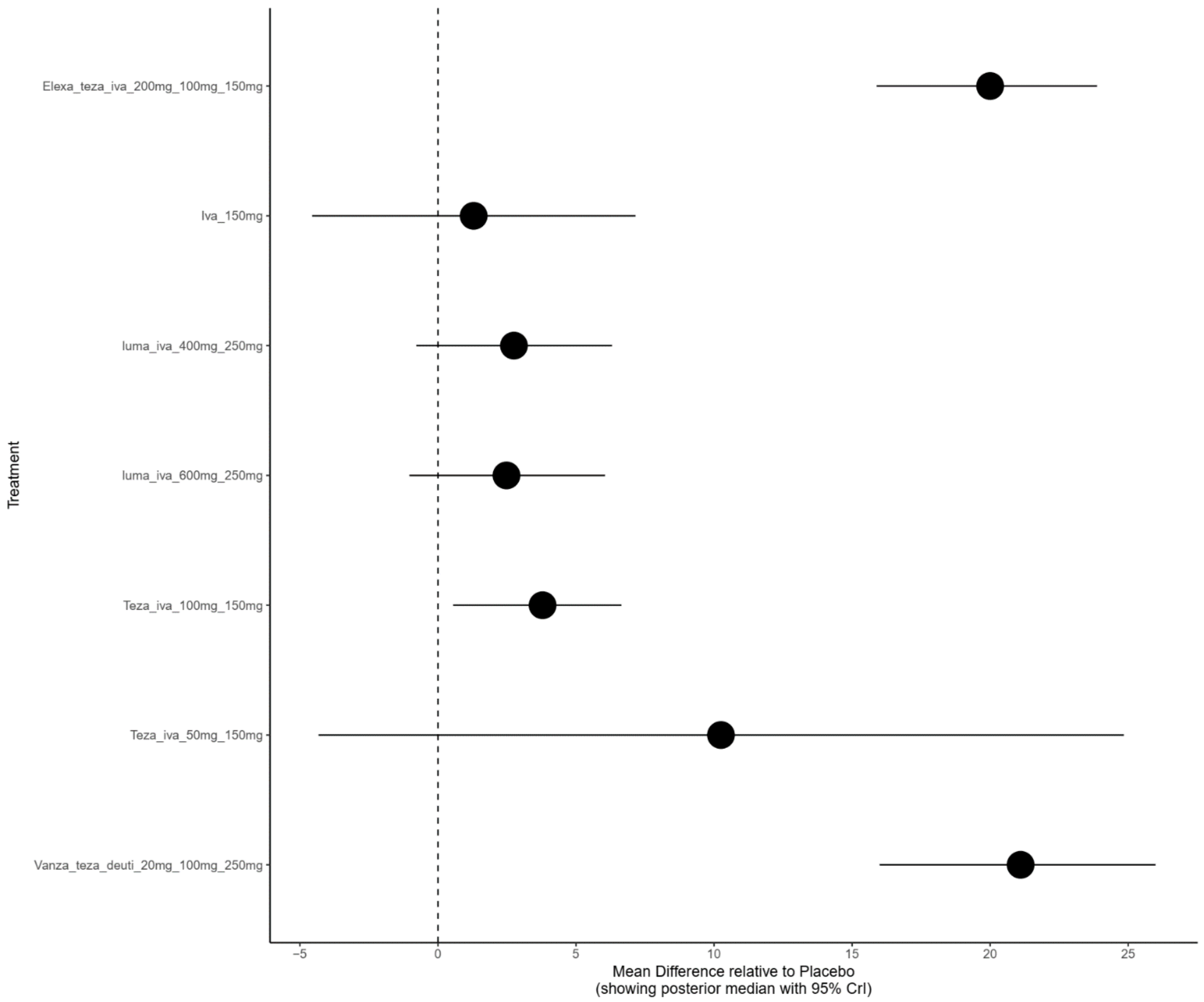

eFigure 15 Forest plot of CFQ-R in adults treated for greater than 8 weeks

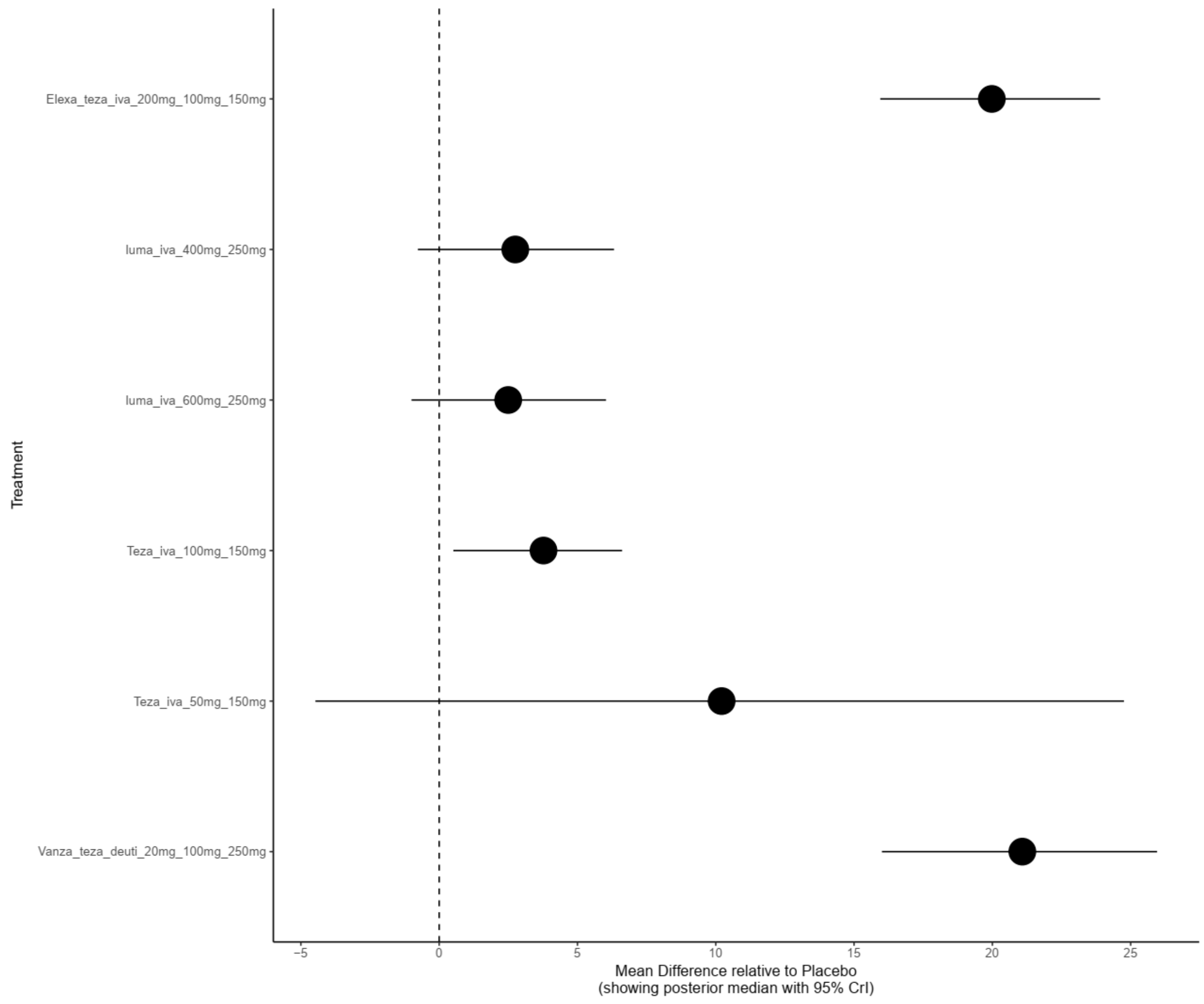

eFigure 16 Forest plot of CFQ-R in adults treated for greater than 8 weeks (excluding flume et al.)

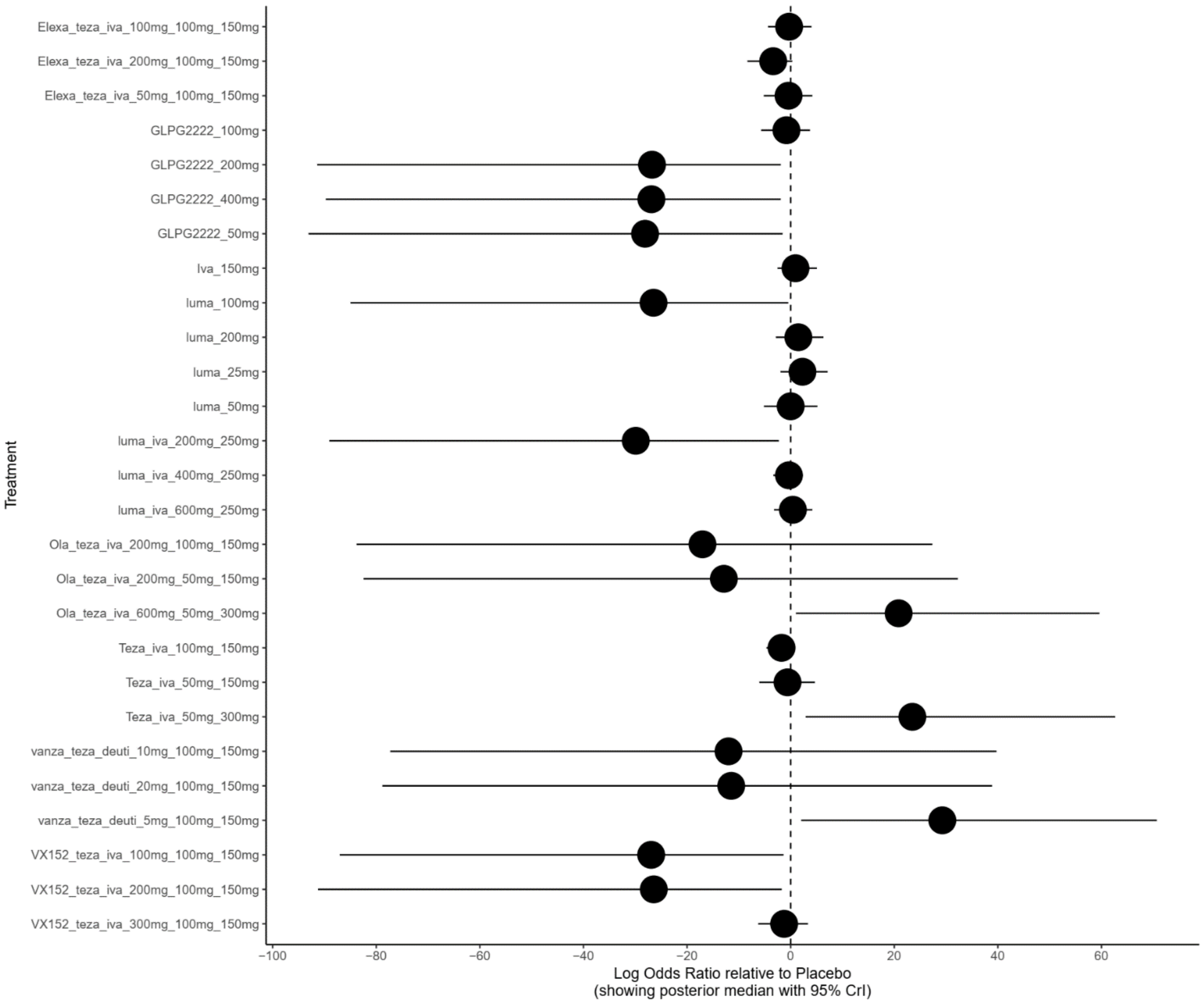

eFigure 17 Forest plot of serious adverse event in adults treated for 4 to 8 weeks

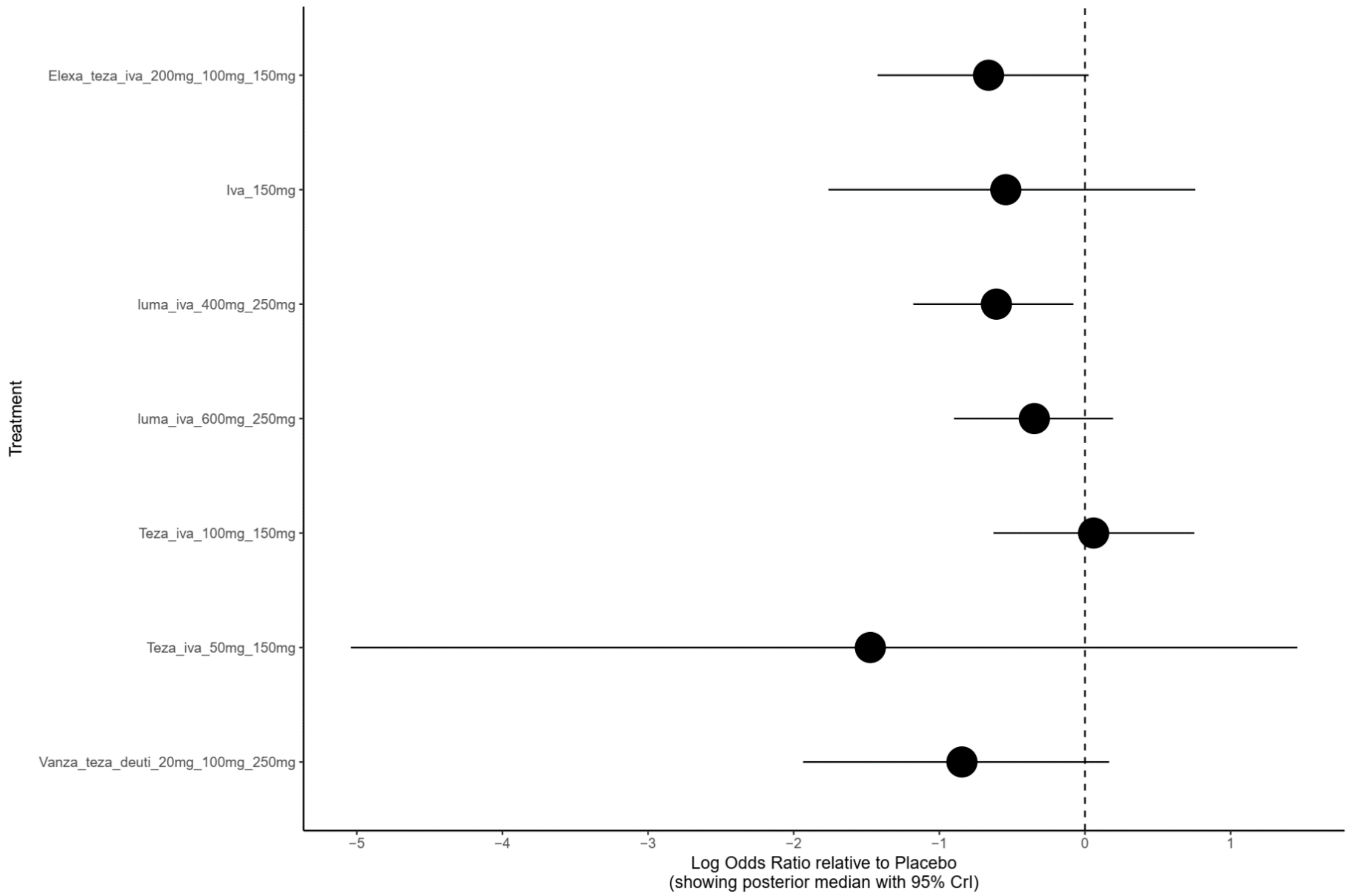

eFigure 18 Forest plot of serious adverse event in adults treated for greater than 8 weeks

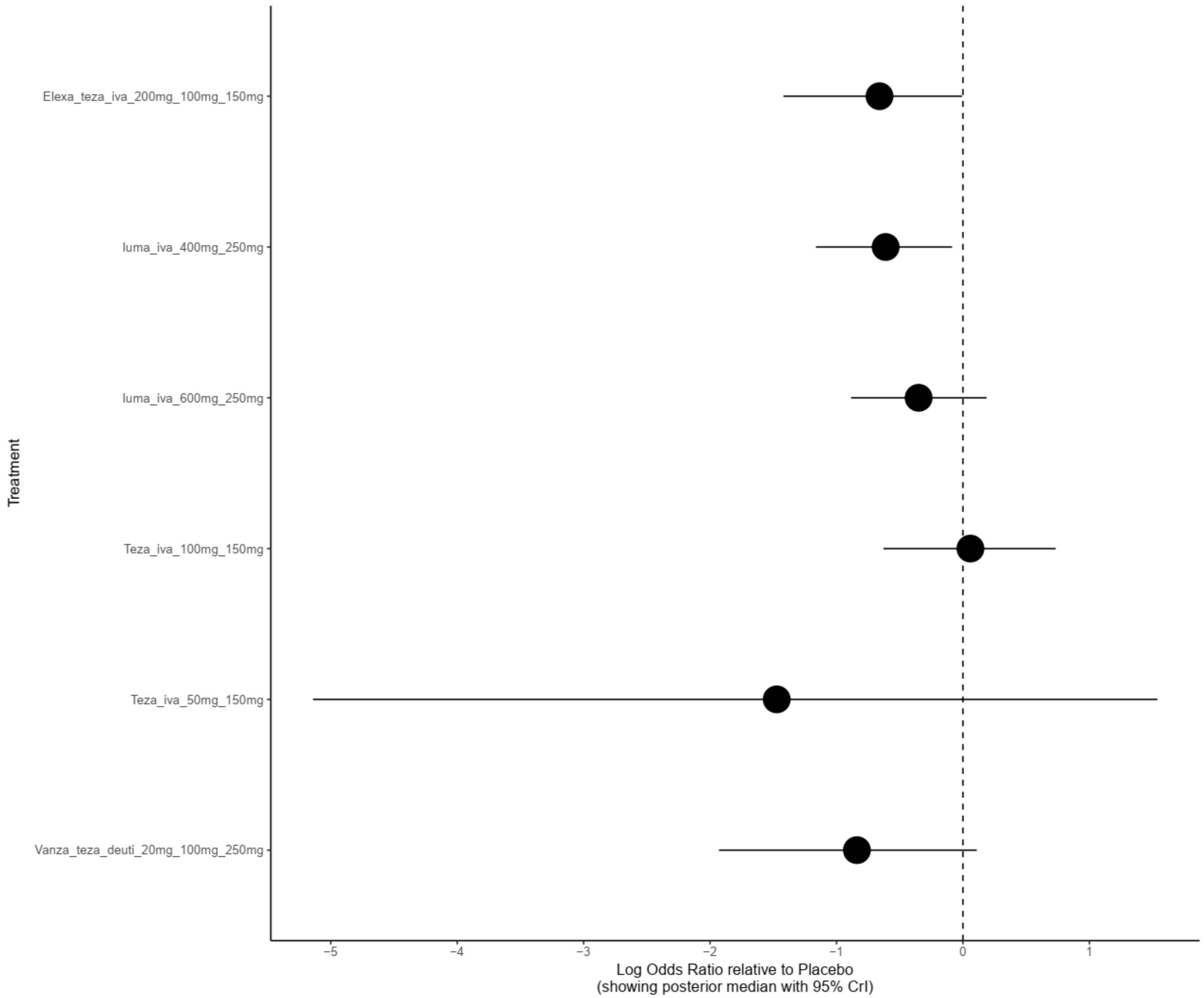

eFigure 19 Forest plot of serious adverse event in adults treated for greater than 8 weeks (excluding flume et al.)

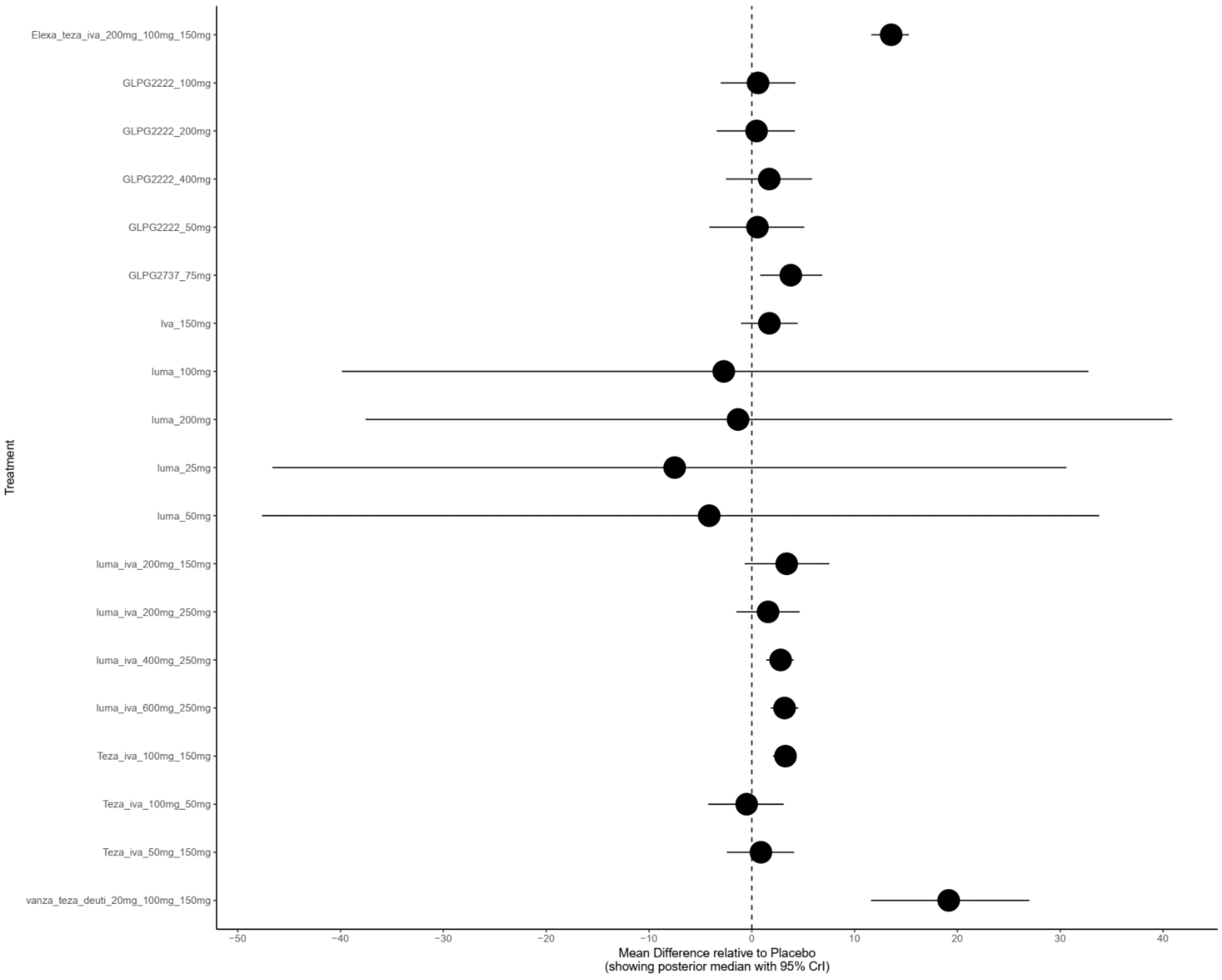

eFigure 20 Forest plot of $ppFEV_1$ in adults homozygous to phe508del mutation

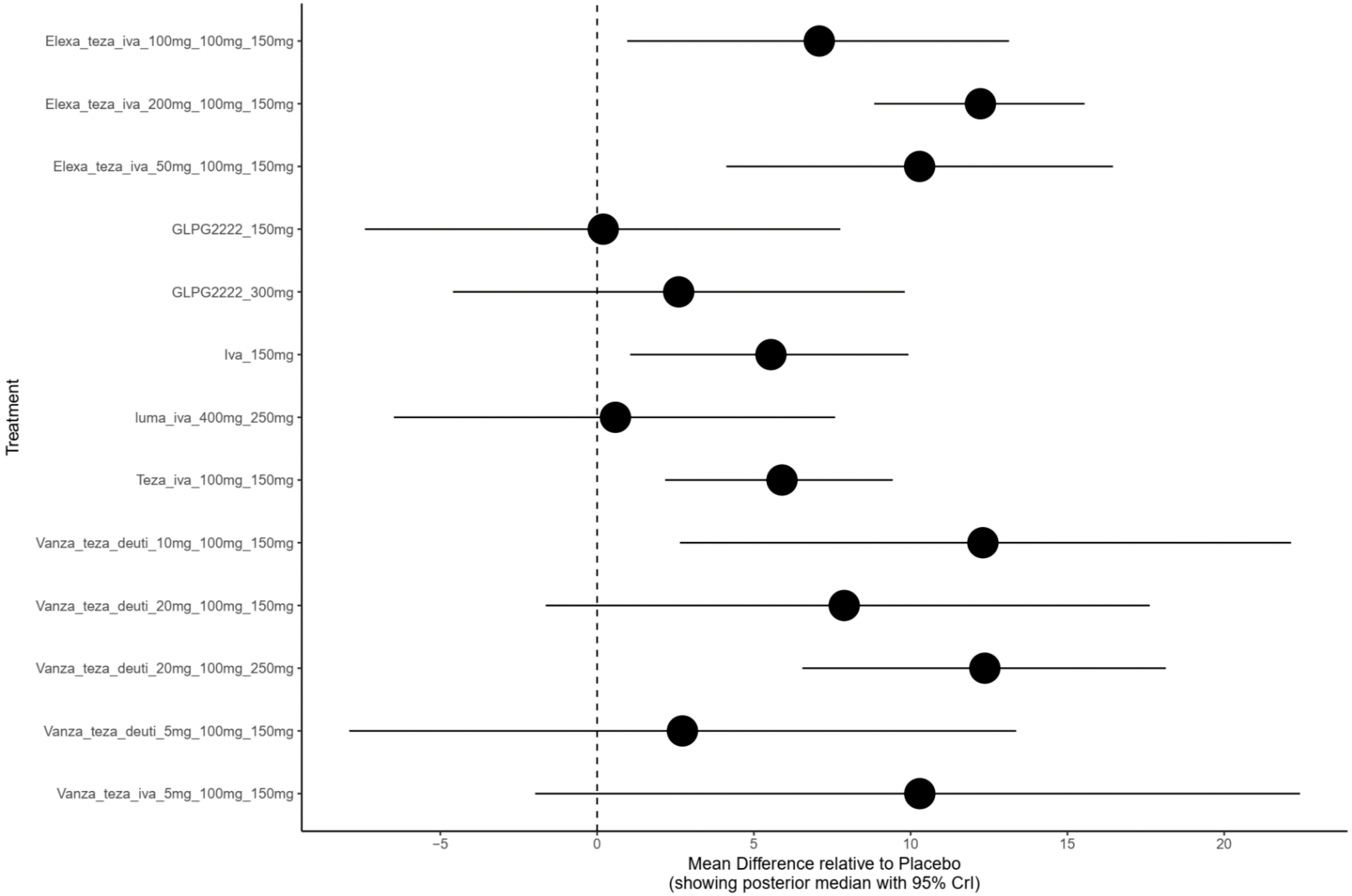

eFigure 21 Forest plot of ppFEV$_1$ in adults heterozygous to phe508del mutation

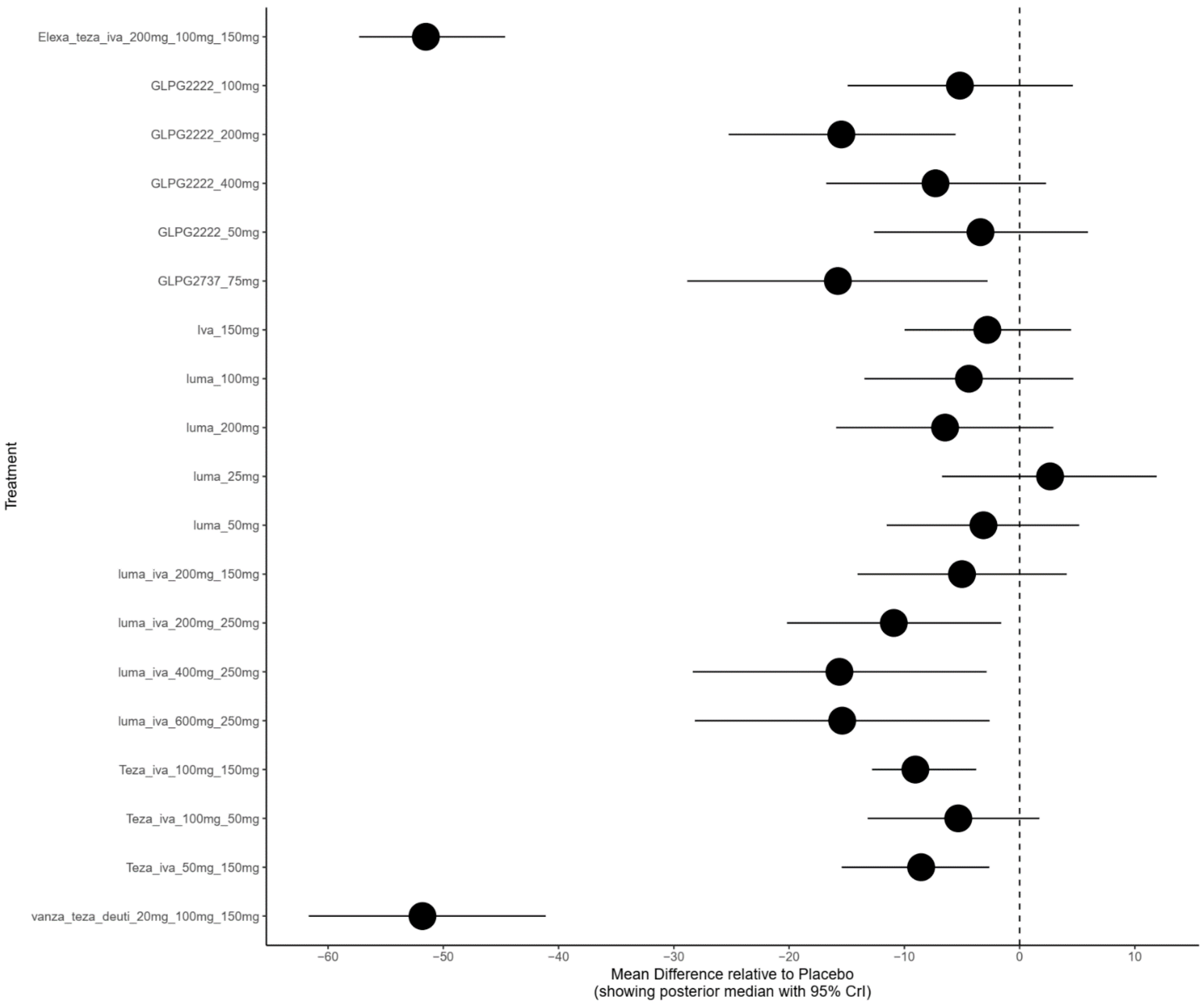

eFigure 22 Forest plot of sweat chloride in adults homozygous to phe508del mutation

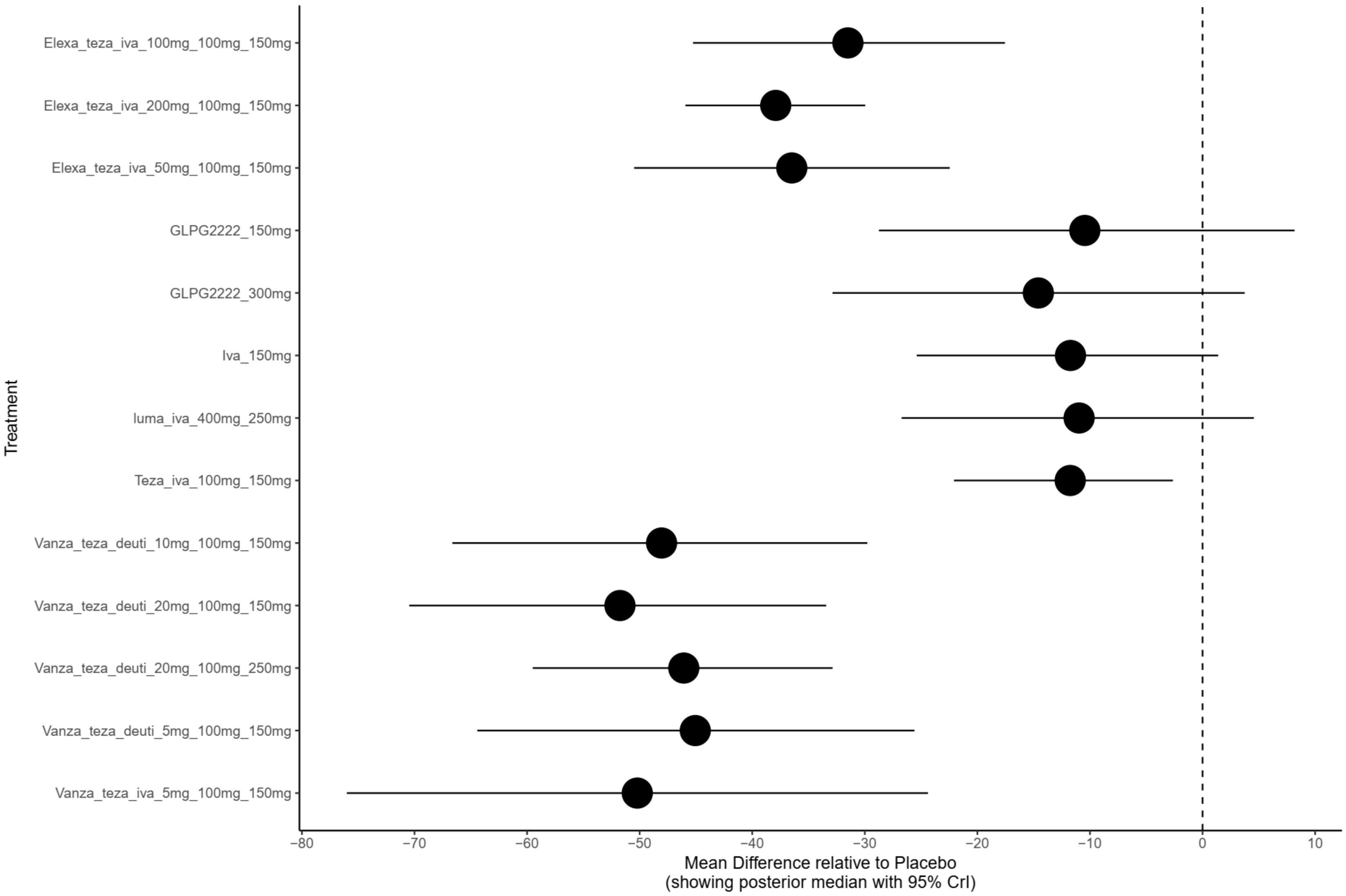

eFigure 23 Forest plot of sweat chloride in adults heterozygous to phe508del mutation

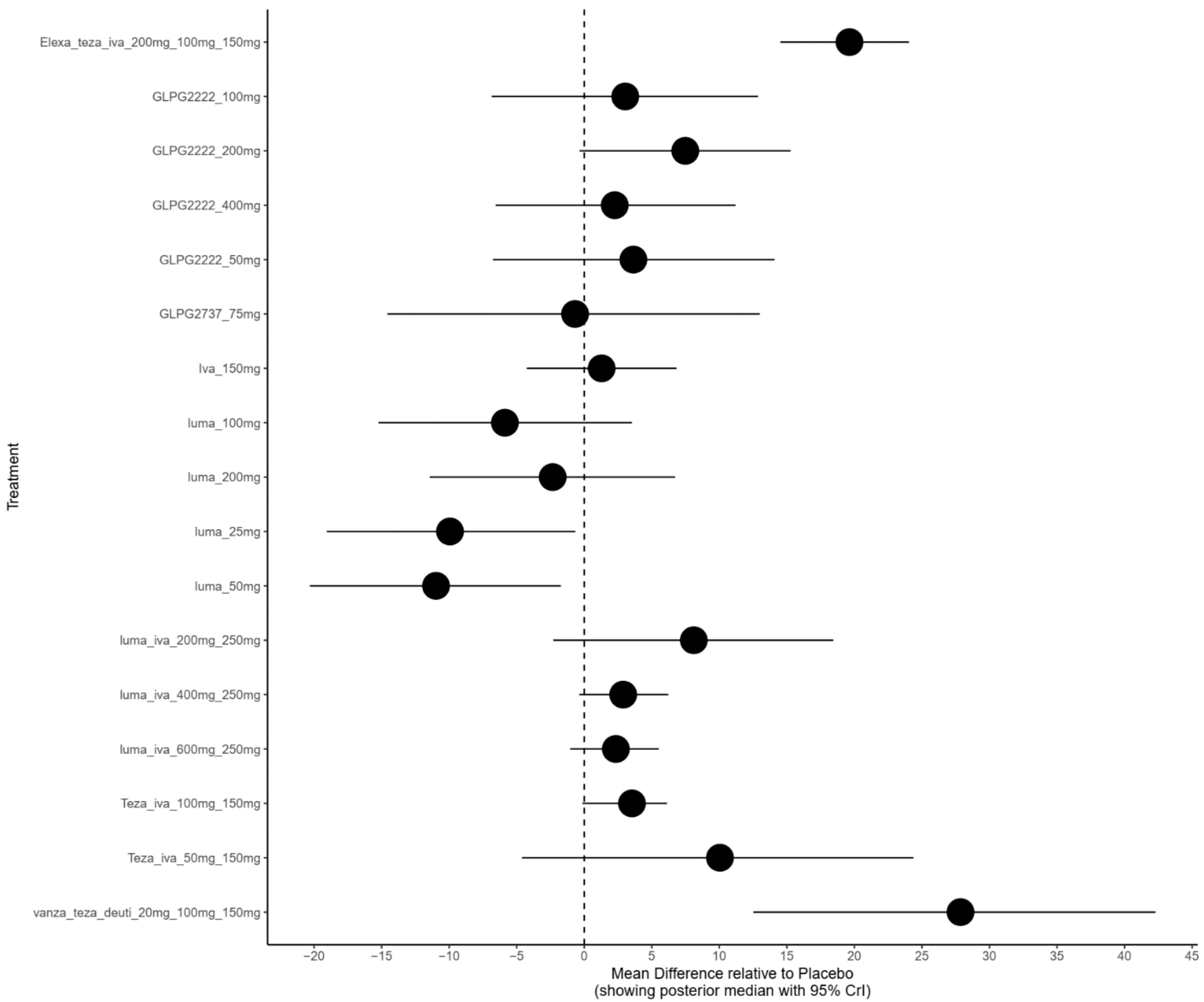

eFigure 24 Forest plot of CFQ-R in adults homozygous to phe508del mutation

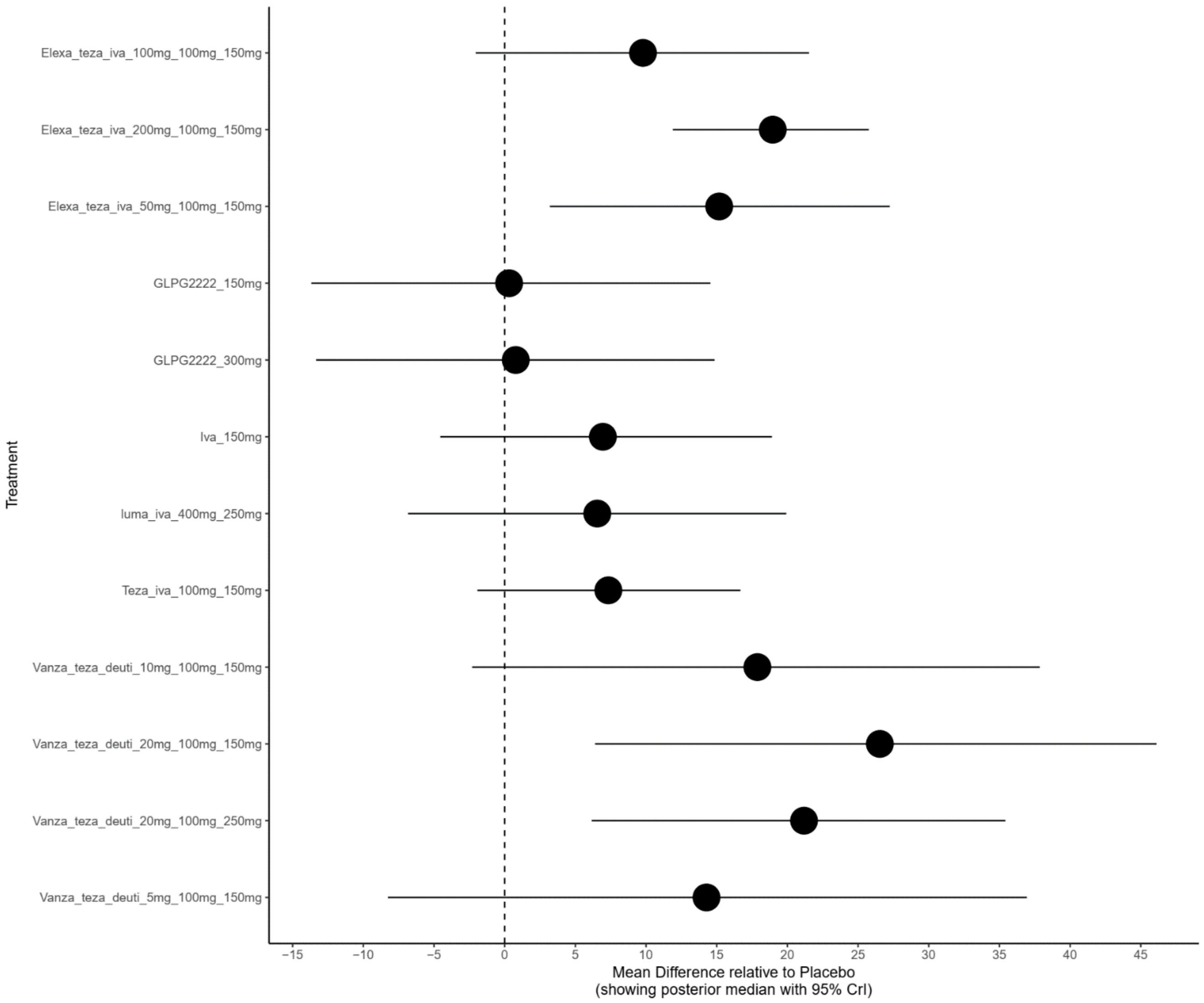

eFigure 25 Forest plot of CFQ-R in adults heterozygous to phe508del mutation

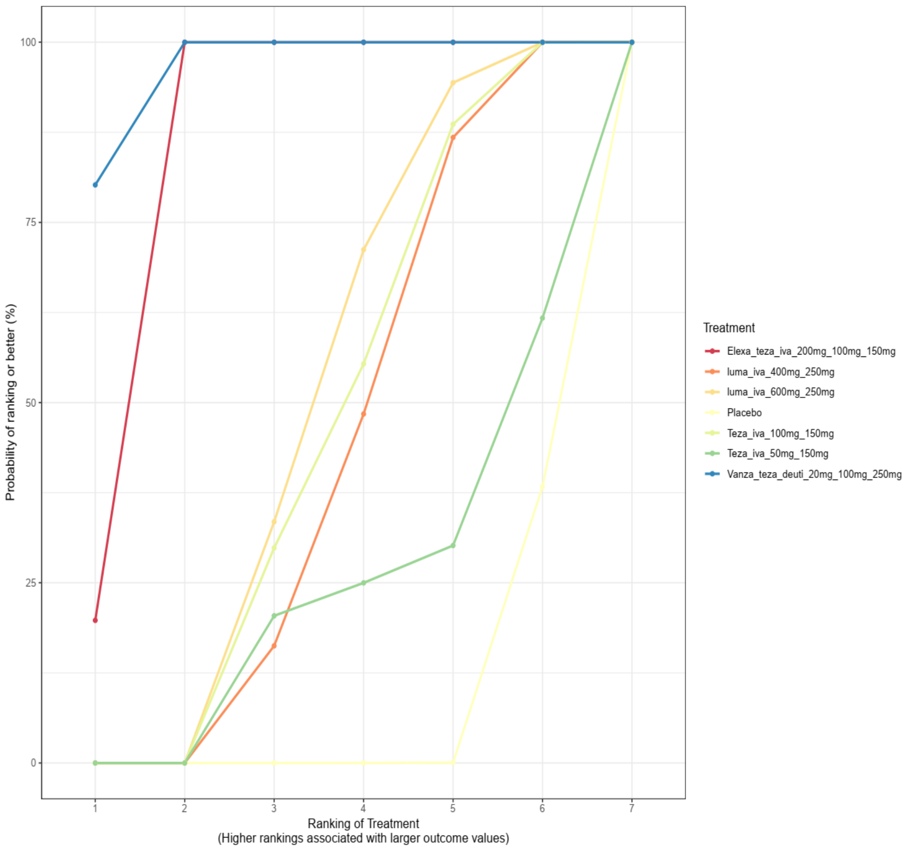

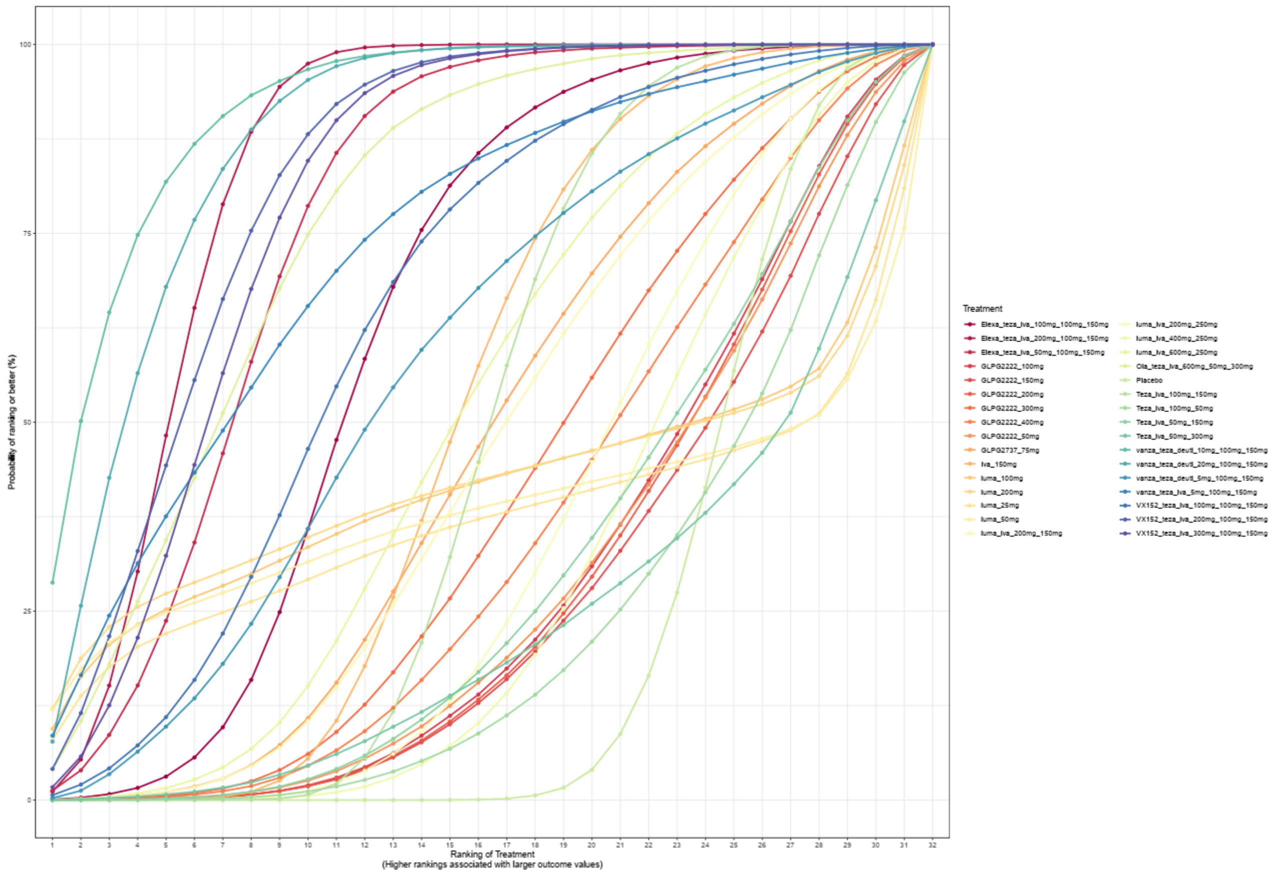

A **B**

eFigure 26 SUCRA plot of ppFEV1 in A) Adults treated for greater than 8 weeks (excluding flume et al.) B) Adults treated for 4 to 8 weeks

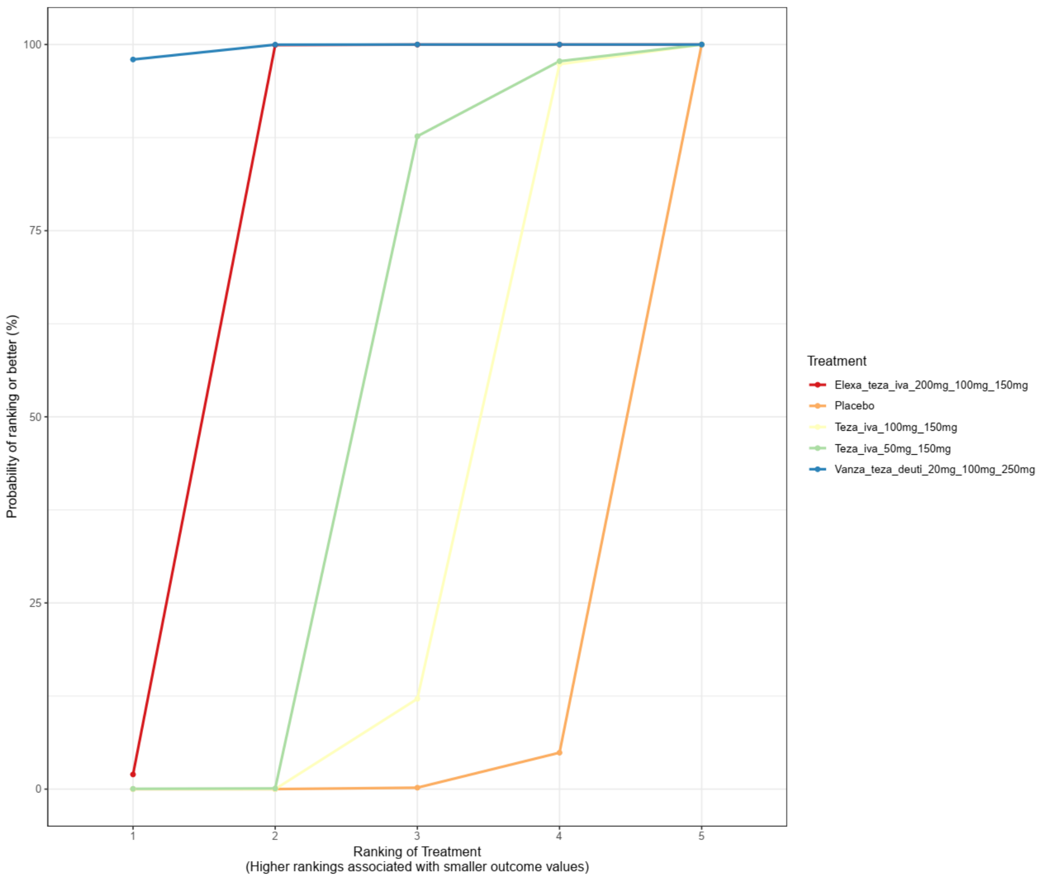
Probability of ranking or better (%)
Ranking of Treatment
(Higher rankings associated with smaller outcome values)
Treatment
Elexa_teza_iva_200mg_100mg_150mg
Placebo
Teza_iva_100mg_150mg
Teza_iva_50mg_150mg
Vanza_teza_deuti_20mg_100mg_250mg

A

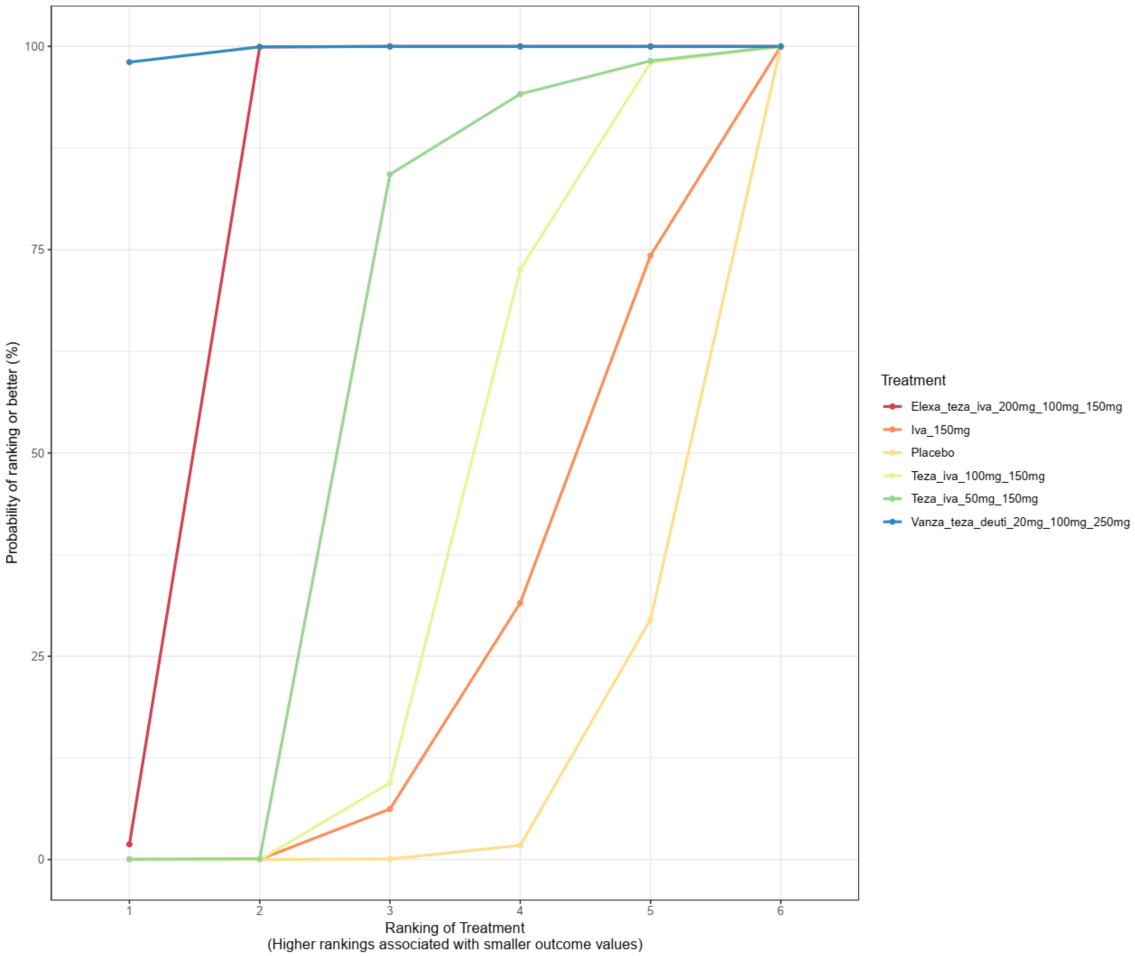
Probability of ranking or better (%)
Ranking of Treatment
(Higher rankings associated with smaller outcome values)
Treatment
Elexa_teza_iva_200mg_100mg_150mg
Iva_150mg
Placebo
Teza_iva_100mg_150mg
Teza_iva_50mg_150mg
Vanza_teza_deuti_20mg_100mg_250mg

B

C)

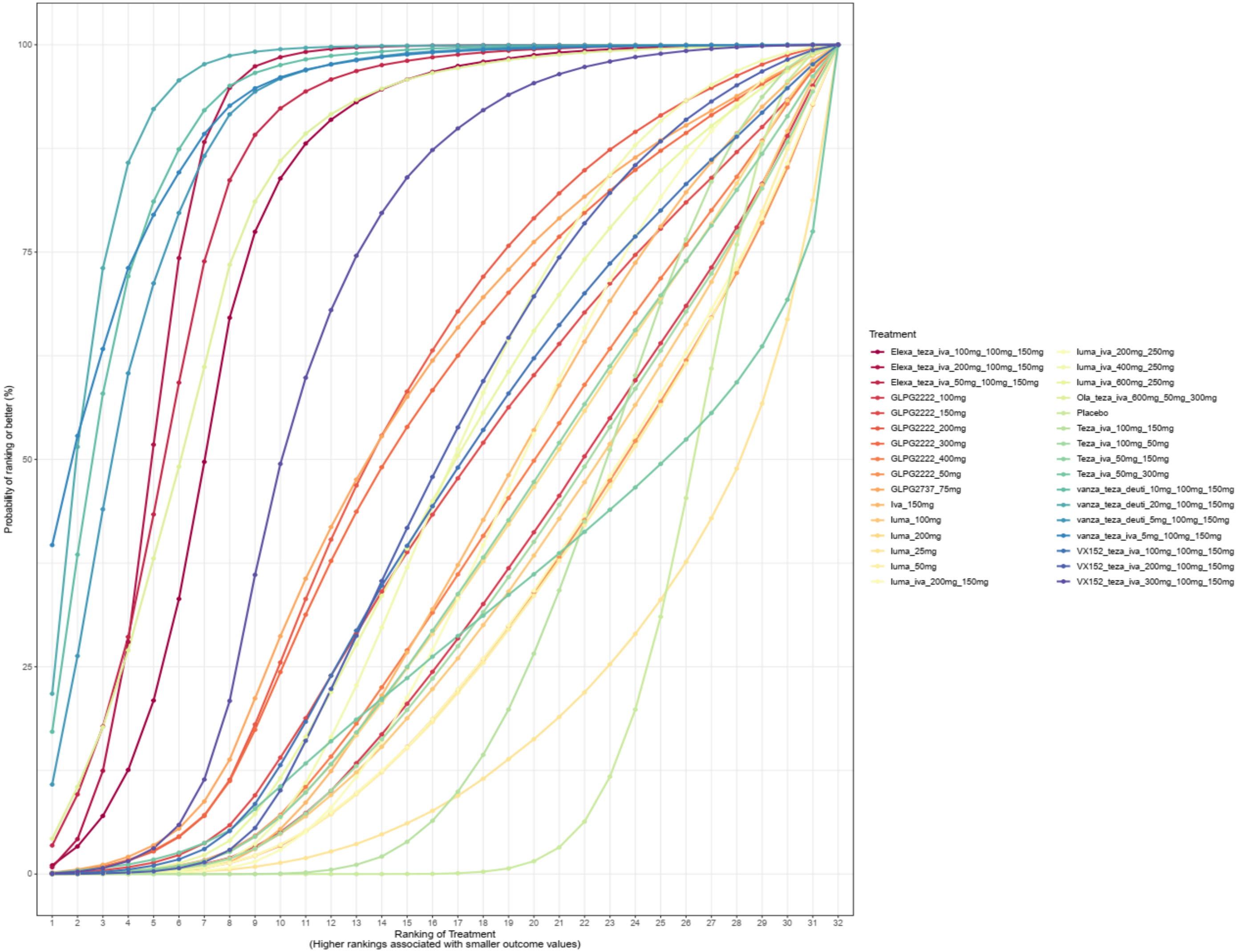

eFigure 27 SUCRA plot of sweat chloride in A) Adults treated for greater than 8 weeks (excluding flume et al.) B) Adults treated for greater than 8 weeks C) Adults treated for 4 to 8 weeks

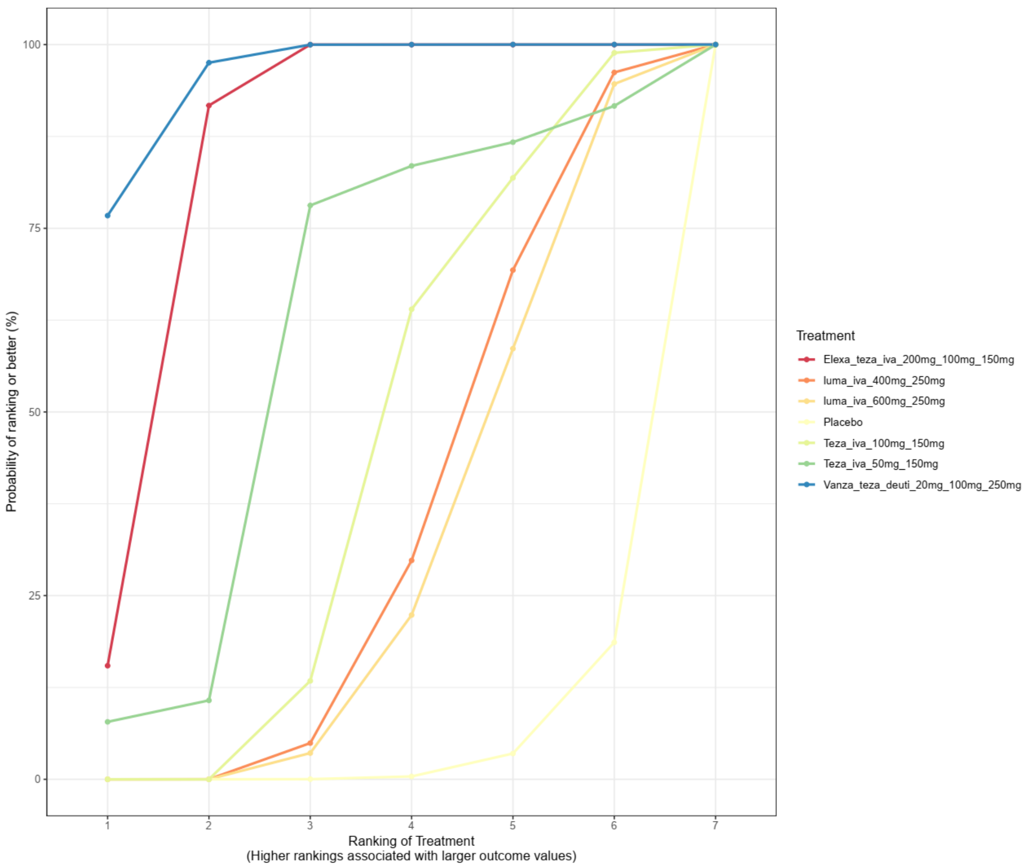
Probability of ranking or better (%)
100
75
50
25
0
1
2
3
4
5
6
7
Ranking of Treatment
(Higher rankings associated with larger outcome values)
Treatment
Elexa_teza_iva_200mg_100mg_150mg
luma_iva_400mg_250mg
luma_iva_600mg_250mg
Placebo
Teza_iva_100mg_150mg
Teza_iva_50mg_150mg
Vanza_teza_deuti_20mg_100mg_250mg

A)

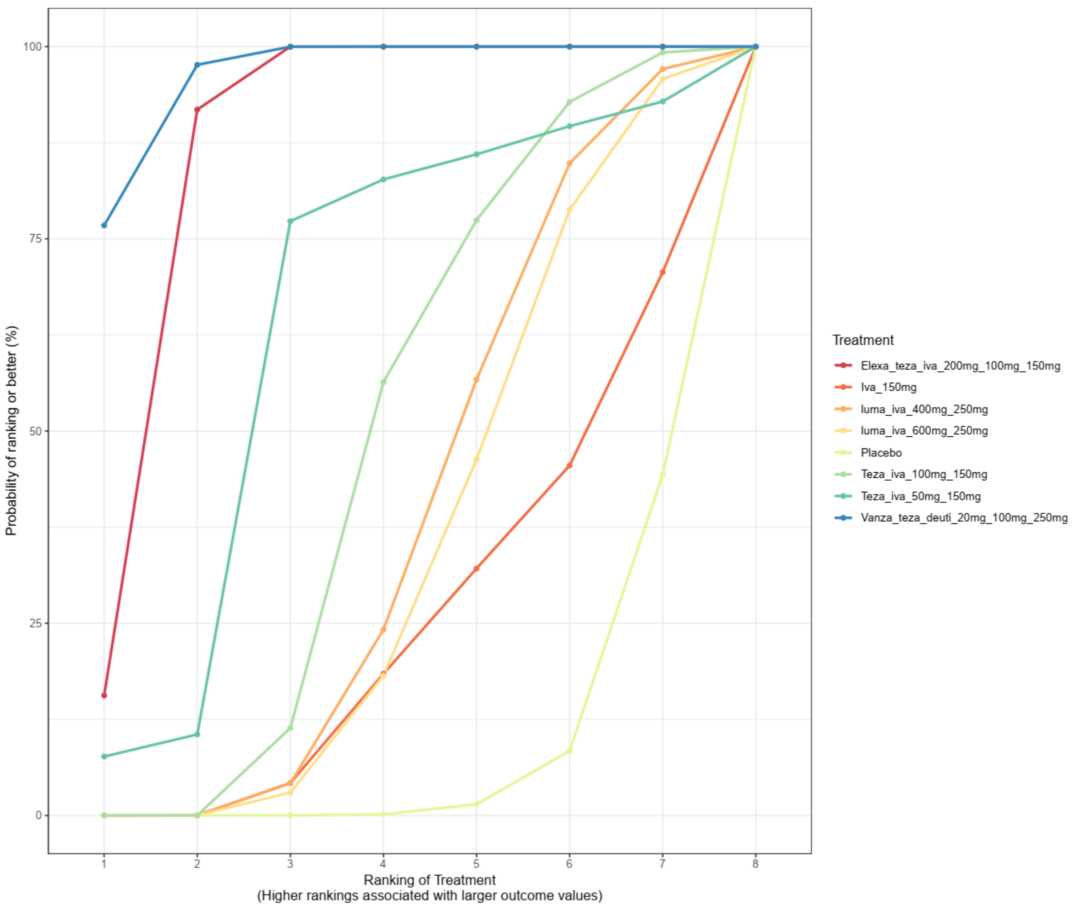
Probability of ranking or better (%)
100
75
50
25
0
1
2
3
4
5
6
7
8
Ranking of Treatment
(Higher rankings associated with larger outcome values)
Treatment
Elexa_teza_iva_200mg_100mg_150mg
Iva_150mg
luma_iva_400mg_250mg
luma_iva_600mg_250mg
Placebo
Teza_iva_100mg_150mg
Teza_iva_50mg_150mg
Vanza_teza_deuti_20mg_100mg_250mg

B)

C)

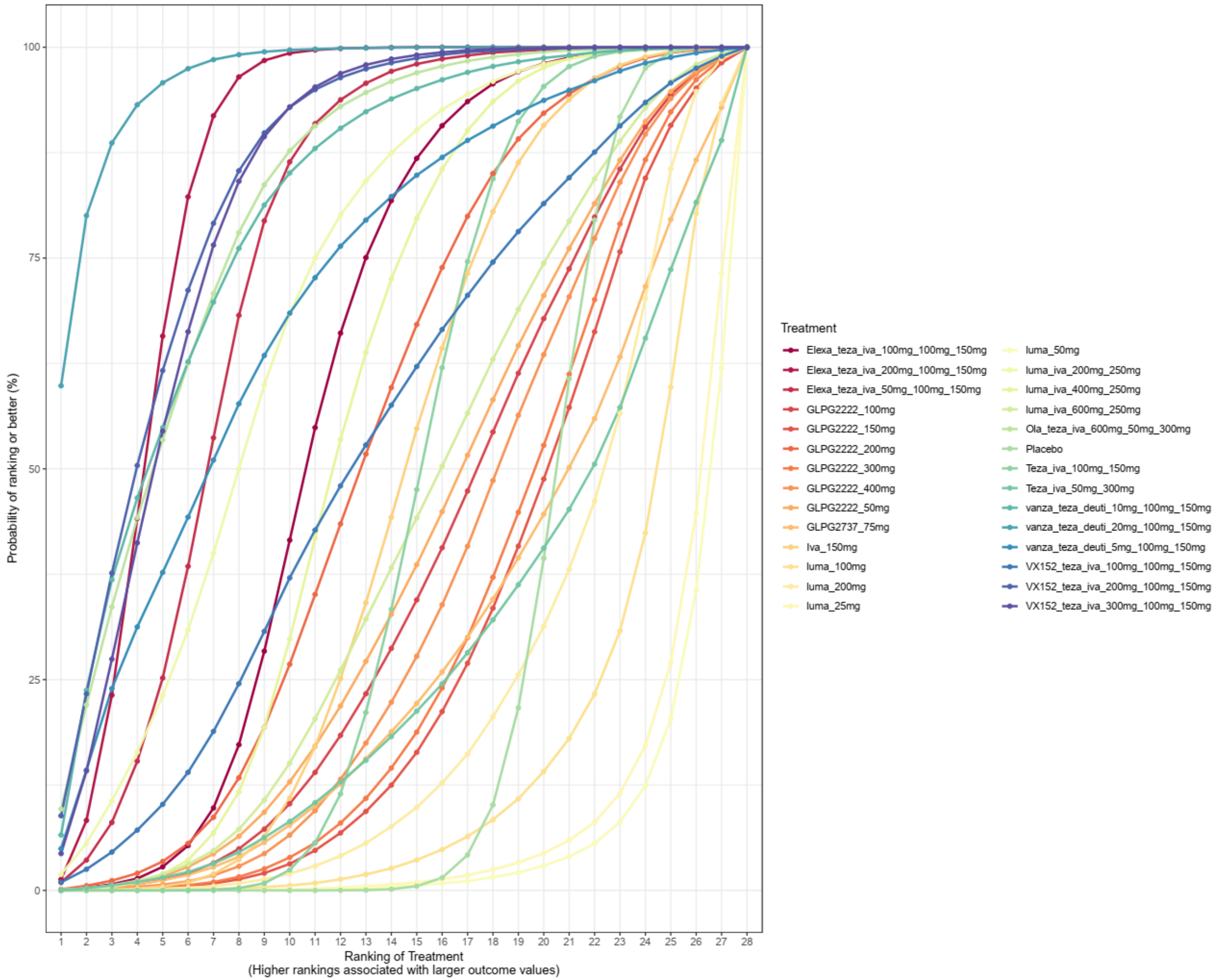

eFigure 28 SUCRA plot of CFQ-R in A) Adults treated for greater than 8 weeks (excluding flume et al.) B) Adults treated for greater than 8 weeks C) Adults treated for 4 to 8 weeks

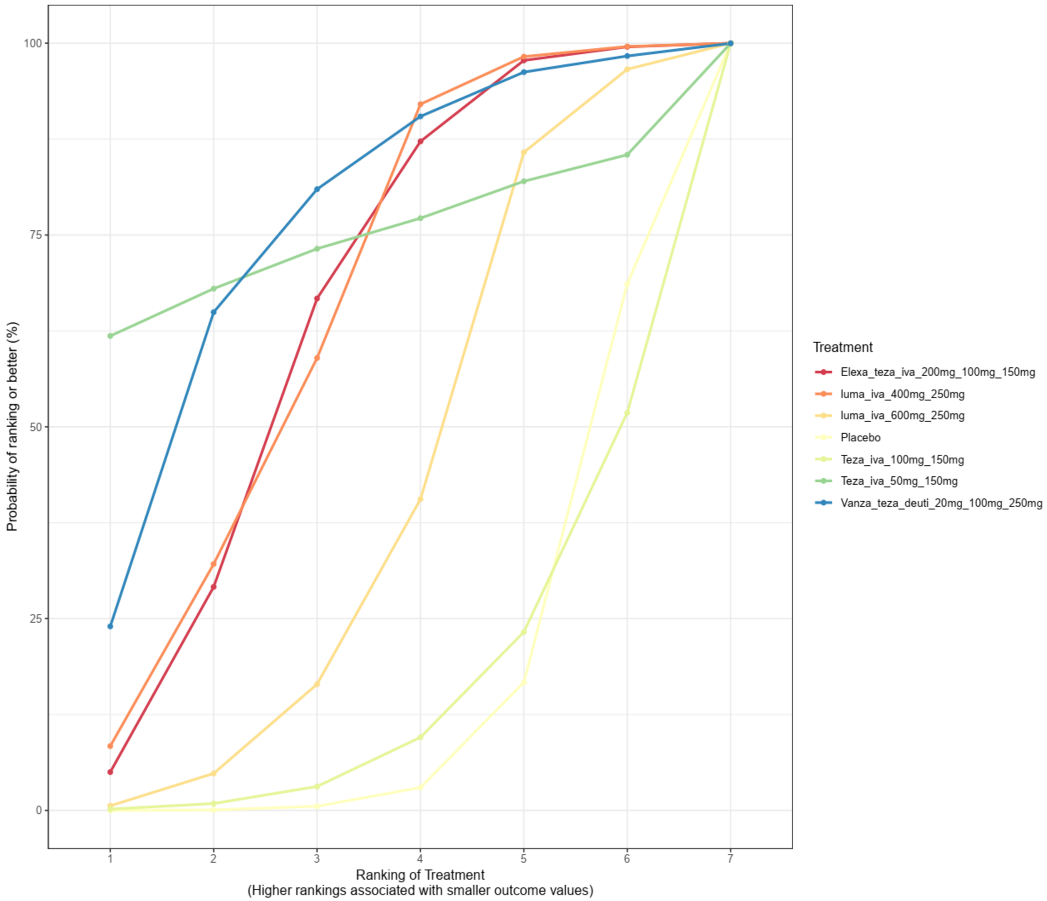
Probability of ranking or better (%)
100
75
50
25
0
1
2
3
4
5
6
7
Ranking of Treatment
(Higher rankings associated with smaller outcome values)
Treatment
Elexa_teza_iva_200mg_100mg_150mg
luma_iva_400mg_250mg
luma_iva_600mg_250mg
Placebo
Teza_iva_100mg_150mg
Teza_iva_50mg_150mg
Vanza_teza_deuti_20mg_100mg_250mg

A)

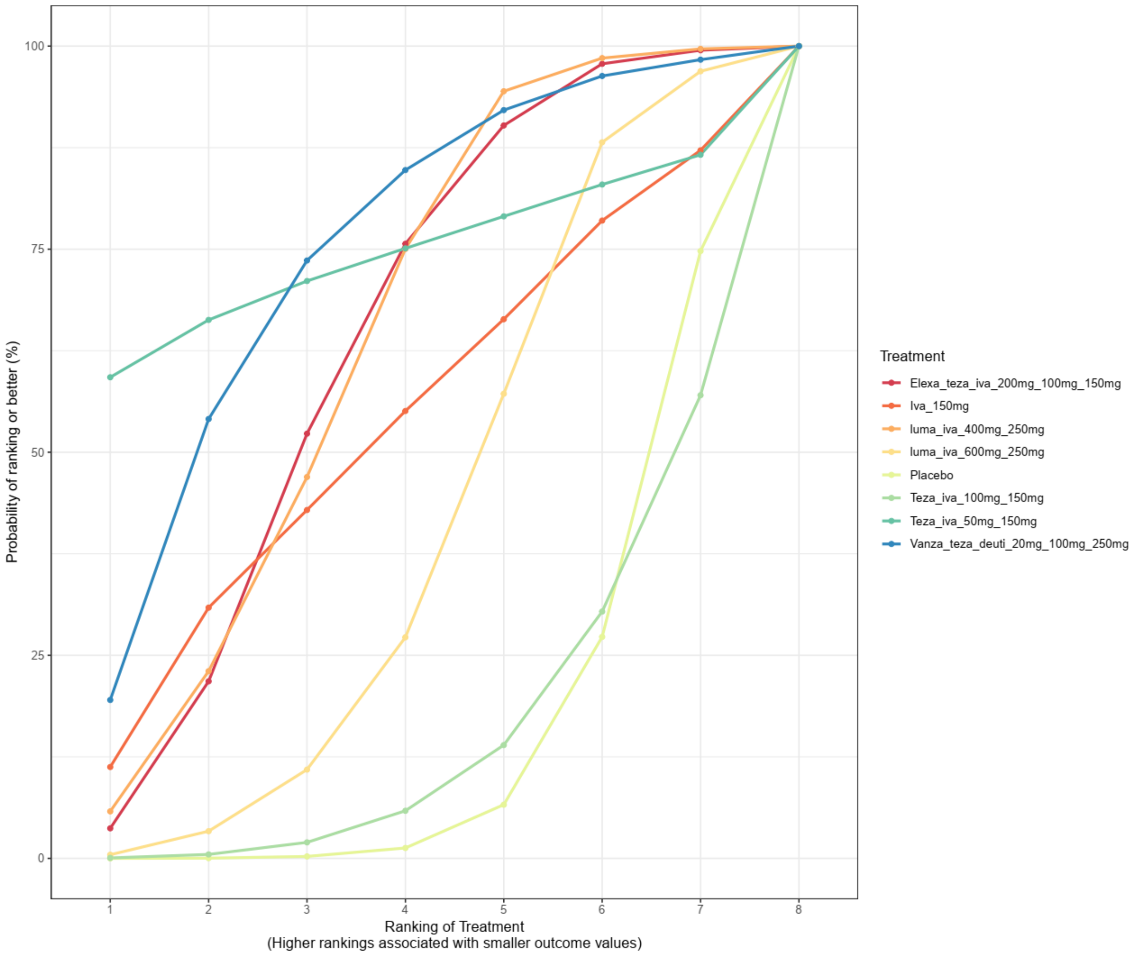
Probability of ranking or better (%)
100
75
50
25
0
1
2
3
4
5
6
7
8
Ranking of Treatment
(Higher rankings associated with smaller outcome values)
Treatment
Elexa_teza_iva_200mg_100mg_150mg
Iva_150mg
luma_iva_400mg_250mg
luma_iva_600mg_250mg
Placebo
Teza_iva_100mg_150mg
Teza_iva_50mg_150mg
Vanza_teza_deuti_20mg_100mg_250mg

B)

C)

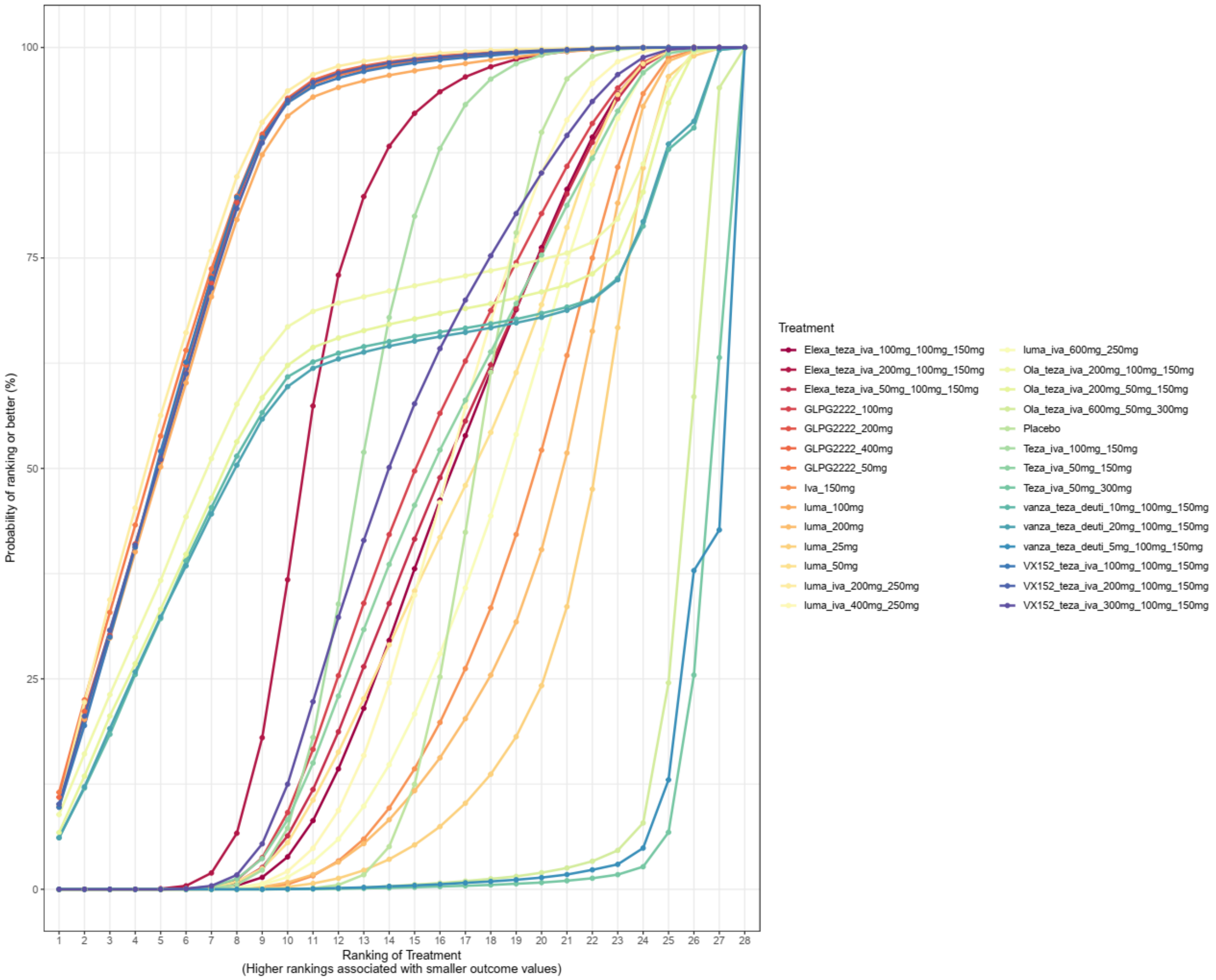

eFigure 29 SUCRA plot of serious adverse event in A) Adults treated for greater than 8 weeks (excluding flume et al.) B) Adults treated for greater than 8 weeks C) Adults treated for 4 to 8 weeks

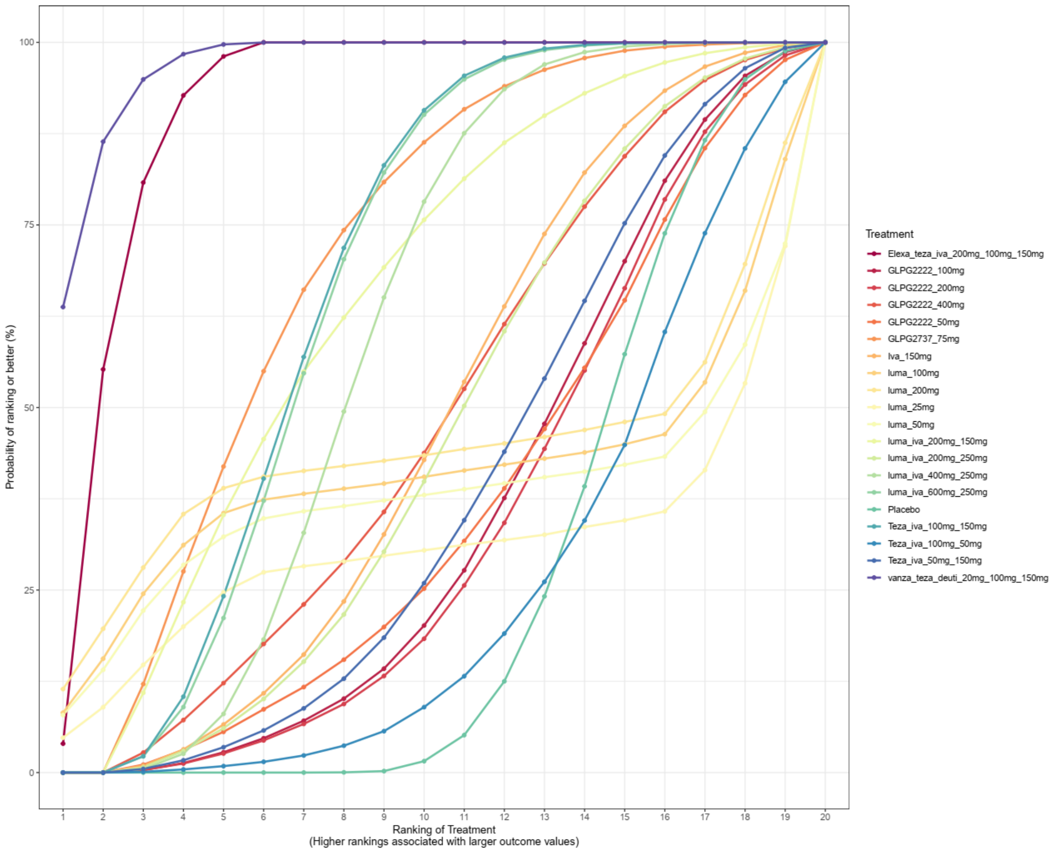

A)

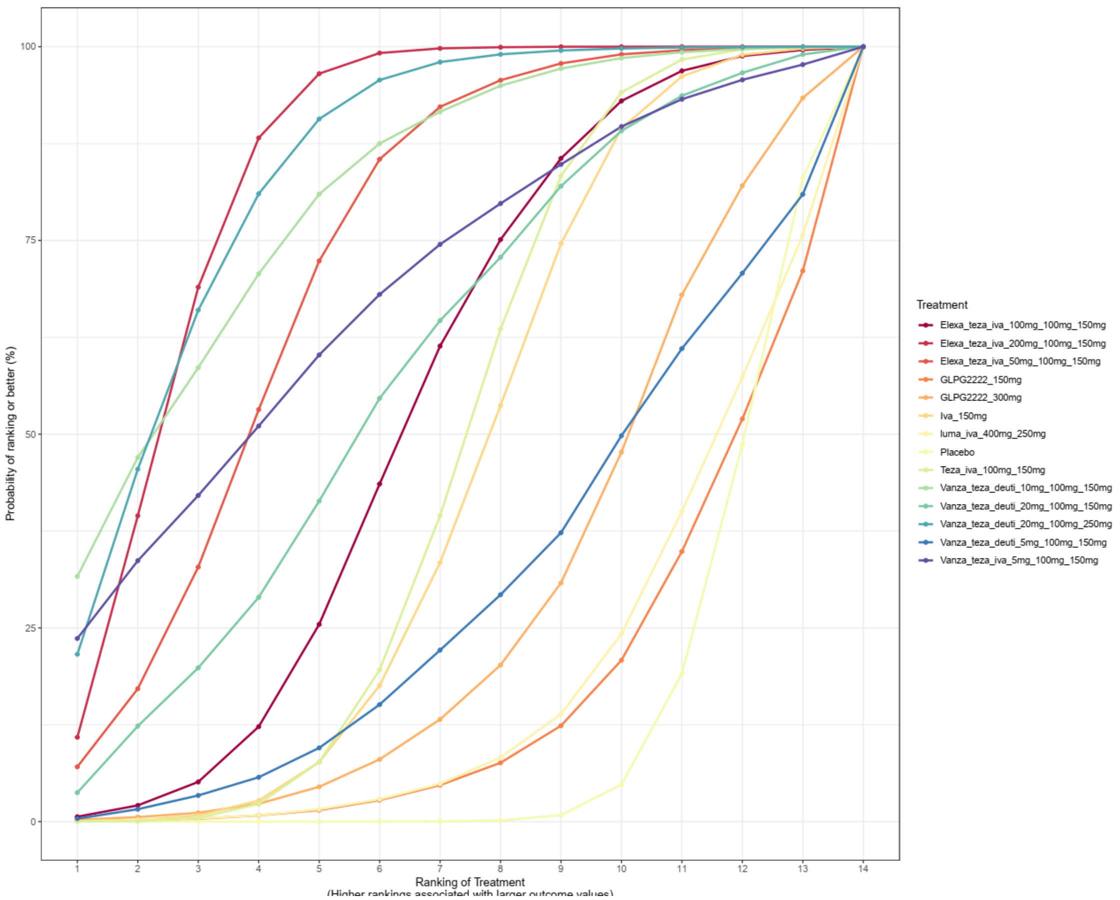

B)

eFigure 30 SUCRA plot of ppFEV$_1$ in adults A) homozygous to phe508del mutation B) heterozygous to phe508del mutation

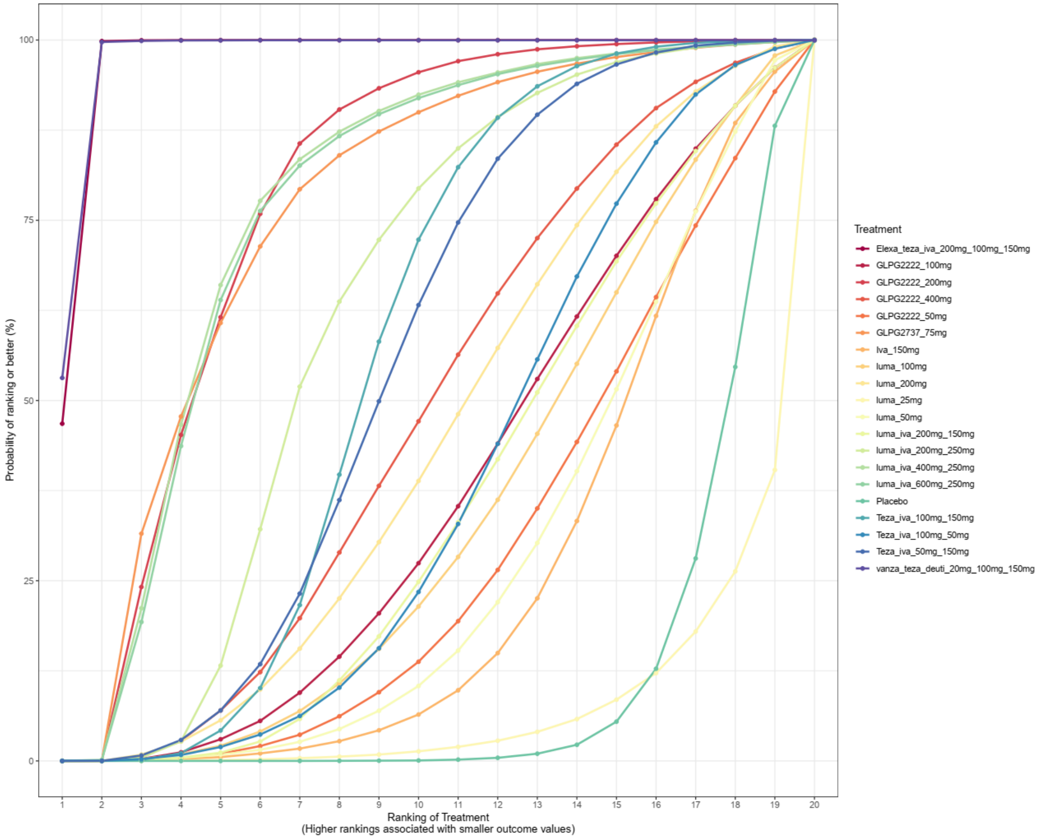

A)

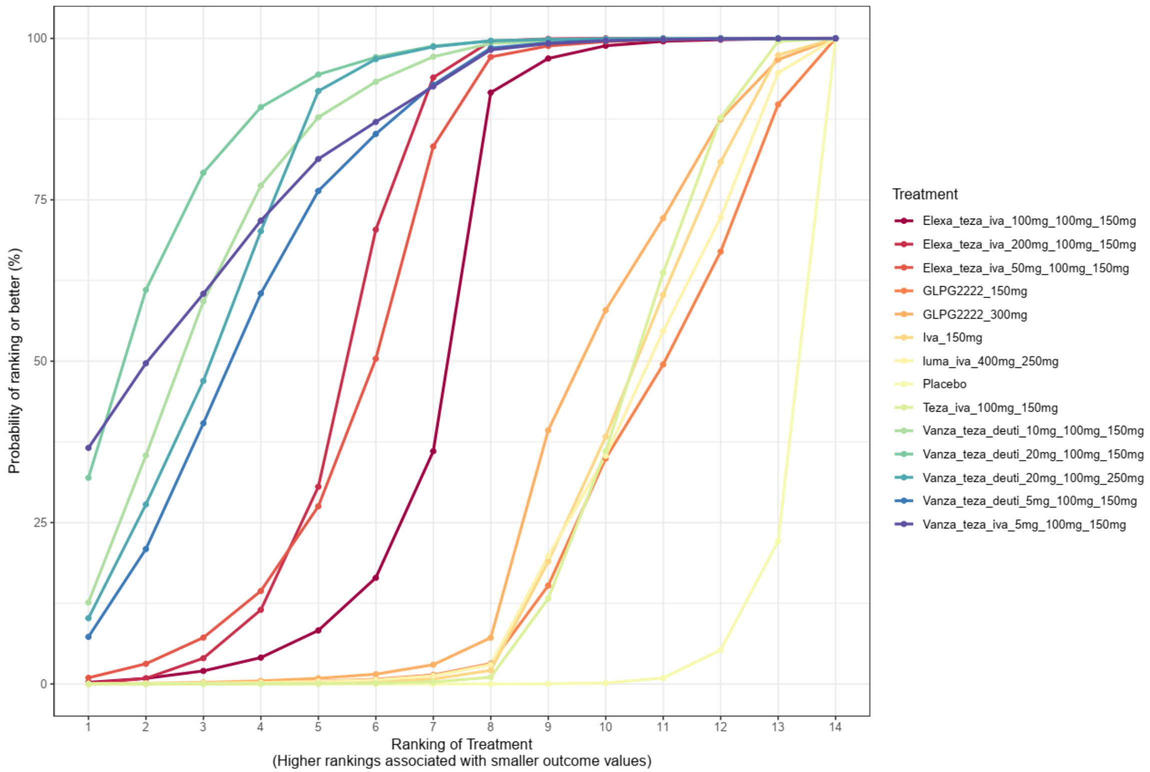

B)

eFigure 31 SUCRA plot of sweat chloride in adults A) homozygous to phe508del mutation B) heterozygous to phe508del mutation

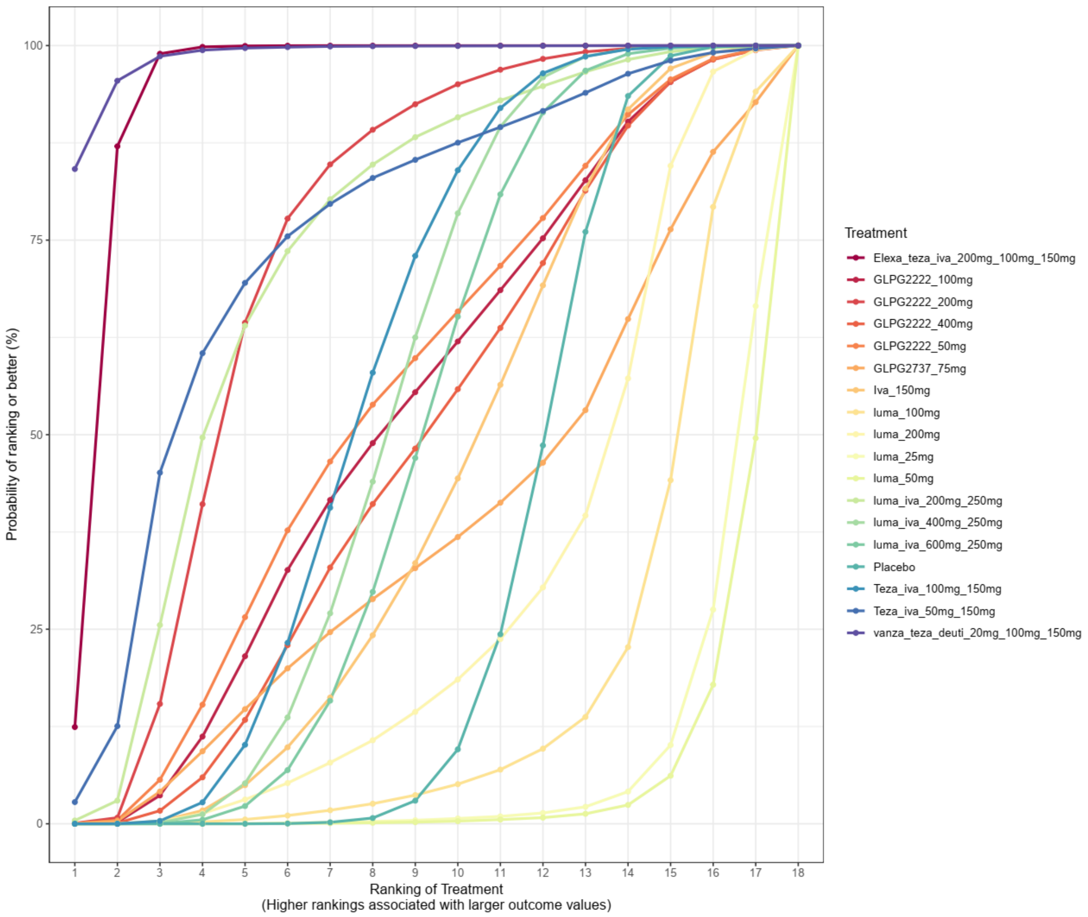

A)

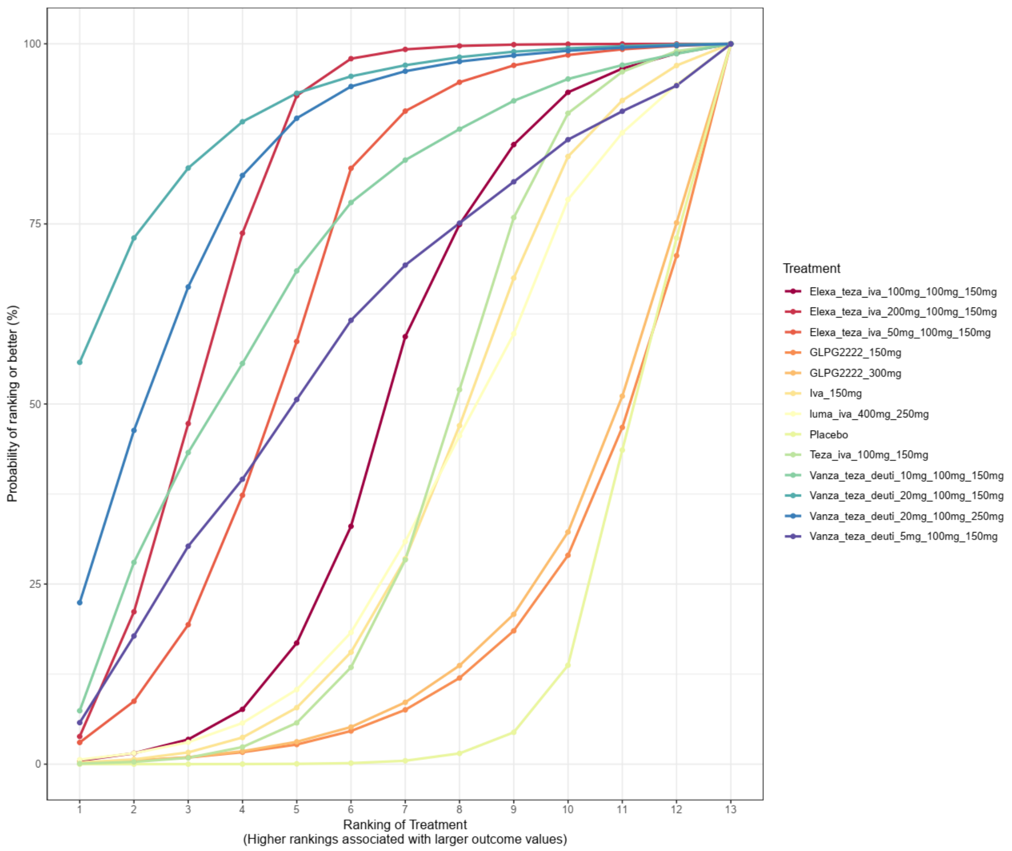

B)

eFigure 32 SUCRA plot of CFQ-R in adults A) homozygous to phe508del mutation B) heterozygous to phe508del mutation
